# All-Heteroatom-Substituted Carbon Spiro Stereocenters: Synthesis, Resolution, Enantiomeric Stability, and Absolute Configuration

**DOI:** 10.1021/jacs.5c06394

**Published:** 2025-06-04

**Authors:** Olivier Viudes, Céline Besnard, Alexander F. Siegle, Oliver Trapp, Thomas Bürgi, Gennaro Pescitelli, Jérôme Lacour

**Affiliations:** †Department of Organic Chemistry, 27212 University of Geneva, Quai Ernest Ansermet 30, 1211 Geneva 4, Switzerland; ‡ Laboratory of Crystallography, University of Geneva, Quai Ernest Ansermet 24, 1211 Geneva 4, Switzerland; § Department of Chemistry, 9183 Ludwig-Maximilians-University Munich, Butenandtstr. 5-13, Munich 81377, Germany; ⊥ Dipartimento di Chimica e Chimica Industriale, University of Pisa, Via G. Moruzzi 13, 56124 Pisa, Italy; ∥ Department of Physical Chemistry, 27212 University of Geneva, Quai Ernest Ansermet 30, 1211 Geneva 4, Switzerland

## Abstract

Chiral tetra-heterosubstituted methanes (i.e., tetraoxa and azatrioxa carbon spiro stereocenters) are synthesized under CpRu catalysis, using cyclic carbonates and carbamates as substrates and α-diazo-β-ketoesters as reagents. Single enantiomers, isolated by chiral stationary phase chromatography, display chiroptical properties, from *g*_abs_ ∼10^–5^ to ∼10^–4^, which, together with TD-DFT calculations, provide robust absolute configuration assignments. Crystalline spiro diastereomers were also obtained, confirming further the structural and configurational assignments. Using enantioselective dynamic chromatography, remarkable enantiomerization barriers were determined for the ortho-carbonates and ortho-carbamates, with values of up to 27.6 and 34.6 kcal/mol (half-lives 227 days and >84,000 years at 25 °C, respectively). DFT further elucidates the origin of this large difference pointing toward preferred C–O or C–N bond cleavages in the rate-determining step of the S_N_1-like mechanism.

## Graphical abstract

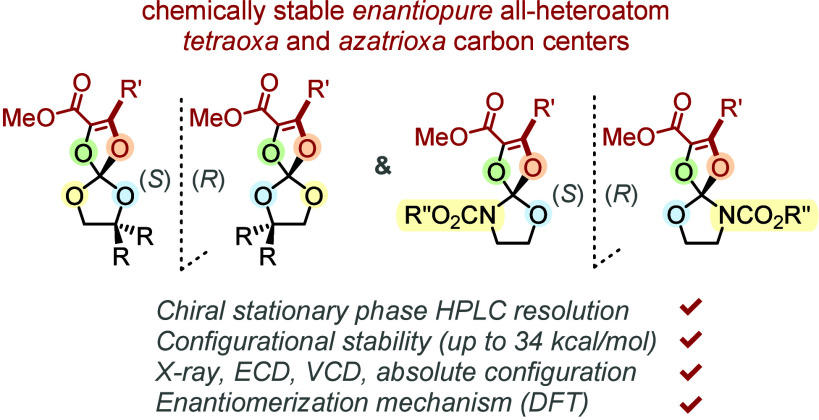

## Introduction

Chirality, defined as “*the geometric property of a rigid object* [***···***] *of being non-superposable on its mirror image*”,^1^ is a fundamental property encountered at many levels in chemistry (molecular, supramolecular, coordination, polymer, clusters, surfaces, etc.) and in many different forms denominated usually as center, axial, planar, or helical.^2^ This scientific domain is essential to biology, pharmacology, and physics alike.^3^ Chirality centers are a generalized extension of the concept of stereogenic carbon atoms often abbreviated as Cabcd (**1**, Figure 1A) or C­(ab)­(a′b′) of spiro geometry (**2**, Figure 1B).^4^ They exist as pairs of enantiomers defined by their absolute configuration (i.e., by the spatial arrangement of the atoms with stereochemical descriptors (*S*) and (*R*)). Some of these moieties represent the most fundamental forms of molecular chirality (e.g., the chiral methyl group **3**,^5^ the butylethyl­methylpropyl­methane **4**,^6^ the isotopically differentiated 2,2′-dimethylpropane **5**,^7^ or the bromochloro­fluoromethane **6**).^6^ These derivatives have further tested our ability to detect and determine their absolute configuration.^8^ While it was possible to generate and study the enantiomers of **6**,^6^ the all-heteroatom bromochloro­fluoroiodomethane analog **7** cannot be accessed synthetically. In fact, to our knowledge, tetrasubstituted all-heteroatom carbon centers have never been reported in configurationally stable forms (half-life > 1000 s at 25 °C).^9^ Only spiro **8**, made of two benzothiazole rings, was reported as a configurationally labile derivative.

**Figure 1:**
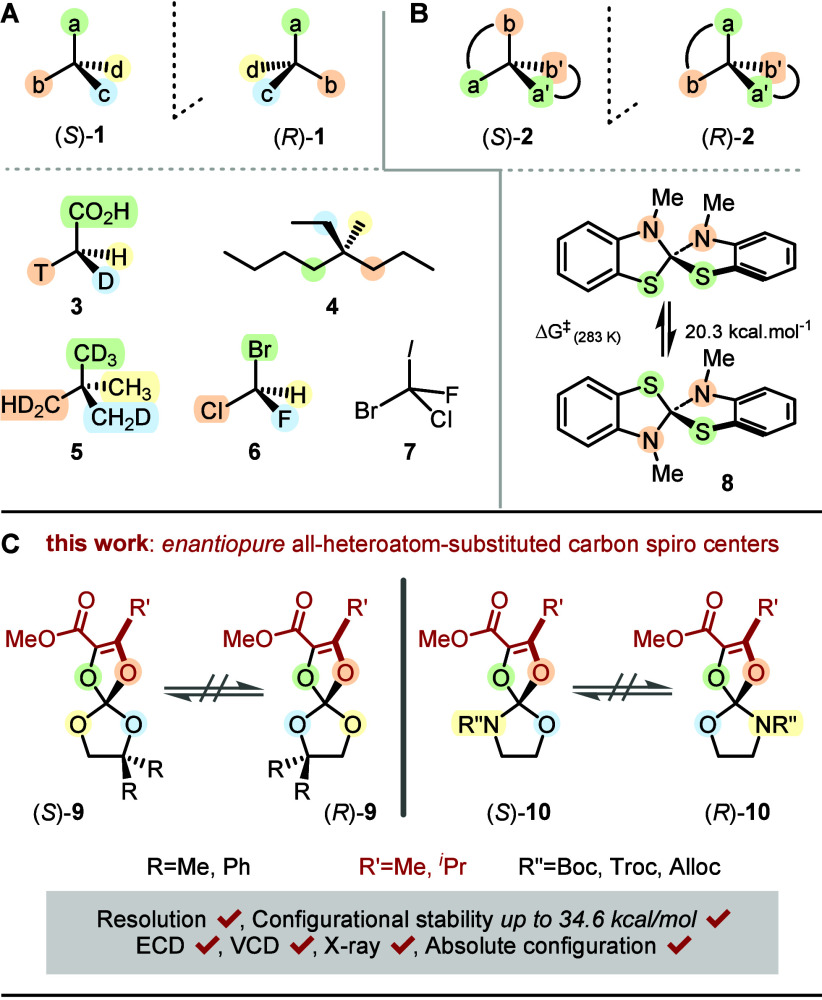
Carbon stereocenters. (A) Type Cabcd (priority a > b > c > d, *S* and *R* enantiomers) and key examples **3**–**7**. (B) Spiro C­(ab)­(a′b′) (a > a′ > b > b′) and unique configurationally labile diazadithia **8**. (C) All-heteroatom-substituted spiro stereocenters: tetraoxa **9** and azatrioxa **10**.

The enantiomers of **8**, separated by chiral stationary phase (CSP) high-performance liquid chromatography (HPLC) at low temperature, racemize relatively rapidly at 25 °C (enantiomerization barrier ΔG^⧧^_(283 K)_ 20.3 kcal/mol, half-life 560 s at 10 °C).^10^ Herein, in an effort to expand this domain of stereochemistry with novel targets for stereoselective synthesis, medicinal chemistry,^11^ and, also importantly, chiroptical studies,^8^ we report the direct synthesis of spiro stereocenters (Figure 1C) of types **9** (tetraoxa) and **10** (azatrioxa) using α-diazo-β-ketoesters **11** and either cyclic carbonates **12** or carbamates **13** as substrates (Figures 2A and 3A). Under CpRu catalysis, at moderate temperature (60 °C), diazo decomposition and carbonyl-ylide reactivity provide bis spiro products **9** or **10** in good to excellent yields (Figures 2–5, up to 98%). NMR and isolated yields are very similar for the various spiro constructs. Then, evidence of configurational stability was provided by the enantiomeric separation of **9a** (R, R′ = Me) and **10a** (R′ = Me, R′′ = Boc) using CSP-HPLC. Dynamic gas chromatography (DGC) analysis^12^ of *rac*-**9b** (R = Me, R′ = ^*i*^Pr, 140–180 °C) and *rac*-**10b** (R′ = ^*i*^Pr, R′′ = Boc, 105–125 °C) confirmed the results with enantiomerization barriers, ΔG^⧧^_(298 K)_, reaching values of up to 27.6 and 34.6 kcal/mol (half-lives 227 days and >84,000 years at 25 °C, respectively). DFT elucidates the S_N_1 mechanism of the enantiomerization and affords an explanation for the large configurational stability difference. Levo- and dextrorotatory enantiomers (*ee* > 99%) were further studied by electronic circular dichroism (ECD). While low levels of the chiroptical response were observed for cryptochiral moieties **9** carrying four oxygen atoms around the spiro center (*g*_abs_ ±5 × 10^–5^), a strong enhancement was obtained in azatrioxa series **10** representing a 10-fold gain in the dissymmetry factor (*g*_abs_ ±5 × 10^–4^). In all cases, ECD experiments were allied with robust absolute configuration assignment by TD-DFT calculations. Also, X-ray crystallographic analyses of diastereomeric spiro **9e′** and **9e′′** derived from (−)-2,3-pinanediol were achieved. Of note, the rigid chiral terpane skeleton strongly influenced the chiroptical properties of product **9e** at the expense of the weakly discriminating tetraoxa center. This was not the case anymore with diastereomers **10i′**/**10i′′** and **10j′**/**10j′′** derived from (+)-valinol with a chiroptical predominance of the azatrioxa spiro center over the chiral oxazolidine framework. Relative and absolute configurations were further determined by X-ray, ECD, and vibrational circular dichroism (VCD).

**Figure 2:**
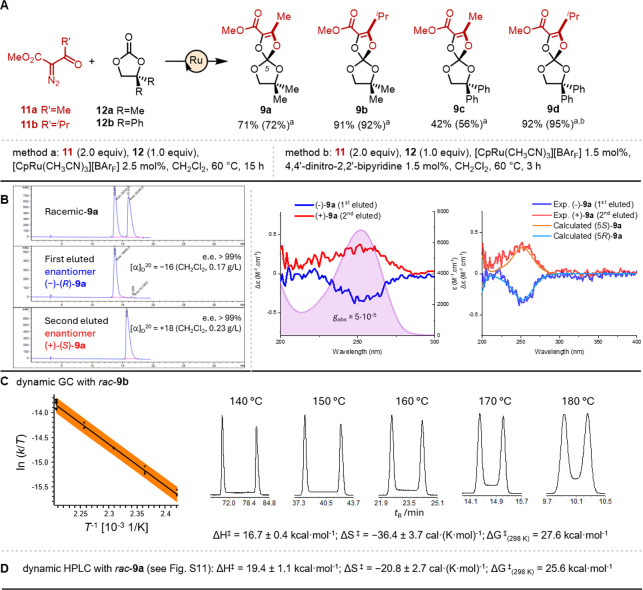
Ortho-carbonates. (A) Synthetic procedure (methods a and b) and substrate scope: **9a** to **9d**. Isolated yields (NMR yields in parentheses). (B) CSP-HPLC analysis of **9a** (racemic and enantiomers) and ECD spectra of (−)-**9a** (blue) and (+)-**9a** (red) with absorption spectra (underlying filled curve) in the UV range. Right panel: comparison between experimental and calculated ECD spectra. (C) Enantioselective DGC (140–180 °C) and Eyring analysis of the temperature-dependent kinetics. The orange band represents the confidence interval of the linear regression with a confidence level of 95%. (D) Activation parameters for the enantiomerization of *rac*-**9a** (Figure S12).

**Figure 3:**
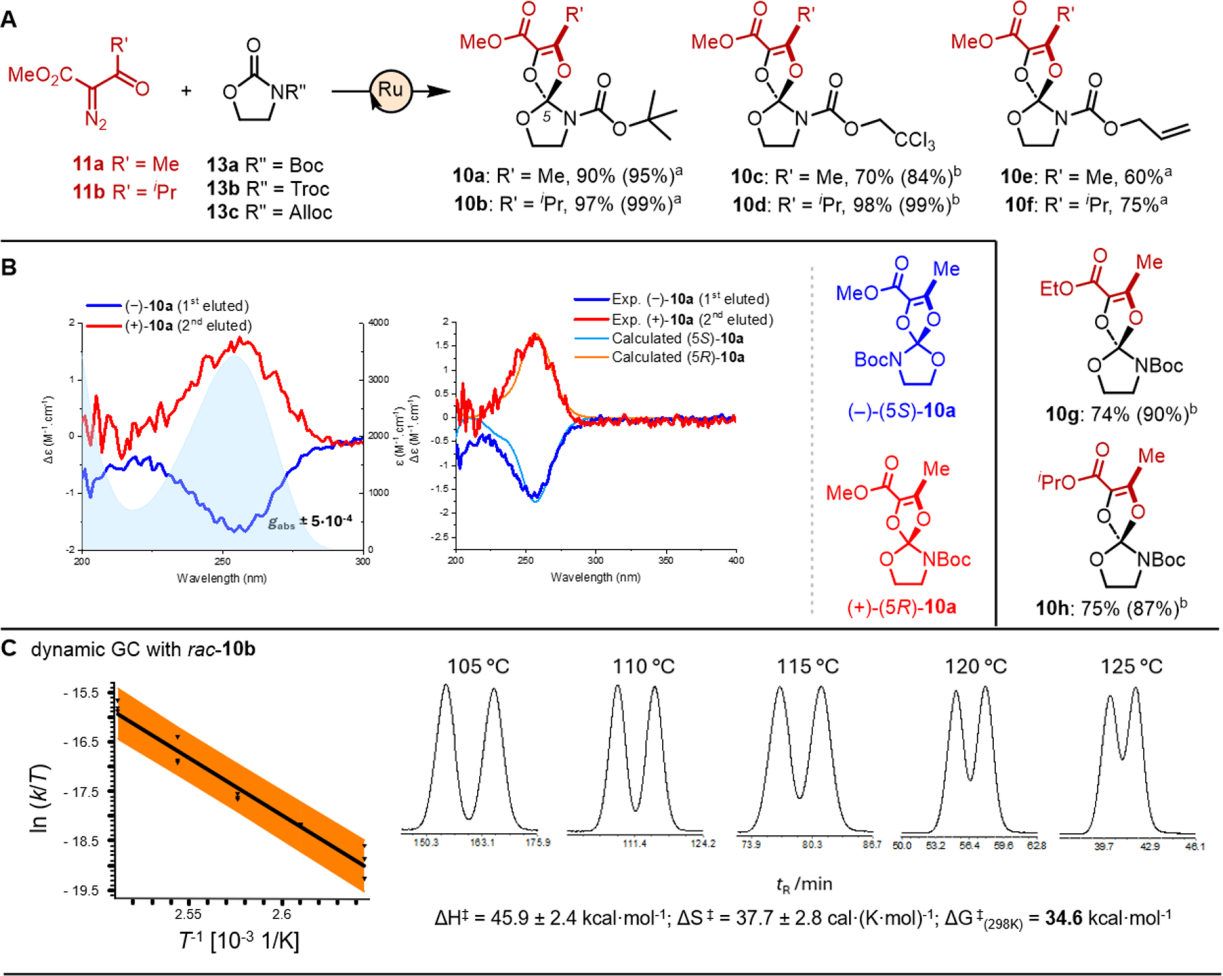
Ortho-carbamates. (A) Synthetic procedure (methods a and b, see Figure 2 for details) and reactivity scope: **10a** to **10h**. Isolated yields (NMR yields in parentheses). (B) ECD spectra of (−)-**10a** (blue) and (+)-**10a** (red) with absorption spectra (underlying filled curve) in the UV range. Comparison between experimental and calculated ECD spectra and configuration assignment. (C) Enantioselective DGC of **10b** (105–125 °C) and Eyring analysis of the temperature-dependent kinetics. The orange band represents the confidence interval of the linear regression with a level of confidence of 95%.

## Results and Discussion

Ortho-carbonates are relatively uncommon tetraoxa-substituted carbon centers that can display good chemical stability.^10a,13^ Among various strategies for their synthesis, a procedure reported by our group providing unsaturated tetraoxa derivatives in good to excellent yields was selected;^14^ the resulting spiro products are easily isolable by chromatography over silica gel. Chiral ortho-carbonates **9** were hence synthesized by the condensation of α-diazo-β-ketoesters **11** (2 equiv)^15^ and cyclic carbonates **12** (1 equiv) under [CpRu­(N≡CCH_3_)_3_]­[BAr_F_][Bibr ref16] (**14**) catalysis (Figure 2A). The mild Lewis acidic conditions, promoted by the ruthenium complex, favored both diazo decomposition^17^ and carbonyl-ylide formation^18^ to provide the bis spiro constructs in good to excellent yields (up to 92%).^14,19^ A mechanistic rationale is proposed in the Supporting Information (Figure S1). In practice, at 60 °C, a concentrated solution of **12a** (1 M, CH_2_Cl_2_),^10b^ catalyst **14** (2.5 mol %), and **11a** (2.0 equiv) afforded product **9a** in 71% isolated yield (method a). Reacting **11b** carrying an isopropyl residue next to the carbonyl group was beneficial as both isolated and NMR yields increased significantly to 91%. These conditions were, however, challenging when applied to diphenyl derivatives **9c** and **9d**. In the first case, the incomplete conversion (56%) of precursor **12b** ^20^ was observed, so only a 56% NMR yield of **9c** was obtained. In addition, chromatographic separation (SiO_2_) of spiro **9c** from **12b** was difficult, resulting in a low isolated yield (42%). These reactivity and separation issues remained for the preparation of **9d** but to a lower extent (86% conversion, 86% NMR and 62% isolated yields). Driving the reaction to completion thanks to the addition of electron-poor ligand 4,4′-dinitro-2,2′-bipyridine (method b) was beneficial to yielding **9d** in 95% NMR and 92% isolated yields, respectively.

All orthocarbonates **9** demonstrated remarkable bench and solution robustness in organic solvents (e.g., DCM, DMSO, and MeOH). Their configurational (optical) stability being unproven, the enantiomeric resolution of racemic **9a** was attempted by semipreparative CSP-HPLC. Optimal separation conditions (CHIRALPAK IG, Figure 2B) could be found and afforded levo- and dextrorotatory enantiomers as first and second eluted fractions in excellent enantiomeric purity (*e.r*. > 99.5:1, Figures S2 and S3). In our hands, the enantiomers were configurationally stable at 25 °C in solutions from apolar (toluene) to polar (acetonitrile) conditions. ECD spectra displayed mirror-image curves in the UV region (Figure 2B). Their detailed analysis allied with the absolute configuration assignment is discussed later (*vide infra*).

Care was then taken to determine the configurational stability of the *all*-O spiro stereocenter and the barrier of enantiomerization by dynamic chromatography specifically.^12g,21^ Using *rac*-**9b** as an analyte and heptakis­(2,3-di-O-acetyl-6-O-*tert*-butyldimethylsilyl)-β-cyclodextrin^22^ as the CSP in DGC, peak profiles were obtained from 140 to 180 °C (Figures 2C and S10). Then, the chromatograms, characterized by peak broadening and plateau formations, were evaluated to obtain the apparent rate constants (considering the enantiomerization process in the gas and stationary phases) for the on-column *R*–*S* enantiomerization. From these values, the corresponding enantiomerization parameters were calculated,^23^ whose values are ΔH^⧧^ = 16.7 ± 0.4 kcal·mol^–1^, ΔS^⧧^ = −36.4 ± 3.7 cal·(K·mol)^−1^, and ΔG^⧧^_(298 K)_ = 27.6 kcal·mol^–1^. Analogous studies were conducted for *rac*-**9a** using CSP-HPLC (CHIRALPAK IG-3, 60–80 °C, Figure S12), as chiral separations could not be achieved by CSP-GC. Slightly different activation parameters were obtained in solution: ΔH^⧧^ = 19.4 ± 1.1 kcal·mol^–1^, ΔS^⧧^ = −20.8 ± 2.7 cal·(K·mol)^−1^, and ΔG^⧧^_(298 K)_ = 25.6 kcal·mol^–1^ (Figure 2D). Despite being measured near the upper limit of the HPLC temperature range, these values for **9a** are consistent with that of **9b**, confirming the configurational stability of the spiro products.

With these results in hand, the absolute configuration assignment was primarily realized by electronic circular dichroism (ECD).^24^ Following a well-established computational protocol,^25^ the conformational landscape of compound **9a** was explored by a molecular mechanics conformational search and all structures thus found were optimized by DFT at the B3LYP-D3BJ/6-311+G­(d,p) level using a polarizable continuum solvent model (PCM) for acetonitrile. Four low-energy minima were obtained, with populations ranging from 20 to 31% at 300 K (Table S1 and Figure S13), differing in the *s*-*cis*/*s*-*trans* orientation of the ester moiety and in the puckering of the 1,3-dioxolane ring. TD-DFT calculations run at the CAM-B3LYP/aug-cc-pVTZ/PCM level led to a weighted-average ECD spectrum with a positive band allied to the enoate π–π* transition at around 250 nm for the 5*S* configuration, corresponding to the second eluted (+)-enantiomer (Figure 2B). The calculated *g*_abs_ values were in good agreement with the experiment (±3 × 10^–5^ vs ±5 × 10^–5^), confirming the cryptochiral character of compound **9a**.

Ortho-carbamates are compounds in which central carbons are bound to one nitrogen atom and three oxygen atoms. These derivatives are clearly less common than ortho-carbonates, sometimes isolated as byproducts of reactions.^26,27^ To our knowledge, a general methodology to access such a structural motif in a predictive manner has not been reported so far, particularly for spiro compounds. It was hence tempting to apply previously detailed catalytic conditions to cyclic carbamates **13**. Chiral ortho-derivatives **10** were thus synthesized by the condensation of α-diazo-β-ketoesters **11** (2 equiv) and substrates **13** (1 equiv) under CpRu **14** catalysis (Figure 3A). The first attempt by the addition of a concentrated solution of **13a** (R′′ = Boc, 1 M, CH_2_Cl_2_) to catalyst **14** (2.5 mol %) with **11a** or **11b** (2.0 equiv) at 60 °C afforded products **10a** (R′ = Me, 90%) and **10b** (R′ = ^*i*^Pr, 97%) in excellent yields using method a, as detailed in Figure 2A. Following this positive result, oxazolidinone **13b** (R′′ = Troc) was tested and afforded isolated yields of 70% for **10c** and 98% for **10d**. In this case, method b was necessary to ensure the complete conversion of more hindered isopropyl diazo **11b**. With carbamate **13c** (R′′ = Alloc), isolation of carbene addition products **10e** (R′ = Me) and **10f** (R′ = ^*i*^Pr) was more challenging. The milder conditions of method a were favorable, yielding **10e** and **10f** in 60 and 75% isolated yields, respectively. Of note, competing cyclopropanation was not observed on this unsaturated substrate. The lack of olefin reactivity seems to be a trait of Alloc derivatives under CpRu catalysis.^19,28^ Finally, two additional diazo reagents analogous to **11a** (R′ = Me) but with ethyl and isopropyl ester groups (instead of CO_2_Me) were prepared, and the formation of products **10g** and **10h** resulted in good yields (74 and 75%, respectively). In these two instances, NMR yields were higher (87 and 90%) but isolation of the desired spiro derivatives **10g** and **10h** from byproducts generated upon Wolff rearrangement of the carbenes was difficult.^29^ The scalability of the process was furthermore investigated with the formation of ortho-carbamate **10b** in 96% yield on a 1 mmol scale (317 mg, method a, eq S1).

Remarkable bench stability and solution robustness in organic solvents (e.g., DCM, DMSO, and MeOH) were again noticed for all ortho-carbamates **10**. Handling of these products was comparable to that of orthocarbonates **9**, and it did not require particular precautions. The enantiomeric resolution of *rac*-**10a** was attempted by semipreparative CSP-HPLC. A successful separation provided (−)-**10a** and (+)-**10a** as first and second eluted enantiomers, respectively, with excellent levels of enantiomeric purity for both fractions (*e.r*. > 97:3).^30^ Absorption spectra were recorded in acetonitrile and revealed, as previously noted for ortho-carbonates, a predominant band with a maximum at around 255 nm (Figure 3B). ECD spectra were next recorded. Enantiomers (−)-**10a** and (+)-**10a** displayed mirror-image spectra presenting negative and positive Cotton effects, respectively (Figure 3B). Importantly, and as initially expected, stronger dissymmetry factors were determined (*g*_abs_ = −4.7 and +4.6 × 10^–4^). These values are ten times higher than the dissymmetry factors of ortho-carbonates **9a**. ECD calculations were run on **10a** using the same procedure as detailed above (Table S4) and allowed us to assign the 5*R* configuration to the second eluted (+)-enantiomer (Figure 3B). The calculated *g*_abs_ values were ±2 × 10^–4^, reproducing the experimentally observed trend between ortho-carbonates and ortho-carbamates. The main band at 250 nm preserves the same enoate π–π* character with little participation from the carbamate group (Supporting Information).

Finally, care was taken to measure the enantiomerization barrier of ortho-carbamates **10** and establish another important difference with ortho-carbonates **9**. Among several derivatives tested, only *rac*-**10b** could be analyzed successfully by dynamic chromatography with heptakis­(2,3-di-O-methyl-6-O-*tert*-butyldimethylsilyl)-β-cyclodextrin as a CSP in GC. Different peak profiles were afforded from 105 to 125 °C (Figures 3C and S11). The chromatograms, characterized by peak broadening and plateau formations from the initial temperature value of 105 °C, afforded apparent rate constants for the on-column *R*–*S* interconversion process. From these values, the enantiomerization parameters were calculated,^23^ whose values are ΔH^⧧^ = 45.9 ± 2.4 kcal·mol^–1^, ΔS^⧧^ = 37.7 ± 2.8 cal·(K·mol)^−1^, and ΔG^⧧^_(298 K)_ = 34.6 kcal·mol^–1^. This corresponds to a remarkably high Gibbs free energy of activation and, considering a first-order enantiomerization mechanism (*vide infra*), a half-life of >84,000 years at 298 K.

### Enantiomerization Mechanism

As just shown, ortho-carbonates **9** and ortho-carbamates **10** present high to very high configurational barriers at 298 K, from 27.6 to 34.6 kcal·mol^–1^ as measured by VT-GC. The data well fit a dissociative S_N_1-like pathway with C–O (or C–N) bond cleavages as first steps, generating anionic O or N leaving groups in the rate-determining steps of the enantiomerization process.^10a^

Of all of the possible intermediates, enolates stabilized by conjugation with the electron-withdrawing ester groups were deemed to be the most likely. DFT calculations were conducted to confirm this hypothesis. To ensure a full mechanistic picture, dissociation of all C–O (or C–N) bonds was considered, leading to four possible zwitterionic intermediates of types **I**, **II**, **III**, and **IV** for structures **9a** and **10a** (Figure 4A). As expected, structures of type **I** were more stable than the others by at least 10–15 kcal·mol^–1^ (internal energy).

**Figure 4:**
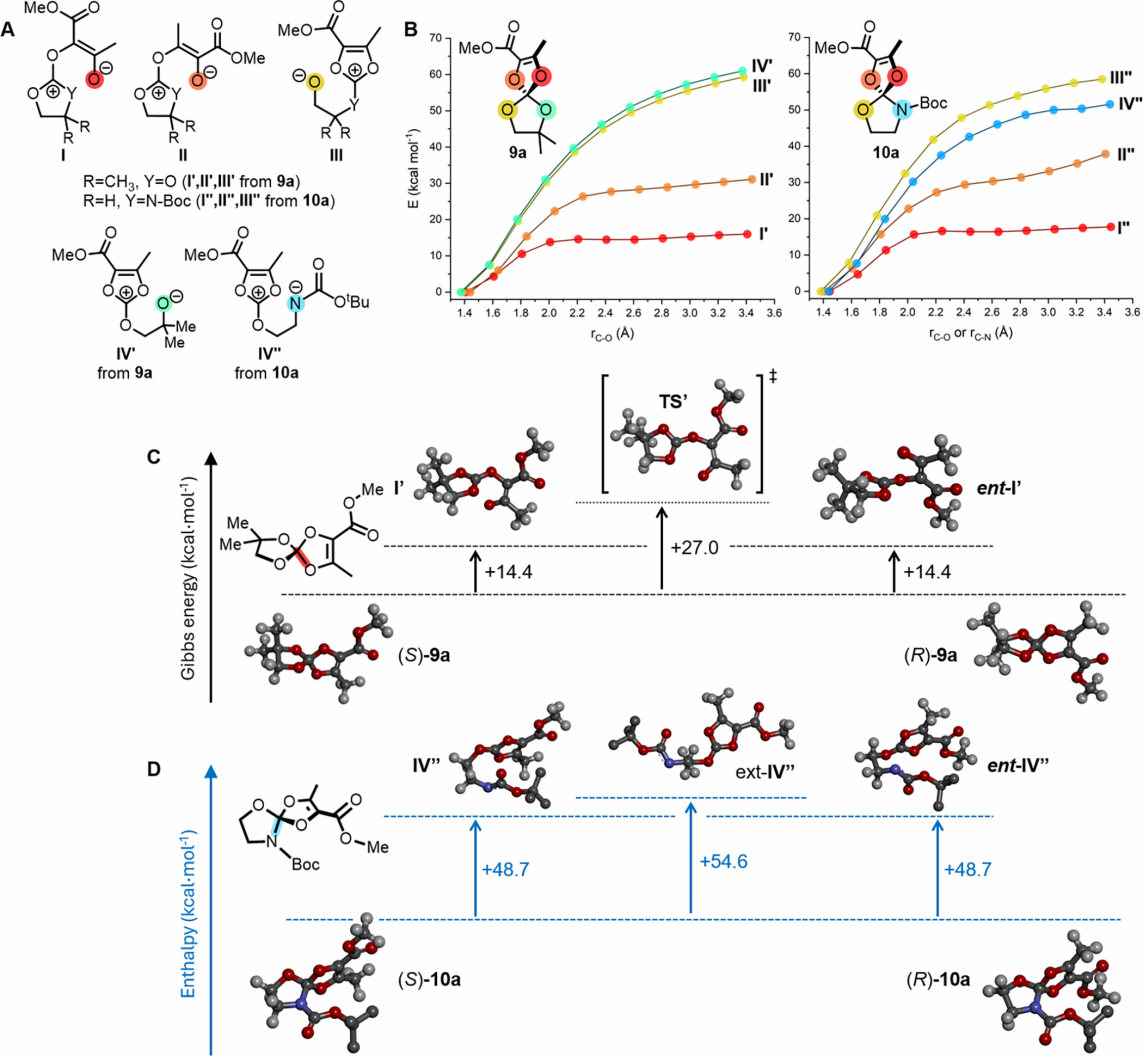
(A) Zwitterionic intermediates **I** to **IV** obtained by the dissociation of the four possible C–O or C–N bonds in **9a** and **10a**. (B) Energy scans following the dissociation of the four bonds. (C, D) Suggested enantiomerization mechanisms exemplified for **9a** and **10a**, with calculated ΔG (black) or ΔH (blue; H atoms of Boc are removed for clarity).

For ortho-carbonate **9a**, once the zwitterion **I′** has formed, it must undergo rotation around C^+^–O and/or O–C= bonds to proceed along the enantiomerization pathway through a quasi-planar transition state **TS′** (Figures 4C and S21, Supporting Information). Its geometry allows for extended conjugation over all sp^2^-hybridized atoms (see frontier MOs in the Supporting Information) at the expense of angle and steric strain. The calculated Gibbs energy for the whole process of **9a** (ΔΔG°_**9a**/**TS′**_ = +27.0 kcal/mol) reproduces well the experimental kinetic barriers measured for **9a** and **9b**, and the rigid structure of **TS′** is in accord with the large experimental negative ΔS^⧧^.

For carbamate **10a**, the favored racemization pathway was again expected to involve the dissociation of the β-enol spiro C–O bond leading to intermediate **I′′** (Figure 4A,B). However, the very different activation parameters measured by VT-GC on **10b** with respect to **9a**/**9b** pointed to a substantially different mechanism. In fact, following the formation of **I′′**, we could not isolate any transition-state structure like **TS′**. The carbamate group adds further steric strain in the plane containing the OC­(=O)­NC^+^O_2_ moiety and adds additional cost to the angle strain necessary to achieve planarity (Figure S21).^31^ In this situation, the enantiomerization pathway proceeds through the unfavored C–N bond-breaking coordinate, leading to zwitterion **IV′′** (Figure 4B). After an almost barrierless conformational rearrangement, ring closure may then occur in a stereorandom fashion. The calculated enthalpy difference between **IV′′** in its extended conformation and **10a** (ΔΔH°_**10a**/**IV′′**_ = +54.6 kcal/mol) is comparable to the experimental racemization enthalpy barrier ΔH^⧧^ measured for **10b**. It is advisable here to consider calculated ΔH rather than ΔG values because the latter would include only the vibrational contribution to entropy but not the conformational one, which is instead expected to play a major role in the formation of **IV′′** (endowed with low-frequency vibrations) and would be difficult to evaluate in an accurate manner.^32^

### Diastereomeric Series

Finally, to determine additional structural information about compounds **9** and **10**, and reinforce the previous ECD configuration assignments, carbonates **9e′** and **9e′′** and also carbamates **10i′**, **10i′′**, **10j′**, and **10j′′** were obtained as single diastereomers, and when possible, X-ray diffraction analyses were conducted. In fact, carbonate **12c** derived from enantiopure (1*R*,2*R*,3*S*,5*R*)-pinane diol^33^ was synthesized (Figure 5A)^10b^ and used as a substrate to afford **9e** in excellent yield (90%, method a, 1.2:1 mixture of diastereomers). Chromatographic separation on regular silica gel (eluent pentane:EtOAc 97:3) afforded (2*S*,3a′*R*,4′*R*,6′*R*,7a′*S*)-**9e′** (first eluted, 43%) and (2*R*,3a′*R*,4′*R*,6′*R*,7a′*S*)-**9e′′** (second eluted, 34%) as fully separated diastereomers. The isolated compounds were heated independently in DMSO at 80 °C for 1 h without any evidence of epimerization, hence confirming the relatively high configurational stability of such spiro centers, even in a strongly polar (dissociating) solvent. For **9e′** and **9e′′**, crystals suitable for measurements were obtained by slow evaporation of *n*-pentane solutions (Figure 5A). The corresponding X-ray crystal structures were determined by establishing without ambiguity the relative configurations and confirming the absolute configurations of the products.

**Figure 5:**
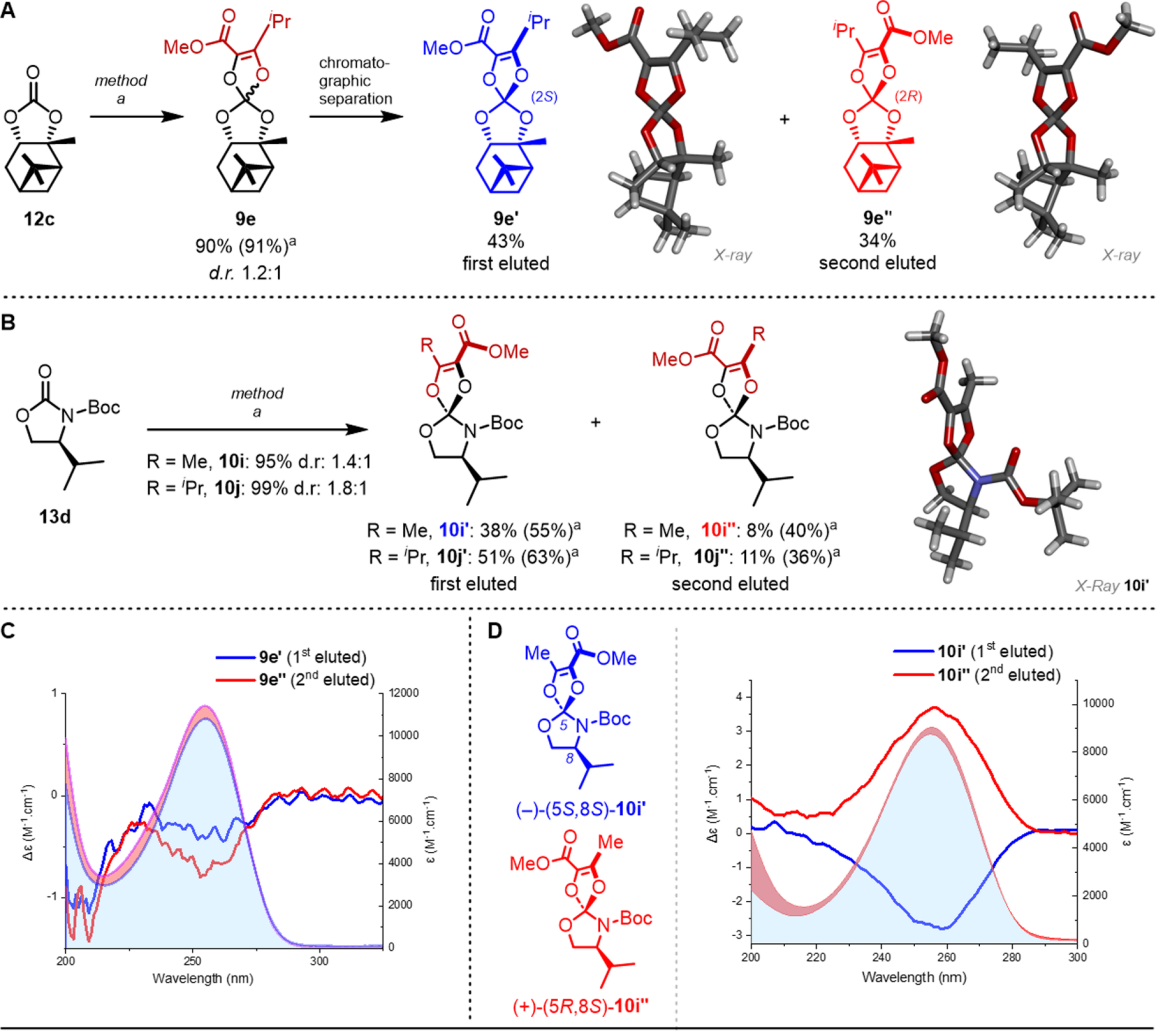
(A) Synthesis of **9e** as a 1.2:1 mixture of diastereomers. Chromatographic separation into (2*S*,3a′*R*,4′*R*,6′*R*,7a′*S*)-**9e′** (first eluted, CCDC 2406355) and (2*R*,3a′*R*,4′*R*,6′*R*,7a′*S*)-**9e′′** (second eluted, CCDC 2406356). (B) Synthesis of **10i** and **10j** as 1.4:1 and 1.8:1 mixtures of diastereomers. Preparative TLC separation into (5*S*,8*S*)-**10i′** (CCDC 2440356)/**10i′′** and **10j′**/**10j′′** (first/second eluted). (C) ECD spectra of **9e′** (blue) and **9e′′** (red) with absorption spectra (underlying filled curve) in the UV range. (D) ECD spectra of **10i′** (blue) and **10i′′** (red) with absorption spectra (underlying filled curve) in the UV range.

Analogously, enantiopure oxazolidinone **13d** ^34^ derived from (*S*)-valinol was employed as a substrate to generate aza-trioxa-substituted methanes **10i** (R′ = Me) and **10j** (R′ = ^*i*^Pr) as mixtures of diastereomers in excellent combined yields of 95 and 99% (method a, Figure 5B) and *d.r*.’s of 1.4:1 and 1.8:1, respectively. For **10i**, after preparative TLC (eluent pentane:EtOAc 90:10), major **10i′** and minor **10i′′** diastereomers were isolated as first and second eluted fractions in 38% and 8% isolated yields, respectively. The modest isolated yield of **10i′′** is due to the difficult separation of the two consecutive diastereomers on TLC. Similarly, major and minor diastereomers of **10j** were isolated as **10j′** (51%, first eluted) and **10j′′** (11%, second eluted). For diastereomerically pure **10i′**, single crystals were obtained by the slow diffusion of heptane in CH_2_Cl_2_ solution; absolute and relative configurations were determined to be (5*S*,8*S*) by X-ray diffraction analysis (CCDC 2440356). Isolated **10j** and **10j′** were also heated independently in DMSO at 100 °C for 1 h without, as expected, any evidence of epimerization or decomposition.

The crystal structures of **9e′**, **9e′′**, and **10i′** show the same geometry around the spiro center, with a tetrahedral coordination of the central atom (the tetrahedral distortion being around 8° in **9e′** and **9e′′** and 17° in **10i′**) and a close to 90° rotation between the 5-membered rings sharing the central atom. As visible in Figure 5, while **9e′** and **9e′′** adopt *s-cis* conformations for the ester moiety, a preferred *s-trans* geometry is observed for **10i′**, reflecting the facile exchange between the conformations in solution. In the case of **9e′** and **9e′′**, due to an allylic 1,3-strain,^35^ the tertiary C–H bond of the isopropyl group is periplanar to the dioxolene ring and to the ester group. In the solid state, the ester conformations may be influenced by other inter- or intramolecular interactions present (Supporting Information). The unsaturated dioxolene ring is flat, with a root-mean-square deviation from the plane of less than 0.04 Å for all compounds. The other 5-membered rings are more distorted and adopt a slightly twisted conformation, as shown by the puckering analysis (Supporting Information).

In terms of chiroptical properties, the two diastereomers **9e′** and **9e**″ have similar, though not identical, ECD spectra with negative bands around 255 (*g*_abs_ up to −6 × 10^–5^) and 205 nm (Figure 5C). This means that the pinane moiety dominates the ECD response over the spiro center. In particular, reasoning within the framework of chirality spheres,^36^ we infer that the strongest chiral perturbation is exerted by the methyl group at the C-3a′ position of the pinane moiety, closest to the chromophore (see Supporting Information). TD-DFT calculations reproduce the experimental ECD spectra reasonably well (Figure S18), validating the calculation approach employed above for the configurational assignment of **9a** and **10a**.

To complete the characterization, IR and VCD analyses of **9e′** and **9e′′** were also performed (Figures S8 and S9).^37^ As expected, the spectra are dominated by strong pinane peaks below about 1300 cm^–1^ and are thus quite similar for the two diastereomers. Marked differences still occur with a strong negative band around 1250 cm^–1^ for **9e′**, which is less pronounced for **9e′′**. More apparent is the strong negative band at 1200 cm^–1^ for **9e′′**. At this wavenumber, **9e′** has a (weaker) positive band next to a negative band at lower wavenumber.

Calculated VCD spectra, presented in Figures S8 and S9, are Boltzmann averages of the three most stable conformers with populations of 62, 37, and 1%, respectively. The corresponding conformers for the two diastereomers have almost identical relative stability. More importantly, the calculated spectra reproduce the spectral differences mentioned above for **9e′** and **9e′′**. Distinction of the two diastereomers and configuration assignment are hence possible by VCD; the assignment agrees with that of ECD and X-ray crystallography.

For ortho-carbamates **10**, a totally different situation is observed as for the (chir)­optical properties of the diastereomeric compounds **10i′**/**10i′′** (Figure 5D) and **10j′**/**10j′′** (Figure S7). Remarkably similar ECD spectra were obtained that presented, this time, essentially mirror-image situations within each diastereomeric series. Negative Cotton effects were obtained for **10i′** and **10j′**, while positive ECD bands were observed for epimers **10i′′** and **10j′′**, corresponding to opposite configurations at the spiro center. It was then safe to assign the following configurations: (5*S*,8*S*)-**10i′** (CCDC 2440356), (5*S*,8*S*)-**10j′**, (5*R*,8*S*)-**10i′′**, and (5*R*,8*S*)-**10j′′**. In view of the spectral similarity, this configuration assignment also fits well with that obtained by ECD for enantiomeric compounds (−)-(5*S*)-**10a** and (+)-(5*R*)-**10a** (Figure 3B). The influence of the stereogenic carbon imported from (*S*)-valinol is thus minimal as the ECD response is dominated by the configuration at the spiro center. Finally, *g*_abs_ values at 255 nm are −3.1 and −3.5 × 10^–4^ for **10i′** and **10j′**, respectively, whereas epimers **10i′′** and **10j′′** afford values of 4.2 and 5.1 × 10^–4^, respectively. ECD spectra of pairs **10i′**/**10i″** and **10j′**/**10j′′** were also reproduced by TD-DFT calculations (Supporting Information). The similar character of the major transition around 250 nm among all analyzed compounds highlights that the different ECD behavior observed between ortho-carbonates **9e** and ortho-carbamates **10** *i* **/10j** has a structural (larger vs smaller chiral perturbation exerted by chiral moieties) rather than electronic origin.

## Conclusions

Carbon stereocenters carrying oxygen and nitrogen atoms as only direct substituents were readily prepared under CpRu catalysis using cyclic carbonates and carbamates as substrates and α-diazo-β-ketoesters as reagents. Single enantiomers were isolated using CSP-HPLC, and cryptochiral character (*g*_abs_ ≈ 10^–5^) was revealed for tetraoxa compounds **9** by ECD analysis, which, together with TD-DFT calculations, provided robust absolute configuration assignments. Enantiopure aza-trioxa derivatives **10** demonstrated stronger chiroptical properties with dissymmetry factors ten times higher (*g*_abs_ ≈ 10^–4^) than for tetraoxa counterparts **9**. Crystalline spiro diastereomers were also obtained, further confirming the structural and configurational assignments. Using enantioselective dynamic chromatography, remarkable enantiomerization barriers were determined for ortho-carbonates **9** and ortho-carbamates **10** with barriers of up to 27.6 and 34.6 kcal/mol (half-lives of 227 days and >84,000 years at 25 °C, respectively). The large difference in configurational stability is rationalized *in silico*. While both series enantiomerize via an S_N_1-like mechanism, distinct C–O and C–N bond cleavages must be considered to take into account the experimental results and the variations in ΔH^⧧^ and ΔS^⧧^ values. Overall, our study highlights that spiro ortho-carbonates **9** and ortho-carbamates **10** are readily prepared and isolated in enantiomeric form and may constitute valuable targets for functional group manipulations, including in medicinal chemistry and asymmetric synthesis.

## Supplementary Material



## Data Availability

The data that support the findings of this study are openly available in yareta.unige.ch at 10.26037/yareta:26l4n6rn5bgovfx45rlfedw4ou. It will be preserved for 10 years.

## Notes

Experimental conditions, general methods, characterization of all new compounds, HPLC chromatograms, spectra (UV–vis, ECD, IR, VCD, NMR), experimental enantiomerization studies, computational results, X-ray crystallographic data, Cartesian coordinates, and references (PDF)

## Abbreviations

CSP: chiral stationary phase
DCM: dichloromethane
DMSO: dimethyl sulfoxide
ECD: electronic circular dichroism
MOs: molecular orbitals
TD-DFT: time-dependent density functional theory
