# Supplementary material for: All-Heteroatom-Substituted Carbon Spiro Stereocenters: Synthesis, Resolution, Enantiomeric Stability, and Absolute Configuration
Source: J Am Chem Soc. 2025 Jun 4;147(24):21121–30. doi: 10.1021/jacs.5c06394 (PMC12200235; doi:10.1021/jacs.5c06394)
Supplement: Supplementary file 1 [file ja5c06394_si_001.pdf]

Supporting Information

all-Heteroatom-Substituted Carbon Spiro Stereocenters: Synthesis, Resolution, Enantiomeric Stability, and Absolute Configuration

Olivier Viudes,[a] Céline Besnard,[b] Alexander F. Siegle,[c] Oliver Trapp,[c] Thomas Bürgi,[d] Gennaro Pescitelli,*[e] Jérôme Lacour*[a]

[a] Department of Organic Chemistry, University of Geneva
Quai Ernest Ansermet 30, 1211 Geneva 4, Switzerland.

[b] Laboratory of crystallography, University of Geneva
Quai Ernest Ansermet 24, 1211 Geneva 4, Switzerland.

[c] Department of Chemistry, Ludwig-Maximilians-University Munich,
Butenandtstr. 5-13, Munich 81377, Germany.

[d] Department of Physical Chemistry, University of Geneva
Quai Ernest Ansermet 30, 1211 Geneva 4, Switzerland.

[e] Dipartimento di Chimica e Chimica Industriale, University of Pisa
Via G. Moruzzi 13, Pisa, Italy.

Table of Contents

1. General information S3
2. General methods S7
    2.1. N-carbamate oxazolidinone substrates S7
    2.2. Ortho-carbonates 9 and ortho-carbamates 10 .... S9
    2.3. Reaction on 1 mmol scale S17
3. Mechanistic rationale of the reaction S17
4. HPLC chromatograms S18
5. UV, ECD, IR and VCD Spectra S21
6. Enantiomerization studies by enantioselective dynamic chromatography S25
7. Computational results S28
8. X-Ray Crystallographic Data S45
9. ^{1}H and ^{13}C NMR spectra S56
10. Cartesian coordinates S101
11. References and Notes S120

1. General information

Reagents: All reagents and solvents were purified using standard methods. Anhydrous dichloromethane (CH_2Cl_2) was dried by passing through activated alumina under a positive pressure of nitrogen. Anhydrous reactions were carried out in flame-dried glassware under an argon atmosphere. All other chemicals were used as received.

Carbonate reactants **12a** and **12c** were prepared following the procedure of Baral, Lee and Kim.[1] Compound **12b** was synthesized according to Burk and Roof.[2] All spectral data of **12a** to **12c** fitted previously reported descriptions.[1-2]

Carbamate reactants **13a** to **13d** were prepared following the reported procedure from the literature on lactam functionalization.[3]

Diazo derivatives **11a** and **11b** were synthesized according to the literature procedures.[4] Catalyst $[CpRu(CH_3CN)_3][BAr_F]$ **14** was prepared according to the previously reported procedure.[5]

Important note: Diazo compounds are high energy materials and should be handled with caution.[6] Although α-diazomalonates are stable at room temperature, it is advisable to carry out reactions behind a blast shield.

Reaction setup:

NMR: ^{1}H and ^{13}C NMR spectra were recorded on 300, 400, or 500 MHz instruments at 25 °C. The chemical shifts are given in ppm on the δ scale. The solvent peak was used as reference value: for ^{1}H NMR: $CDCl_3$ δ = 7.26 ppm; for ^{13}C NMR: $CDCl_3$ = 77.2 ppm; Data are presented as follows; chemical shift, multiplicity (s = singlet, d = doublet, t = triplet, q = quadruplet, p = pentet (quintet) and m = multiplet), coupling constants (J in Hz), integration, and carbons with same chemical shift (x carbons). For NMR yield, 1,3,5-trimethoxybenzene was used as external standard.

Standard IR: Spectra were recorded neat on a Perkin-Elmer Spectrum 100 FT-IR spectrometer using a diamond ATR Golden Gate sampling. Wavelengths of maximum absorbance are quoted in nm.

UV-visible absorption: Measurements were performed in 1 cm optical quartz cells and spectra were recorded on a JASCO V-650 spectrophotometer at 20 °C with parameters as follows: scan speed – 100 nm/min, bandwidth – 1 nm, and data interval – 1 nm. All solvents were of spectroscopic or HPLC grade and were used as received.

Electronic circular dichroism (ECD): Spectra were recorded on JASCO J-815 spectropolarimeter at 20 °C with parameters as follows: scan speed – 100 nm/min, bandwidth – 1 nm, data interval – 1 nm, and integration time – 0.1 sec. Measurement were performed in analytical grade solvents in 1 cm optical path quartz cells. All spectra are the average of 10 accumulations each.

Optical rotations: Measured in CH_2Cl_2, on a Perkin Elmer 343 Polarimeter using a sodium lamp (λ 589 nm, D-line) or mercury lamp (λ 365 nm). α values are reported at a given temperature (20 °C) in degrees.$cm^2.g^{-1}$ with concentration in g/L.

High-resolution mass spectra (HRMS): Data were recorded by the Department of Mass Spectroscopy of the University of Geneva on a Xevo G2 TOF spectrometer equipped with orthogonal electrospray interface (ESI). The parent ions $[M + Na]^+$, $[M + H]^+$ are quoted.

Chromatography: Analytical TLC was carried out on silica gel 60 F254 plates with visualization by ultraviolet light. Flash chromatography was carried out using silica gel 60 (40–63 µm).

Chiral stationary phase HPLC analyses and resolutions were performed on Agilent LC 1100 instruments using CHIRALPAK® IG columns, analytical (4.6 x 250 mm) or semi-preparative (100 x 250 mm).

For both silica gel chromatography and CSP-HPLC, **blue and red colors** indicate first and second eluted fractions of stereoisomers, respectively.

Enantioselective dynamic chromatography: The stereodynamics of **9b** and **10b** were investigated by dynamic GC performed on a Trace GC Ultra single quadrupole ISQ mass spectrometer equipped with a split injector (250 °C) and a flame ionization detector (250 °C). Helium was used as inert carrier gas. Separation of **9b** was performed on a fused silica capillary (25 m, 0.25 mm *i.d.*, film thickness 0.25 µm) coated with heptakis(2,3-di-O-acetyl-6-O-TBDMS) β-cyclodextrin in PS 086 (50% w/w) and separation of **10b** was performed on a fused silica capillary (8.5 m, 0.25 mm *i.d.*, film thickness 0.50 µm) coated with heptakis(2,3-di-O-methyl-6-O-TBDMS) β-cyclodextrin in PS 086 (50% w/w).

The stereodynamics of **9a** was investigated by dynamic HPLC performed on an Agilent Technologies 1200 HPLC using a CHIRALPAK® IG-3 column (4.6 x 150 mm, particle size: 3 µm). All dynamic measurements were repeated at least three times at each temperature.

Reaction rate constants were determined using the unified equation, which allows for the direct calculation of the reaction rate constants k_1 and k_{-1} and Gibbs activation energies $\Delta G^{\ddagger}$ for all types of first-order reactions taking place in chromatographic or electrophoretic systems.[7] The analytical solutions of the unified equation are implemented in the computer program DCXplorer which allows real-time evaluation of elution profiles of dynamic chromatographic experiments. The program DCXplorer can be obtained from O. Trapp upon request or by direct download (https://www.cup.lmu.de/oc/trapp/tools.html).

The Gibbs free activation energy $\Delta G^{\ddagger}(T)$ was calculated according to the Eyring equation (eq. 1) with k_B as the Boltzmann constant ($k_B = 1.381 \times 10^{-23}$ J K^{-1}), T as the enantiomerization temperature [K], h as Planck's constant ($h = 6.626 \times 10^{-34}$ J sec), and R as the gas constant ($R = 8.31$ J K^{-1} mol^{-1}). The statistical factor κ was set to 0.5 for a degenerated interconversion process.

$$(1) \qquad \Delta G^{\ddagger}(T) = -RTln\left(\frac{k_1 h}{\kappa k_B T}\right)$$

The activation enthalpy $\Delta H^{\ddagger}$ was obtained from the slope and the activation entropy $\Delta S^{\ddagger}$ from the intercept of the Eyring plot ($\ln(k_1/T)$ as a function of T^{-1}). Deviations of the activation parameters $\Delta H^{\ddagger}$ and $\Delta S^{\ddagger}$ have been calculated by analysis of the confidence interval of the linear regression with a level of confidence of 95%.

Melting points were measured in open capillary tubes and were uncorrected in open capillary tubes and were uncorrected.

IR and vibrational circular dichroism (VCD) spectra were recorded on a Bruker PMA 50 accessory coupled to a Tensor 27 Fourier transform infrared spectrometer. A photoelastic modulator (Hinds PEM 90) set at l/4 retardation was used to modulate the handedness of the circular polarized light. Demodulation was performed by a lock-in amplifier (SR830 DSP). An optical low-pass filter (< 1800 cm^{-1}) in front of the photoelastic modulator was used to enhance the signal/noise ratio. Spectra were recorded with a transmission cell equipped with CaF_2 windows and a 0.2 mm spacer. Solutions were prepared in deuterated acetonitrile concentrations of 11 mg in 300 μL. The pure solvent was used as the reference for VCD measurements. Both sample and reference were measured at a resolution of 4 cm^{-1} by averaging about 30’000 scans in total for sample and reference, respectively. The reference VCD spectrum was subtracted from the sample spectrum. Spectra are presented without further data processing.

X-ray crystallography: All data were collected on an XtaLAB Synergy-S diffractometer equipped with an Hypix arc 150 detector, using Cu Kα radiation. Data reduction was carried out in the crysalis Pro Software. Structure solution was made using dual space methods in the shelxt program Refinements were carried out in ShexlL within the Olex2 software. Details for the refinement for the structure can be found in the table below, with a representation of the asymmetric unit with displacement ellipsoids drawn at 50 percent probability.

Computational methodology: The starting input structures of (*S*)-**9a**, (2*S*)-**9e’**, (2*R*)-**9e”**, (*R*)-**10a**, (5*R*)-**10i’**, (5*S*)-**10i”**, (5*R*)-**10j’** and (5*S*)-**10j”**, were built in Spartan’20 (Wavefunction, Inc., Irvine, CA) and a conformational search was run with the Monte Carlo algorithm and Molecular Merck Force Field (MMFF), using default parameters. All ring atoms were allowed to “flip” during the conformational search, and all extra-annular single bonds were allowed to rotate. All structures thus found were preoptimized at B3LYP-D3/6-31G(d) level in vacuo, in Spartan’20. Subsequent calculations were run with Gaussian16, rev. C.01,[8] with default grids and convergence criteria. All structures found above were re-optimized at B3LYP-D3BJ/6-311+G(d,p) level, including IEF-PCM solvent model for acetonitrile. Frequency calculations were run at the same level to verify the nature of true energy minimum of each conformer (no imaginary frequency) and to calculate relative Gibbs energies, to be used to estimate Boltzmann population at 300K. Four low-energy minima were identified for each compound with population > 0.5% at 300K (see section 8 herein). To simulate absorption and ECD spectra, TD-DFT calculations were run using two functionals (B3LYP and CAM-B3LYP), aug-cc-pVTZ basis set, and IEF-PCM solvent model for acetonitrile. The two functionals led to consistent results, with CAM-B3LYP reproducing better the experimental spectra. Weighted-average spectra were obtained using Boltzmann populations at 300K, estimated from Gibbs energies, and plotted using SpecDis v. 1.71.[9] Plotting parameters were: exponential band-

width σ= 0.3-0.35 eV; wavelength correction, +4 nm; vertical scaling, 1-1.5. Molecular graphics were prepared with UCSF ChimeraX, v 1.6.1.[10],[11] VCD spectra were calculated at the B3PW91 level using a 6-31+G(d,p) basis set including IEF-PCM solvent model for acetonitrile. Boltzmann-averaged spectra were obtained at 300K. Calculated vibrational frequencies were scaled by a factor of 0.985.

Energy scans and transition state optimizations were run at B3LYPD3BJ/6-31+G(d) level including SMD solvent model for acetonitrile. For both compounds **9a** and **10a**, the lowest-energy conformer (in terms of internal energy) obtained from the previous analysis was considered and re-optimized. Then, 4 energy scans were run by increasing C–O and C–N distances by 0.2Å steps. The first minimum encountered along the lowest-energy path was optimized to yield the zwitterionic intermediate structures **I'** and **IV"**, which were verified to be true minima (zero imaginary frequencies). For finding the lowest-energy conformer of **IV"** a systematic conformational analysis could not be employed because it converged toward **10a**; therefore, the conformational space was sampled by scanning selected torsions (N–CH_2 and C^+O–CH_2). The transition state **TS'** was first grossly identified by means of torsional energy scans around C^+–O bonds by 30° steps. The lowest-energy maximum (among the two observed ones) and the two closest points on both sides were used as starting points for transition state optimization using the STQN algorithm (QST3 option in Gaussian), to yield the zwitterionic transition state structure **TS'**, which was verified to be saddle points (one imaginary frequency).

Non-covalent interaction (NCI) [12] plots were generated with MultiWfn v. 3.8.[13]

Dataset

The dataset for this article can be found at the following DOI: https://doi.org/10.26037/yareta:26l4n6rn5bgovfx45rlfedw4ou. It will be preserved for 10 years. It contains the original files for all NMR, IR, HRMS and X-ray characterization.

2. General methods

2.1. N-carbamate oxazolidinone substrates

Carbamate reactants **13a** to **13d** were prepared following the reported procedure from the literature on lactam functionalization.[3]

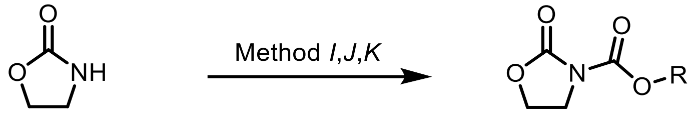

***Method I*:** In a round-bottom flask containing a solution of cyclic carbamate or urea (1 equiv.) in MeCN (c = 0.5 M), 4-dimethylaminopyridine (0.1 equiv.) was added, followed by Boc_2O (1.2 equiv.). The reaction mixture was stirred at room temperature overnight. After cooling to room temperature, the reaction was quenched with 10% HCl_{aq}. The aqueous layer was then extracted with CH_2Cl_2 (3×). The combined organic layers were washed with H_2O (2×) and brine (1×), dried over Na_2SO_4, filtered, and the solvent was removed under reduced pressure. The crude residue was purified by flash chromatography on silica gel (EtOAc/pentane) to afford the desired N-protected carbamates **13**.

Method J: To a solution of cyclic carbamate (1 equiv) in CH_2Cl_2 (c = 0.5 M) at room temperature, Et_3N (1.5 equiv.), 4-dimethylaminopyridine (0.1 equiv.) and 2,2,2-trichloroethyl chloroformate (1.5 equiv.) were sequentially added. The reaction mixture was stirred overnight at room temperature. After completion, the mixture was quenched with water (75 mL), and the aqueous layer was extracted with EtOAc (3×25 mL). The combined organic layers were washed with H_2O (2×20 mL) and brine (1×10 mL), dried over Na_2SO_4, filtered, and the solvent was removed under reduced pressure. The crude residue was purified by flash chromatography to afford the desired N-protected carbamate **13**.

Method K: To a solution of the appropriate cyclic carbamate (1 equiv.) in THF (c = 0.2 M) at -78°C, n-BuLi (1.2 equiv, c = 0.16 M in hexane) was added. The reaction was stirred at this temperature for 30 minutes. Then, either the chloroformate, or sulfonyl chloride (1.5 equiv.) was added, and the mixture was allowed to stir at room temperature overnight. The reaction mixture was quenched with 10% HCl_{aq}. The aqueous layer was extracted with EtOAc (3×25 mL), and the combined organic layers were washed with H_2O (2×) and brine (1×). The organic phase was dried over Na_2SO_4, filtered, and the solvent was removed under reduced pressure. The crude residue was purified by flash chromatography on silica gel (EtOAc/pentane), yielding the desired N-protected carbamate **13**

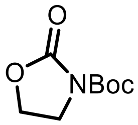

13a

tert*-Butyl 2-oxooxazolidine-3-carboxylate 13a:** Prepared according to *method **I using oxazolidin-2-one (435 mg, 5 mmol) and Boc_2O (1.31 g, 6 mmol) as precursors. Product **13a** (905 mg, 97% yield) was obtained as a white solid after chromatography (SiO_2, eluent: pentane/EtOAc, 70:30 to 50:50).

R*f* 0.47 pentane/EtOAc (50:50) **m.p:** 84−86 °C. **^{1}H NMR** (500 MHz, $CDCl_3$) δ 4.35 – 4.31 (m, 2H), 3.98 – 3.94 (m, 2H), 1.53 (s, 9H). **^{13}C NMR** (126 MHz, $CDCl_3$) δ 152.2 (C=O), 149.7 (C=O), 84.0 (C), 61.4 (CH_2) 43.6 (CH_2), 28.1 ($3xCH_3$). **IR** (CH_2Cl_2): 2998, 1806, 1712, 1475, 1389, 1370, 1306, 1257, 1207, 1158, 1073, 1033, 980, 849, 725 ṽ/cm^{-1} **HRMS** (ESI) m/z calc for $C_8H_{13}NO_4Na$: 210.0737; found: 210.0743 $[M + Na]^+$.

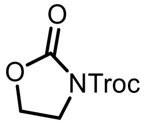

13b

2,2,2-Trichloroethyl 2-oxooxazolidine-3-carboxylate 13b: Prepared according to *method **J*** using oxazolidin-2-one (435 mg, 5 mmol) and Troc-Cl (1.59 g, 7.5 mmol) as precursors. Product **13b** (840 mg, 65% yield) was obtained as a white solid after chromatography (SiO_2, eluent: pentane/EtOAc, 70:30 to 50:50).

R*f* 0.42 pentane/EtOAc (50:50) **m.p:** 99−101 °C.**^{1}H NMR (500 MHz, $CDCl_3$)** δ 4.89 (s, 3H), 4.45 (ddd, *J* = 8.2, 7.4, 1.0 Hz, 2H), 4.13 (ddd, *J* = 8.7, 7.3, 0.9 Hz, 2H). **^{13}C NMR** (126 MHz, $CDCl_3$) δ 151.6 (C=O), 149.3 (C=O), 75.6 (CH_2), 61.9 (CH_2), 43.5 (CH_2). **IR** (CH_2Cl_2): 1824, 1735, 1391, 1306, 1196, 1102, 1040, 798, 763, 723 ṽ/cm^{-1} **HRMS** (ESI) m/z calc for $C_6H_6Cl_3NO_4Na$: 283.9260; found: 283.9262 $[M + Na]^+$.

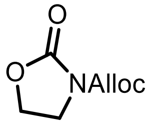

13c

Allyl 2-oxooxazolidine-3-carboxylate 13c: Prepared according to *method **K*** using oxazolidin-2-one (435 mg, 5 mmol) and Alloc-Cl (905 mg, 7.5 mmol) as precursors. Product **13c** (402 mg, 47% yield) was obtained as a white solid after chromatography (SiO_2, eluent: pentane/EtOAc, 70:30 to 50:50).

R*f* 0.41 pentane/EtOAc (50:50) **m.p:** 40−42 °C. **^{1}H NMR** (500 MHz, $CDCl_3$) δ 5.96 (ddtd, *J* = 17.0, 10.3, 5.7, 0.9 Hz, 1H), 5.42 (dt, *J* = 17.1, 1.4 Hz, 1H), 5.29 (dt, *J* = 10.5, 1.2 Hz, 1H), 4.76 (dd, *J* = 5.7, 1.3 Hz, 2H), 4.35 – 4.30 (m, 2H), 3.98 – 3.94 (m, 2H). **^{13}C NMR** (126 MHz, $CDCl_3$) δ 152.0 (C=O), 151.0 (C=O), 131.2 (CH alkene), 119.6 (CH alkene), 67.8 (CH allylic), 61.8 (CH_2), 43.5 (CH_2). **IR** (CH_2Cl_2): 2926, 1813, 1788, 1724, 1648, 1480, 1458, 1388, 1307, 1199, 1073, 1037, 985, 940, 772, 759, 715 ṽ/cm^{-1} **HRMS** (ESI) m/z calc for $C_7H_9NO_4Na$: 194.0429; found: 194.0437 $[M + Na]^+$.

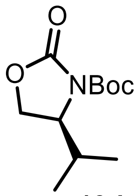

13d

tert*-Butyl *(S)*-4-isopropyl-2-oxooxazolidine-3-carboxylate 13d:** Prepared according to *method **I using (S)-4-isopropyloxazolidin-2-one (646 mg, 5 mmol) and Boc_2O (1.31 g, 6 mmol) as precursors. Product **13d** (1.14 g, 90% yield) was obtained as a white solid after chromatography (SiO_2, eluent: pentane/EtOAc, 70:30 to 50:50).

R*f* 0.50 pentane/EtOAc (50:50) **m.p:** 100−102 °C. **^{1}H NMR** (500 MHz, $CDCl_3$) δ 4.35 – 4.31 (m, 2H), 4.11 (dd, *J* = 8.4, 2.5 Hz, 1H), 2.30 (heptd, *J* = 7.0, 4.0 Hz, 1H), 1.53 (s, 8H), 0.91 (d, *J* = 7.0, 6H) 0.90 (d, *J* = 7.0, 3H). **^{13}C NMR** (126 MHz, $CDCl_3$) δ 152.7 (C=O), 149.7 (C=O), 83.9 (C), 63.0 (CH_2), 59.4 (CH), 29.5 (CH), 28.1 ($3xCH_3$), 18.1 (CH_3), 15.0 (CH_3). **IR** (CH_2Cl_2): 2965, 1799, 1709, 1395, 1372, 1308, 1289, 1260, 1203, 1156, 1120, 1073, 1049, 1037, 1016, 982, 809, 781, 754, 719 ṽ/cm^{-1} **HRMS** (ESI) m/z calc for $C_{11}H_{19}NO_4Na$: 252.1212; found: 252.1214 $[M + Na]^+$

2.2. Ortho-carbonates 9 and ortho-carbamates 10

Method a: In a screw-cap vial containing a solution of the cyclic carbonate **12** or carbamate **13** (0.2 mmol, 1.0 equiv) in CH_2Cl_2 (0.2 mL) and [CpRu(CH_3CN)$_3$][BAr_F] **14** (0.005 mmol, 0.025 equiv) as catalyst, methyl **11a** or isopropyl **11b** α-diazo-β-ketoester (0.4 mmol, 2 equiv) were added in one portion as liquids (syringe addition). Reaction mixtures were stirred for 15 h at 60 °C. After cooling to room temperature, the solvent was removed under reduced pressure. Purification by column chromatography over silica gel afforded the titled compounds.

Method b: In a screw-cap vial containing a solution of the cyclic carbonate **12** or carbamate **13** (0.2 mmol, 1.0 equiv) in CH_2Cl_2 (0.2 mL) and a 1:1 combination of [CpRu(CH_3CN)$_3$][BAr_F] (0.003 mmol, 0.015 equiv) and 4,4' dinitro-2,2'-bipyridine (0.003 mmol, 0.015 equiv) as catalyst, isopropyl α-diazo-β-ketoester **11b** (0.4 mmol, 2 equiv) was added in one portion as a liquid (syringe addition). Reaction mixtures were stirred for 3 h at 60 °C. After cooling to room temperature, the solvent was removed under reduced pressure. Purification by column chromatography over silica gel (eluent pentane/EtOAc) afforded the titled compound.

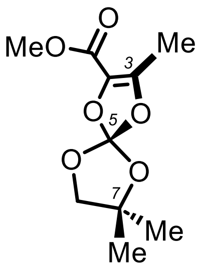

rac-**9a**

Methyl 3,7,7-trimethyl-1,4,6,9-tetraoxaspiro[4.4]non-2-ene-2-carboxylate 9a: Prepared according to *method a* using cyclic carbonate **12a** (23 mg, 0.2 mmol) and methyl α-diazo-β-ketoester **11a** (57 mg, 0.4 mmol) as precursors. Product **9a** (33 mg, 71% yield) was obtained as a colorless oil after chromatography (SiO_2, eluent pentane/EtOAc, 95:5 to 90:10).

R_f 0.36 pentane/EtOAc (9:1)
^{1}H NMR (500 MHz, $CDCl_3$) δ 3.99 (d, ^2J = 7.7 Hz, 1H, diastereotopic H on C^8), 3.96 (d, ^2J = 7.7 Hz, 1H, diastereotopic H on C^8), 3.82 (s, 3H, OCH_3), 2.26 (s, 3H, allylic CH_3), 1.48 (s, 3H, diastereotopic CH_3), 1.47 (s, 3H, diastereotopic CH_3).
^{13}C NMR (126 MHz, $CDCl_3$) δ 160.2 (C=O), 146.7 (C^3), 135.5 (C^5), 126.4 (C^2), 82.5 (C^7), 76.3 (C^8), 51.8 (OCH_3), 25.7 (diastereotopic Me), 25.5 (diastereotopic Me), 11.3 (allylic CH_3).
IR (CH_2Cl_2) 2981, 1715, 1683, 1442, 1378, 1353, 1276, 1234, 1154, 1115, 1086, 1044, 1001, 984, 915, 865, 810, 779, 760, 700, 621 $\tilde{v}$/cm^{-1}.
HRMS (ESI) m/z calc for $C_{10}H_{14}O_6Na$: 253.0683; found: 253.0671 $[M + Na]^+$
$[\boldsymbol{\alpha}]_{\mathbf{20}}^{\boldsymbol{D}}$ −16 and +18 in CH_2Cl_2 (0.2 g/L) after CSP-HPLC resolution (see below); (−)- and (+)-**9a** are the first and second eluted enantiomers on CHIRALPAK® IG (Hexane+0.1% Et_2NH/CH_2Cl_2)

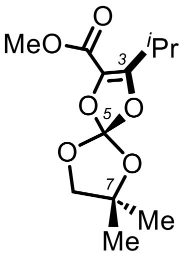

rac-**9b**

Methyl 3-isopropyl-7,7-dimethyl-1,4,6,9-tetraoxaspiro[4.4]non-2-ene-2-carboxylate 9b:

Prepared according to *method a* using cyclic carbonate **12a** (23 mg, 0.2 mmol) and isopropyl α-diazo-β-ketoester **11b** (68 mg, 0.4 mmol) as precursors. Product **9b** (47 mg, 91% yield) was obtained as a colorless oil after chromatography (SiO_2, eluent: pentane/EtOAc, 95:5).

$\mathbf{R_f}$ 0.41 pentane/EtOAc (9:1)

^{1}H NMR (500 MHz, $CDCl_3$) δ 3.97 (s, 2H), 3.81 (s, 3H, OCH_3), 3.41 (hept, ^3J = 7.1 Hz, 1H, allylic CH), 1.47 (s, 6H), 1.193 (d, ^3J = 7.1, 3H, diastereotopic Me isopropyl), 1.188 (d, ^3J = 7.1 Hz, 3H, diastereotopic Me isopropyl)

^{13}C NMR (126 MHz, $CDCl_3$) δ 160.2 (C=O), 154.8 (C^3), 135.6 (C^5), 124.4 (C^2), 82.3 (C^7), 76.2 (C^8), 51.8 (OCH_3), 25.7 (diastereotopic Me), 25.5 (diastereotopic Me), 25.0 (allylic CH), 19.74 (diastereotopic Me isopropyl), 19.70 (diastereotopic Me isopropyl).

IR (CH_2Cl_2) 2976, 1714, 1673, 1467, 1440, 1357, 1320, 1277, 1231, 1170, 1135, 1088, 1038, 984, 930, 861, 807, 780, 762 $\tilde{v}/cm^{-1}$

HRMS (ESI) m/z calc for $C_{12}H_{18}O_6Na$: 281.0996; found: 281.0988 $[M + Na]^+$

Analytical and semi-preparative CSP-HPLC resolution were not attempted on this derivative.

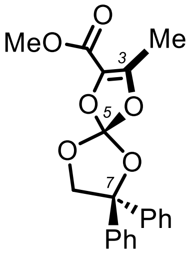

rac-**9c**

Methyl 3-methyl-7,7-diphenyl-1,4,6,9-tetraoxaspiro[4.4]non-2-ene-2-carboxylate 9c:

Prepared according to *method a* using cyclic carbonate **12b** (48 mg, 0.2 mmol) and α-diazo-β-ketoester **11a** (57 mg, 0.4 mmol) as precursors. Product **9c** (30 mg, 42% yield) was obtained as a colorless oil after chromatography (SiO_2, eluent: pentane/THF, 95:5).

$\mathbf{R_f}$ 0.34 pentane/EtOAc (9:1)

^{1}H NMR (500 MHz, $CDCl_3$) δ 7.49 – 7.27 (m, 10H), 4.81 (d, ^2J = 8.3 Hz, 1H, diastereotopic H on C^8), 4.73 (d, ^2J = 8.3 Hz, 1H, diastereotopic H on C^8), 3.82 (s, 3H, OCH_3), 2.27 (s, 3H, allylic CH_3)

^{13}C NMR (126 MHz, $CDCl_3$) δ 160.2 (C=O), 146.9 (C^3), 141.5 (C_{Ar}), 141.3 (C_{Ar}) 135.8 (C^5), 128.7 (2xCH_{Ar}), 128.33 (2xCH_{Ar}), 128.30 (2xCH_{Ar}), 126.7 (C^2), 126.4 (2xCH_{Ar}), 126.3 (2xCH_{Ar}), 82.5 (C^7), 76.3 (C^8), 51.8 (OCH_3), 11.3 (allylic CH_3).

IR (CH_2Cl_2) 1715, 1686, 1493, 1442, 1382, 1353, 1246, 1164, 1117, 1084, 1050, 1036, 988, 920, 866, 782, 758, 699, 670 $\tilde{v}/cm^{-1}$

HRMS (ESI) m/z calc for $C_{20}H_{18}O_6Na$: 377.0996; found: 377.0992 $[M + Na]^+$

Analytical and semi-preparative CSP-HPLC resolution were not attempted on this derivative.

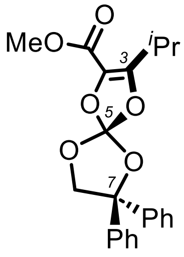

rac-**9d**

Methyl 3-isopropyl-7,7-diphenyl-1,4,6,9-tetraoxaspiro[4.4]non-2-ene-2-carboxylate 9d:

Prepared according to *method* ***b*** using cyclic carbonate **12b** (48 mg, 0.2 mmol) and α-diazo-β-ketoester **11b** (68 mg, 0.4 mmol) as precursors. Product **9d** (70 mg, 92% yield) was obtained as a white solid after chromatography (SiO_2, eluent: pentane/EtOAc, 95:5).

R*f* 0.39 pentane/EtOAc (9:1) **m.p** = 79-81 °C

^{1}H NMR (500 MHz, $CDCl_3$) δ 7.63 – 7.27 (m, 10H), 4.81 (d, ^{2}J = 8.3 Hz, 1H, diastereotopic H on C^8), 4.73 (d, ^{2}J = 8.3 Hz, 1H, diastereotopic H on C^8), 3.81 (s, 3H, OCH_3), 3.42 (hept, ^{3}J = 6.9 Hz, 1H, allylic CH), 1.20 (d, ^{3}J = 7.0 Hz, 3H Me isopropyl), 1.15 (d, ^{3}J = 6.9 Hz, 3H, Me isopropyl).

^{13}C NMR (126 MHz, $CDCl_3$) δ 160.2 (C=O), 154.6 (C^3), 141.6 (C_{Ar}), 141.3 (C_{Ar}), 135.9 (C^5), 128.7 ($2xCH_{Ar}$), 128.5 ($2xCH_{Ar}$), 128.3 (CH_{Ar}), 128.2 (CH_{Ar}), 126.4 ($2xCH_{Ar}$), 126.3 ($2xCH_{Ar}$), 124.8 (C^2), 88.5 (C^7), 76.4 (C^8), 51.8 (OCH_3), 25.1 (allylic CH), 19.8 (diastereotopic Me isopropyl), 19.7 (diastereotopic Me isopropyl).

Some of the aromatic diastereotopic ^{13}C signals are isochronous. 6 peaks CH-Ar

IR (CH_2Cl_2): 2973, 1715, 1676, 1444, 1359, 1319, 1249, 1189, 1140, 1099, 1041, 994, 785, 700 $\tilde{v}/cm^{-1}$

HRMS (ESI): m/z calc for $C_{22}H_{23}O_6$: 383.1490; found: 383.1508 $[M + H]^+$

Analytical and semi-preparative CSP-HPLC resolution were not attempted on this derivative.

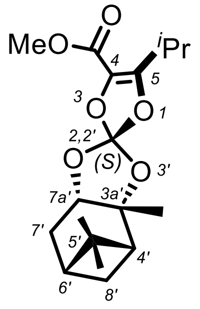

(2*S*,3a'*R*,4'*R*,6'*R*,7a'*S*)-**9e'**

Methyl (2*S*,3a'*R*,4'*R*,6'*R*,7a'*S*)-5-isopropyl-3a',5',5'-trimethyl-3a',4',5',6',7',7a'-hexahydrospiro[[1,3]dioxole-2,2'-[4,6]methanobenzo[d][1,3]dioxole]-4-carboxylate 9e':

Prepared together with **9e"** (see below) according to *method* ***a*** using cyclic carbonate **12c** (48 mg, 0.2 mmol) and α-diazo-β-ketoester **11b** (68 mg, 0.4 mmol) as precursors. Less polar product **9e'** (29 mg, 43% yield) was obtained as a white solid after a first chromatography (SiO_2, eluent: pentane/EtOAc, 97:3) affording a mixed fraction with **9e"** and then a second separation by prep-TLC (pentane/EtOAc, 9:1).

R*f* 0.51 Pentane/EtOAc (9:1)

m.p 72-74 °C

^{1}H NMR (500 MHz, $CDCl_3$) δ 4.46 (dd, *J* = 8.2, 1.9 Hz, 1H on $C^{7a'}$), 3.79 (s, 3H, OCH_3), 3.42 (hept, *J* = 6.9 Hz, 1H, allylic CH), 2.34 – 2.22 (m, 2H, 1H on $C^{8'}$ and 1H on $C^{7'}$), 2.11 (t, *J* = 5.4 Hz, 1H on $C^{4'}$), 2.06 (ddd, *J* = 14.9, 3.6, 2.0 Hz, 1H on $C^{8'}$), 1.96 (dd, *J* = 5.3, 2.7 Hz, 1H on $C^{6'}$), 1.70 (d, *J* = 11.1 Hz, 1H on $C^{7'}$), 1.54 (s, 3H, CH_3 on $C^{3a'}$), 1.32 (s, 3H, CH_3 on $C^{5'}$), 1.19 (d, *J* = 6.9 Hz, 3H, diastereotopic Me isopropyl), 1.18 (d, *J* = 6.9 Hz, 3H, diastereotopic Me isopropyl), 0.85 (s, 3H, CH_3 on $C^{5'}$).

^{13}C NMR (126 MHz, $CDCl_3$) δ 160.3 (C=O), 154.9 (C^5), 135.2 (C^2), 124.2, (C^4) 87.4 ($C^{3a'}$), 77.9 ($C^{7a'}$), 51.6 (OCH_3) 50.8 ($C^{4'}$), 39.6 ($C^{6'}$), 38.2 ($C^{5'}$), 33.6 ($C^{7'}$), 27.8 (CH_3 on $C^{3a'}$), 27.2 (CH_3 on $C^{5'}$), 26.2 ($C^{8'}$), 25.0 (CH on isopropyl), 24.1 (CH_3 on $C^{5'}$), 19.8 (diastereotopic Me isopropyl), 19.7 (diastereotopic Me isopropyl).

IR (CH_2Cl_2): 2923, 1714, 1673, 1363, 1170, 1124, 1095, 1057, 1036, 779 ṽ/cm^{-1}
HRMS (ESI): m/z calc for $C_{18}H_{26}O_6Na$: 361.1622; found: 361.1636 $[M + Na]^+$
$[\alpha]_{20}^{D}$ −3 in CH_2Cl_2 (0.34 g/L)
$[\alpha]_{20}^{365}$ −17 in CH_2Cl_2 (0.34 g/L)

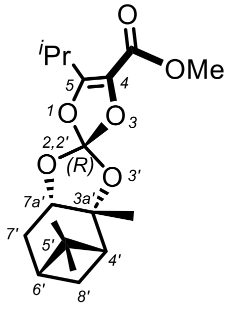

(2*R*,3a'*R*,4'*R*,6'*R*,7a'*S*)-**9e"**

Methyl (2*R*,3a'*R*,4'*R*,6'*R*,7a'*S*)-5-isopropyl-3a',5',5'-trimethyl-3a',4',5',6',7',7a'-hexahydrospiro[[1,3]dioxole-2,2'-[4,6]methanobenzo[d][1,3]dioxole]-4-carboxylate 9e":
Prepared together with **9e'** (see above) according to *method a* using cyclic carbonate **12c** (48 mg, 0.2 mmol) and α-diazo-β-ketoester **11b** (68 mg, 0.4 mmol) as precursors. More polar adduct **9e"** (23 mg, 34% yield) was obtained as a white solid after a first chromatography (SiO_2, eluent: pentane/EtOAc, 97:3) affording a mixed fraction with **9e'** and then a second separation by prep-TLC (pentane/EtOAc, 9:1).

$\mathbf{R_f}$ 0.46 Pentane/EtOAc (9:1)
m.p 75-77 °C
^{1}H NMR (500 MHz, $CDCl_3$) δ 4.49 (dd, *J* = 8.4, 2.1 Hz, 1H on C$^{7a'}$), 3.80 (s, 3H, OCH_3), 3.41 (hept, *J* = 6.9 Hz, 1H, allylic CH) 2.28 (dddd, *J* = 19.3, 12.6, 5.5, 2.3 Hz, 1H on C$^{8'}$ and 1H on C$^{7'}$), 2.13 (t, *J* = 5.5 Hz, 1H on C$^{4'}$), 2.06 – 1.89 (m, 1H on C$^{8'}$ and 1H on C$^{6'}$), 1.71 (d, *J* = 10.9 Hz, 1H on C$^{7'}$), 1.55 (s, 3H, CH_3 on C$^{3a'}$), 1.32 (s, 3H, CH_3 on C$^{5'}$), 1.20 (d, *J* = 6.9 Hz, 3H, diastereotopic Me isopropyl), 1.19 (d, *J* = 6.9 Hz, 3H, diastereotopic Me isopropyl), 0.85 (s, CH_3 on C$^{5'}$).
^{13}C NMR (126 MHz, $CDCl_3$) δ 160.4 (C=O), 154.2 (C^5), 135.4 (C^2), 124.5, (C^4) 88.1 (C$^{3a'}$), 78.0 (C$^{7a'}$), 51.7 (OCH_3), 51.0 (C$^{4'}$), 39.6 (C$^{6'}$), 38.4 (C$^{5'}$), 33.9 (C$^{7'}$), 27.9 (CH_3 on C$^{3a'}$), 27.2 (CH_3 on C$^{5'}$), 26.1 (CH_3 on C$^{8'}$), 25.0 (CH on isopropyl), 24.1 (CH_3 on C$^{5'}$), 19.8 (diastereotopic Me isopropyl), 19.7 (diastereotopic Me isopropyl).
IR (CH_2Cl_2) : ṽ/cm^{-1}: 2972, 1715, 1675, 1440, 1361, 1224, 1178, 1146, 1094, 1057, 1036, 783 ṽ/cm^{-1}
$[\alpha]_{20}^{D}$ −14 in CH_2Cl_2 (0.36 g/L)
$[\alpha]_{20}^{365}$ −52 in CH_2Cl_2 (0.36 g/L)

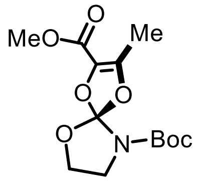

10a

9-(*tert*-Butyl) 2-methyl-3-methyl-1,4,6-trioxa-9-azaspiro[4.4]non-2-ene-2,9-dicarboxylate 10a: Prepared according to *method A* using cyclic carbamate **13a** (35.4 mg, 0.2 mmol) and α-diazo-β-ketoester **11a** (56.8 mg, 0.4 mmol) as precursors. Product **10a** (54 mg, 90% yield) was obtained as a colorless oil after chromatography (SiO_2, eluent: pentane/EtOAc, 95:5 to 90:10).
$\mathbf{R_f}$ 0.33 pentane/EtOAc (80:20). **^{1}H NMR** (500 MHz, $CDCl_3$, 55 °C) δ 4.24 – 4.13 (m, 2H), 3.82 (s, 3H), 3.78 – 3.70 (m, 2H), 2.26 (s, 3H), 1.43 (s, 9H). **^{13}C NMR** (126 MHz, $CDCl_3$, 55 °C) δ 160.3 (C=O), 151.5 (C=O), 146.6 (C=C), 128.0 (C_q), 127.0 (C=C), 82.1 (C_q), 63.9 (CH_2), 51.7 (OCH_3), 44.8 (CH_2), 28.4 ($3xCH_3$), 11.1 (CH_3). **IR** (Neat): 2979, 1713, 1686, 1477, 1442, 1394, 1352, 1250, 1177, 1156, 1106, 1040, 1022, 993, 972, 919, 855, 834, 811, 794, 773, 759, 735, 701, 667, 627. ṽ/cm^{-1} **HRMS** (ESI): m/z calc for $C_{13}H_{19}NO_7Na$: 324.1059; found: 324.1057 $[M + Na]^+$.

$[\alpha]_{20}^{D}$ −57 and +53 in CH_2Cl_2 (0.2 g/L) after CSP-HPLC resolution (see below); (−)- and (+)-**10a** are the first and second eluted enantiomers on CHIRALPAK® IG (Hexane+0.1% Et_2NH/CH_2Cl_2)

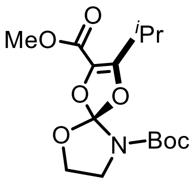

10b

9-(*tert*-Butyl) 2-methyl-3-isopropyl-1,4,6-trioxa-9-azaspiro[4.4]non-2-ene-2,9-dicarboxylate 10b: Prepared according to *method **A*** using cyclic carbamate **13a** (35.4 mg, 0.2 mmol) and α-diazo-β-ketoester **11b** (68.0 mg, 0.4 mmol) as precursors. Product **10b** (64 mg, 97% yield) was obtained as a colorless oil after chromatography (SiO_2, eluent: pentane/EtOAc, 95:5 to 90:10).
R_f 0.38 pentane/EtOAc (80:20). **^{1}H NMR** (500 MHz, $CDCl_3$, 55 °C) δ 4.18 (pd, *J* = 8.2, 6.6 Hz, 2H), 3.81 (s, 3H), 3.78 – 3.70 (m, 2H), 3.43 (dt, *J* = 13.9, 7.0 Hz, 1H), 1.44 (s, 9H), 1.21 (d, *J* = 7.0 Hz 3H), 1.21 (d, *J* = 7.0 Hz 3H). **^{13}C NMR** (126 MHz, $CDCl_3$, 55 °C) δ 160.3 (C=O), 154.5 (C=C), 151.5 (C=O), 128.0 (C_q), 125.0 (C=C), 82.0 (C_q), 63.8 (CH_2), 51.7 (OCH_3), 44.9 (CH_2), 28.5 (3xCH_3), 25.1 (CH), 20.5 (CH_3), 19.2 (CH_3). **IR** (Neat): 2976, 1713, 1675, 1441, 1395, 1360, 1320, 1258, 1163, 1142, 1118, 1092, 1021, 974, 921, 855, 833, 797, 763, 616. $\tilde{v}/cm^{-1}$ **HRMS** (ESI): m/z calc for $C_{15}H_{23}NO_7Na$: 352.1372; found: 352.1378 $[M + Na]^+$.

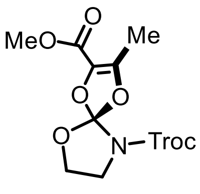

10c

2-Methyl 9-(2,2,2-trichloroethyl) -3-methyl -1,4,6-trioxa-9-azaspiro[4.4]non-2-ene-2,9-dicarboxylate 10c: Prepared according to *method **B*** using cyclic carbamate **13b** (52.4 mg, 0.2 mmol) and α-diazo-β-ketoester **11a** (56.8 mg, 0.4 mmol) as precursors. Product **10c** (53 mg, 70% yield) was obtained as a colorless oil after chromatography (SiO_2, eluent: pentane/EtOAc, 95:5 to 90:10).
R_f 0.30 pentane/EtOAc (80:20). **^{1}H NMR** (500 MHz, DMSO-d_6, 70 °C) δ 4.88 (d, *J* = 2.9 Hz, 1H), 4.28 – 4.20 (m, 1H), 3.83 (td, *J* = 6.8, 2.5 Hz, 1H), 3.72 (s, 1H), 2.19 (s, 1H). **^{13}C NMR** (126 MHz, DMSO-d_6, 70 °C) δ 158.9 (C=O), 149.6 (C=O), 145.7 (C=C), 126.7 (C_q), 125.9 (C=C), 94.7 (C_q), 74.4 (CH_2), 63.6 (CH_2), 51.0 (OCH_3), 44.3 (CH_2), 10.3 (CH_3). **IR** (Neat): 1731, 1692, 1420, 1354, 1253, 1175, 1116, 1033, 818, 760, 719. $\tilde{v}/cm^{-1}$ **HRMS** (ESI): m/z calc for $C_{11}H_{13}Cl_3NO_7$: 375.9758; found: 375.9758 $[M +H]^+$.

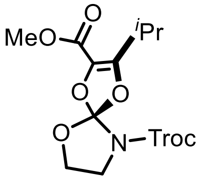

10d

2-Methyl 9-(2,2,2-trichloroethyl) -3-isopropyl -1,4,6-trioxa-9-azaspiro[4.4]non-2-ene-2,9-dicarboxylate 5Bc: Prepared according to *method **B*** using cyclic carbamate **10d** (52.4 mg, 0.2 mmol) and α-diazo-β-ketoester **11b** (68.0 mg, 0.4 mmol) as precursors. Product **10d** (79 mg, 98% yield) was obtained as a colorless oil after chromatography (SiO_2, eluent: pentane/EtOAc, 95:5 to 90:10).
R_f 0.36 pentane/EtOAc (80:20). **^{1}H NMR** (300 MHz, DMSO, 100 °C) δ 5.00 (d, *J* = 12.3 Hz, 1H), 4.76 (d, *J* = 12.3 Hz, 1H), 4.25 (t, *J* = 6.9 Hz, 2H), 3.90 – 3.82 (m, 2H), 3.73 (s, 3H), 3.34 (p, *J* = 6.9 Hz, 1H), 1.15 (d, *J* = 6.9 Hz, 3H), 1.14 (d, *J* = 7.0 Hz, 3H).**^{13}C NMR** (126 MHz, DMSO-d_6, 70 °C) δ 159.0 (C=O), 153.0 (C=C), 149.6 (C=O), 127.4 (C_q), 124.1 (C=C), 94.9 (C_q), 74.1 (CH_2), 63.6 (CH_2), 51.0 (OCH_3), 44.3 (CH_2), 24.0 (CH), 19.4 (CH_3), 18.6 (CH_3). **IR** (Neat): 2975, 1732, 1680, 1416, 1356, 1253, 1193, 1126, 1094, 1031, 814, 781, 761, 718. $\tilde{v}/cm^{-1}$ **HRMS** (ESI): m/z calc for $C_{13}H_{16}Cl_3NO_7$: 425.9890; found: 425.9863 $[M +Na]^+$.

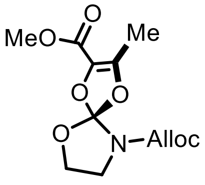

10e

9-Allyl 2-methyl -3-methyl-1,4,6-trioxa-9-azaspiro[4.4]non-2-ene-2,9-dicarboxylate 5Ca: Prepared according to *method A* using cyclic carbamate **10e** (52.4 mg, 0.2 mmol) and α-diazo-β-ketoester **11a** (56.8 mg, 0.4 mmol) as precursors. Product **10e** (34 mg, 60% yield) was obtained as a colorless oil after chromatography (SiO_2, eluent: pentane/EtOAc, 90:10 to 80:20).
$\mathbf{R}_f$ 0.33 pentane/EtOAc (80:20). **^{1}H NMR** (500 MHz, DMSO-d_6, 70 °C) δ 5.87 (td, *J* = 10.9, 5.2 Hz, 1H), 5.29 (dq, *J* = 16.5, 1.5 Hz, 1H), 5.21 (dq, *J* = 10.5, 1.4 Hz, 1H), 4.57 (dt, *J* = 5.4, 1.5 Hz, 2H), 4.25 – 4.13 (m, 2H), 3.75 (m, 2H), 3.73 (s, 3H). **^{13}C NMR** (126 MHz, DMSO-d_6, 70 °C) δ 159.0 (C=O), 150.9 (C=O), 145.6 (C=C), 132.0 (CH vinylic), 127.2 (C_q) 125.6 (C=C), 117.4 (2xCH vinylic), 65.5 (CH_2), 63.6 (CH_2), 51.0 (OCH_3), 43.9 (CH_2), 10.1 (CH_3). **IR** (Neat): 2956, 2922, 1716, 1689, 1441, 1402, 1352, 1251, 1173, 1109, 1030, 993, 915, 824, 760, 730, 647. ṽ/cm^{-1} **HRMS** (ESI): m/z calc for $C_{12}H_{16}NO_7$: 308.0746; found: 308.0763 $[M +Na]^+$.

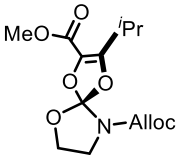

10f

9-Allyl 2-methyl -3-isopropyl-1,4,6-trioxa-9-azaspiro[4.4]non-2-ene-2,9-dicarboxylate 10f: Prepared according to *method A* using cyclic carbamate **13c** (52.4 mg, 0.2 mmol) and α-diazo-β-ketoester **11b** (68.0 mg, 0.4 mmol) as precursors. Product **10f** (47 mg, 75% yield) was obtained as a colorless oil after chromatography (SiO_2, eluent: pentane/EtOAc, 90:10 to 80:20).
$\mathbf{R}_f$ 0.35 pentane/EtOAc (80:20). **^{1}H NMR** (500 MHz, DMSO-d_6, 70 °C) δ 5.85 (ddt, *J* = 16.0, 10.4, 6.5 Hz, 1H), 5.28 (dd, *J* = 17.2, 1.7 Hz, 1H), 5.20 (dd, *J* = 10.5, 1.5 Hz, 1H), 4.62 (ddt, *J* = 13.6, 5.4, 1.5 Hz, 1H), 4.52 (dd, *J* = 13.6, 5.5 Hz, 1H), 4.20 (t, *J* = 6.8 Hz, 2H), 3.76 (t, *J* = 6.8 Hz, 3H), 3.73 (s, 4H), 3.34 (hept, *J* = 6.9 Hz, 1H), 1.13 (d, *J* = 7.0 Hz, 4H), 1.11 (d, *J* = 7.0 Hz, 4H **^{13}C NMR** (126 MHz, DMSO-d_6, 70 °C) δ 159.0 (C=O), 153.0 (C=C), 150.9 (C=O), 132.0 (2xCH vinylic), 127.2 (Cq), 123.8 (C=C), 117.4 (2xCH vinylic), 65.5 (CH_2), 63.5 (CH_2), 51.0 (OCH_3), 44.0 (CH_2), 24.0 (CH), 19.1 (CH_3), 18.8 (CH_3). **IR** (Neat): 2974, 1728, 1678, 1406, 1356, 1254, 1094, 1032, 822, 763. ṽ/cm^{-1} **HRMS** (ESI): m/z calc for $C_{14}H_{19}NO_7Na$: 336.1059; found: 336.1064 $[M +Na]^+$.

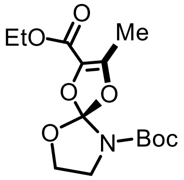

10g

9-(*tert*-Butyl) 2-ethyl-3-methyl-1,4,6-trioxa-9-azaspiro[4.4]non-2-ene-2,9-dicarboxylate 10g: Prepared according to *method B* using cyclic carbamate **13a** (35.4 mg, 0.2 mmol) and α-diazo-β-ketoester **11c** (62.4 mg, 0.4 mmol) as precursors. Product **10g** (47 mg, 75% yield) was obtained as a colorless oil after chromatography (SiO_2, eluent: pentane/EtOAc, 95:5 to 90:10).
$\mathbf{R}_f$ 0.35 pentane/EtOAc (80:20). **^{1}H NMR** (500 MHz, $CDCl_3$, 55 °C) 4.30 (qd, *J* = 7.1, 1.1 Hz, 2H), 4.18 (dddd, *J* = 23.0, 8.2, 7.2, 6.3 Hz, 2H), 3.80 – 3.68 (m, 2H), 2.25 (s, 3H), 1.43 (s, 9H), 1.32 (t, *J* = 7.1 Hz, 3H). **^{13}C NMR** (126 MHz, $CDCl_3$, 55 °C) δ 160.0 (C=O), 151.6 (C=O), 146.3 (C=C), 127.9 (C_q), 127.2 (C=C), 82.0 (C_q), 64.0 (CH_2), 60.8 (OCH_2), 44.7 (CH_2), 28.4 ($3xCH_3$), 14.6 (CH_3), 11.1 (CH_3). **IR** (Neat): 2979, 1713, 1686, 1477, 1393, 1368, 1338, 1250, 1157, 1104, 1040, 1023, 993, 972, 924, 845, 828, 794, 773, 760. ṽ/cm^{-1} **HRMS** (ESI): m/z calc for $C_{13}H_{19}NO_7Na$: 338.1216; found: 338.1200 $[M + Na]^+$.

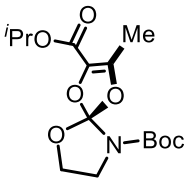

10h

9-(*tert*-Butyl) 2-isopropyl-3-methyl-1,4,6-trioxa-9-azaspiro[4.4]non-2-ene-2,9-dicarboxylate 10h: Prepared according to *method **B*** using cyclic carbamate **13a** (35.4 mg, 0.2 mmol) and α-diazo-β-ketoester **11d** (68.0 mg, 0.4 mmol) as precursors. Product **10h** (49 mg, 74% yield) was obtained as a colorless oil after chromatography (SiO_2, eluent: pentane/EtOAc, 95:5 to 90:10).
$\mathbf{R}_f$ 0.38 pentane/EtOAc (80:20). **^{1}H NMR** (500 MHz, $CDCl_3$, 55 °C) 5.19 (hept, *J* = 6.4 Hz, 1H), 4.19 (dddd, *J* = 24.6, 8.3, 7.2, 6.3 Hz, 2H), 3.81 – 3.69 (m, 2H), 2.25 (s, 3H), 1.43 (s, 9H), 1.31 (d, *J* = 6.3 Hz, 6H). **^{13}C NMR** (126 MHz, $CDCl_3$, 55 °C) δ 159.6 (C=O), 151.6 (C=O), 146.0 (C=C), 127.9 (Cq), 127.4 (C=C), 82.0 (Cq), 68.4 (OCH), 63.9 (CH_2), 44.7 (CH_2), 28.4 ($3xCH_3$), 22.14 (CH_3), 22.11 (CH_3), 11.1 (CH_3). **IR** (Neat): 2980, 1712, 1685, 1476, 1376, 1367, 1321, 1249, 1157, 1125, 1040, 1022, 992, 972, 927, 846, 793, 773, 760. ṽ/cm^{-1} **HRMS** (ESI): m/z calc for $C_{13}H_{19}NO_7Na$: 352.1372; found: 352.1378 $[M + Na]^+$.

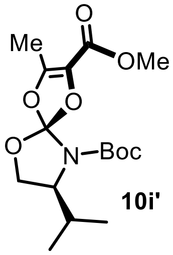

10i'

9-(*tert*-Butyl) 2-methyl (5*S*,8*S*)-8-isopropyl-3-methyl-1,4,6-trioxa-9-azaspiro[4.4]non-2-ene-2,9-dicarboxylate 10i': Prepared together with **10i"** (see below) according to *method **A*** using cyclic carbamate **13e** (45.8 mg, 0.2 mmol) and α-diazo-β-ketoester **11a** (56.8 mg, 0.4 mmol) as precursors. Product **10i'** (26 mg, 38% yield) was obtained as a white solid after chromatography (SiO_2, eluent: pentane/EtOAc, 95:5 to 90:10).
$\mathbf{R}_f$ 0.40 pentane/EtOAc (80:20). **m.p:** 81−83 °C. **^{1}H NMR** (500 MHz, $CDCl_3$) δ 4.13 (dd, *J* = 8.7, 7.0 Hz, 1H), 4.03 (dt, *J* = 12.5, 6.3 Hz, 2H), 3.81 (s, 3H), 2.26 (s, 3H), 2.24 (m, 1H), 1.43 (s, 9H), 0.99 (d, *J* = 6.8 Hz, 3H), 0.93 (d, *J* = 7.0 Hz, 3H).**^{13}C NMR** (126 MHz, $CDCl_3$, 55 °C) δ 160.4 (C=O), 151.7 (C=O), 146.8 (C=C), 128.7 (C_q), 127.0 (C=C), 81.8 (C_q), 65.2 (CH_2), 61.2 (CH), 51.6 (OCH_3), 29.8 (CH), 28.4 ($3xCH_3$), 19.1 (CH_3), 16.5 (CH_3), 11.1 (CH_3). **IR** (Neat): 2962, 2929, 2230, 2160, 1714, 1687, 1443, 1386, 1367, 1354, 1286, 1240, 1174, 1046, 1028, 989, 917, 862, 835, 812, 791, 760, 743, 699, 643. ṽ/cm^{-1} **HRMS** (ESI): m/z calc for $C_{16}H_{25}NO_7$: 366.1529; found: 366.1545 $[M +Na]^+$.

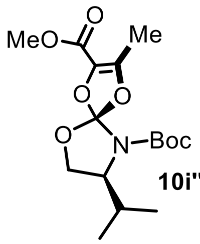

10i"

9-(*tert*-Butyl) 2-methyl (5*R*,8*S*)-8-isopropyl-3-methyl-1,4,6-trioxa-9-azaspiro[4.4]non-2-ene-2,9-dicarboxylate 10i": Prepared together with **10i'** (see above) according to *method **A*** using cyclic carbamate **13e** (45.8 mg, 0.2 mmol) and α-diazo-β-ketoester **11a** (56.8 mg, 0.4 mmol) as precursors. Product **10i'** (6 mg, 8% yield) was obtained as a white solid after chromatography (SiO_2, eluent: pentane/EtOAc, 95:5 to 90:10).
$\mathbf{R}_f$ 0.37 pentane/EtOAc (80:20). **m.p:** 67−69 °C. **^{1}H NMR** (500 MHz, $CDCl_3$, 55 °C) δ 4.20 (dd, *J* = 8.8, 7.4 Hz, 1H), 4.04 – 3.98 (m, 2H), 3.83 (s, 3H), 2.27 (s, 3H), 2.25 (m, 1H), 1.43 (s, 9H), 0.97 (d, *J* = 6.9 Hz, 3H), 0.93 (d, *J* = 7.0 Hz, 3H). **^{13}C NMR** (126 MHz, $CDCl_3$, 55 °C) δ 160.5 (C=O), 151.7 (C=O), 146.6 (C=C), 128.6 (C_q), 127.2 (C_q), 81.8 (C_q), 65.3 (CH_2), 61.1 (CH), 51.7 (OCH_3), 29.7 (CH), 28.4 ($3xCH_3$), 18.9 (CH_3), 16.3 (CH_3), 11.1 (CH_3). **IR** (Neat): 2961, 2928, 2162, 1716, 1688, 1443, 1385, 1367, 1354, 1284, 1220, 1180, 1110, 1045, 1026, 994, 862, 840, 796, 760. ṽ/cm^{-1}

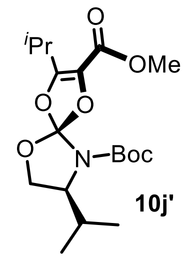
10j'

9-(*tert*-Butyl) 2-methyl (5*S*,8*S*)-3,8-diisopropyl -1,4,6- trioxa-9-azaspiro[4.4]non-2-ene-2,9-dicarboxylate 10j': Prepared together with **10j"** (see below) according to *method A* using cyclic carbamate **13e** (45.8 mg, 0.2 mmol) and α-diazo-β-ketoester **11b** (68.0 mg, 0.4 mmol) as precursors. Product **10j'** (38 mg, 51% yield) was obtained as a colorless oil after chromatography (SiO_2, eluent: pentane/EtOAc, 95:5 to 90:10).
R_f 0.46 pentane/EtOAc (80:20). **^{1}H NMR** (500 MHz, $CDCl_3$,, 55 °C) δ δ 4.11 (dd, *J* = 8.6, 7.0 Hz, 1H), 3.99 (dd, *J* = 8.6, 2.0 Hz, 1H), 3.96 (m, 1H), 3.79 (s, 3H), 3.43 (hept, *J* = 7.0 Hz, 1H), 2.33 – 2.20 (m, 1H), 1.44 (s, 9H), 1.20 (d, *J* = 6.9 Hz, 3H), 1.19 (d, *J* = 7.0 Hz, 3H), 0.98 (d, *J* = 6.9 Hz, 3H), 0.93 (d, *J* = 7.0 Hz, 3H)..**^{13}C NMR** (126 MHz, $CDCl_3$, 55 °C) δ 160.2 (C=O), 154.5 (C=C), 151.4 (C=O), 128.3 (C_q), 124.7 (C=C), 81.5 (C_q), 64.7 (CH_2), 60.9 (OCH_3), 51.2 (CH_2), 28.2 (3xCH_3), 24.8 (CH), 20.3 (CH_3), 18.9 (CH_3), 18.8 (CH_3), 16.2 (CH_3) **IR** (Neat): 2975, 1732, 1680, 1416, 1356, 1253, 1193, 1126, 1094, 1031, 814, 781, 761, 718. $\tilde{v}$/cm^{-1} **HRMS** (ESI): m/z calc for $C_{18}H_{29}NO_7$: 394.1842; found: 394.1833 [M +Na]$^+$.

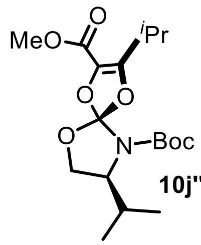
10j"

9-(*tert*-Butyl) 2-methyl (5*R*,8*S*)-3,8-diisopropyl -1,4,6- trioxa-9-azaspiro[4.4]non-2-ene-2,9-dicarboxylate 10j" (see above) according to *method A* using cyclic carbamate **13e** (45.8 mg, 0.2 mmol) and α-diazo-β-ketoester **11b** (68.0 mg, 0.4 mmol) as precursors. Product **10j"** (8 mg, 11% yield) was obtained as a colorless oil after chromatography (SiO_2, eluent: pentane/EtOAc, 95:5 to 90:10).
R_f 0.42 pentane/EtOAc (80:20). **^{1}H NMR** (500 MHz, $CDCl_3$, 55 °C) δ 4.20 – 4.14 (m, 1H), 4.05 – 3.95 (m, 2H), 3.82 (s, 3H), 3.41 (hept, *J* = 6.9 Hz, 1H), 2.26 (pd, *J* = 6.9, 4.7 Hz, 1H), 1.43 (s, 9H), 1.22 (d, *J* = 6.8 Hz, 3H), 1.21 (d, *J* = 7.0 Hz, 3H), 0.97 (d, *J* = 6.9 Hz, 3H), 0.92 (d, *J* = 7.0 Hz, 3H). **^{13}C NMR** (126 MHz, $CDCl_3$, 55 °C) δ 160.5 (C=O), 154.5 (C=C), 151.7 (C=O), 128.5 (C_q), 125.1 (C=C), 81.8 (C_q), 65.2 (CH_2), 61.3 (CH), 51.6 (OCH), 28.5 (3xCH_3), 25.1 (CH), 20.5 (CH_3), 19.0 (CH_3), 18.9 (CH_3), 16.4 (CH_3). **IR** (Neat): 2973, 1714, 1678, 1469, 1441, 1385, 1366, 1321, 1238, 1177, 1142, 1124, 1092, 1046, 1023, 978, 862, 838, 798, 763. $\tilde{v}$/cm^{-1}.

2.3. Reaction on 1 mmol scale

Equation S1. The reaction was then scaled up to 1 mmol of oxazolidinone **13a** with 2 mmol of diazo reagent **11b** to generate 317 mg of product **10b** in an excellent isolated yield of 96%.

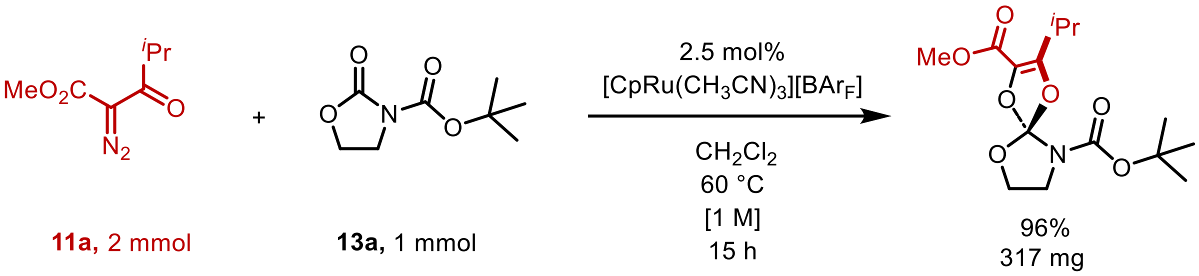

3. Mechanistic rationale of the reaction

Fig. S1. Mechanistic rationale on the decomposition of α-diazo-β-ketoesters **11** by [CpRu(CH3CN)3][BArF] complex **14** (method a) in presence of cyclic carbonates **12** or carbamates **13**. Two acetonitrile moieties can be replaced by a diimine ligand such as 4,4'-dinitro-2,2'-bipyridine (method b).

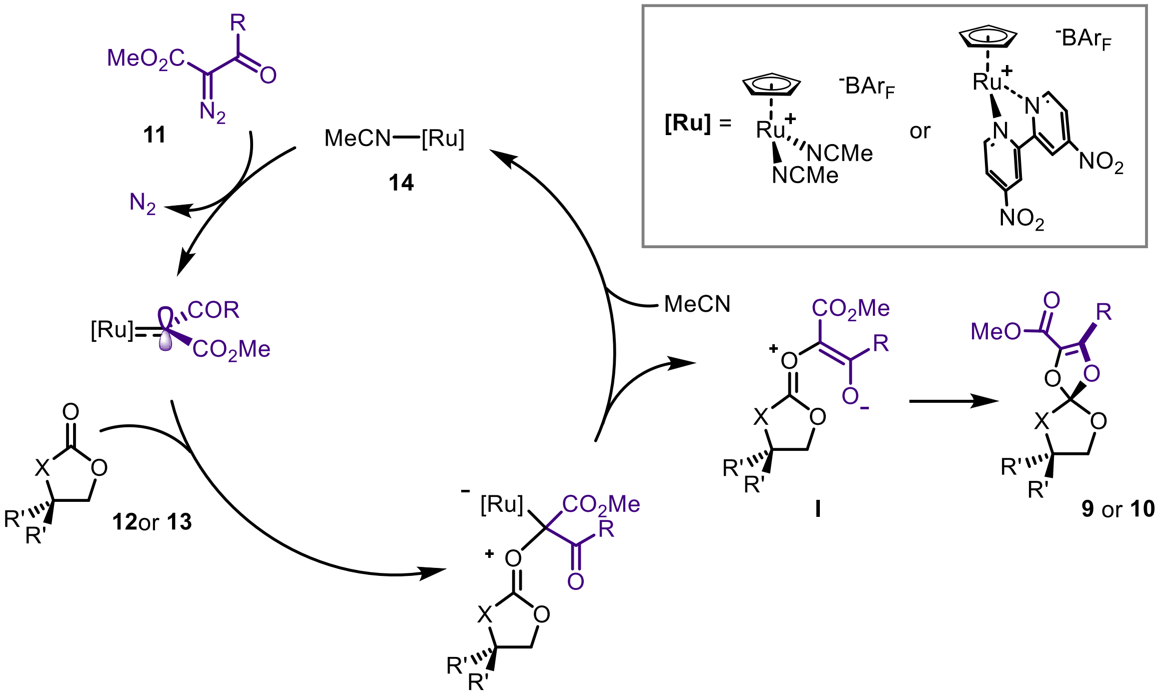

Figure S1 details: This mechanistic rationale is proposed based on literature precedents of transformations between Lewis basic substrates, *e.g.* epoxides or esters, and acceptor-acceptor carbenes generated by decomposition of the corresponding diazo reagents in the presence of CpRu complexes. Steps like the CpRu-catalyzed diazo decomposition, the metal bound ylide and subsequent metal-free ylide formations are substantiated by previous DFT calculations.[14] Of note, metal-free ylide intermediate **I** is also of importance for the racemization / enantiomerization mechanism.

4. HPLC chromatograms

Fig. S2. Chromatogram of semi-preparative HPLC of racemic **9a**. Sample dissolved in CH_2Cl_2 10 mg/mL, 100 µL injection: CHIRALPAK IG (Hexane+0.1% Et_2NH/CH_2Cl_2= 85/15, 4.0 mL min^{-1}, 23 °C, 254 nm) t_R = 19.7, 24.1 minutes.

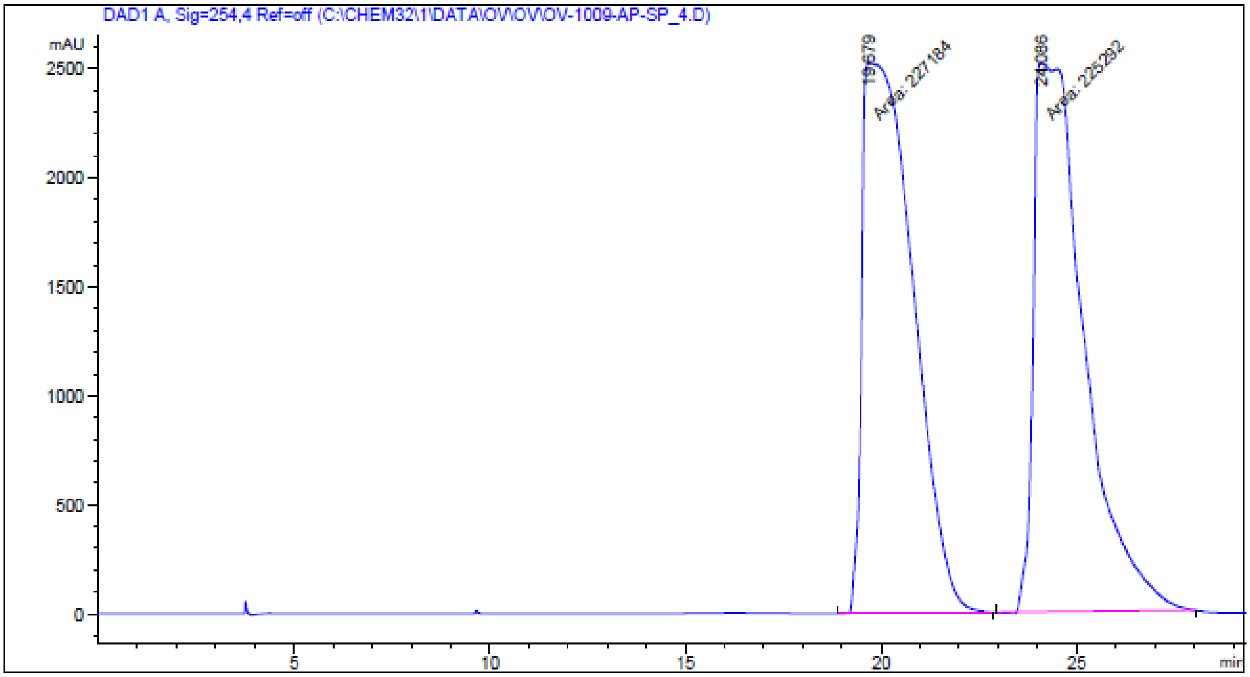

Signal 1: DAD1 A, Sig=254,4 Ref=off

Peak #	RetTime [min]	Type	Width [min]	Area [mAU*s]	Height [mAU]	Area %
1	19.679	MM	1.5022	2.27184e5	2520.60303	50.2091
2	24.086	MM	1.4920	2.25292e5	2516.74854	49.7909
Totals :				4.52477e5	5037.35156	

Fig. S3. Analytical HPLC chromatograms of **9a** dissolved in CH_2Cl_2 1 mg/mL 10 µL injection: CHIRALPAK IG (Hexane+0.1% Et_2NH/CH_2Cl_2= 85/15, 1.0 mL min^{-1}, 23 °C, 254 nm) t_R = 13.7, 15.9 minutes.

Racemic analyte

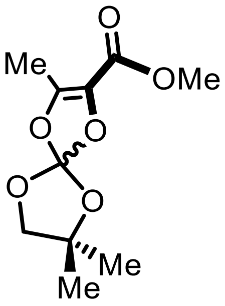

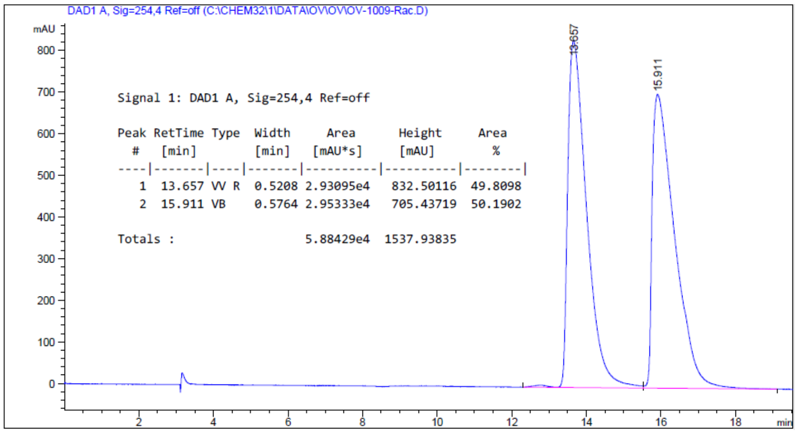

First eluted enantiomer (-)

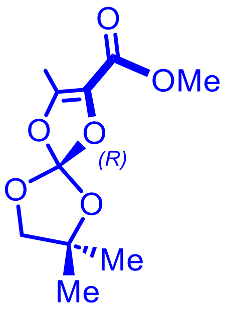

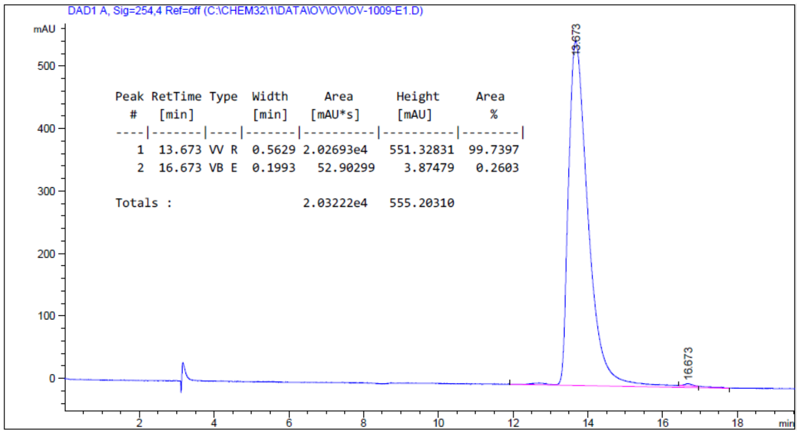

Second eluted enantiomer (+)

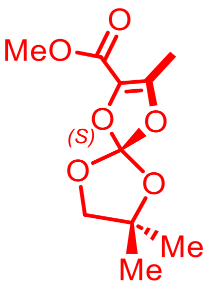

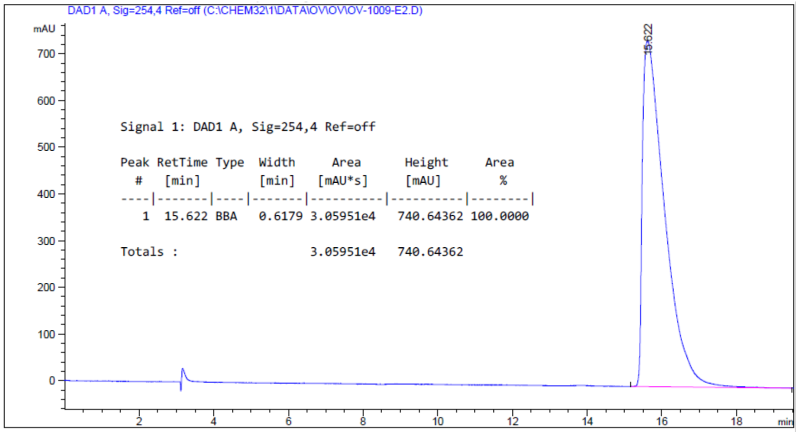

Fig. S4. Analytical HPLC chromatograms of **10a** dissolved in CH_2Cl_2 1 mg/mL 10 μL injection: CHIRALPAK IG (Hexane+0.1% Et_2NH/CH_2Cl_2= 85/15, 1.0 mL min^{-1}, 23 °C, 254 nm) t_R = 25.4, 29.9 minutes

Racemic analyte

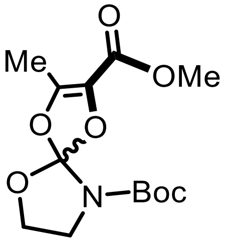

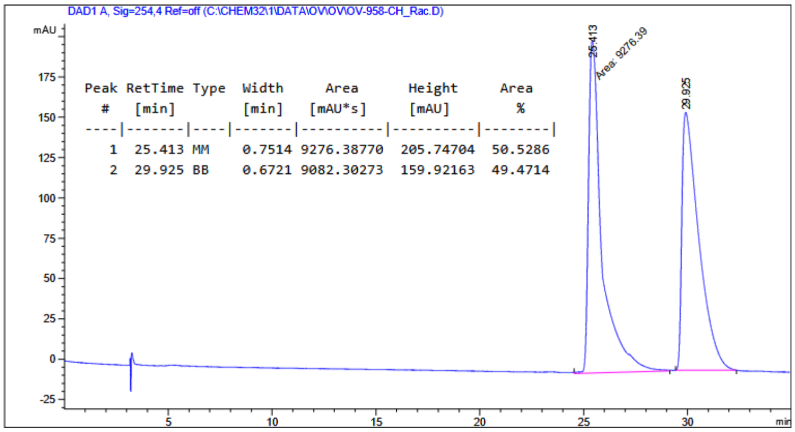

First eluted enantiomer (-)

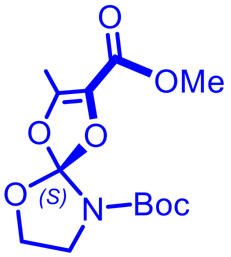

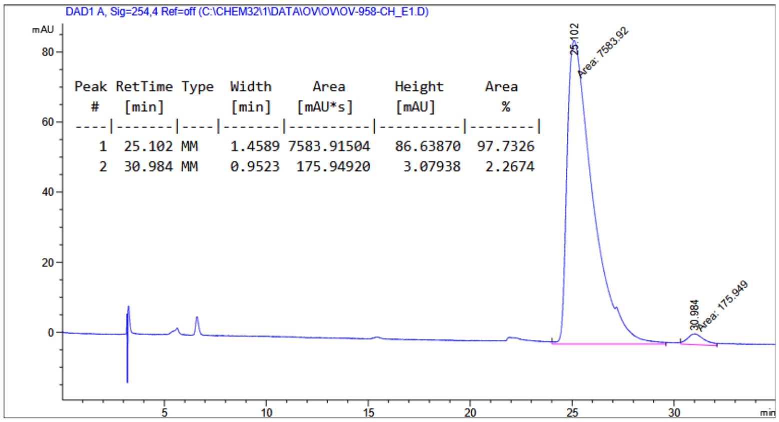

Second eluted enantiomer (+)

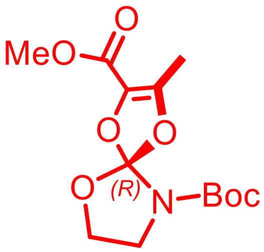

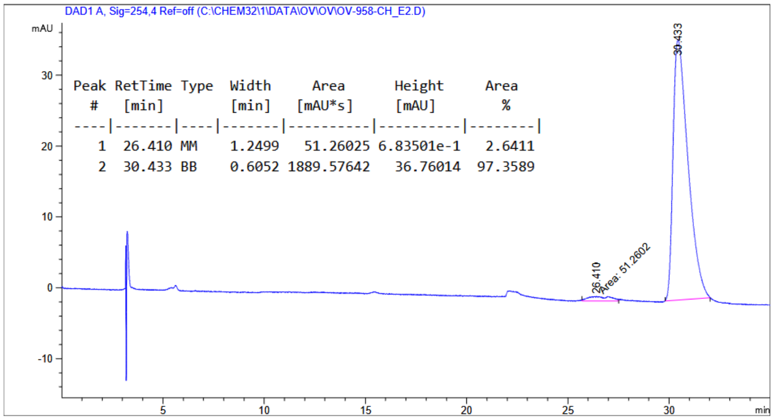

5. UV, ECD, IR and VCD Spectra

Fig. S5. ECD spectra of compound **9a**, first eluted (−)-(*R*) enantiomer (blue) and second eluted (+)-(*S*) enantiomer (red) in air-equilibrated MeCN at RT between 200 and 300 nm (C 1.02 x 10^{-4} M). The underlying filled curves correspond to the respective absorption spectrum. g_{abs} (at 252 nm) -5.1/+5.6x 10^{-5}.

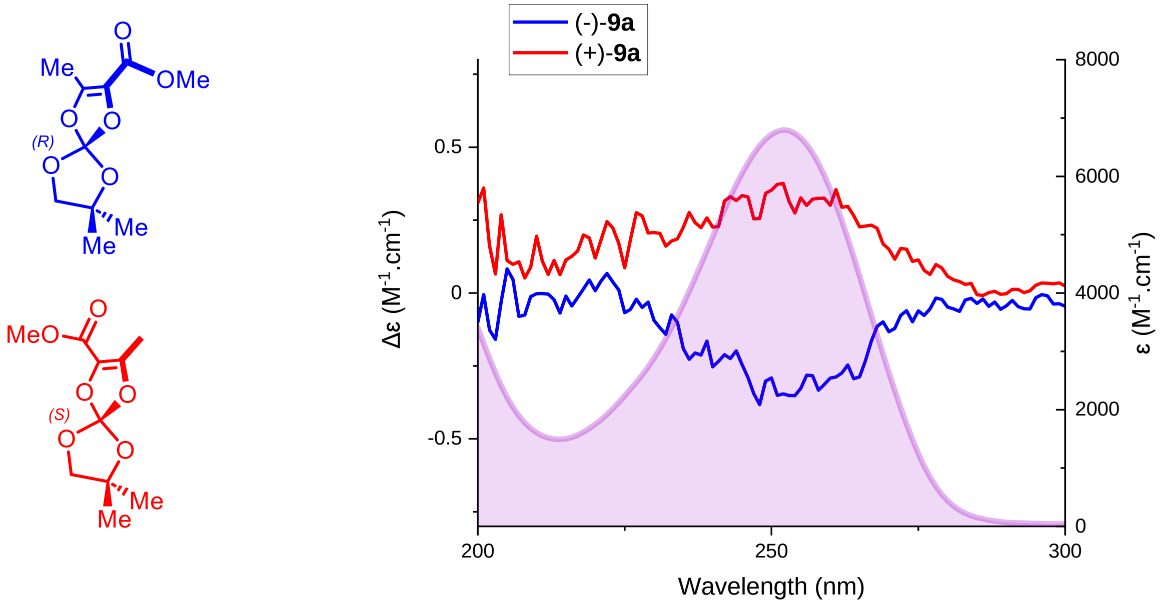

Fig. S6. Absorption spectra of diastereomeric (5*S*,8*S*)-**10j'** and (5*S*,8*R*)-**10j"** in air-equilibrated MeCN at RT between 200 and 400 nm (C *ca*. 5 x 10^{-5} M).

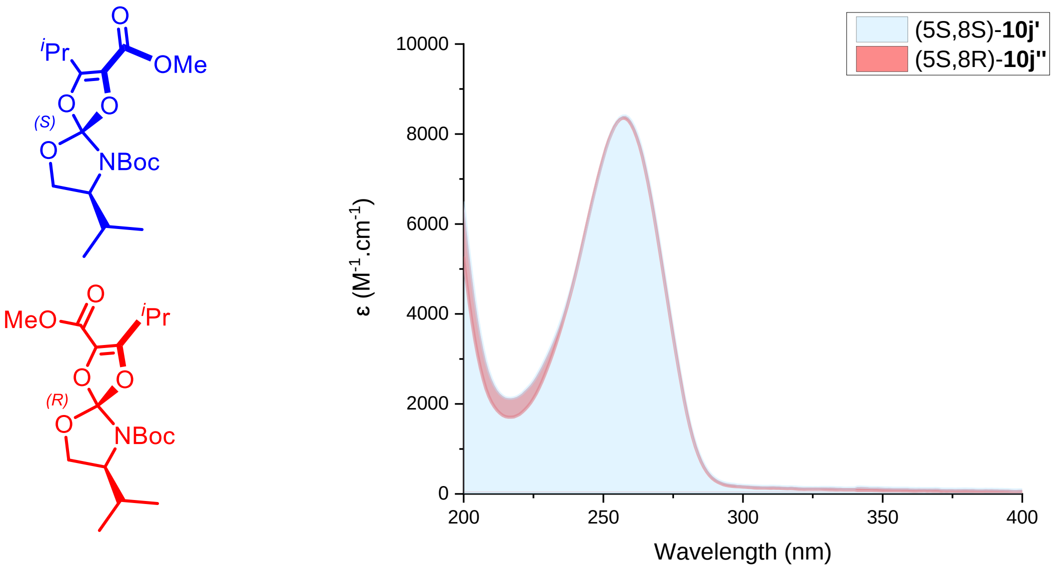

Fig. S7. ECD spectra of first eluted (5*S*,8*S*)-**10j'** (blue) and second eluted (5*S*,8*R*)-**10j"** (red) diastereomers in air-equilibrated MeCN at RT between 200 and 300 nm (C *ca*. 5 x 10^{-5} M). The underlying filled curves correspond to the respective absorption spectrum. g_{abs} (at 255 nm) −3.5/+5.1x 10^{-4}.

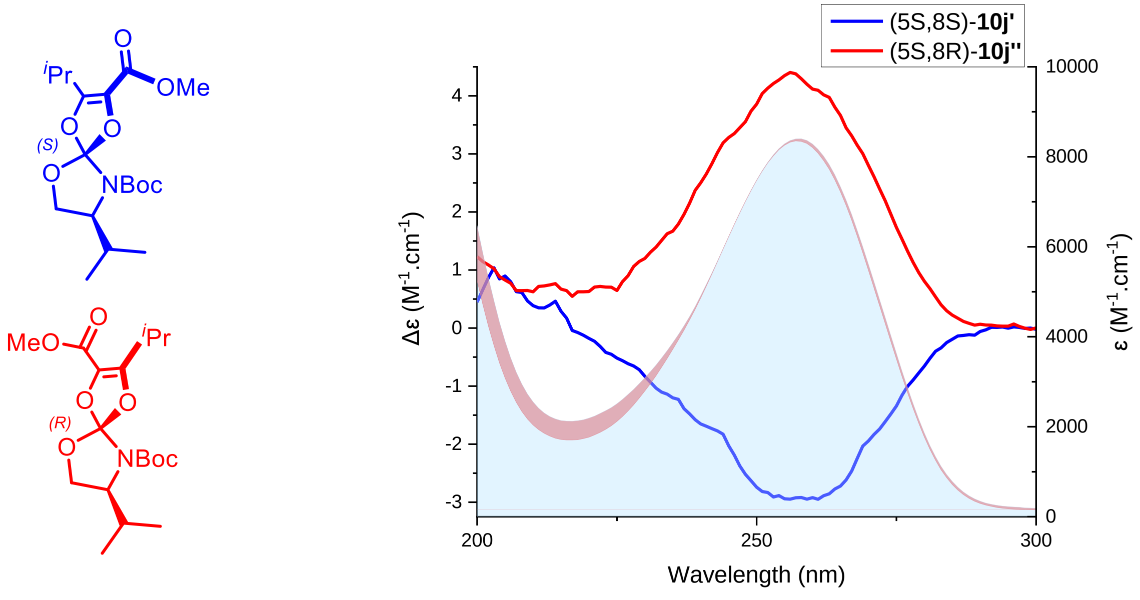

Fig. S8. IR spectra of **9e'** and **9e"** measured in deuterated acetonitrile (11 mg in 300 microliter).

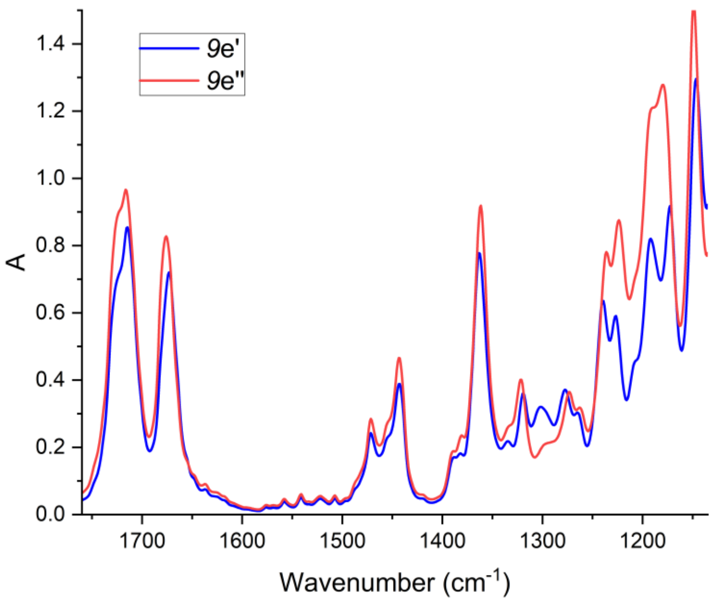

Fig. S9. VCD spectra of **9e’** and **9e”** measured in deuterated acetonitrile (11 mg in 300 microliter).

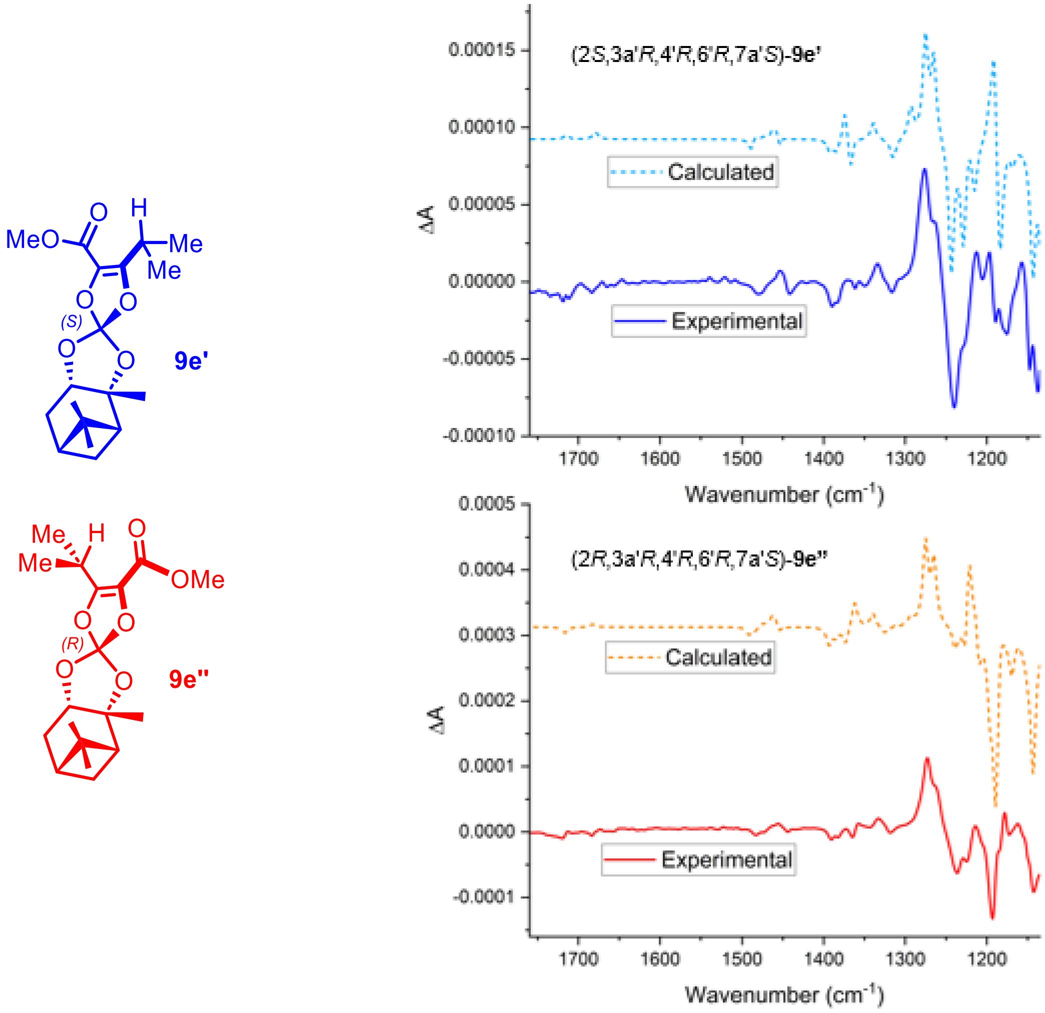

6. Enantiomerization studies by enantioselective dynamic chromatography

Fig. S10. Dynamic GC chromatograms, Eyring plot and activation parameters for compound **9b**. Separation conditions: 25 m fused silica capillary (i.d. 0.25 mm, 0.25 µm film thickness) coated with heptakis(2,3-di-*O*-acetyl-6-*O*-TBDMS) β-cyclodextrin in PS 086 (50% w/w), T = 140-180 °C, 80 kPa He. The orange band represents the confidence interval of the linear regression with a level of confidence of 95%.

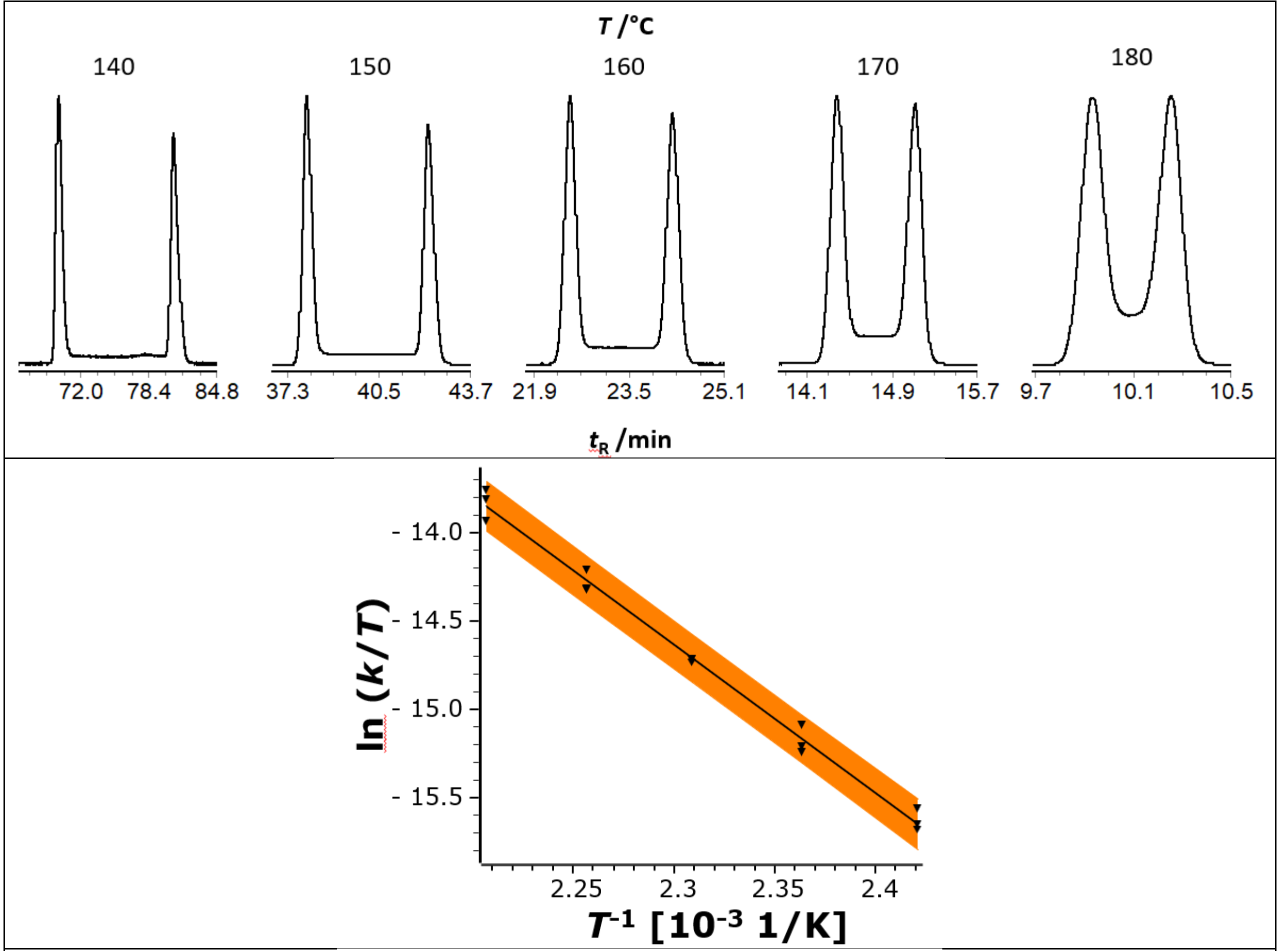

Linear Regression

y = -8.41703E+0003 x + 4.71994E+0000

r = 0.9960

sy = 0.0612

Std deviation slope = 209.1209

Std deviation intercept = 0.4836

$\Delta H^{\ddagger}$ = 69.98 ± 1.74 kJ/mol = 16.7 ± 0.4 kcal/mol

$\Delta S^{\ddagger}$ = -152.5 ± 15.6 J/(K mol) = -36.4 ± 3.7 cal/(K mol)

$\Delta G^{\ddagger}$(298.15K) = 115.46 kJ/mol = 27.6 kcal/mol

Fig. S11. Dynamic GC chromatograms, Eyring plot and activation parameters for compound **10b**. Separation conditions: 8.5 m fused silica capillary (i.d. 0.25 mm, 0.50 µm film thickness) coated with heptakis(2,3-di-*O*-methyl-6-*O*-TBDMS) β-cyclodextrin in PS 086 (50% w/w), T = 105-125 °C, 80 kPa He. The orange band represents the confidence interval of the linear regression with a level of confidence of 95%.

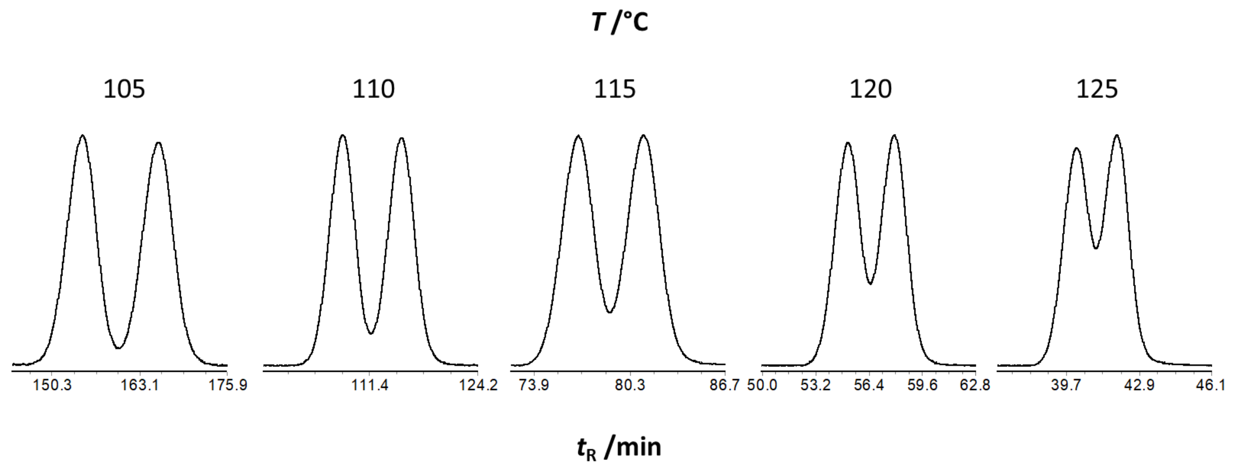

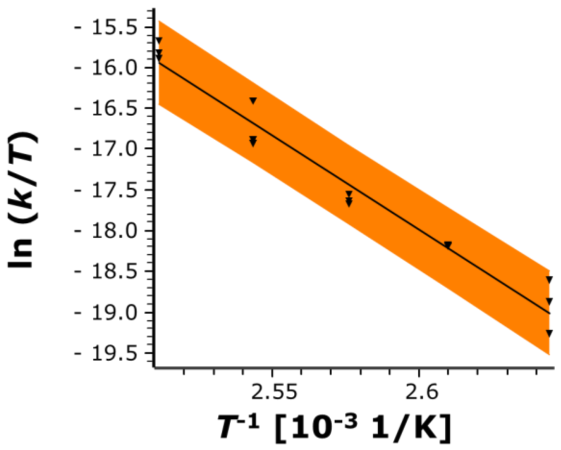

Linear Regression

y = -2.30803E+0004 x + 4.20226E+0001

r = 0.9828

sy = 0.2186

Std deviation slope = 1201.8433

Std deviation intercept = 3.0979

ΔH‡ = 191.90 ± 9.99 kJ/mol = 45.9 ± 2.4 kcal/mol

ΔS‡ = 157.6 ± 11.6 J/(K mol) = 37.7 ± 2.8 cal/(K mol)

ΔG‡ (298.15K) = 144.91 kJ/mol = 34.6 kcal/mol

Fig. S12: Dynamic HPLC chromatograms, Eyring plot and activation parameters for compound **9a**. Separation conditions: CHIRALPAK IG-3 (Hexane/CH_2Cl_2 = 85/15 +0.1% Et_2NH, 1.0 mL min^{-1}, T = 60-80 °C, 254 nm, 1 µL injection). The orange band represents the confidence interval of the linear regression with a level of confidence of 95%.

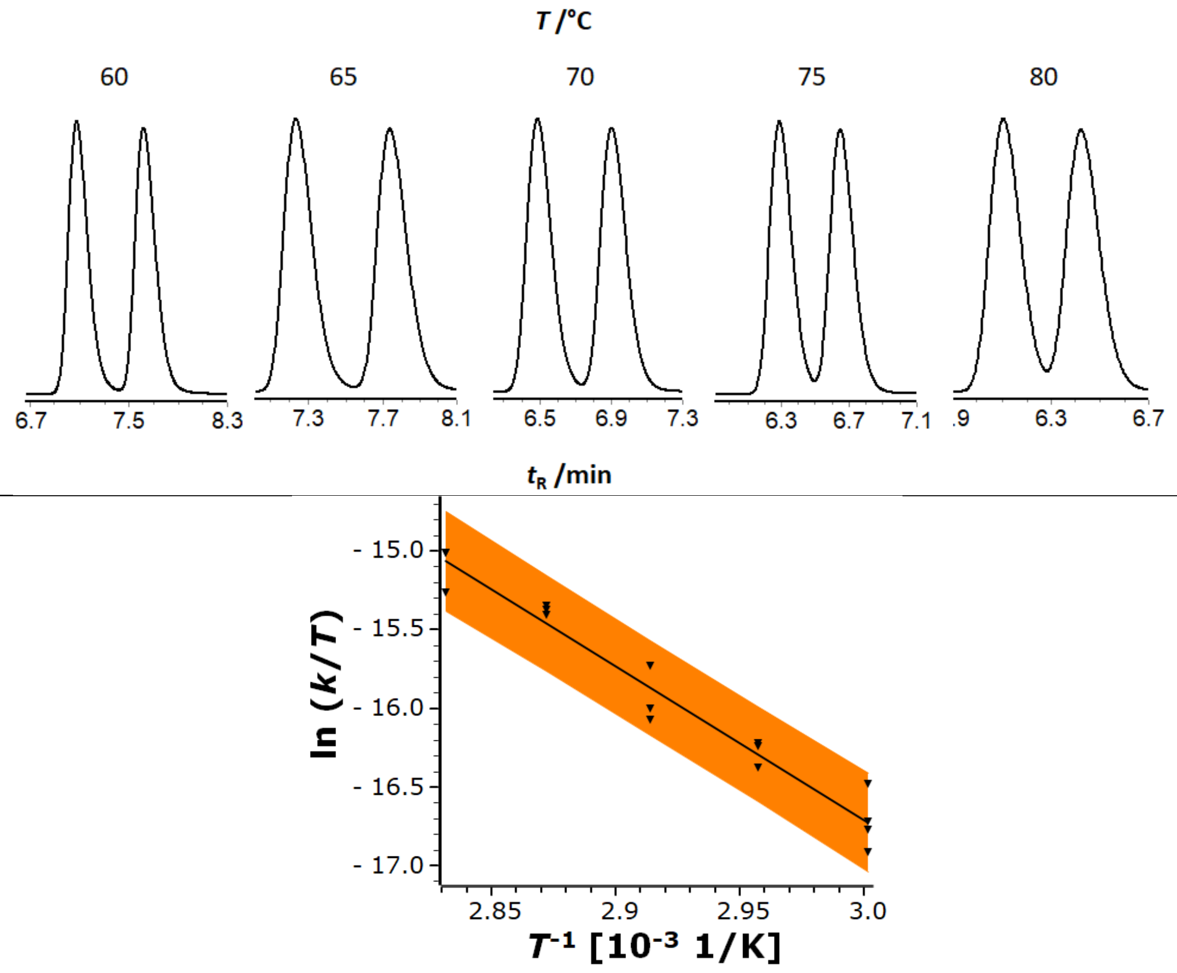

Linear Regression
y = -9.76430E+0003 x + 1.25860E+0001
r = 0.9785
sy = 0.1361

Std deviation slope = 550.5147
Std deviation intercept = 1.6083

$\Delta H^{\ddagger}$ = 81.18 ± 4.58 kJ/mol = 19.4 ± 1.1 kcal/mol
$\Delta S^{\ddagger}$ = -87.1 ± 11.1 J/(K mol) = -20.8 ± 2.7 cal/(K mol)
$\Delta G^{\ddagger}$(298.15K) = 107.17 kJ/mol = 25.6 kcal/mol

7. Computational results

Table S1. Structures, relative Gibbs energies (in kcal/mol) and populations at 300K calculated for the lowest-energy conformers of (*S*)-**9a** at B3LYP-D3BJ/6-311+G(d,p)/PCM (ACN) level.

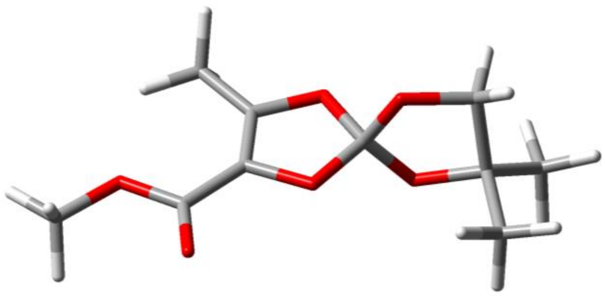 Abs. min. (31.3%)	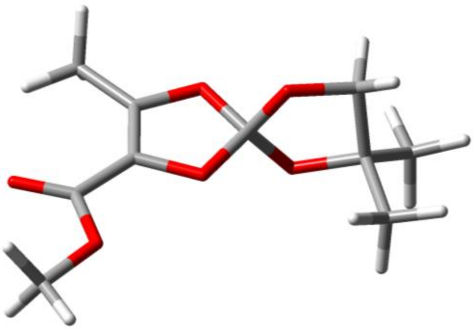 +0.14 (24.9%)
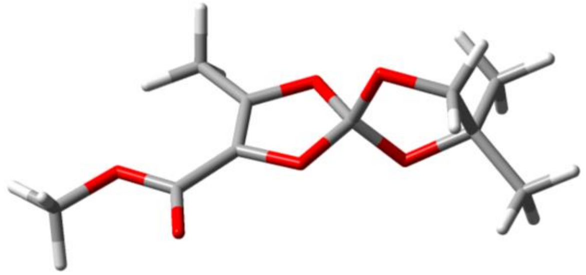 +0.18 (23.2%)	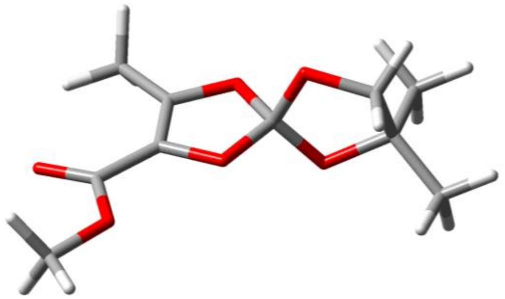 +0.25 (20.6%)

Table S2. Structures, relative Gibbs energies (in kcal/mol) and populations at 300K calculated for the lowest-energy conformers of (2*S*,3a'*R*,4'*R*,6'*R*,7a'*S*)-**9e'** at B3LYP-D3BJ/6-311+G(d,p)/PCM (ACN) level.

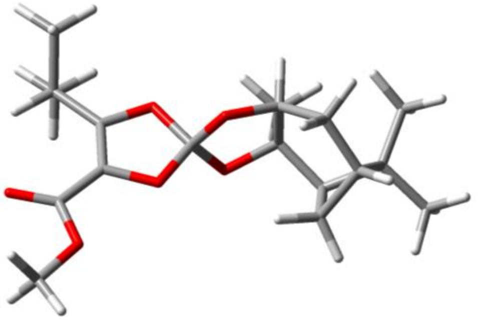 Abs. min. (52.3%)	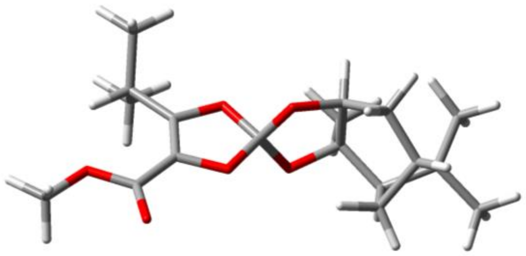 +0.07 (46.1%)
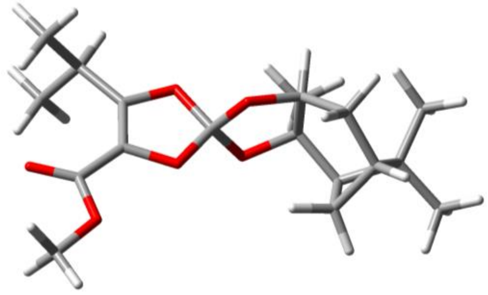 +2.49 (0.8%)	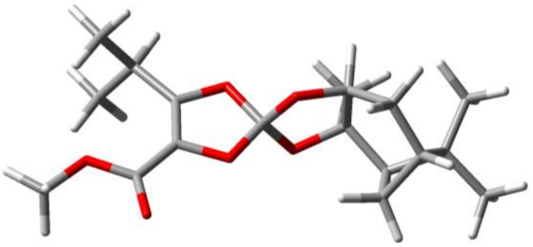 +2.50 (0.8%)

Table S3. Structures, relative Gibbs energies (in kcal/mol) and populations at 300K calculated for the lowest-energy conformers of (2*R*,3a'*R*,4'*R*,6'*R*,7a'*S*)-**9e”** at B3LYP-D3BJ/6-311+G(d,p)/PCM (ACN) level.

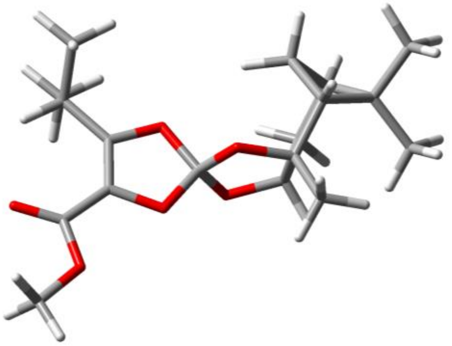 Abs. min. (50.0%)	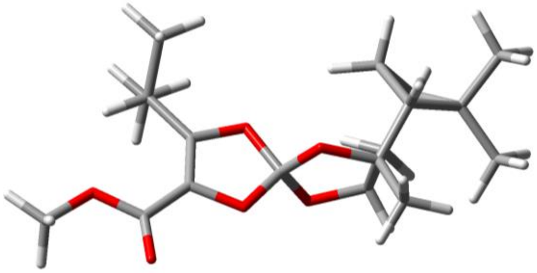 +0.02 (48.6%)
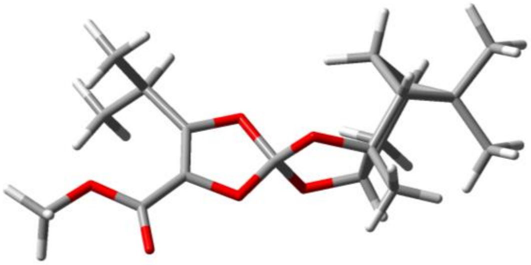 +2.46 (0.8%)	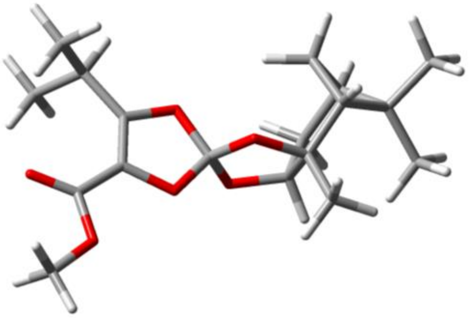 +2.65 (0.6%)

Fig. S13. HOMO and LUMO orbitals involved in the first electronic transition of (*S*)-**9a**, calculated at CAM-B3LYP/aug-cc-pVTZ/PCM//B3LYP-D3BJ/6-311+G(d,p)/PCM (ACN) level for the 4 lowest-energy conformers. The numbers on the arrows are the CI coefficients.

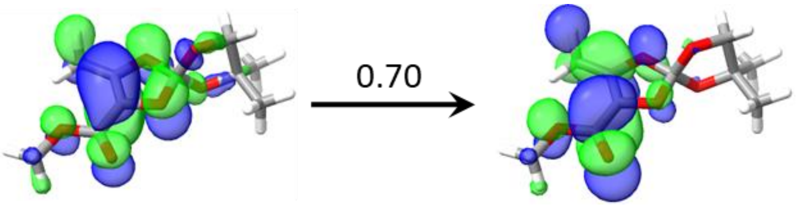

(2*S*)-**9a**, conf. 1

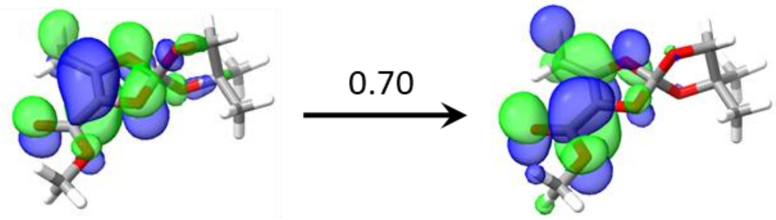

(2*S*)-**9a**, conf. 2

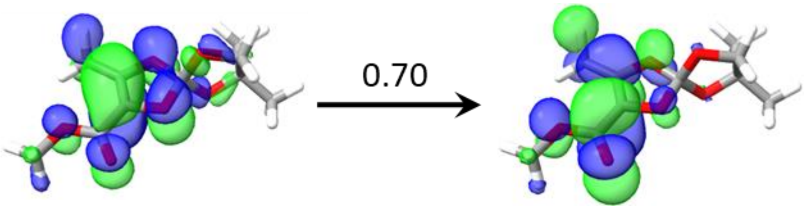

(2*S*)-**9a**, conf. 3

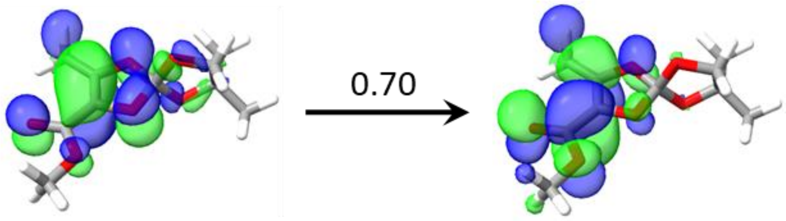

(2*S*)-**9a**, conf. 4

Fig. S14. HOMO and LUMO orbitals involved in the first electronic transition of (2*S*,3a'*R*,4'*R*,6'*R*,7a'*S*)-**9e'**, calculated at CAM-B3LYP/aug-cc-pVTZ/PCM//B3LYP-D3BJ/6-311+G(d,p)/PCM (ACN) level for the 2 lowest-energy conformers. The numbers on the arrows are the CI coefficients.

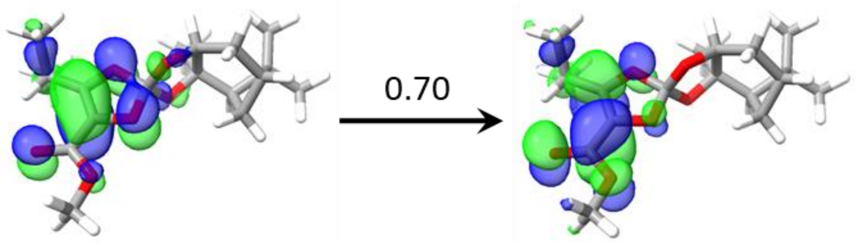

(2*S*)-**9e'**, s-*cis*

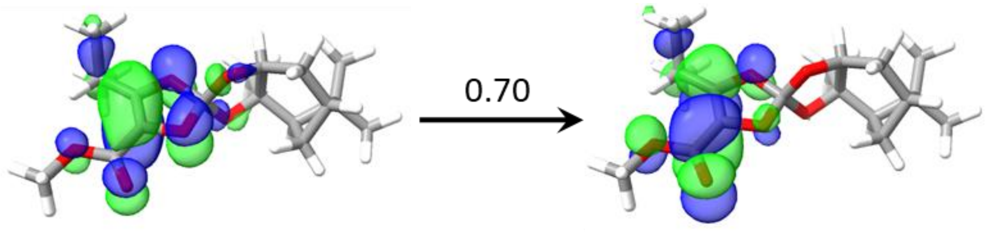

(2*S*)-**9e'**, s-*trans*

Fig. S15. HOMO and LUMO orbitals involved in the first electronic transition of (2*R*,3a'*R*,4'*R*,6'*R*,7a'*S*)-**9e”**, calculated at CAM-B3LYP/aug-cc-pVTZ/PCM//B3LYP-D3BJ/6-311+G(d,p)/PCM (ACN) level for the 2 lowest-energy conformers. The numbers on the arrows are the CI coefficients.

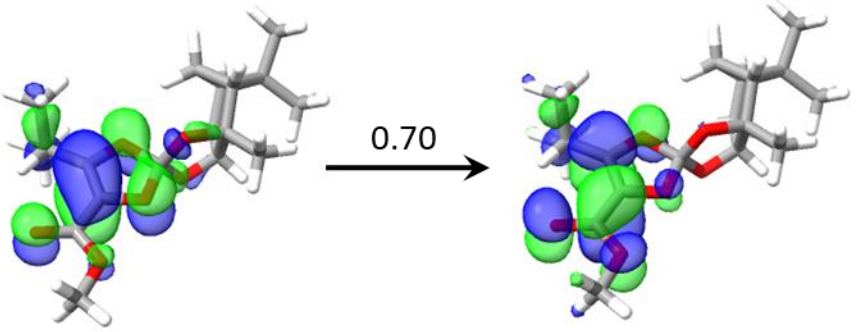

(2*R*)-**9e”**, s-*cis*

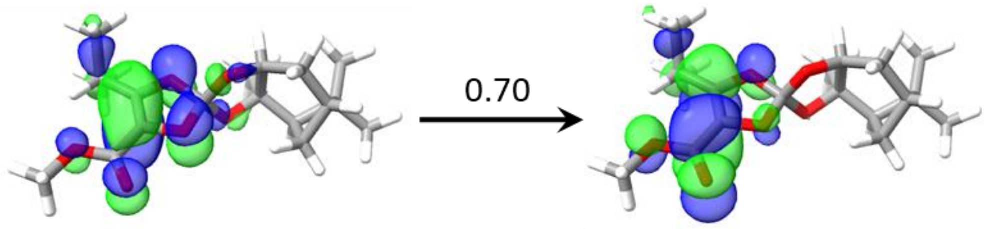

(2R)-**9e”**, s-*trans*

Fig. S16. Comparison between experimental (solid lines) and calculated (dotted lines) ECD spectra of (2*S*,3a'*R*,4'*R*,6'*R*,7a'*S*)-**9e’** (blue) and (2*R*,3a'*R*,4'*R*,6'*R*,7a'*S*)-**9e”** (red).

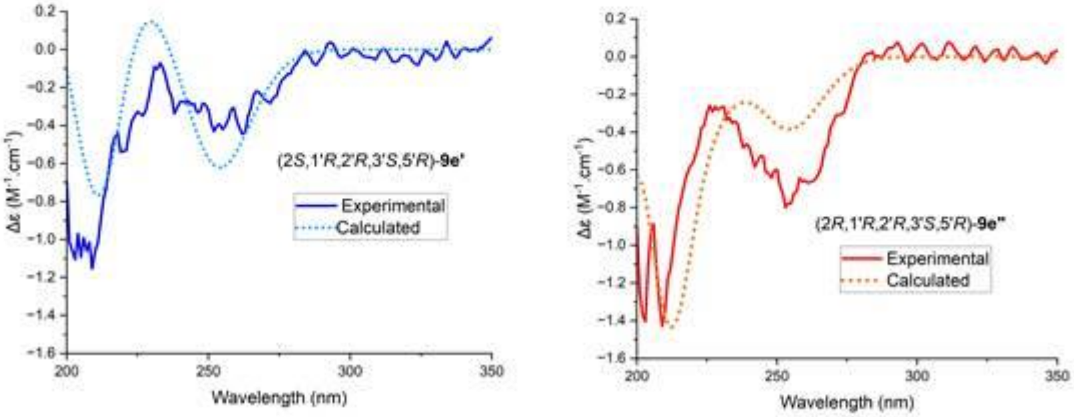

Fig. S17. Partition of the space around the chromophore in sectors defined by the chromophore plane of symmetry (green, horizontal) and the nodal orbital planes (gray and brown, vertical) shown for the lowest-energy s-*cis* conformers of (2*S*,3a'*R*,4'*R*,6'*R*,7a'*S*)-**9e'** and (2*R*,3a'*R*,4'*R*,6'*R*,7a'*S*)-**9e''**. Highlighted in yellow on the left and right panels is the position of the methyl group attached at C2', which occupies a negative sector in both epimers.

Structure	Octant definition	Octant sign and C2'-methyl position
2*S* 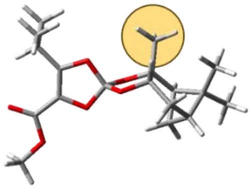	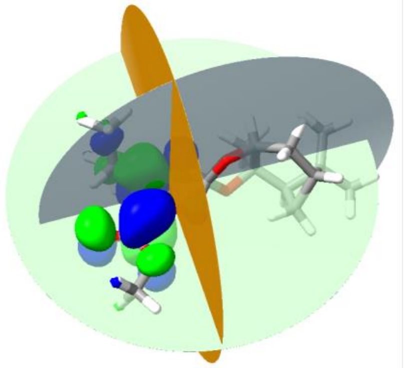	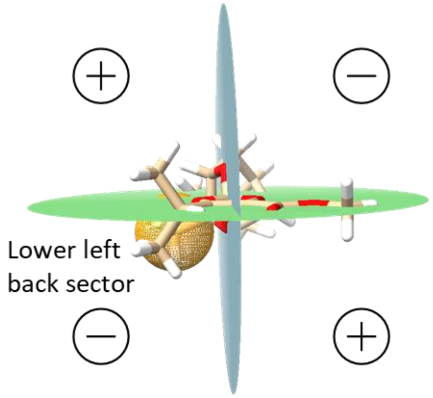
2*R* 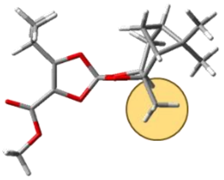	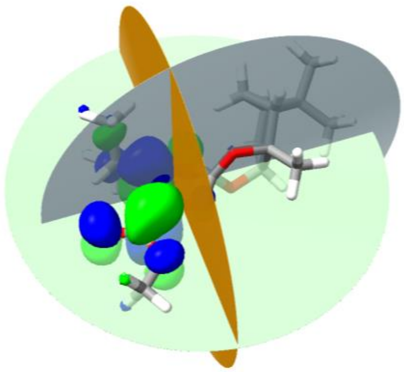	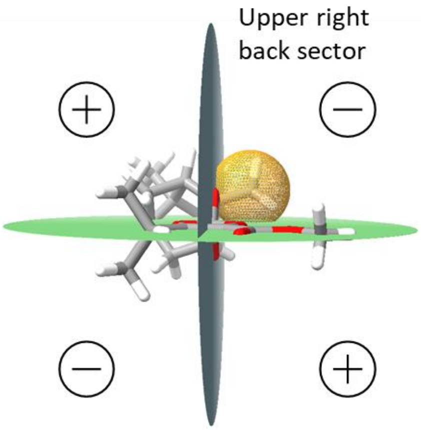

Fig. S18. ECD spectra calculated at CAM-B3LYP/aug-cc-pVTZ/PCM//B3LYP-D3BJ/6-311+G(d,p)/PCM (ACN) level for the lowest-energy s-*cis* conformers of (2*S*,3a'*R*,4'*R*,6'*R*,7a'*S*)-**9e'** and (2*R*,3a'*R*,4'*R*,6'*R*,7a'*S*)-**9e"** and for the respective truncated models where only the three carbon atoms attached at C1' and C2' were retained (and capped with H atoms).

Diagrams<br>(full molecules and models)	Truncated model<br>optimized structure	Calculated spectra
2*S* 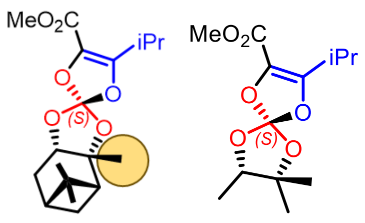	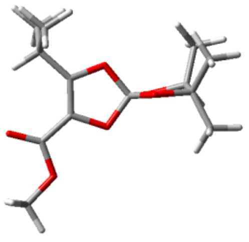	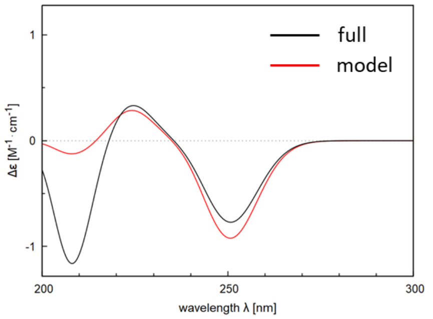
2*R* 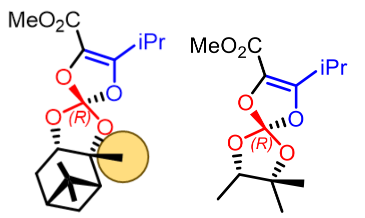	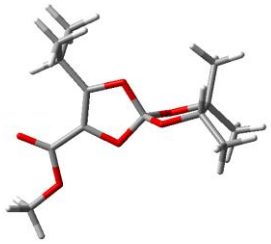	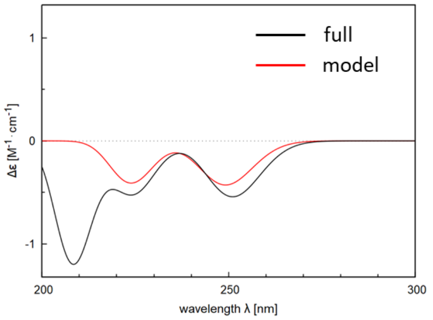

The consistent negative sign found in all cases for the ECD band around 250 nm (π-π* transition) demonstrates that this latter is dominated by the orientation of the methyl group attached at the C1' position.

Table S4. Structures, relative Gibbs energies (in kcal/mol) and populations at 300K calculated for the lowest-energy conformers of (*R*)-**10a** at B3LYP-D3BJ/6-311+G(d,p)/PCM (ACN) level.

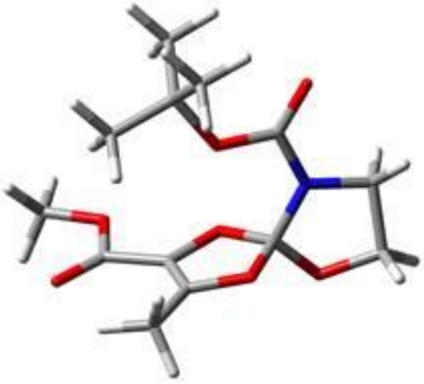<br>Abs. min. (40.9%)[a]	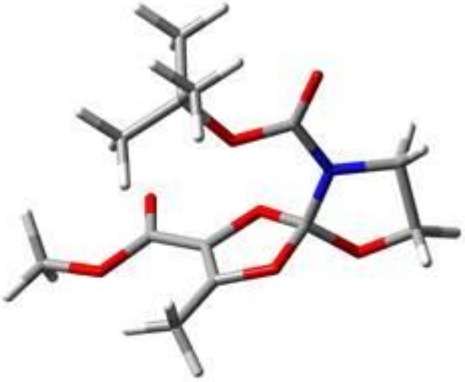<br>+0.39 (21.2%)[b]
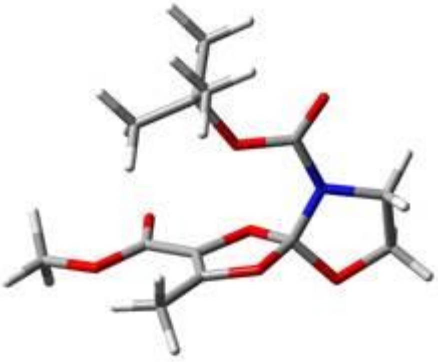<br>+0.51 (17.1%)[b]	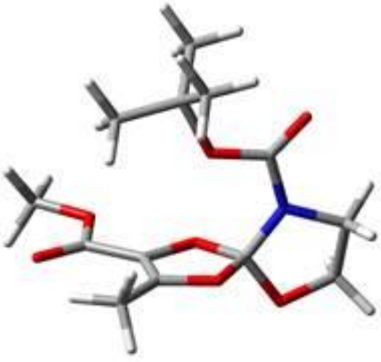<br>+0.55 (16.1%)[a]
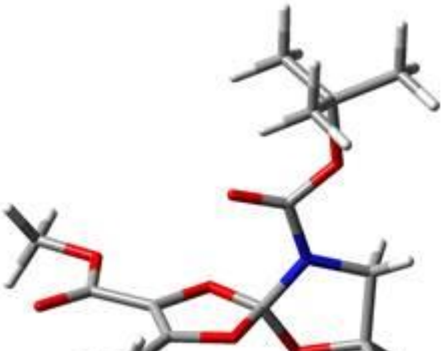<br>+1.58 (2.8%)[c]	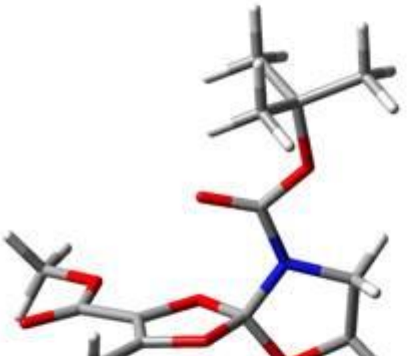<br>+1.85 (1.8%)[c]

[a,b,c] These conformers differ in the puckering of the oxazolidine ring.

Table S5. Structures, relative Gibbs energies (in kcal/mol) and populations at 300K calculated for the lowest-energy conformers of (5*R*,8*S*)- **10i'** at B3LYP-D3BJ/6-311+G(d,p)/PCM (ACN) level. Two conformers with population < 1% were included in the calculations but not listed.

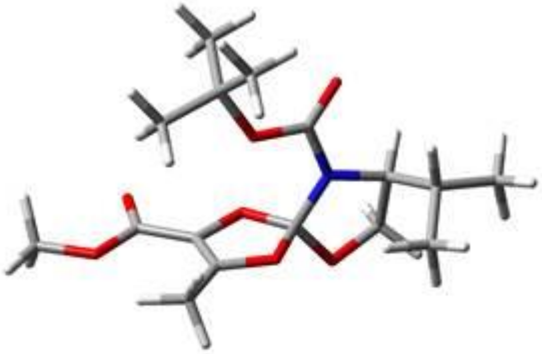<br>Abs. min. (36.3%)	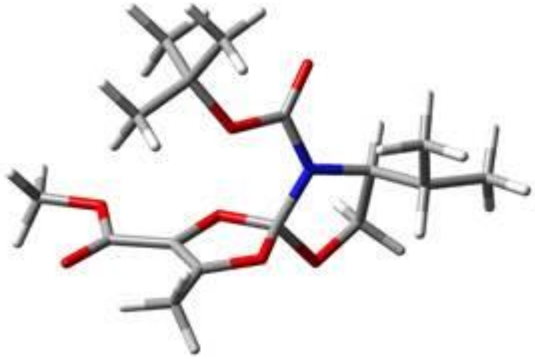<br>+0.24 (24.3%)
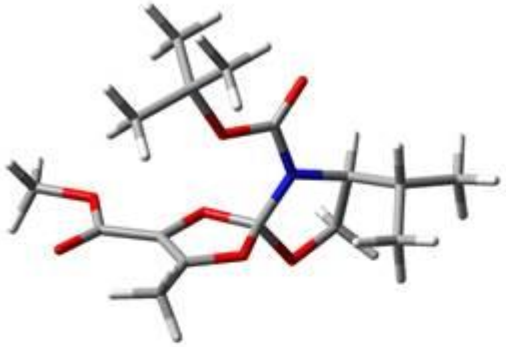<br>+0.31 (21.5%)[a]	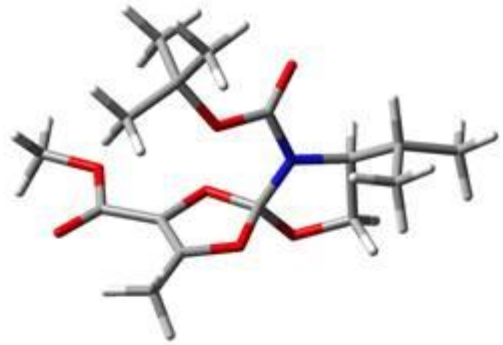<br>+0.83 (8.9%)[a]
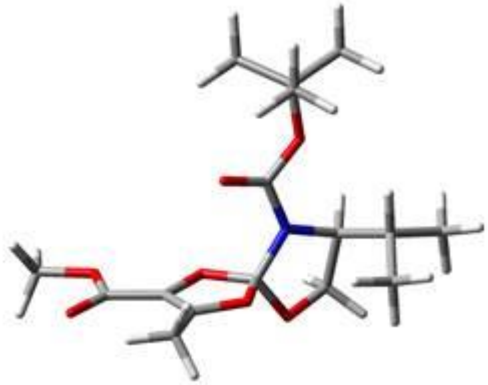<br>+1.12 (5.4%)	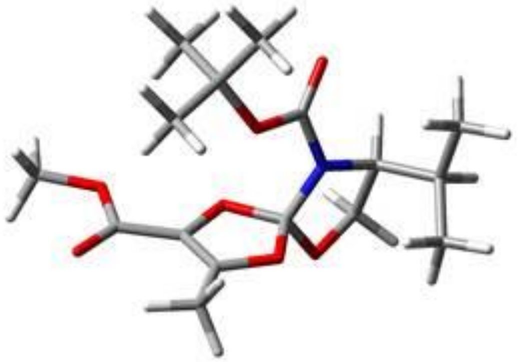<br>+1.64 (2.3%)

[a] These conformers differ in the puckering of the oxazolidine ring.

Table S6. Structures, relative Gibbs energies (in kcal/mol) and populations at 300K calculated for the lowest-energy conformers of (5*S*,8*S*)-**10i”** at B3LYP-D3BJ/6-311+G(d,p)/PCM (ACN) level. Two conformers with population < 1.5% were included in the calculations but not listed.

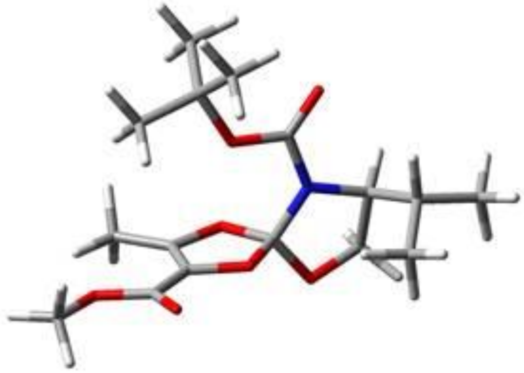 Abs. min. (48.4%)	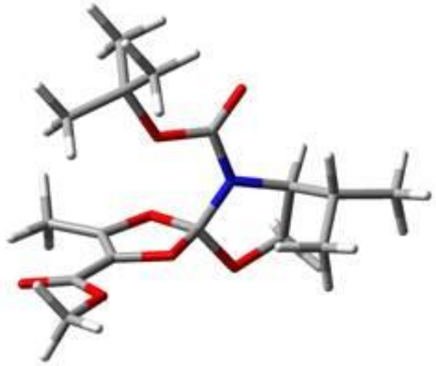 +0.28 (30.0%)
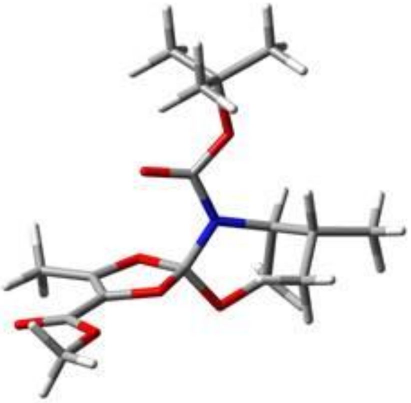 +1.12 (7.3%)	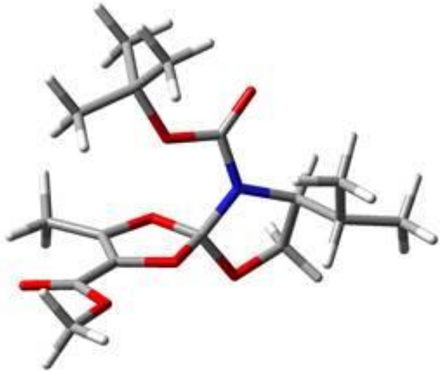 +1.24 (6.0%)
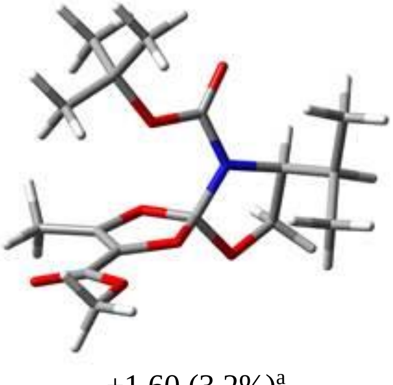 +1.60 (3.2%)[a]	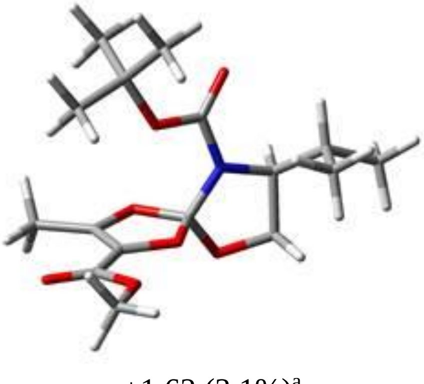 +1.62 (3.1%)[a]

[a] These conformers differ in the puckering of the oxazolidine ring.

Table S7. Structures, relative Gibbs energies (in kcal/mol) and populations at 300K calculated for the lowest-energy conformers of (5*R*,8*S*)-**10j’** at B3LYP-D3BJ/6-311+G(d,p)/PCM (ACN) level. One conformer with population < 2% was also included in the calculations but not listed.

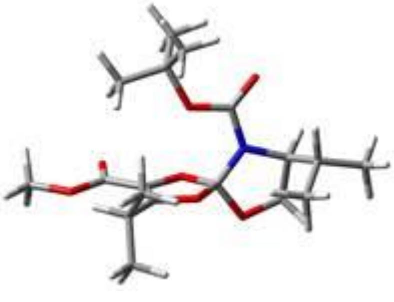<br>Abs. min. (38.9%)	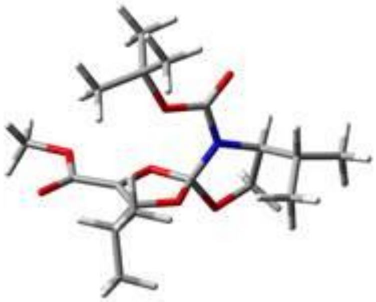<br>+0.38 (20.4%)
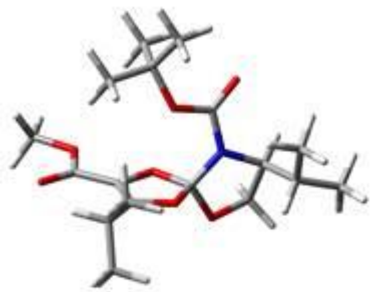<br>+0.83 (9.5%)[a]	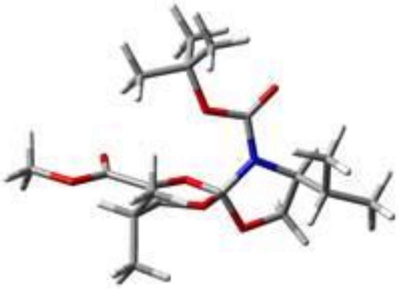<br>+0.96 (7.7%)[a]
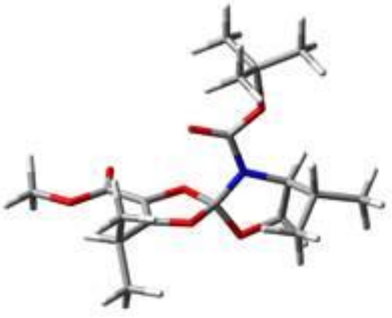<br>+0.98 (7.5%)	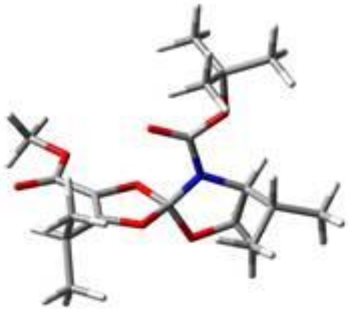<br>+1.07 (6.4%)
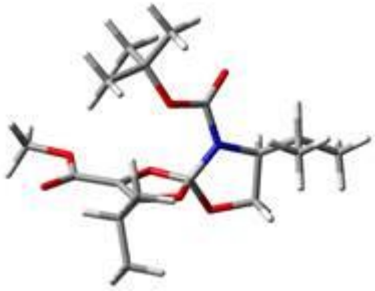<br>+1.26 (4.6%)	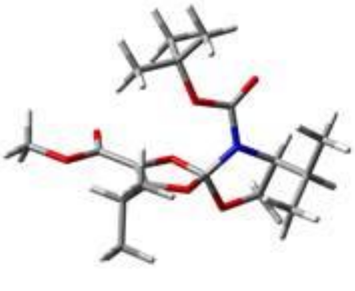<br>+1.45 (3.3%)

Table S8. Structures, relative Gibbs energies (in kcal/mol) and populations at 300K calculated for the lowest-energy conformers of (5*S*,8*S*)-**10j”** at B3LYP-D3BJ/6-311+G(d,p)/PCM (ACN) level. Five conformers with population < 2% were also included in the calculations but not listed.

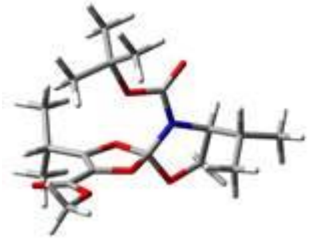<br>Abs. min. (39.4%)	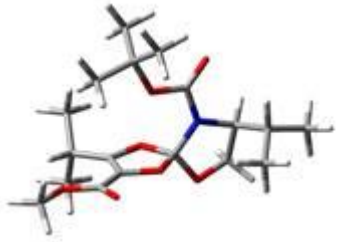<br>+0.21 (27.6%)
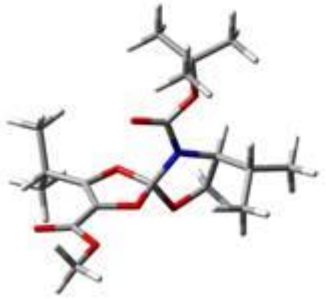<br>+0..69 (12.3%)[a]	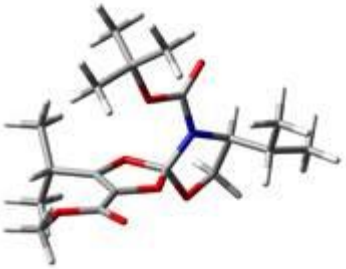<br>+1.15 (5.6%)[a]
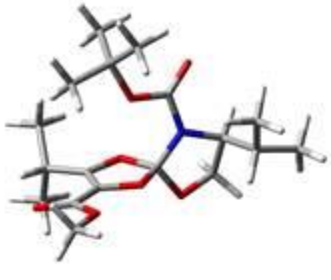<br>+1.31 (4.3%)	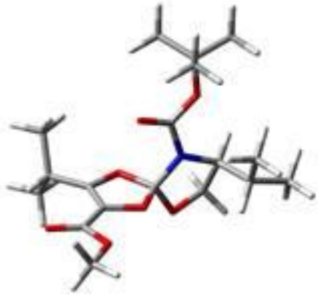<br>+1.63 (2.5%)
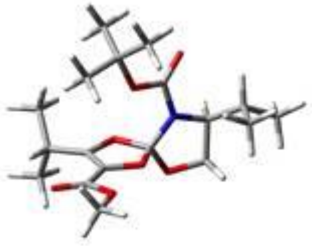<br>+1.64 (2.5%)	

Fig. S19. HOMO and LUMO orbitals involved in the first electronic transition of (*R*)-**10a**, calculated at CAM-B3LYP/def2-TZVP/PCM//B3LYP-D3BJ/6-311+G(d,p)/PCM (ACN) level for the respective lowest-energy conformers. The numbers on the arrows are the CI coefficients.

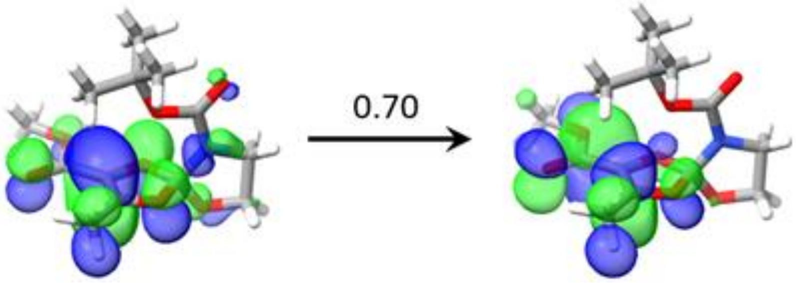

(*R*)-**10a**

Fig. S20. HOMO and LUMO orbitals involved in the first electronic transition of (5*R*,8*S*)-**10i'** and (5*S*,8*S*)-**10i''**, calculated at CAM-B3LYP/def2-TZVP/PCM//B3LYP-D3BJ/6-311+G(d,p)/PCM (ACN) level for the respective lowest-energy conformers. The numbers on the arrows are the CI coefficients.

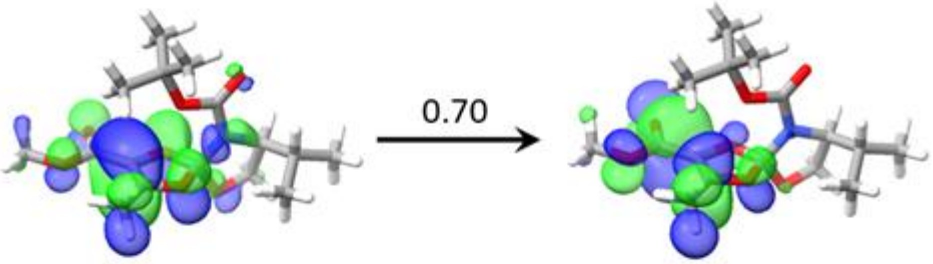

(5*R*,8*S*)-**10i'**

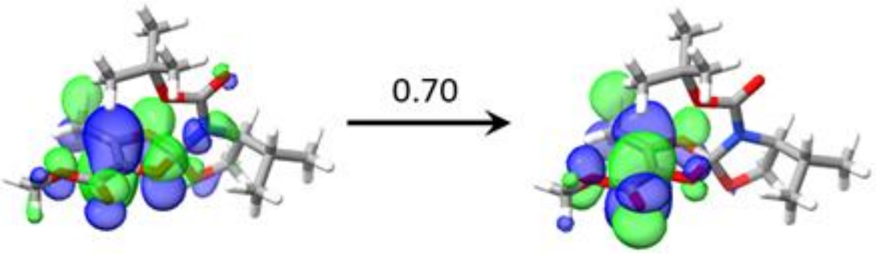

(5*S*,8*S*)-**10i''**

Fig. S21. (A) HOMO and LUMO orbitals of the zwitterionic transition state **TS’** of compound **9a** obtained after O–C bond breaking from **9a**, calculated at B3LYP-D3BJ/6-311+G(d,p)/PCM (ACN) level. (B) Structure of **TS’** highlighting the conjugated planar moiety (H atoms removed for clarity) and NCI analysis. Measurements of relevant angles and dihedral angles are listed below. (C) Putative structure of **TS”** of compound **10a** corresponding to **TS’**, with measurements and NCI analysis. Additional steric contacts involving the carbamate group are the orange areas indicated by red arrows.

(A)

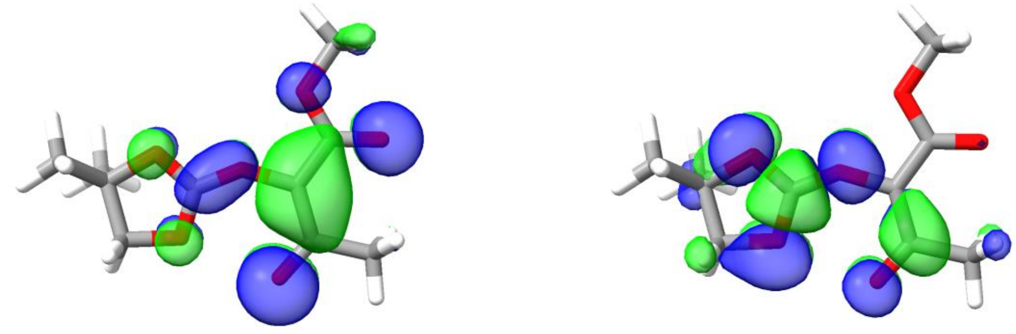

HOMO LUMO

(B)

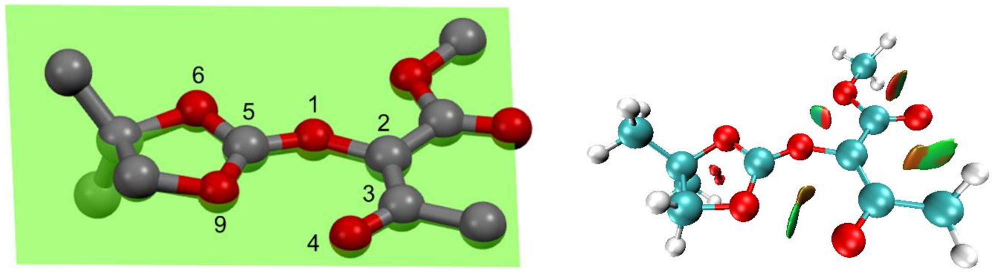

<u>Angles</u>
O1-C2-C3, 121.8°; C2-C3-O4, 123.1°; C5-O1-C2, 141.8°; O1-C5-O6, 116.2°; O1-C5-O9, 128.5°.
<u>Dihedrals</u>
O1-C2-C3-O4, –0.45°; C3-C2-O1-C5, 4.1°; C2-O1-C5-O6, 174.2°; C2-O1-C5-O9, –6.9°.

(C)

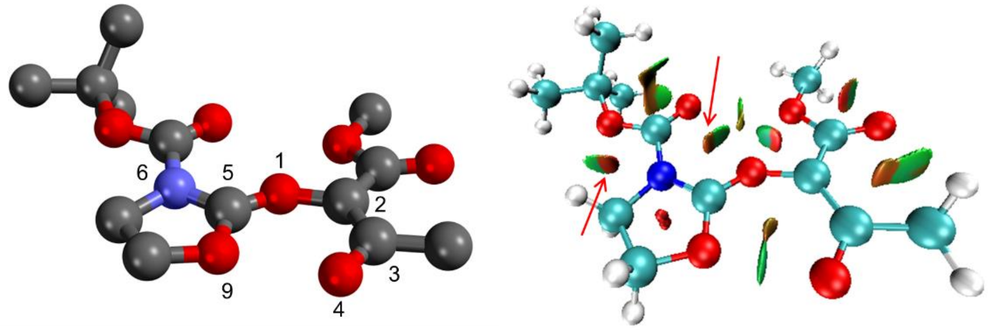

<u>Angles</u>
O1-C2-C3, 123.2°; C2-C3-O4, 124.1°; C5-O1-C2, 139.6°; O1-C5-N6, 121.7°; O1-C5-O9, 126.0°.

Fig. S.22. Comparison between experimental (solid lines) and calculated (dotted lines) ECD spectra of (5*S*,8*S*)-**10i'** (blue) and (5*R*,8*S*)-**10i''** (red).

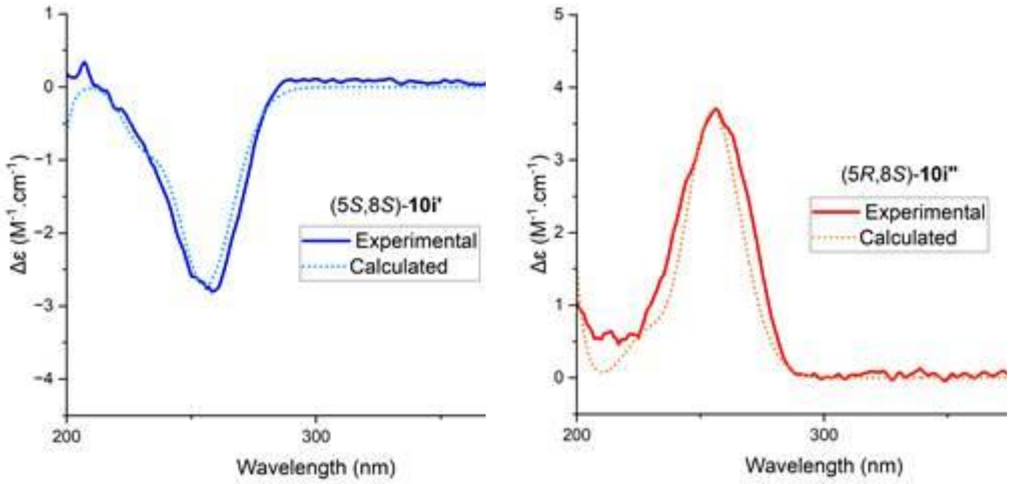

Fig. S23. Comparison between experimental (solid lines) and calculated (dotted lines) ECD spectra of (5*S*,8*S*)-**10j'** (blue) and (5*R*,8*S*)-**10j''** (red).

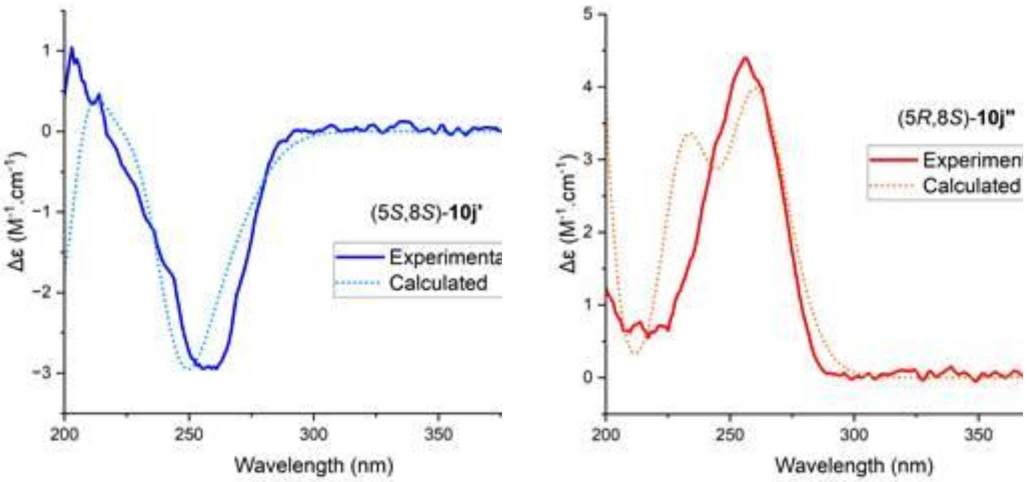

8. X-Ray Crystallographic Data

Fig. S24. View of the asymmetric unit for (2*S*,3a'*R*,4'*R*,6'*R*,7a'*S*)-**9e'** (displacement parameters at 50 percent probability level)

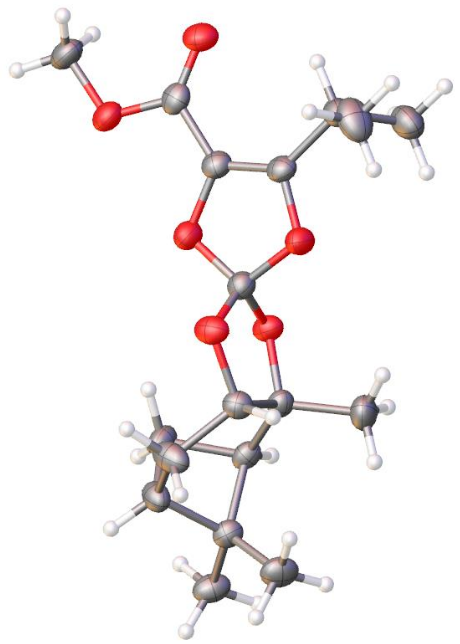

Table S9. Crystal data and structure refinement for **9e'**

CCDC number	2406355
Empirical formula	C18 H26 O6
Formula weight	338.39
Temperature	100(1) K
Wavelength	1.54184 Å
Crystal system	Orthorhombic
Space group	$P2_12_12_1$
Unit cell dimensions	
a = 6.06136(7) Å	α= 90°
b = 15.77865(19) Å	β= 90°
c = 18.7204(2) Å	γ = 90°
Volume	1790.43(4) Å3
Z	4
Density (calculated)	1.255 Mg/m3
Absorption coefficient	0.773 mm-1
F(000)	728
Crystal size	0.403 x 0.285 x 0.049 mm3
Theta range for data collection	3.664 to 73.673°.

Index ranges	-7≤ h ≤7, -19≤ k ≤18, -22≤ l ≤22
Reflections collected	36087
Independent reflections	3475 [R(int) = 0.0628]
Completeness to theta = 67.684°	99.9 %
Absorption correction	Gaussian
Max. and min. transmission	1.000 and 0.266
Refinement method	Full-matrix least-squares on F2
Data / restraints / parameters	3475 / 0 / 223
Goodness-of-fit on F2	1.088
Final R indices [I>2sigma(I)]	R1 = 0.0430, wR2 = 0.1161
R indices (all data)	R1 = 0.0458, wR2 = 0.1197
Absolute structure parameter	-0.18(12)
Largest diff. peak and hole	0.327 and -0.210 e.Å-3

Comments

The resonant scattering is weak for this sample (Friedif 35) as reflected by the large error of the refined Flack parameter (0.0(3)). However, all indicators confirm the absolute structure (Hooft -0.12(6) with P3(true)=1 and the post refinement Parsons-Flack x determined using 1264 quotients is -0.18(12)

Fig. S25. View of the asymmetric unit for (2*R*,3a'*R*,4'*R*,6'*R*,7a'*S*)-**9e”** (displacement parameters at 50 percent probability level)

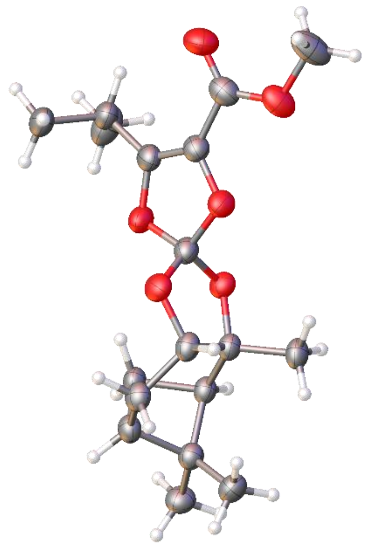

Table S10. Crystal data and structure refinement for **9e”**.

CCDC number	2406356
Empirical formula	C18 H26 O6
Formula weight	338.39
Temperature	120.15 K
Wavelength	1.54184 Å
Crystal system	Monoclinic
Space group	P 1 21 1
Unit cell dimensions	
a = 8.26951(16) Å	α= 90°
b = 11.14258(17) Å	β= 100.4748(17)°
c = 9.67458(17) Å	γ = 90°
Volume	876.60(3) Å3
Z	2
Density (calculated)	1.282 Mg/m3
Absorption coefficient	0.789 mm-1
F(000)	364
Crystal size	0.346 x 0.231 x 0.129 mm3
Theta range for data collection	4.648 to 74.812°.
Index ranges	-8≤h ≤10, -13≤ k ≤13, -12≤ l ≤12
Reflections collected	20364
Independent reflections	3497 [R(int) = 0.0417]
Completeness to theta = 67.684°	99.8 %
Absorption correction	Gaussian
Max. and min. transmission	1.000 and 0.556
Refinement method	Full-matrix least-squares on F2

Data / restraints / parameters	3497 / 1 / 223
Goodness-of-fit on F2	1.105
Final R indices [I>2sigma(I)]	R1 = 0.0379, wR2 = 0.1067
R indices (all data)	R1 = 0.0381, wR2 = 0.1069
Absolute structure parameter	0.00(10)
Largest diff. peak and hole	0.172 and -0.184 e.Å-3

Comments

The resonant scattering is weak for this sample (Friedif 35) as reflected by the large error of the refined Flack parameter (-0.1(2)). However, all indicators confirm the absolute structure (Hooft -0.05(4) with P3(true)=1 and the post refinement Parsons-Flack x determined using 1573 quotients is 0.00(10)

Fig. S26. View of the asymmetric unit for (5*S*,8*S*)-**10i’** (displacement parameters at 50 percent probability level)

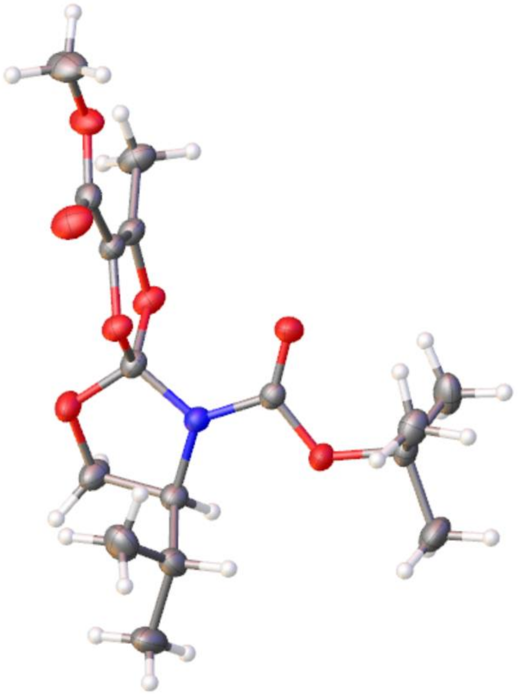

Table S11. Crystal data and structure refinement for **10i’**.

CCDC number	2440356	
Identification code	ov1022	
Empirical formula	C16 H25 N O7	
Formula weight	343.37	
Temperature	120.00(10) K	
Wavelength	1.54184 Å	
Crystal system	Orthorhombic	
Space group	$P2_12_12_1$	
Unit cell dimensions	a = 9.10960(9) Å	a= 90°
b = 10.58981(10) Å	b= 90°	
c = 18.99069(17) Å	g = 90°	
Volume	1832.01(3) Å3	
Z	4	
Density (calculated)	1.245 Mg/m3	
Absorption coefficient	0.820 mm-1	

F(000)	736
Crystal size	0.102 x 0.065 x 0.03 mm3
Theta range for data collection	4.657 to 75.604°.
Index ranges	-11≤ h ≤10, -12≤ k ≤12, -23≤ l ≤22
Reflections collected	43128
Independent reflections	3732 [R(int) = 0.0275]
Completeness to theta = 67.684°	100.0 %
Absorption correction	Semi-empirical from equivalents
Max. and min. transmission	1.00000 and 0.72915
Refinement method	Full-matrix least-squares on F2
Data / restraints / parameters	3732 / 0 / 224
Goodness-of-fit on F2	1.047
Final R indices [I>2sigma(I)]	R1 = 0.0256, wR2 = 0.0666
R indices (all data)	R1 = 0.0259, wR2 = 0.0670
Absolute structure parameter	0.06(3)
Extinction coefficient	n/a
Largest diff. peak and hole	0.119 and -0.188 e.Å-3

Comments

The resonant scattering is weak for this sample (Friedif 35) as reflected by the large error of the refined Flack parameter 0.00(15). However, all indicators confirm the absolute structure (Hooft 0.07(3) with P3(true)=1 and the post refinement Parsons-Flack x determined using 1576 quotients is -0.04(3)

Table S12. selected geometrical parameters. The numbering scheme is shown below. For **9e'** and **9e"** C6 is the first carbon of the $CH(CH_3)_2$ group and for **10i'** a CH_3 group. The atom in green is an oxygen atom in **9e'** and **9e"** and a nitrogen atom in **10i'**. The plane P1 is the mean plane containing C3 C2 O1 C1 O2 and P2 is the mean plane containing C1 O6/N6 C8 C7 O5,

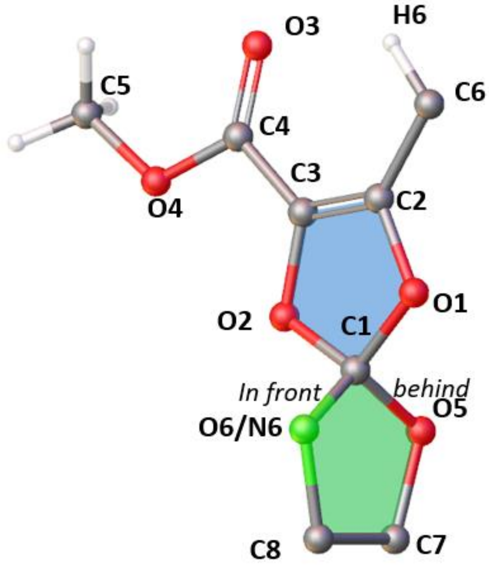

	**9e'**	**9e"**	**10i'**
RMSD plane *P*1 (Å)	0.017	0.001	0.035
RMSD plane *P*2 (Å)	0.077	0.057	0.125
O1C1O2 (°)	105.8(2)	105.7(2)	105.8(1)
O1C1O5 (°)	111.2(2)	109.2(2)	110.7(1)
O5C1N6/O6 (°)	109.3(2)	109.5(2)	105.7(1)
O6/C6C1O2(°)	109.9(2)	110.7(2)	114.4(1)
O1C1N6/O6	110.3(2)	110.1(2)	111.6(1)
O2C1O5	111.1(2)	111.7(2)	108.6(1)
Tetrahedral distortion* (°)	8.4	8.1	16.7
C2 C3 C4 O3 (°)	7.6(5)	14.0(4)	179.0(2)
C2 C3 C4 O4(°)	-171.5(3)	-165.5(3)	-0.5(2)
Angle(*P*(C1,O1,O2),*P*(O5,C1,O6/N6))	90.41(9)	90.66(9)	88.23(6)
C3 O2 C1 O1 (°)	4.3(3)	0.3(2)	8.3(1)
O2 C1 O1 C2 (°)	-3.9(3)	-0.2(2)	-8.1(1)
C1 O1 C2 O3 (°)	2.0(3)	0.1(2)	4.8(2)
O1 C2 C3 O2(°)	0.7(3)	0.1(3)	0.5(2)
C2 C3 O2 C1 (°)	-3.2(3)	-0.2(3)	-5.6(1)
C1 O5 C7 C8 (°)	15.2(2)	-12.5(2)	31.6(2)
O5 C7 C8 O6/N6 (°)	-4.5(2)	5.1(2)	-24.5(2)
C7 C8 O6/N6 C1 (°)	-7.5(3)	3.7(2)	10.9(2)
C8 O6/N6 C1 O5 (°)	17.7(3)	-11.9(3)	7.4(2)
O6/N6 C1 O5 C7 (°)	-20.9(3)	15.5(3)	-24.3(2)

*calculated as $0.5 * \sum(XC1Y - 109.5)$ $with\ \ X\ and\ Y\ in\ \{O1, O2, O5, O6/N6\} and\ X \neq Y$

Table S13. Analysis of Potential Hydrogen Bonds for (2*S*,3a'*R*,4'*R*,6'*R*,7a'*S*)-**9e'** with Donors (D) and Acceptors (A). The criteria selection was d(D...A) < R(D)+R(A), d(H...A) < R(H)+R(A)-0.12 Ang and D-H...A > 100.0 Deg

Nr	Typ	Res	Donor --- H....Acceptor [ARU]	D - H	H...A	D...A	D - H...A
1	Intra	1	C7 --H7 ..O12 [ ]	1.00	2.52	3.188(3)	124
2	Intra	1	C23 --H23A ..O5 [ ]	0.99	2.45	2.849(3)	103

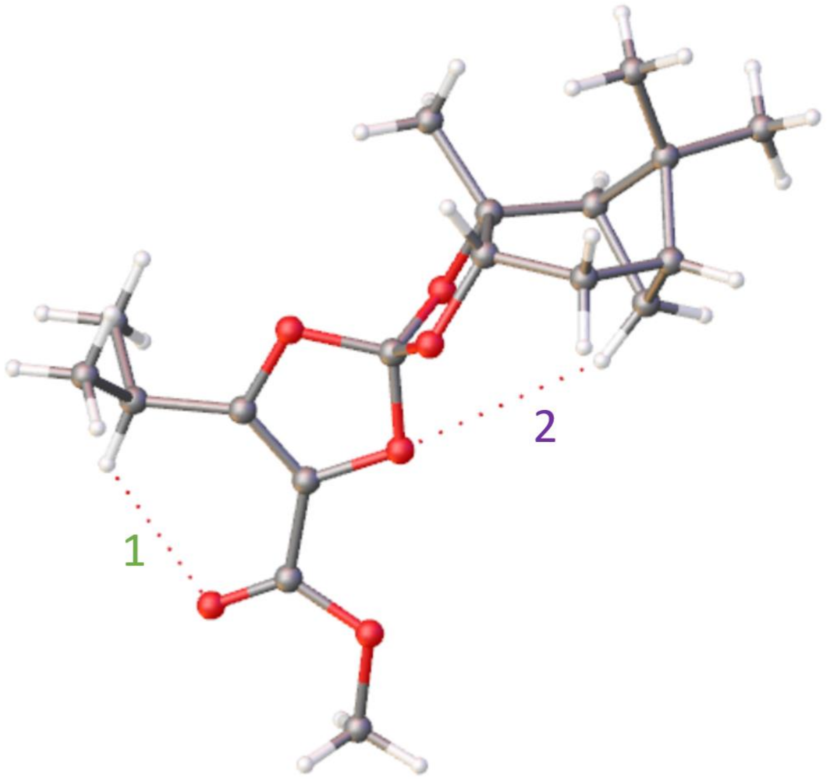

Table S14. Analysis of Potential Hydrogen Bonds for (2*R*,3a'*R*,4'*R*,6'*R*,7a'*S*)-**9e"** with donors (D) and Acceptors (A). The criteria selection was d(D...A) < R(D)+R(A), d(H...A) < R(H)+R(A)-0.12 Ang and D-H...A > 100.0 Deg

Nr	Typ	Res	Donor --- H....Acceptor	[sym op]	D - H	H...A	D...A	D - H...A
1	Intra	1	C7 --H7 ..O12	[ ]	1.00	2.52	3.204(3)	125
2		1	C14 --H14C ..O5	[-x,y,z]	0.98	2.55	3.372(3)	142
3	Intra	1	C20 --H20A ..O4	[ ]	0.99	2.55	3.181(3)	121
4	Intra	1	C20 --H20A ..O5	[ ]	0.99	2.55	2.913(3)	101

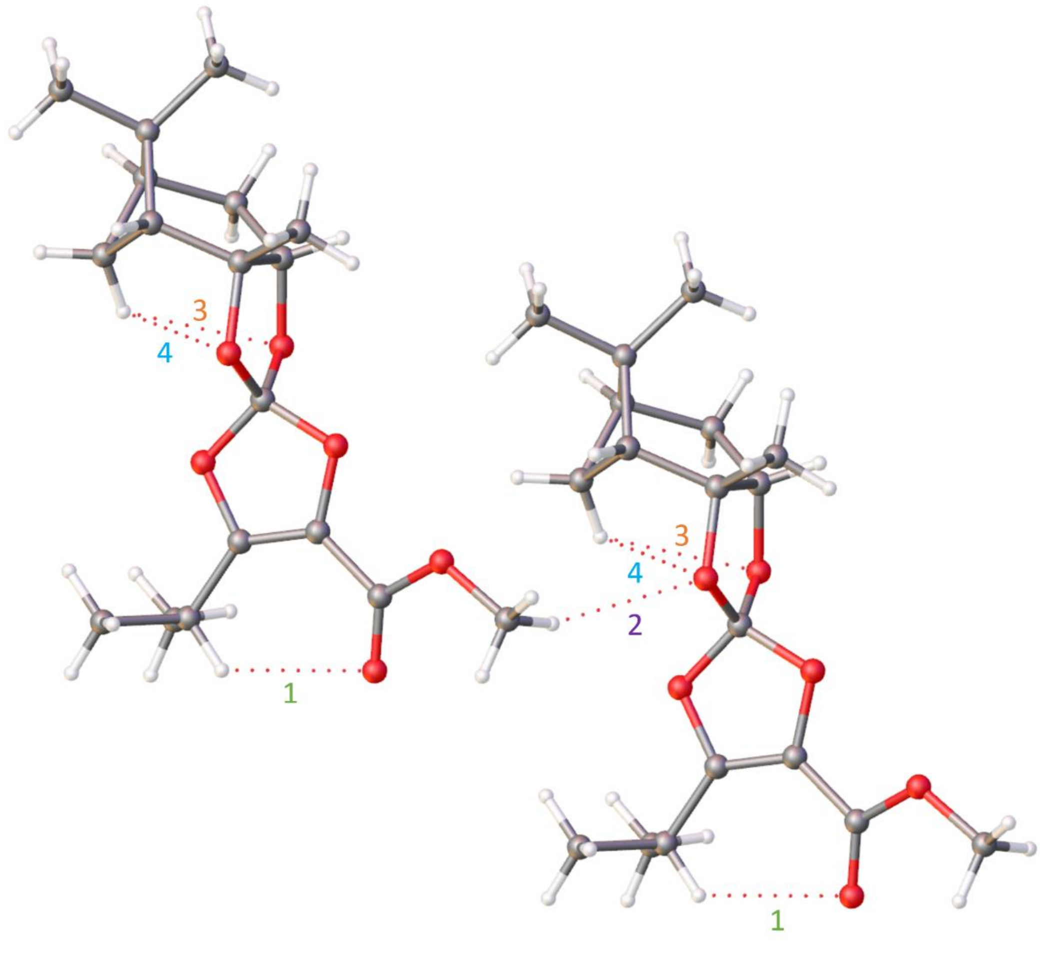

Table S15. Analysis of Potential Hydrogen Bonds for (5*S*,8*S*)-**10i'** with donors (D) and Acceptors (A). The criteria selection was d(D...A) < R(D)+R(A), d(H...A) < R(H)+R(A)-0.12 Ang and D-H...A > 100.0 Deg

Nr	Typ	Res	Donor --- H....Acceptor [ARU]	D - H	H...A	D...A	D - H...A
1		1	C1 --H1C ..O3[1-x,y-0.5,1.5-z]	0.98	2.54	3.468(2)	158
2	Intra	1	C7 --H7B ..O2 [ ]	0.98	2.38	3.038(2)	124
3	Intra	1	C20 --H20A ..O16 [ ]	0.98	2.49	3.006(2)	113
4		1	C20 --H20B ..O16[-x,0.5+y,1.5-z]	0.98	2.58	3.426(2)	145
5	Intra	1	C21 --H21C ..O16 [ ]	0.98	2.48	3.070(2)	118
6	Intra	1	C22 --H22 ..O17 [ ]	1.00	2.55	3.100(2)	114
7		1	C24 --H24C ..O8[0.5+x,0.5-y,1-z]	0.98	2.51	3.460(2)	162

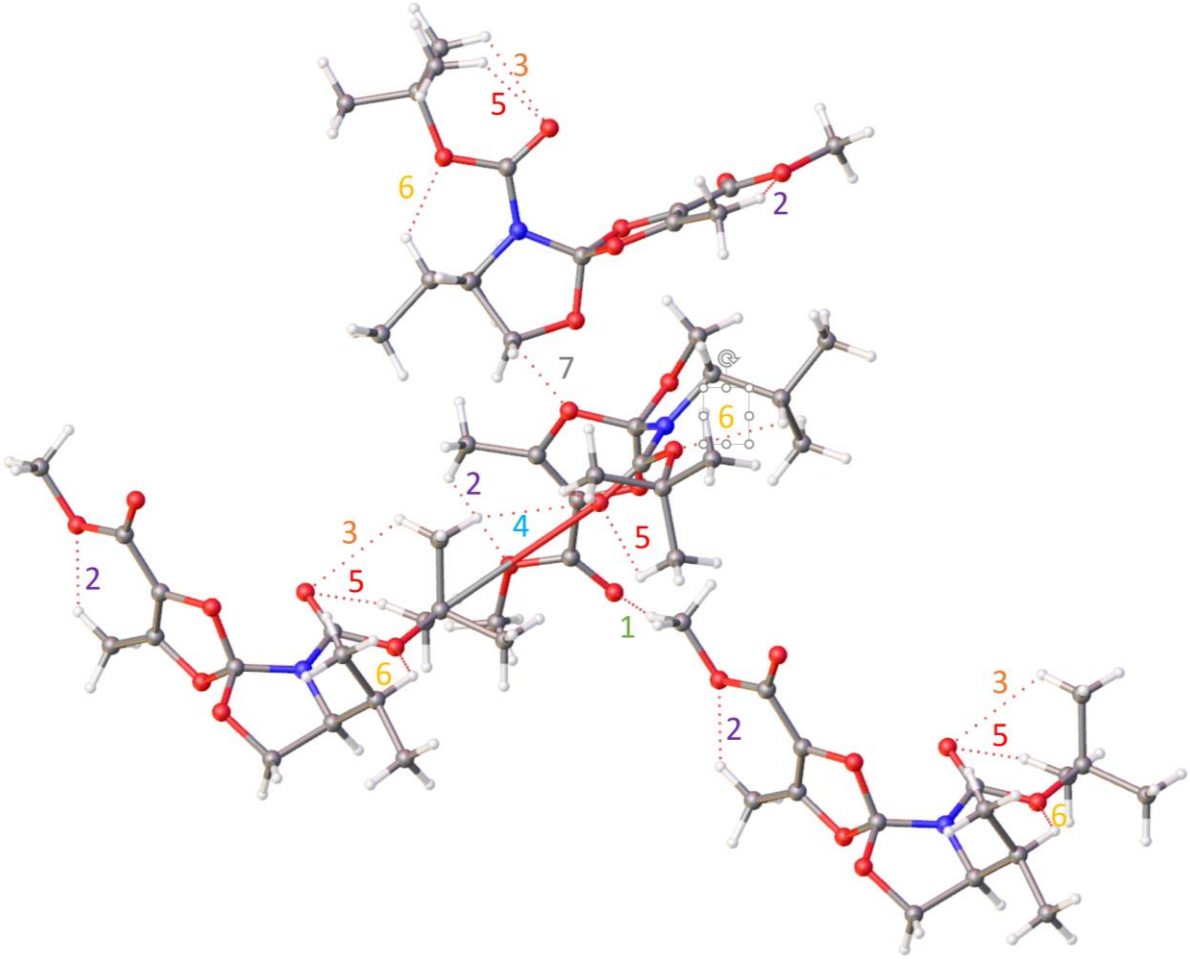

Table S16. Ring puckering parameters on the ring C1-O6-C8-C7-O5. The parameters are calculated according to Cramer et Pope.[15] Q gives the amplitude of the deformation and Phi the geometry. On the closest geometry scheme, the full circle represents an atom above and the empty one below the plane of the other three atoms. The bond between the twisted atoms is drawn in violet in the view of the ring.

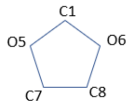

	9e'	*9e''*	*10i'*
**Q(2)**	**0.172(2)**	**0.128(2)**	**0.2796(15)**
**Phi(2)**	**164.6 (8)**	**336.6(10)**	**120.6(3)**
**Closest geometry**	Twisted on O5-C1 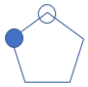	Twisted on O5-C1 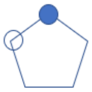	Twisted on C7-O5 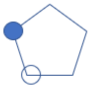
**View of the ring**	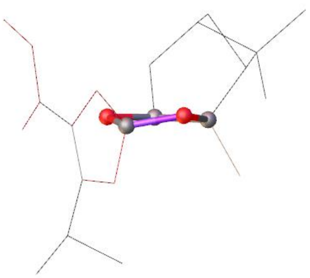	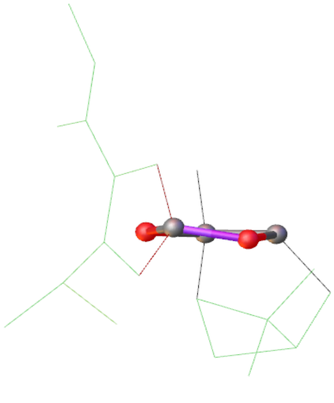	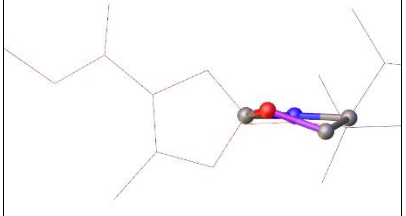

9. ^{1}H and ^{13}C NMR spectra

^{1}H NMR (500 MHz, $CDCl_3$) Methyl 3,7,7-trimethyl-1,4,6,9-tetraoxaspiro[4.4]non-2-ene-2-carboxylate **9a**

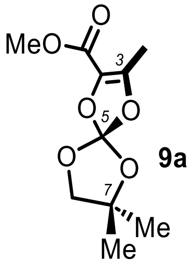

9a

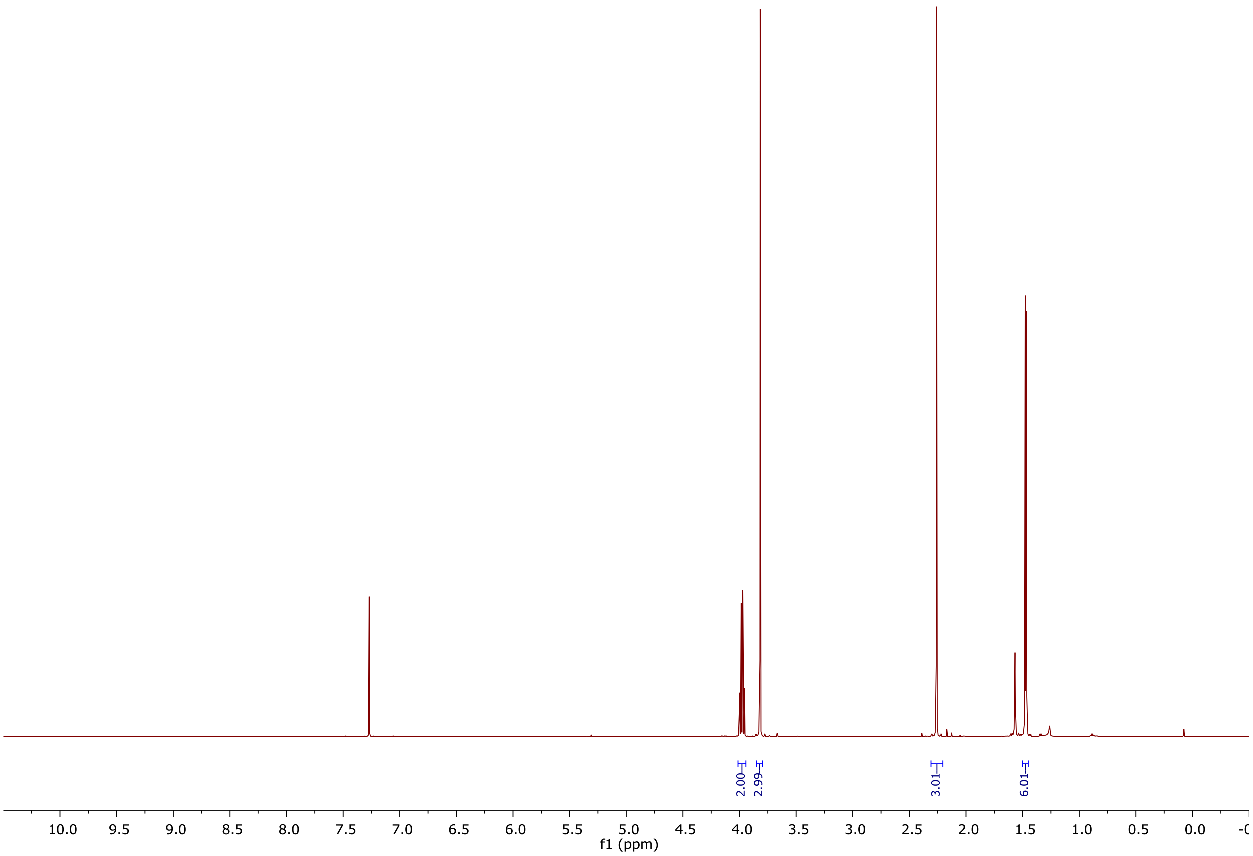

^{13}C NMR (126 MHz, $CDCl_3$) Methyl 3,7,7-trimethyl-1,4,6,9-tetraoxaspiro[4.4]non-2-ene-2-carboxylate **9a**

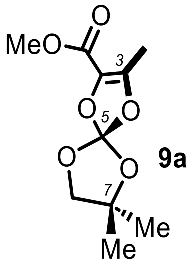

9a

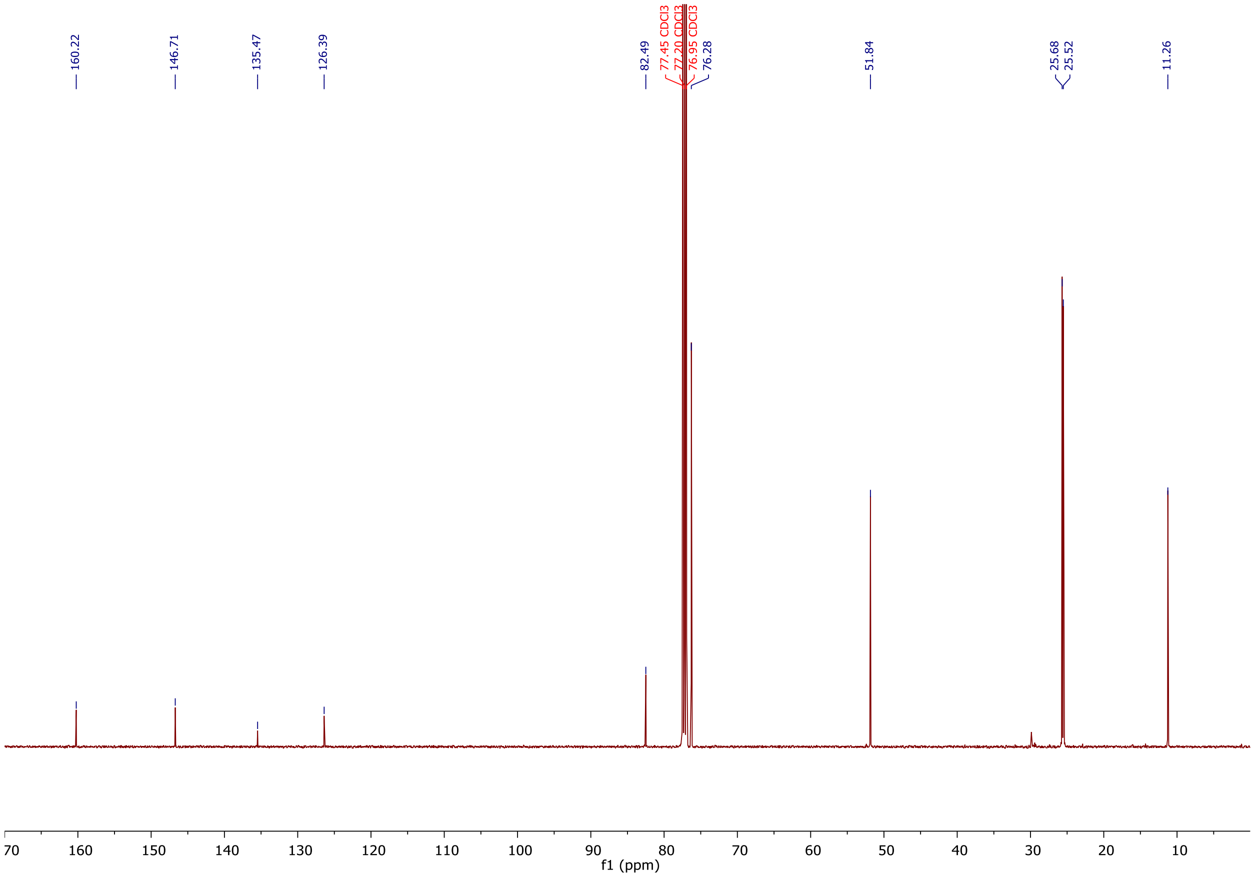

^{1}H NMR (500 MHz, $CDCl_3$) 3-isopropyl-7,7-dimethyl-1,4,6,9-tetraoxaspiro [4.4]non-2-ene-2-carboxylate **9b**

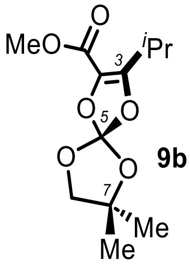

9b

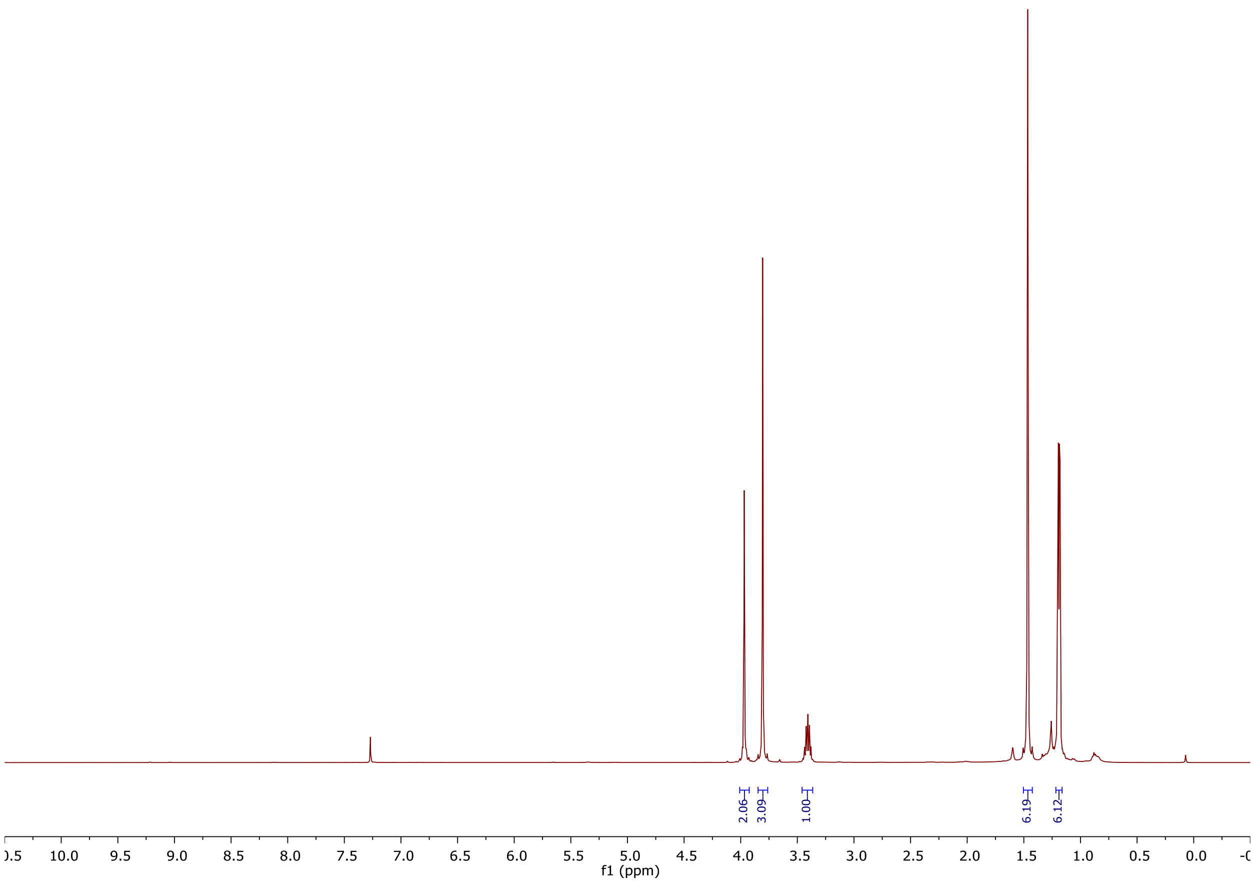

^{13}C NMR (126 MHz, $CDCl_3$) 3-isopropyl-7,7-dimethyl-1,4,6,9-tetraoxaspiro [4.4]non-2-ene-2-carboxylate **9b**

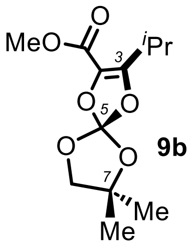

9b

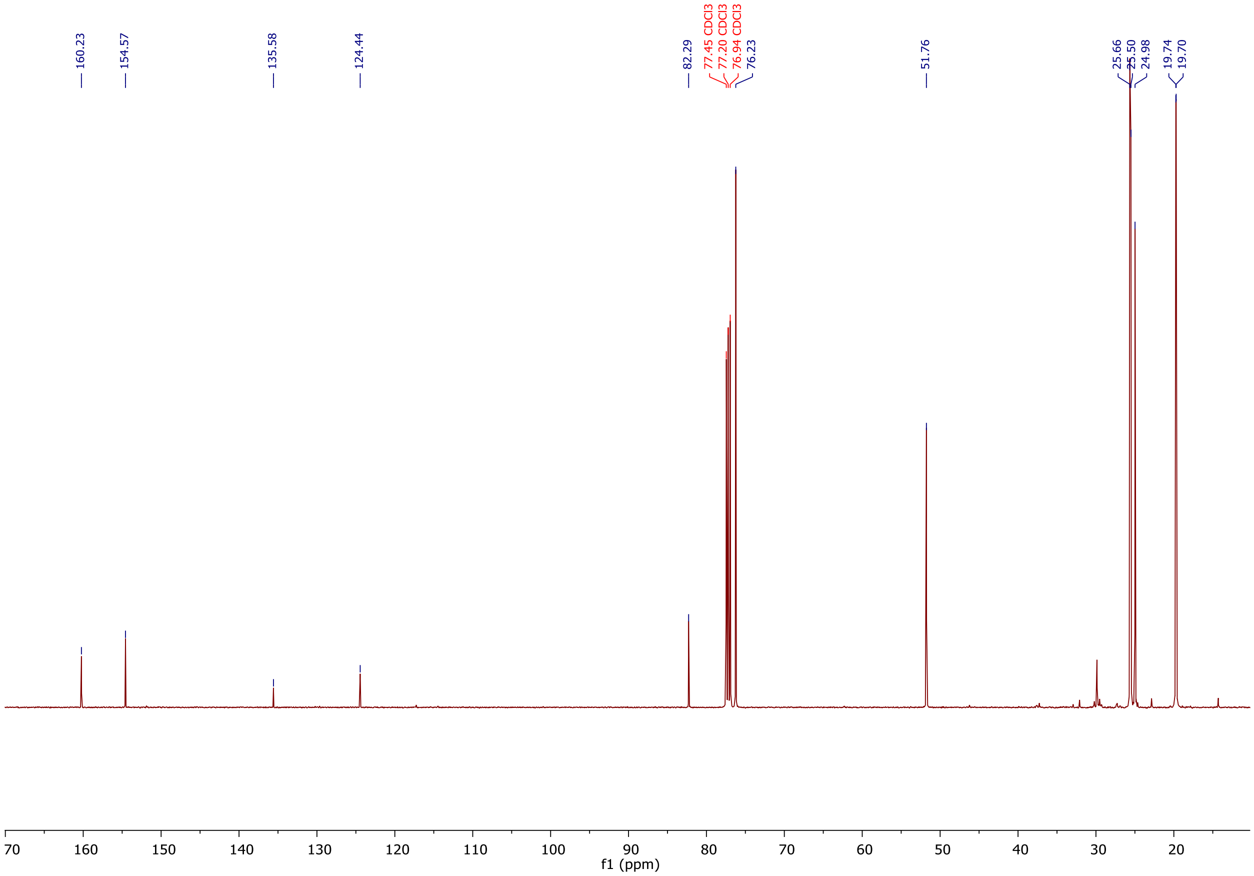

^{1}H NMR (500 MHz, $CDCl_3$) Methyl 3-methyl-7,7-diphenyl-1,4,6,9-tetraoxaspiro[4.4]non-2-ene-2-carboxylate **9c**

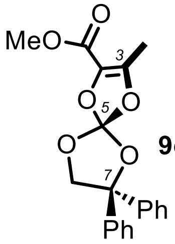

9c

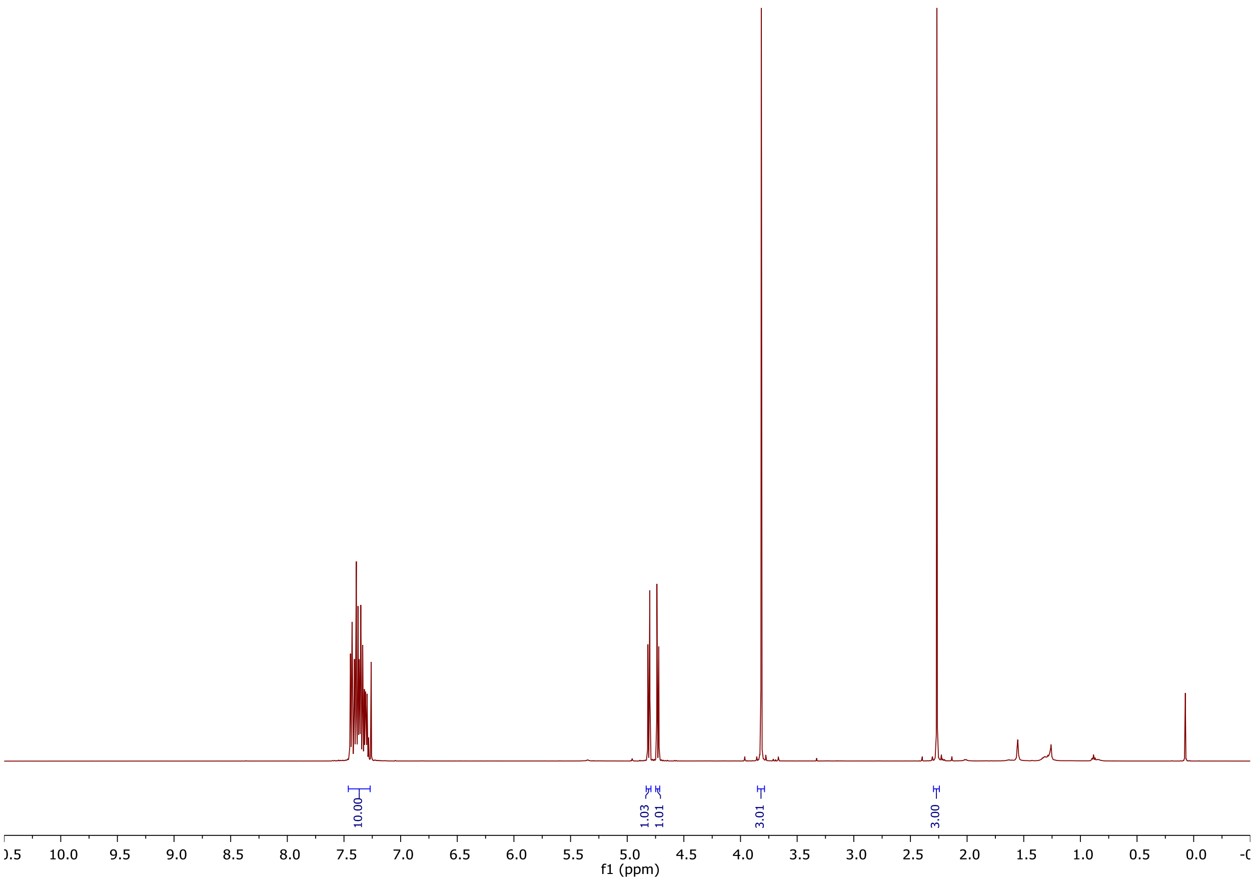

^{13}C NMR (126 MHz, $CDCl_3$) Methyl 3-methyl-7,7-diphenyl-1,4,6,9-tetraoxaspiro[4.4]non-2-ene-2-carboxylate **9c**

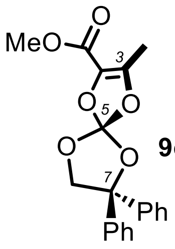

9c

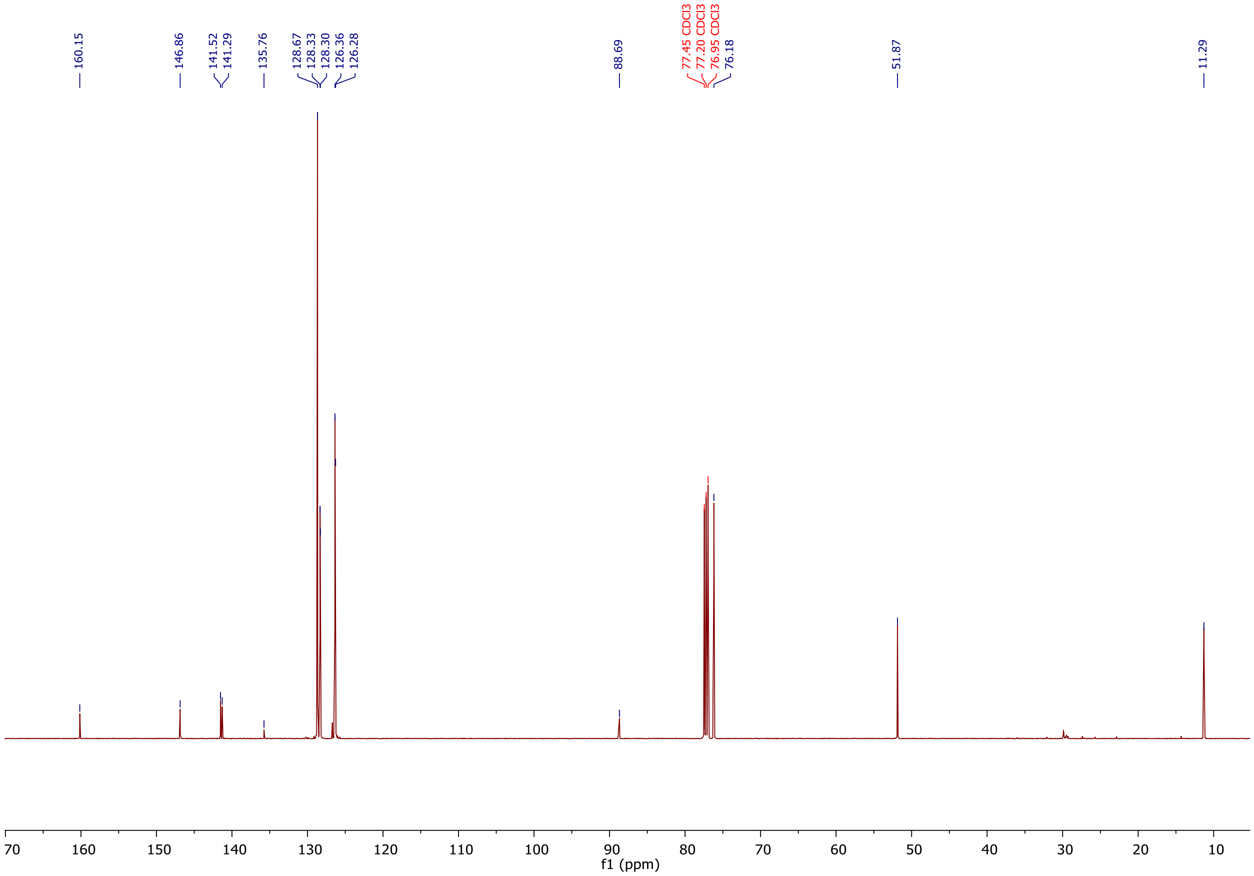

^{1}H NMR (500 MHz, $CDCl_3$) Methyl 3-isopropyl-7,7-diphenyl-1,4,6,9-tetraoxaspiro[4.4]non-2-ene-2-carboxylate **9d**

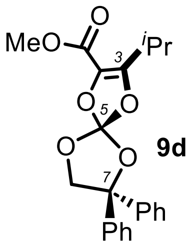

9d

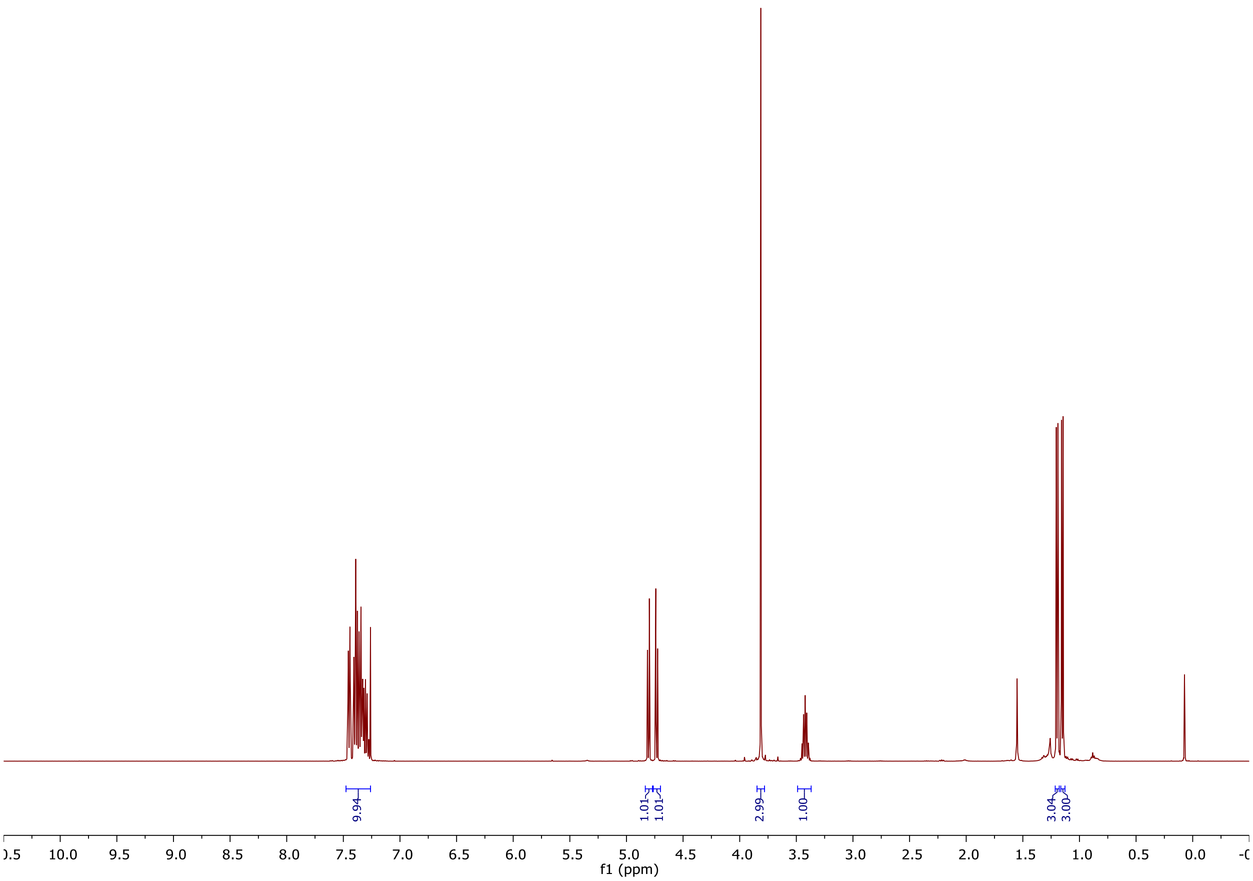

^{13}C NMR (126 MHz, $CDCl_3$) Methyl 3-isopropyl-7,7-diphenyl-1,4,6,9-tetraoxaspiro[4.4]non-2-ene-2-carboxylate **9d**

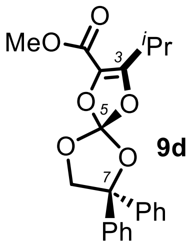

9d

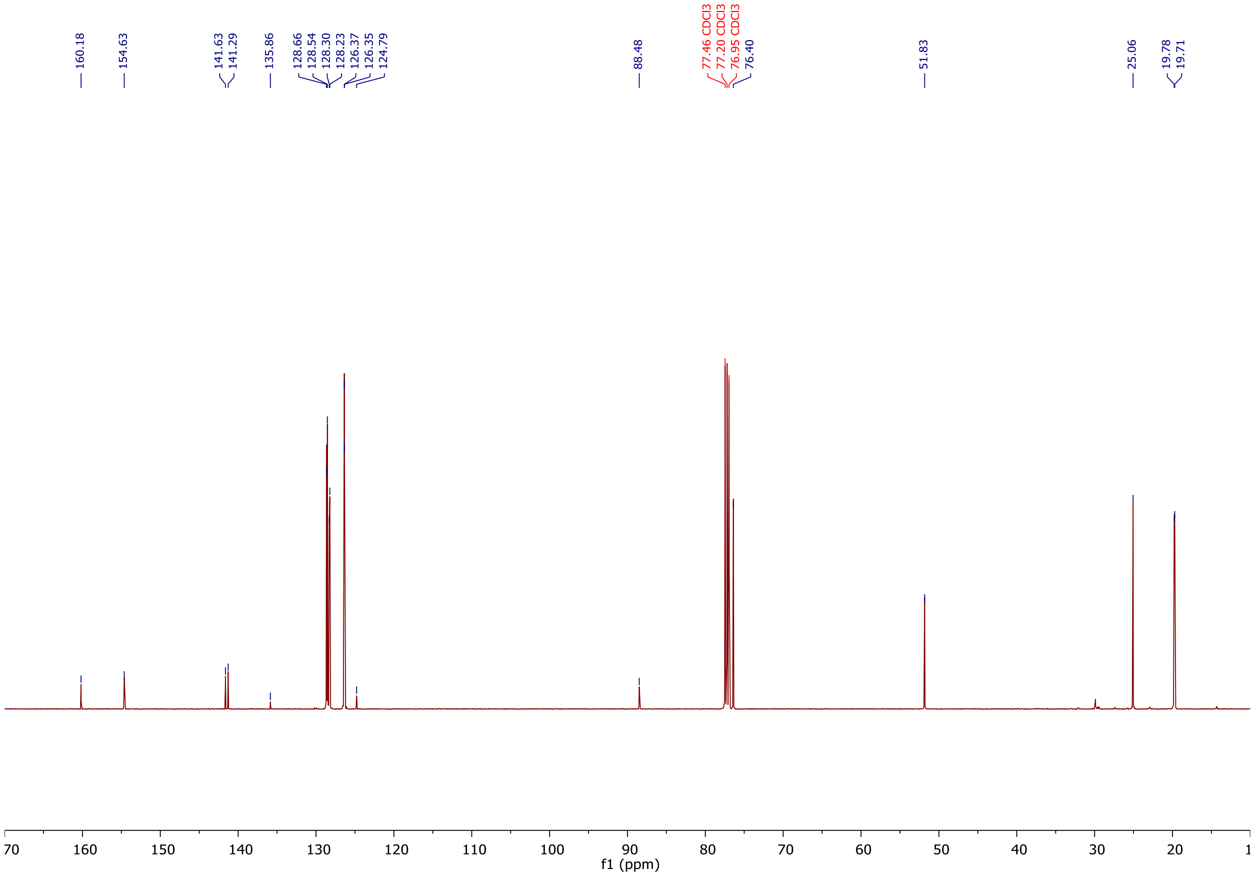

^{1}H NMR (500 MHz, $CDCl_3$) (2*S*/2*R*,3a'*R*,4'*R*,6'*R*,7a'*S*)-5-isopropyl-3a',5',5'-trimethyl-3a',4',5',6',7',7a'-hexahydrospiro[[1,3]dioxole-2,2'-[4,6]methanobenzo[d][1,3]dioxole]-4-carboxylate **9e**

Diastereoselectivity measured on the crude mixture

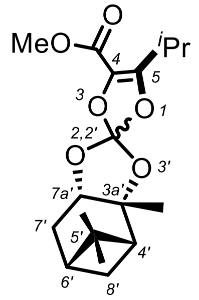

9e' + 9e"
(1.2:1 d.r)

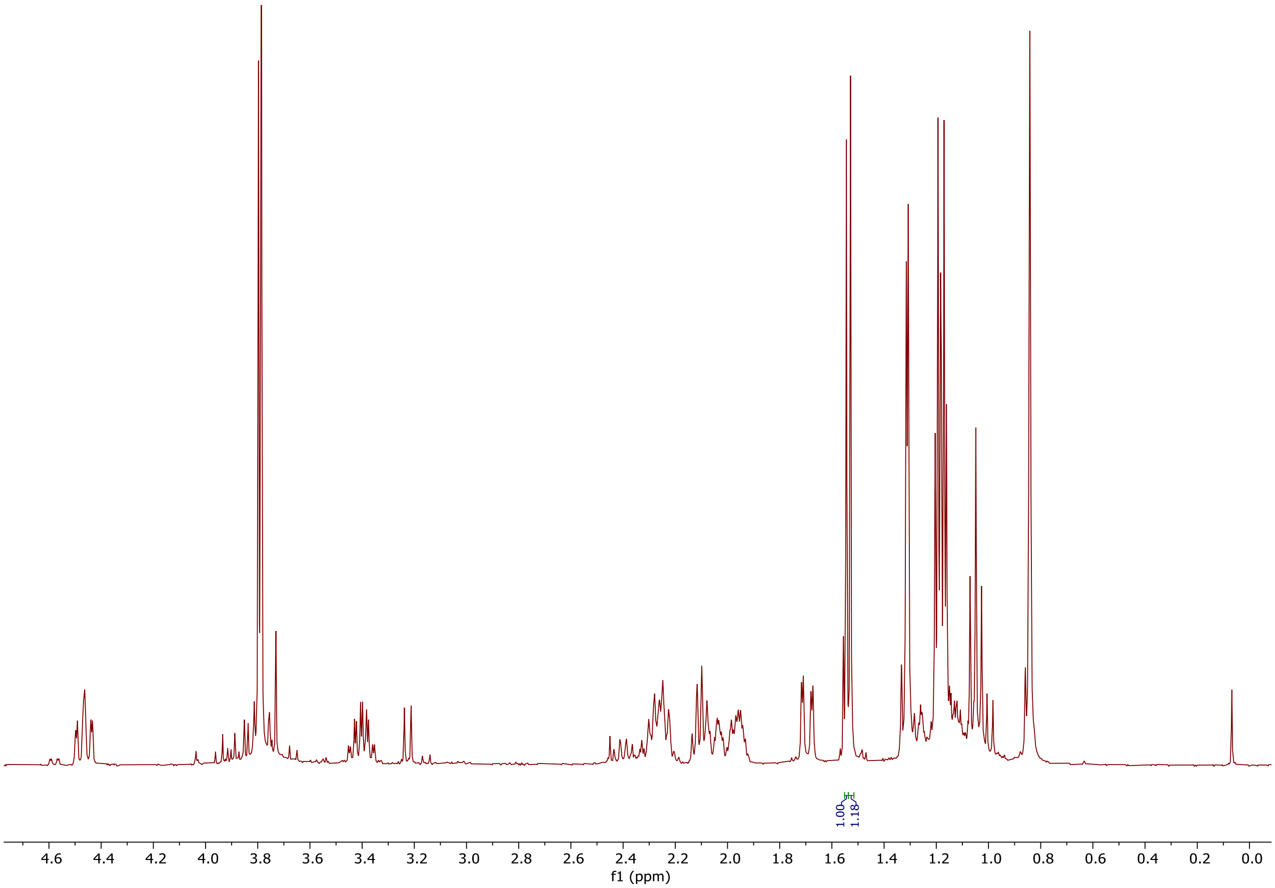

^{1}H NMR (500 MHz, CDCl3) (2*S*,3a'*R*,4'*R*,6'*R*,7a'*S*)-5-isopropyl-3a',5',5'-trimethyl-3a',4',5',6',7',7a'-hexahydrospiro[[1,3]dioxole-2,2'-[4,6]methanobenzo[d][1,3]dioxole]-4-carboxylate **9e'**

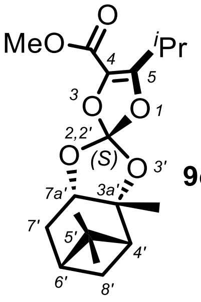

9e'

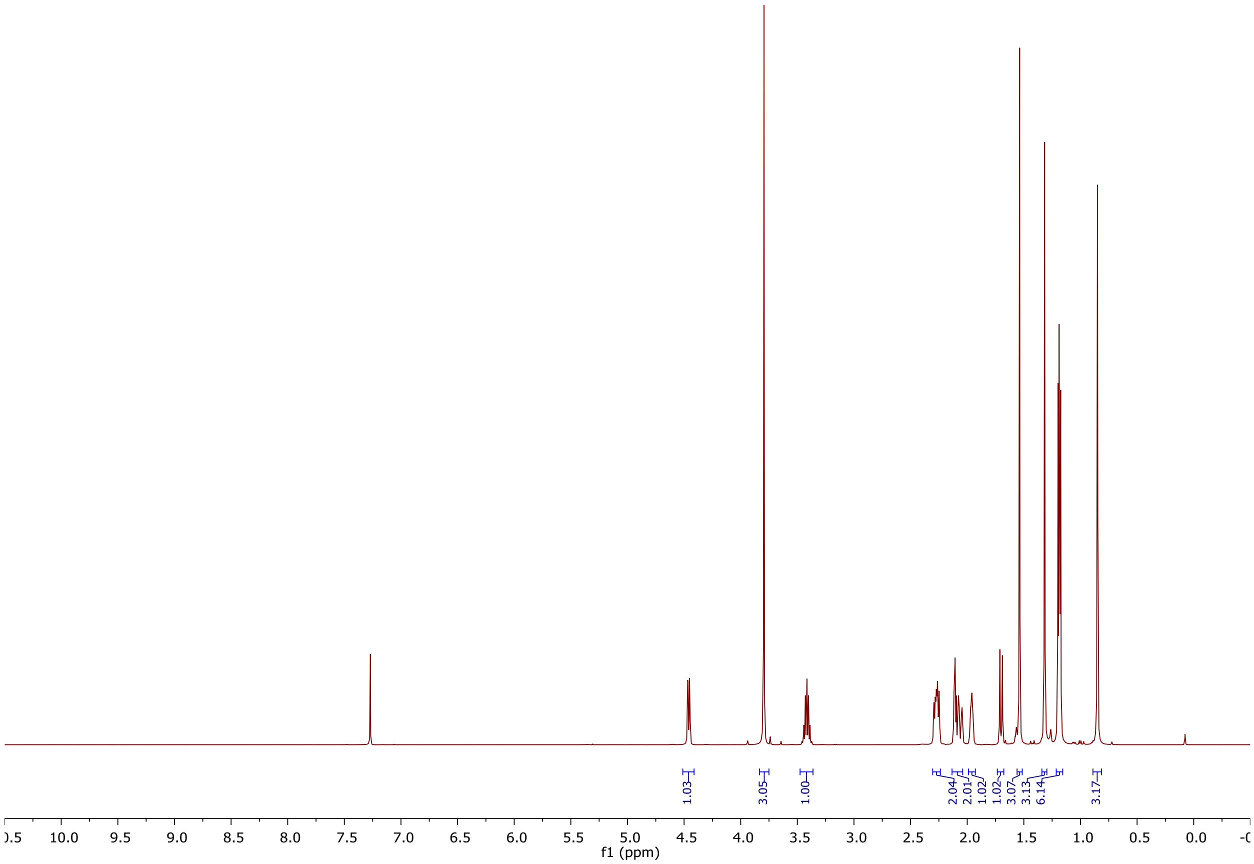

^{13}C NMR (126 MHz, $CDCl_3$) (2*S*,3a'*R*,4'*R*,6'*R*,7a'*S*)-5-isopropyl-3a',5',5'-trimethyl-3a',4',5',6',7',7a'-hexahydrospiro[[1,3]dioxole-2,2'-[4,6]methanobenzo[d][1,3]dioxole]-4-carboxylate **9e'**

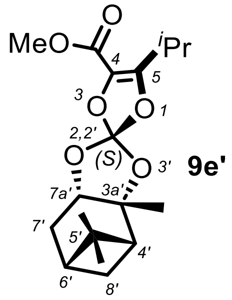

9e'

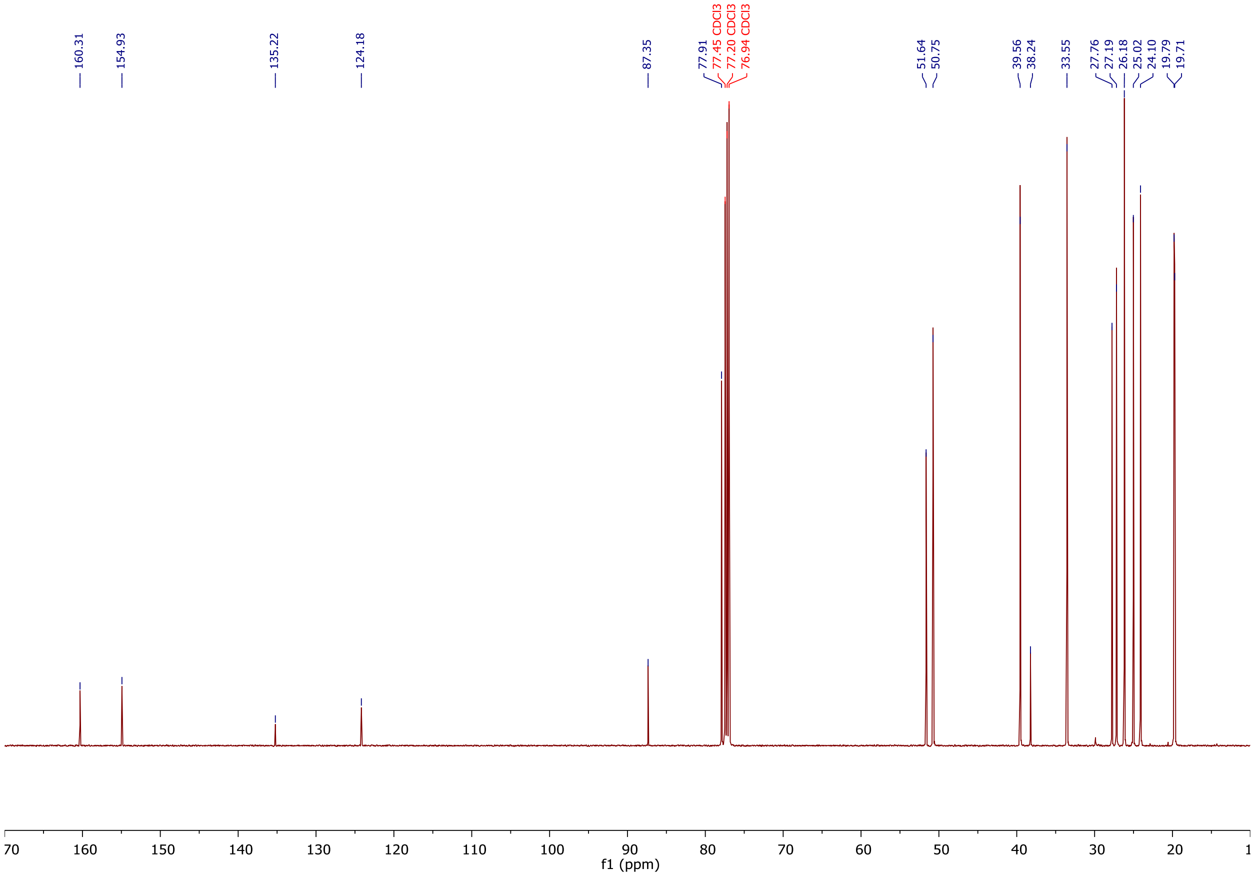

^{1}H NMR (500 MHz, $CDCl_3$) (2*R*,3a'*R*,4'*R*,6'*R*,7a'*S*)-5-isopropyl-3a',5',5'-trimethyl-3a',4',5',6',7',7a'-hexahydrospiro[[1,3]dioxole-2,2'-[4,6]methanobenzo[d][1,3]dioxole]-4-carboxylate **9e”**

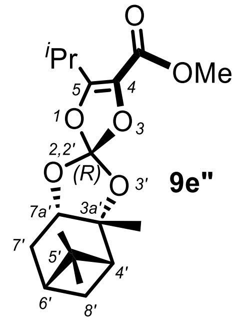

9e"

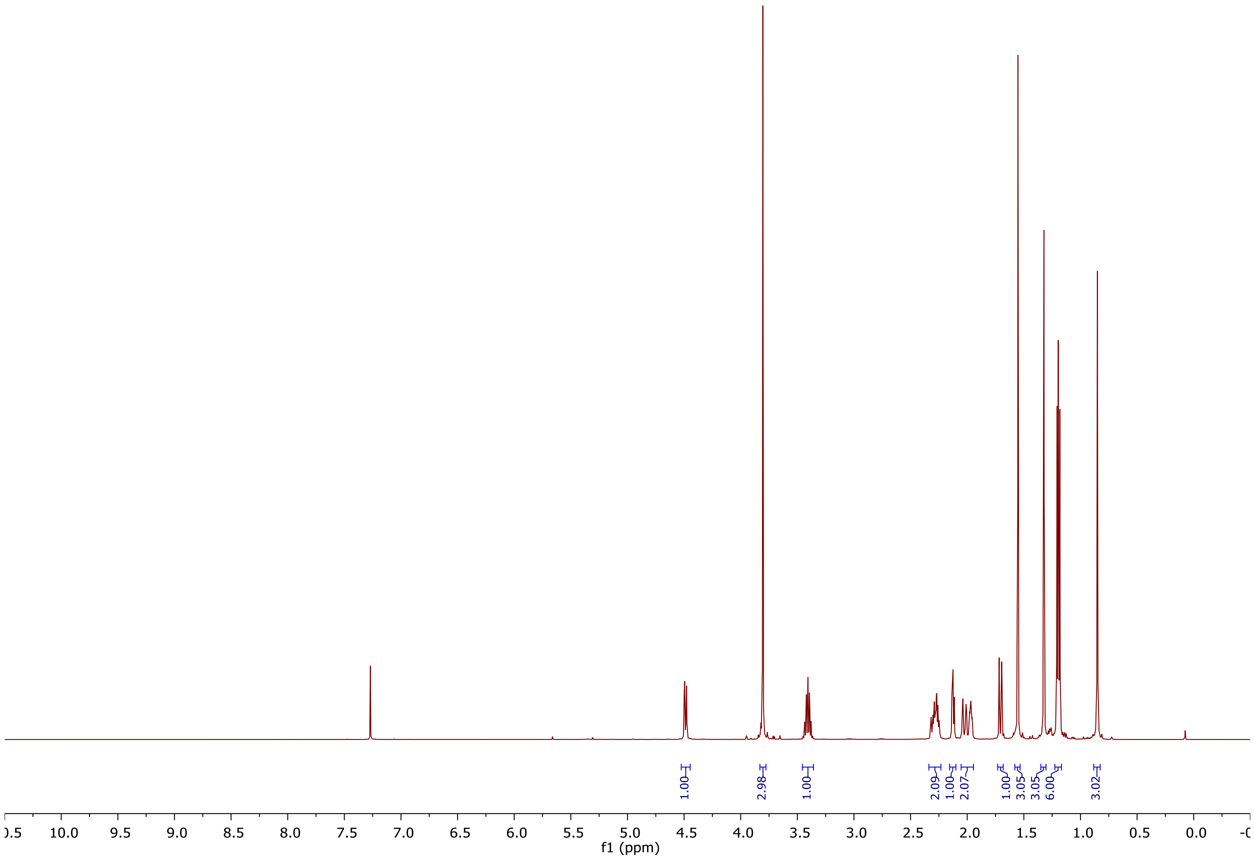

^{13}C NMR (126 MHz, $CDCl_3$) (2*R*,3a'*R*,4'*R*,6'*R*,7a'*S*)-5-isopropyl-3a',5',5'-trimethyl-3a',4',5',6',7',7a'-hexahydrospiro[[1,3]dioxole-2,2'-[4,6]methanobenzo[d][1,3]dioxole]-4-carboxylate **9e”**

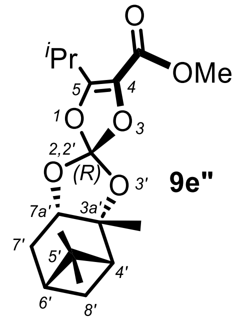

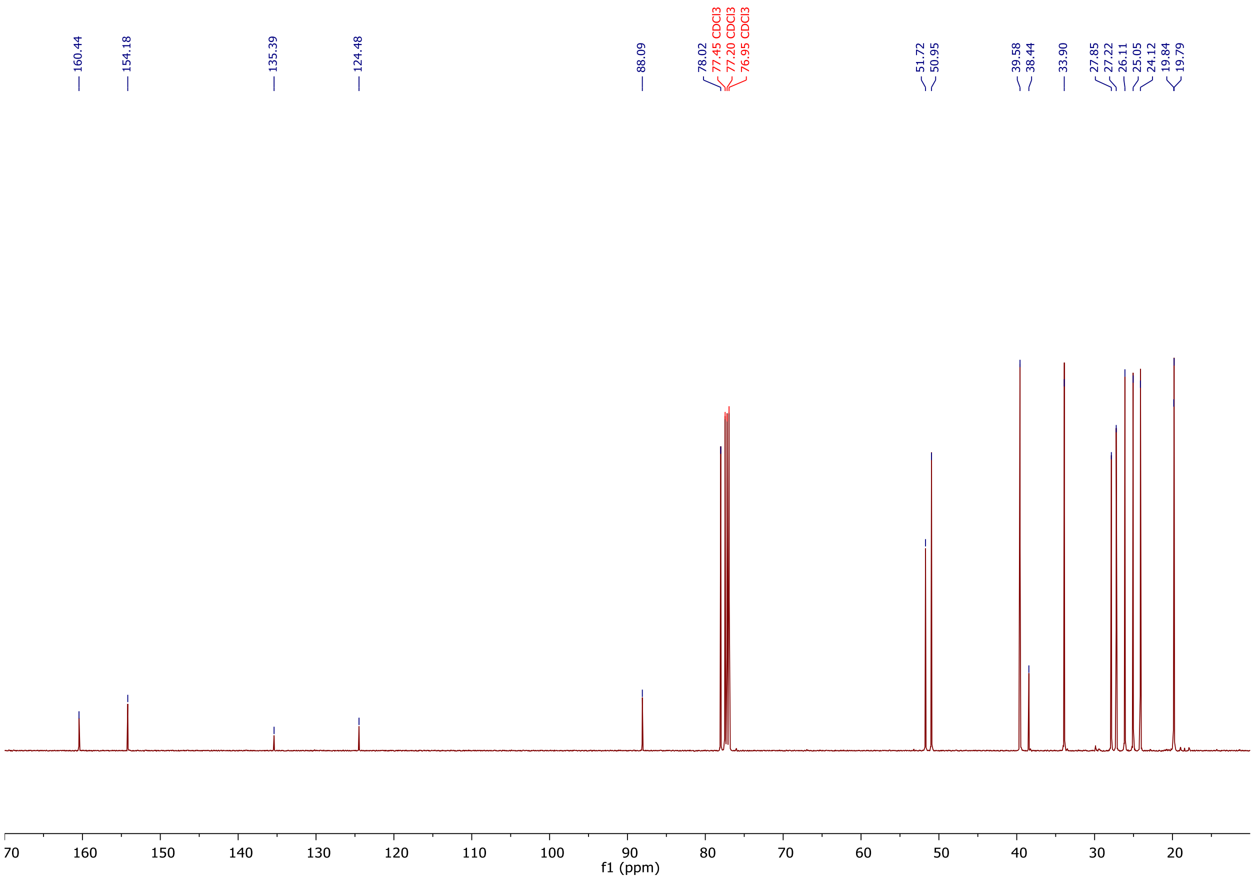

^{1}H NMR (500 MHz, $CDCl_3$) *tert*-Butyl 2-oxooxazolidine-3-carboxylate **13a**

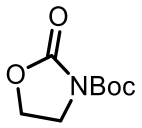

13a

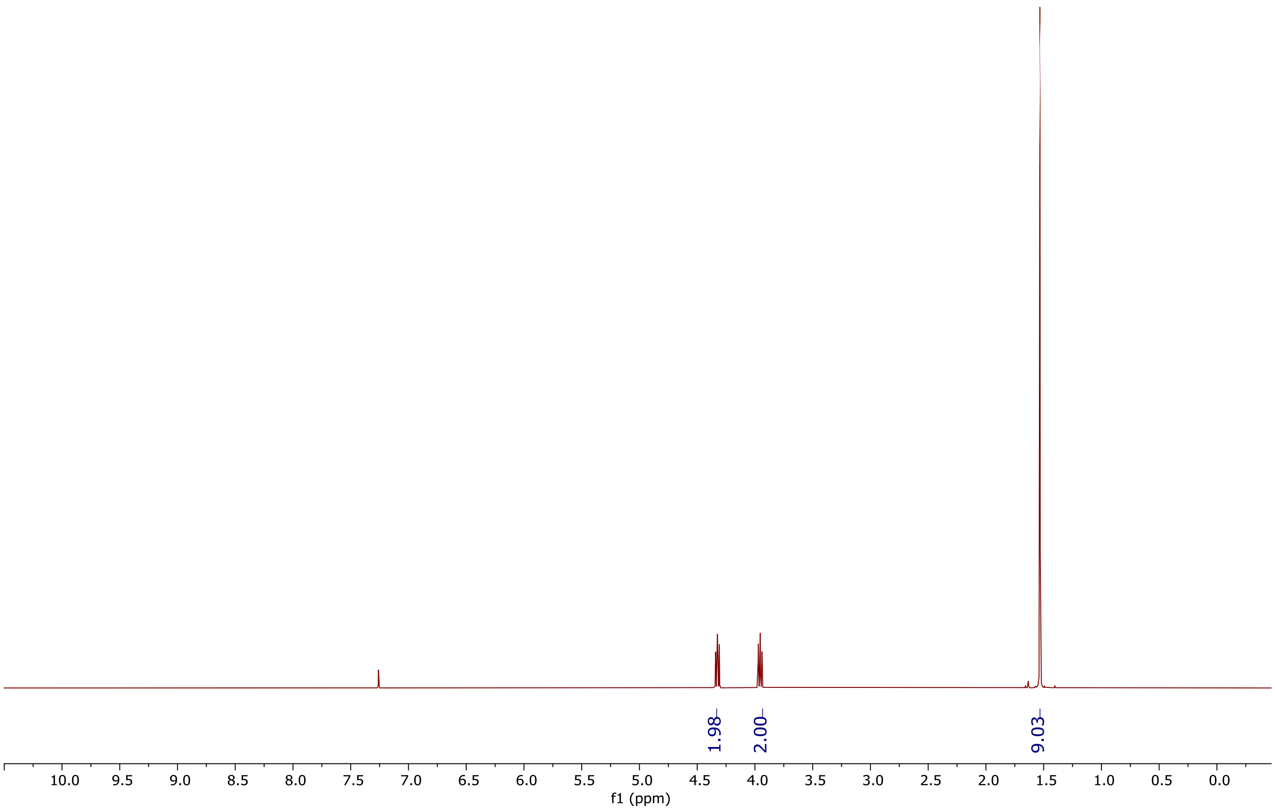

^{13}C NMR (126 MHz, $CDCl_3$) *tert*-Butyl 2-oxooxazolidine-3-carboxylate **13a**

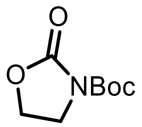

13a

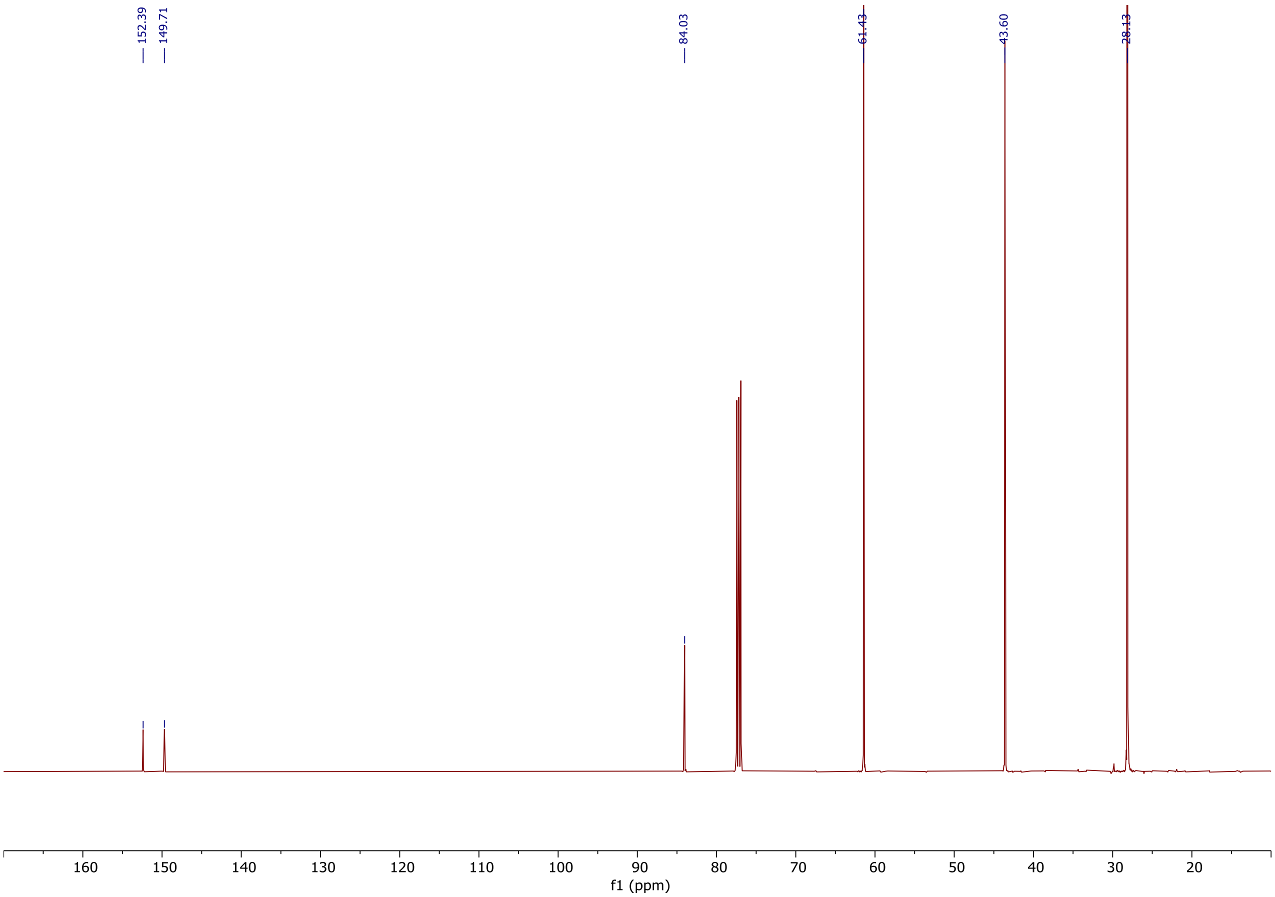

^{1}H NMR (500 MHz, $CDCl_3$) 2,2,2-trichloroethyl 2-oxooxazolidine-3-carboxylate **13b**

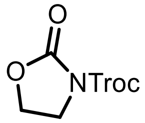

13b

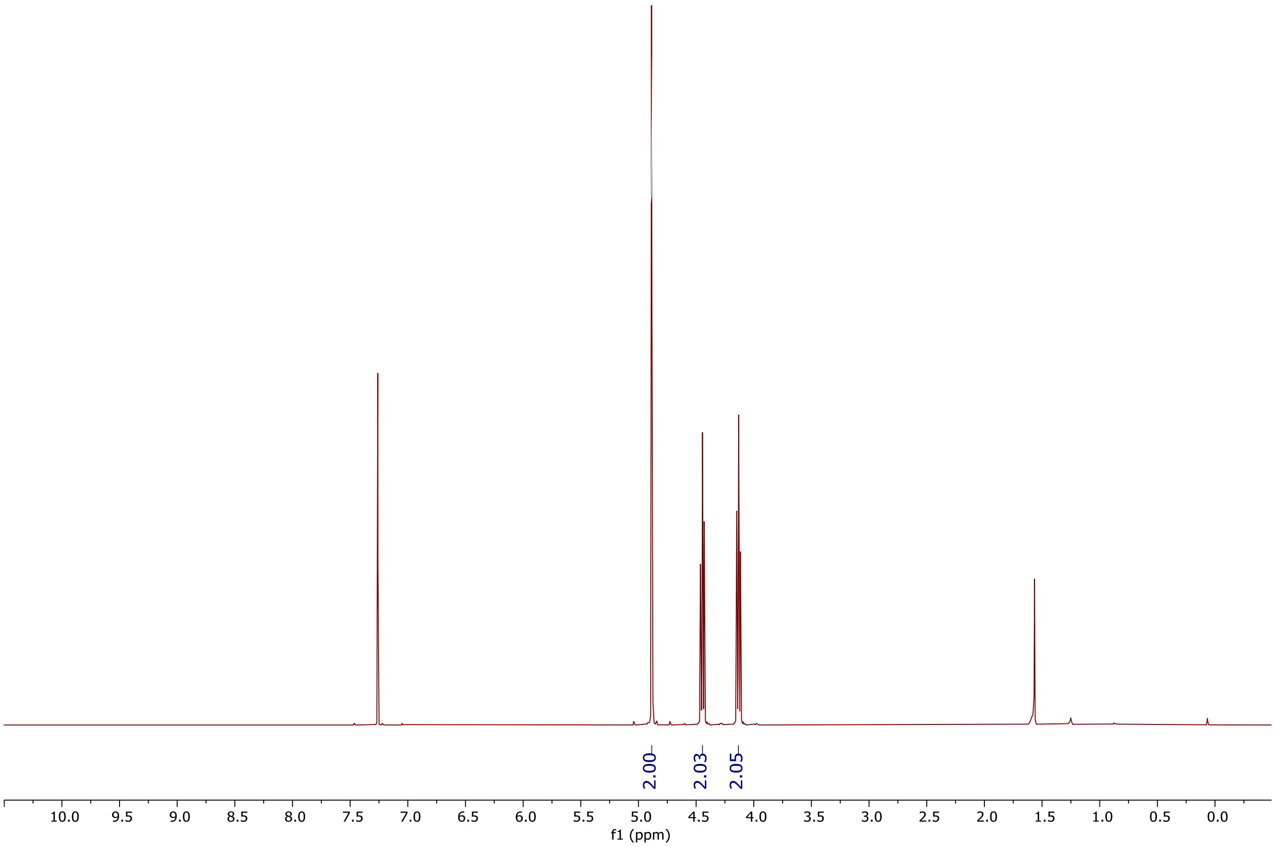

^{13}C NMR (75 MHz, $CDCl_3$) 2,2,2-trichloroethyl 2-oxooxazolidine-3-carboxylate **13b**

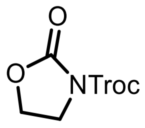

13b

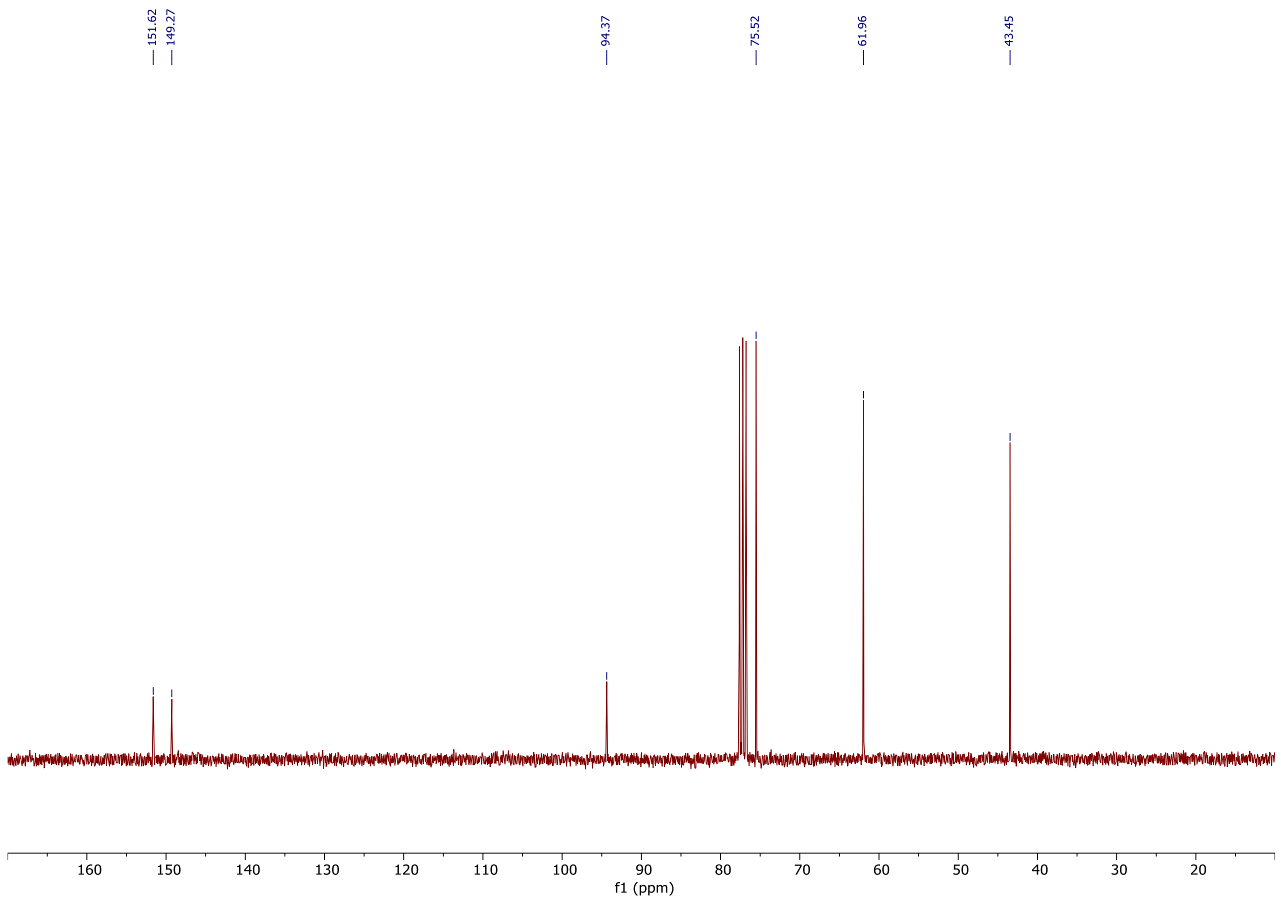

^{1}H NMR (500 MHz, $CDCl_3$) allyl 2-oxooxazolidine-3-carboxylate
13c

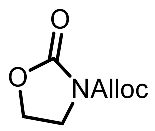

13c

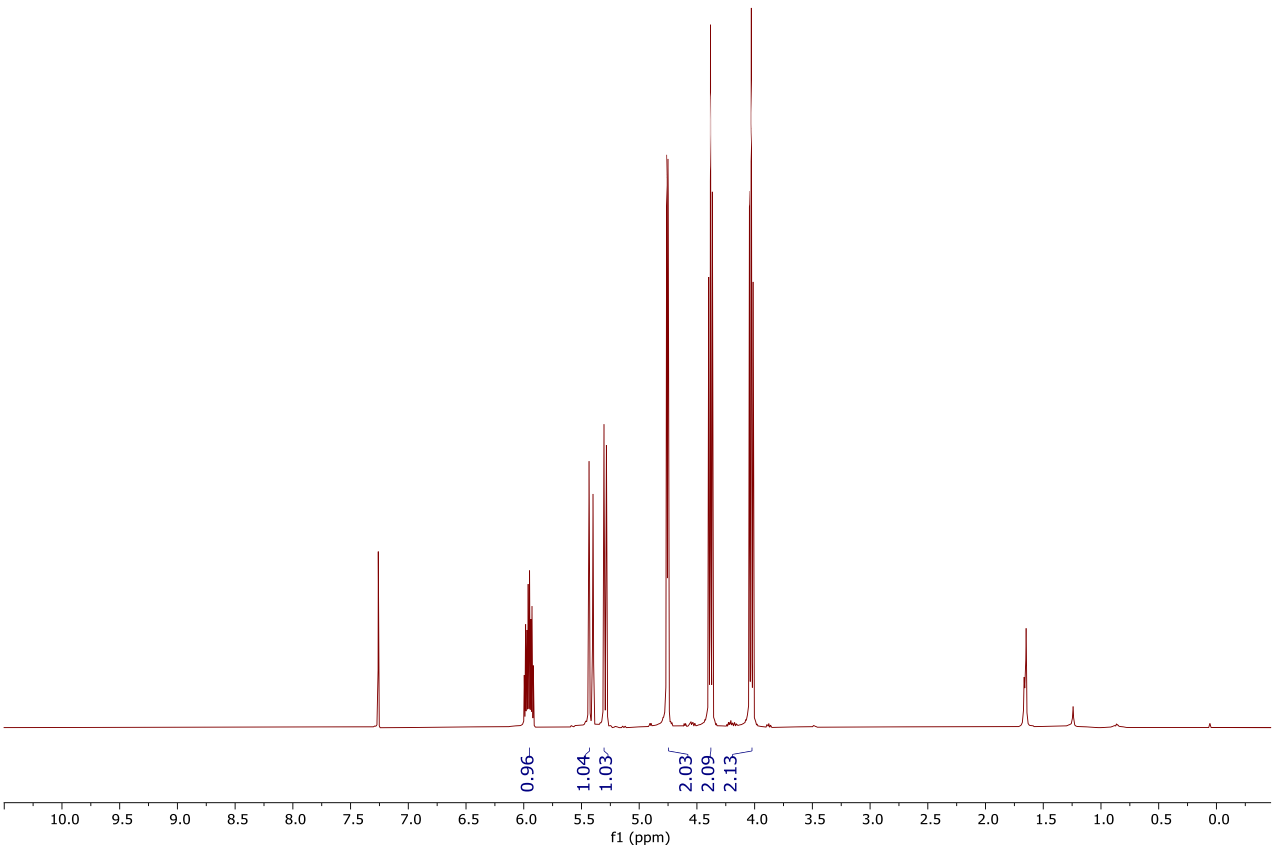

^{13}C NMR (500 MHz, $CDCl_3$) allyl 2-oxooxazolidine-3-carboxylate **13c**

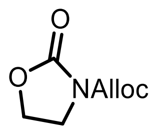

13c

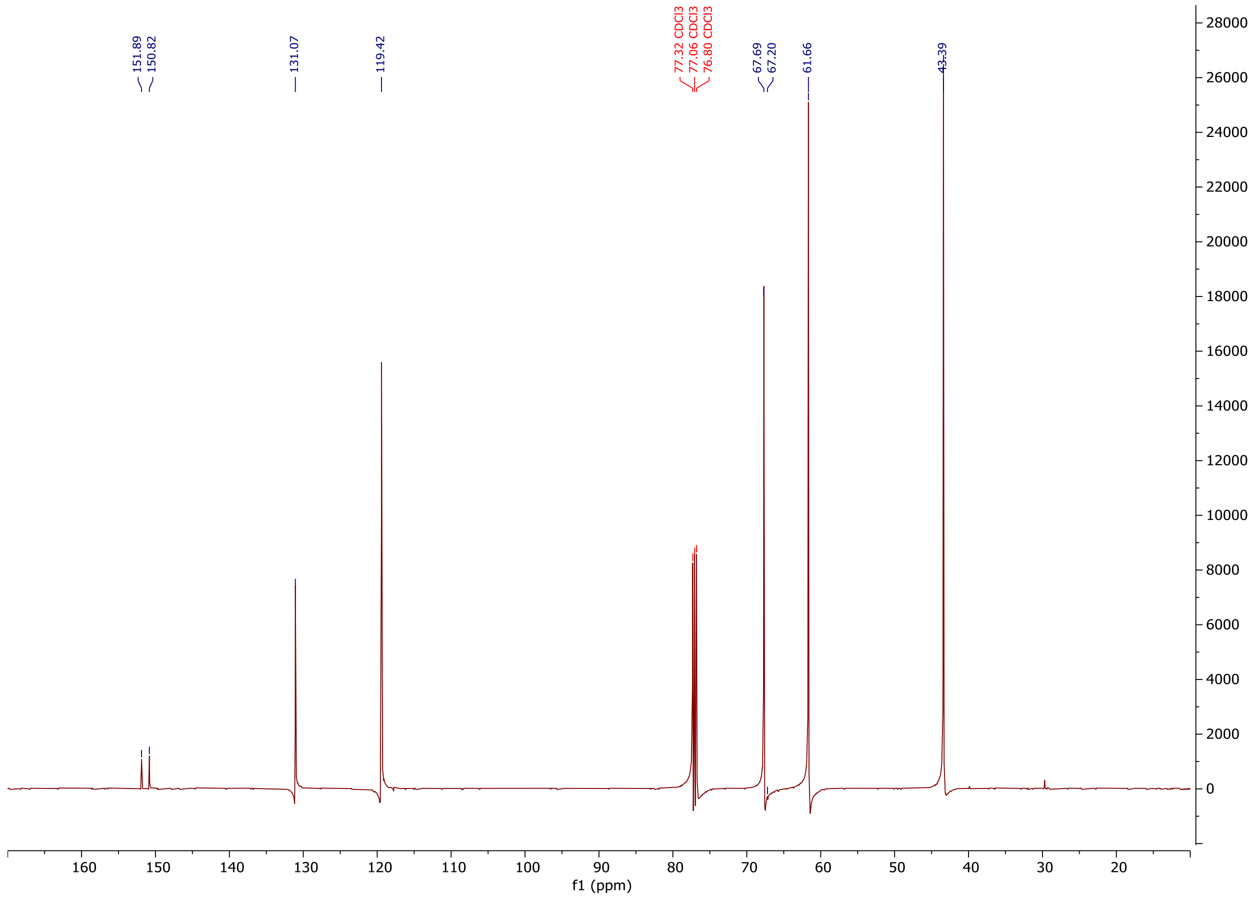

^{1}H NMR (500 MHz, $CDCl_3$) *tert*-Butyl *(S)*-4-isopropyl-2-oxooxazolidine-3-carboxylate **13d**

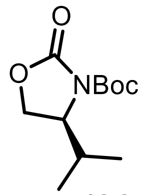

13d

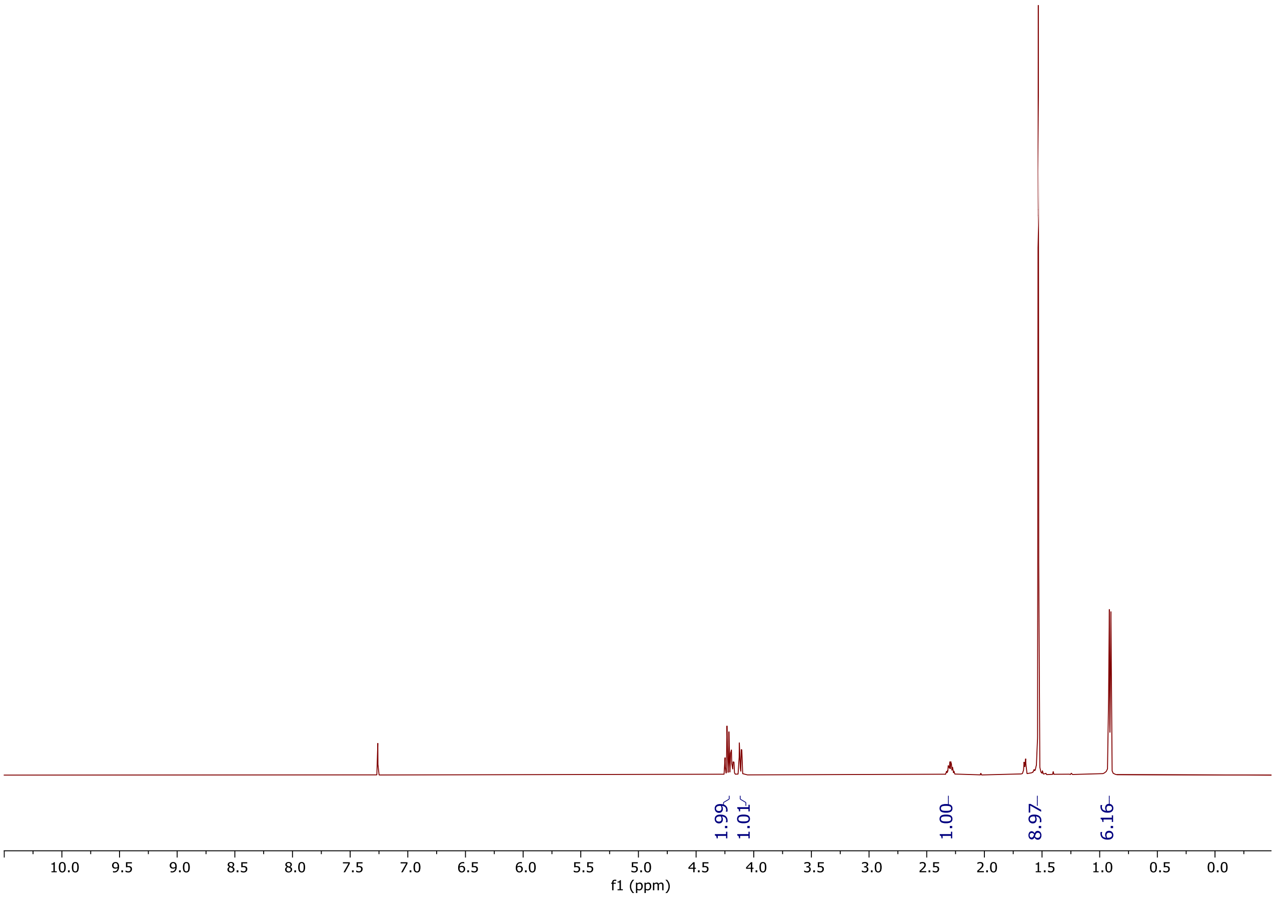

^{13}C NMR (126 MHz, $CDCl_3$) *tert*-Butyl *(S)*-4-isopropyl-2-oxooxazolidine-3-carboxylate **13d**

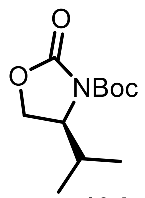

13d

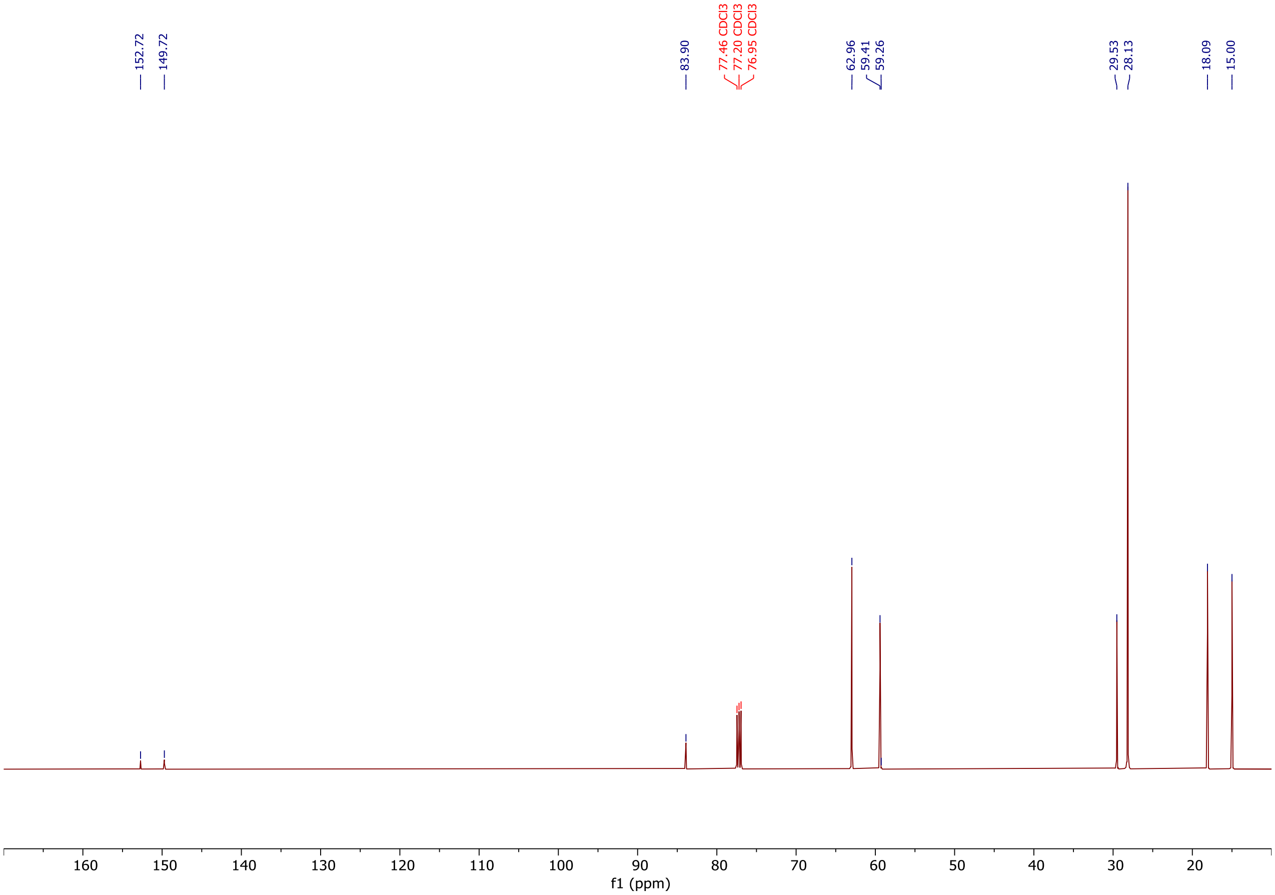

^{1}H NMR (500 MHz, $CDCl_3$) 9-(*tert*-Butyl) 2-methyl-3-methyl-1,4,6-trioxa-9-azaspiro[4.4]non-2-ene-2,9-dicarboxylate **10a**

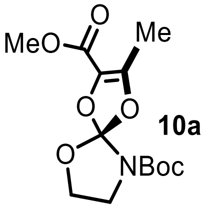

10a

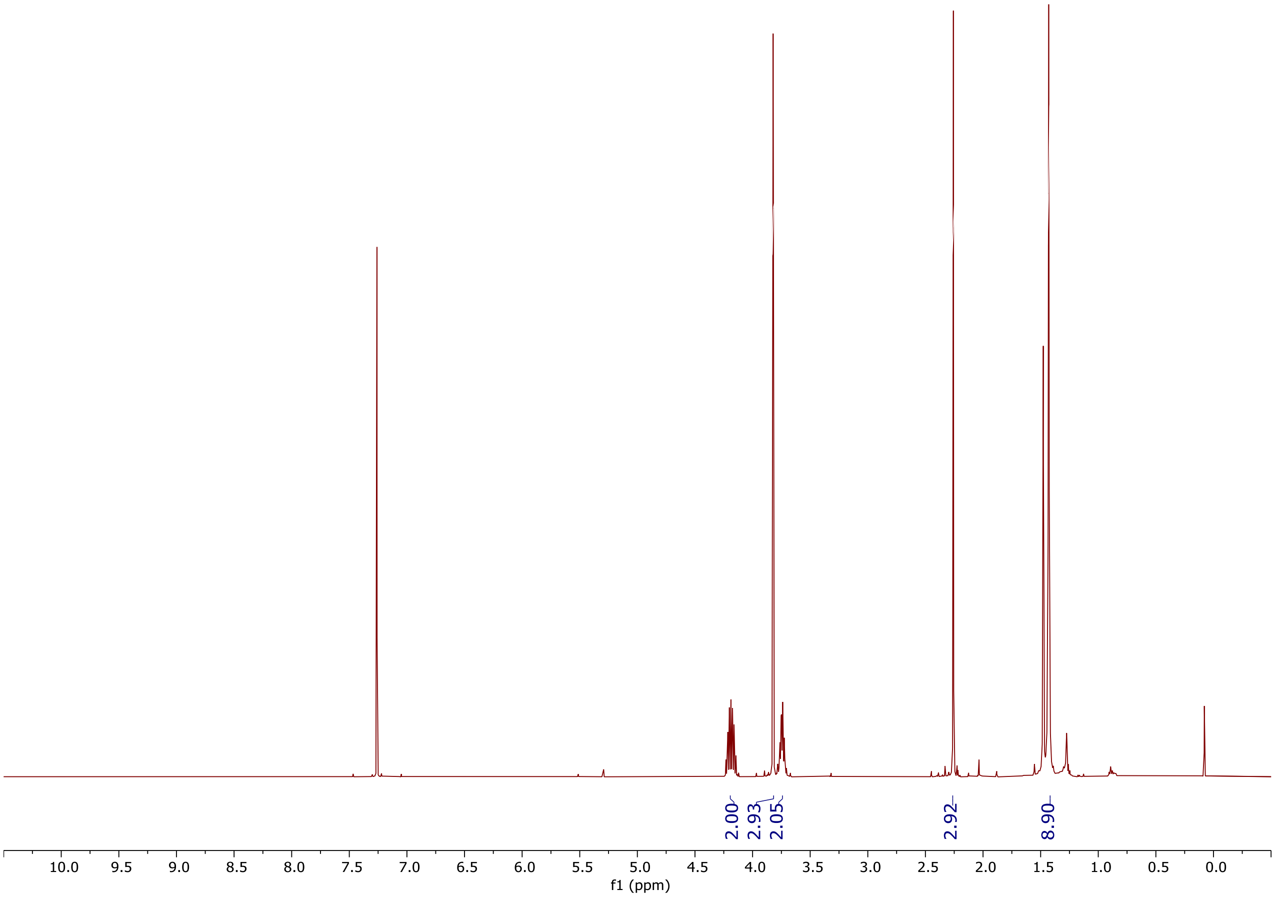

^{13}C NMR (126 MHz, $CDCl_3$) 9-(*tert*-Butyl) 2-methyl-3-methyl-1,4,6-trioxa-9-azaspiro[4.4]non-2-ene-2,9-dicarboxylate **10a**

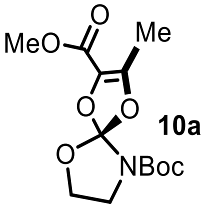

10a

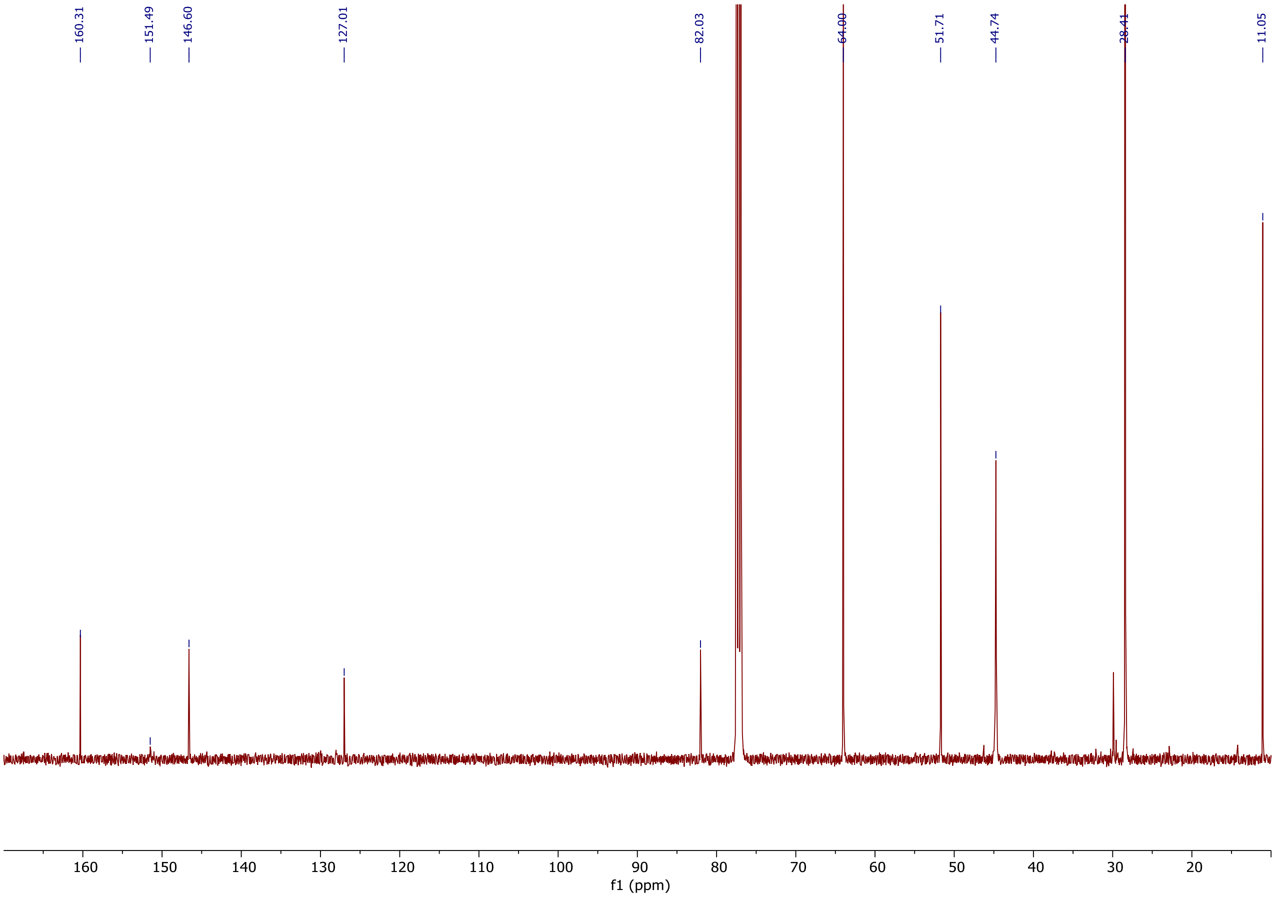

^{1}H NMR (500 MHz, CDCl3) 9-(*tert*-Butyl) 2-methyl-3-isopropyl-1,4,6-trioxa-9-azaspiro[4.4]non-2-ene-2,9-dicarboxylate **10b**

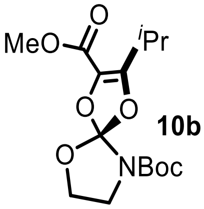

10b

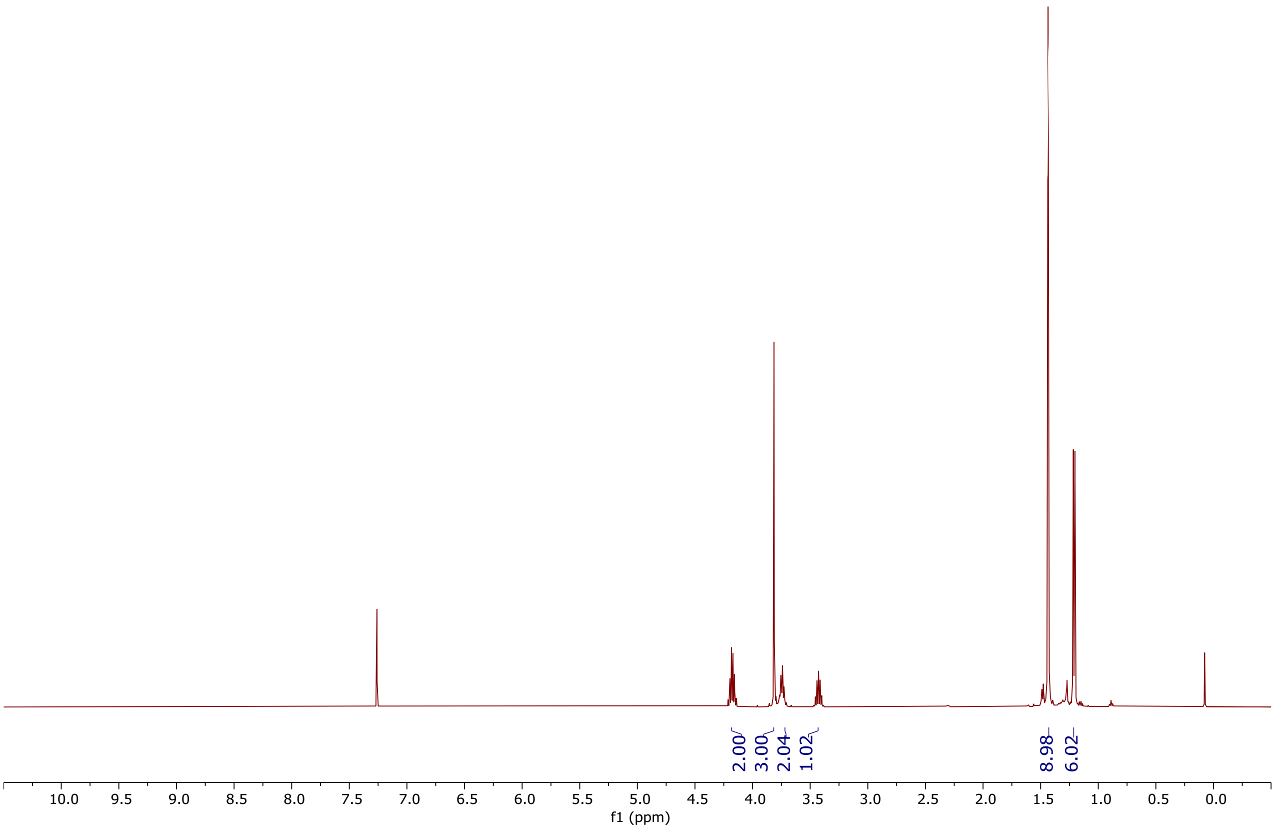

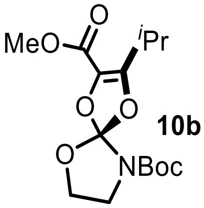

10b

^{13}C NMR (126 MHz, $CDCl_3$) 9-(*tert*-Butyl) 2-methyl-3-isopropyl-1,4,6-trioxa-9-azaspiro[4.4]non-2-ene-2,9-dicarboxylate **10b**

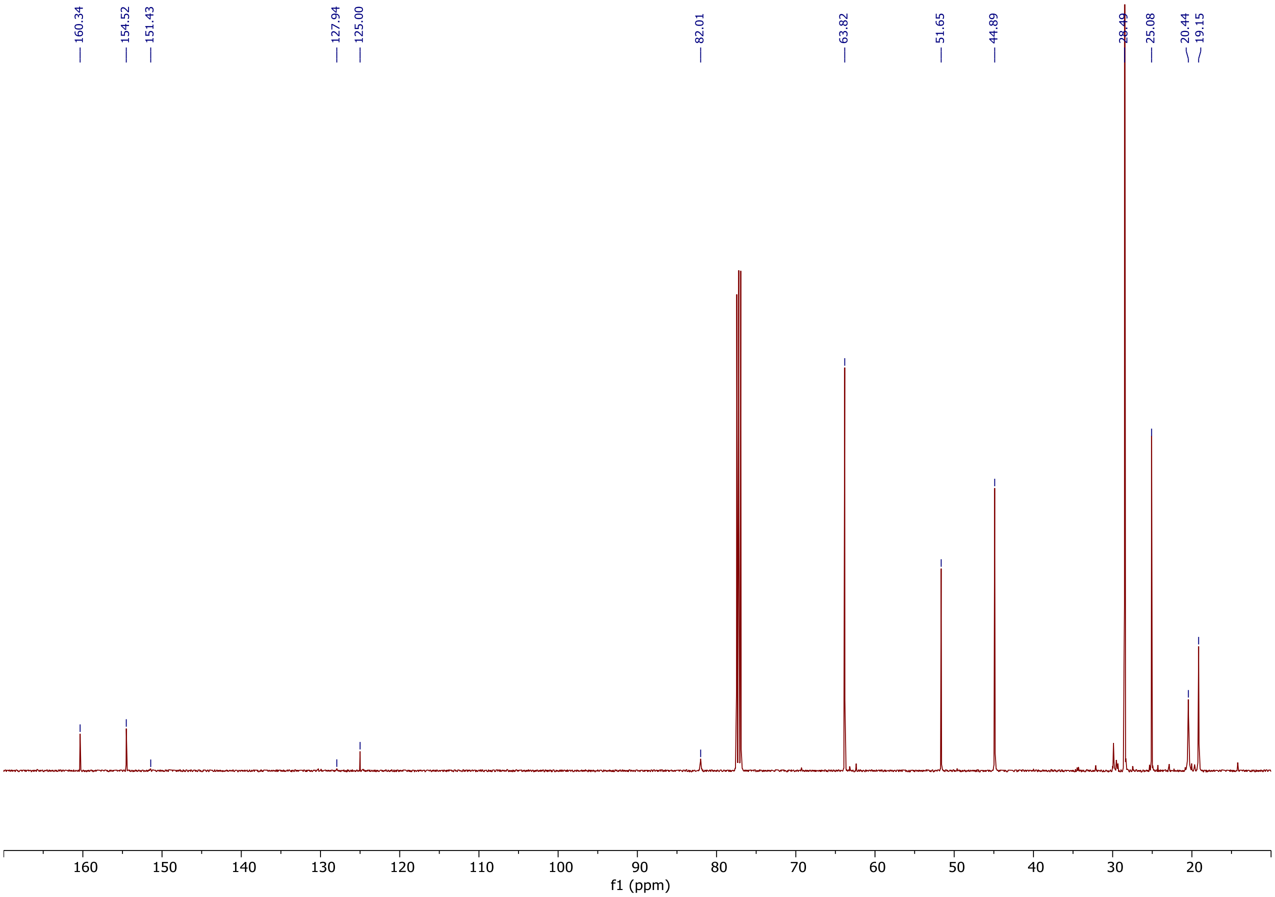

^{1}H NMR (500 MHz, DMSO-d_6) 2-methyl 9-(2,2,2-trichloroethyl) -3-methyl -1,4,6-trioxa-9-azaspiro[4.4]non-2-ene-2,9-dicarboxylate **10c**

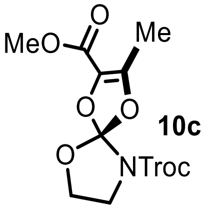

10c

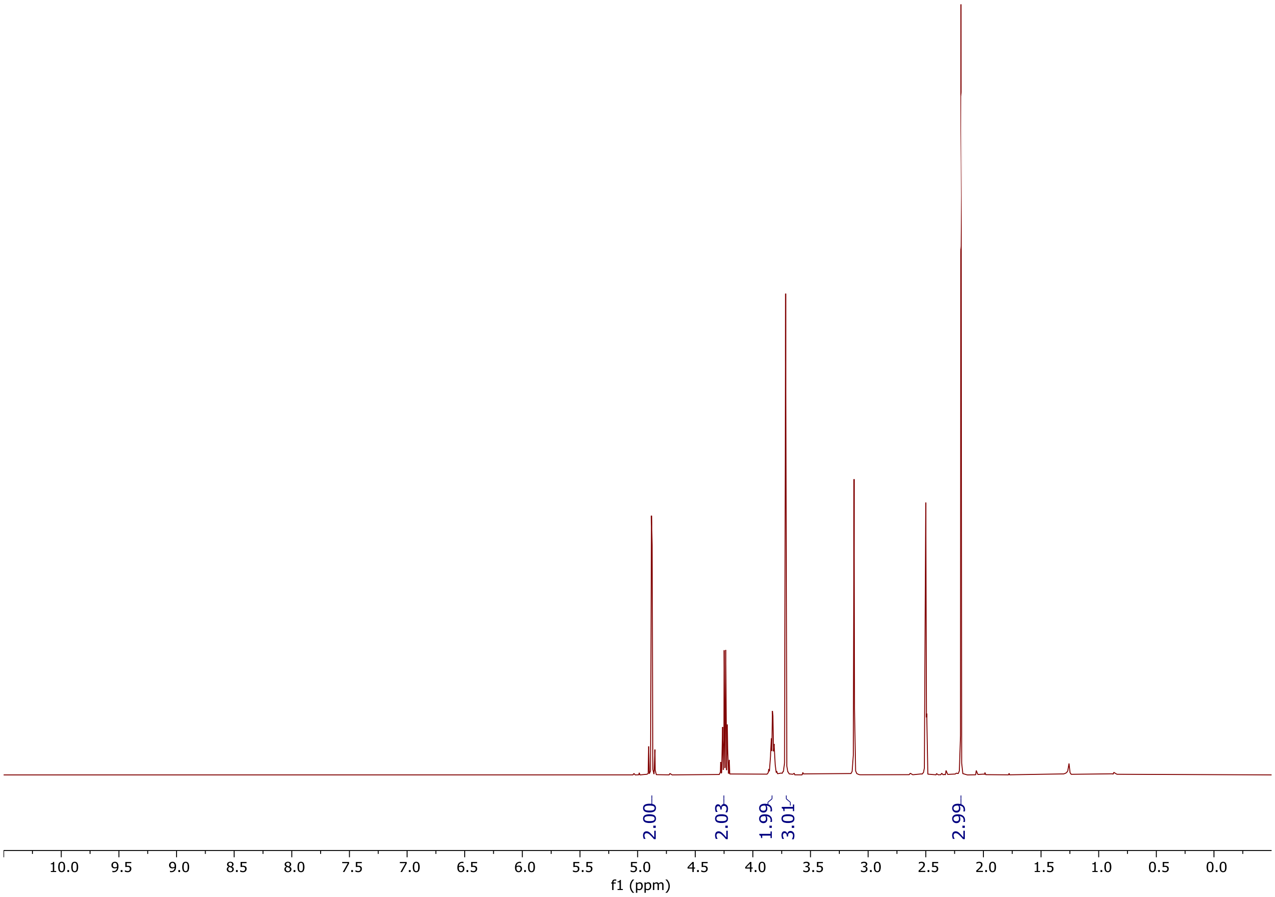

^{13}C NMR (126 MHz, DMSO-d$_6$) 2-methyl 9-(2,2,2-trichloroethyl) -3-methyl -1,4,6-trioxa-9-azaspiro[4.4]non-2-ene-2,9-dicarboxylate **10c**

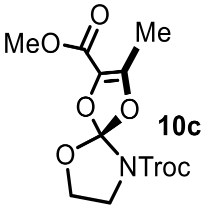

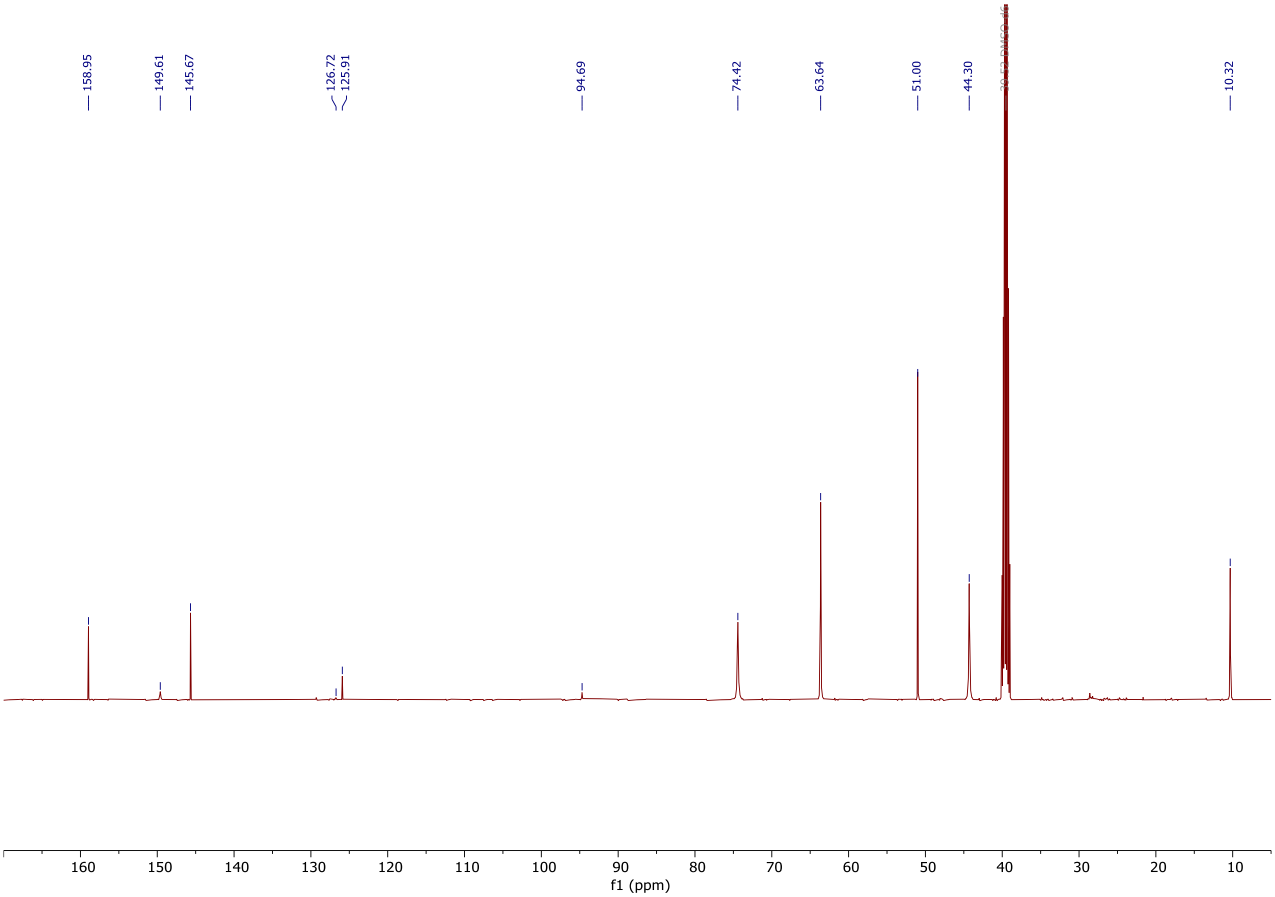

^{1}H NMR (300 MHz, DMSO-d_6) 2-methyl 9-(2,2,2-trichloroethyl) -3-isopropyl -1,4,6-trioxa-9-azaspiro[4.4]non-2-ene-2,9-dicarboxylate
10d

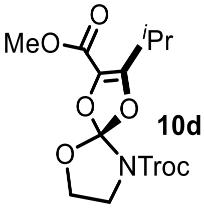

10d

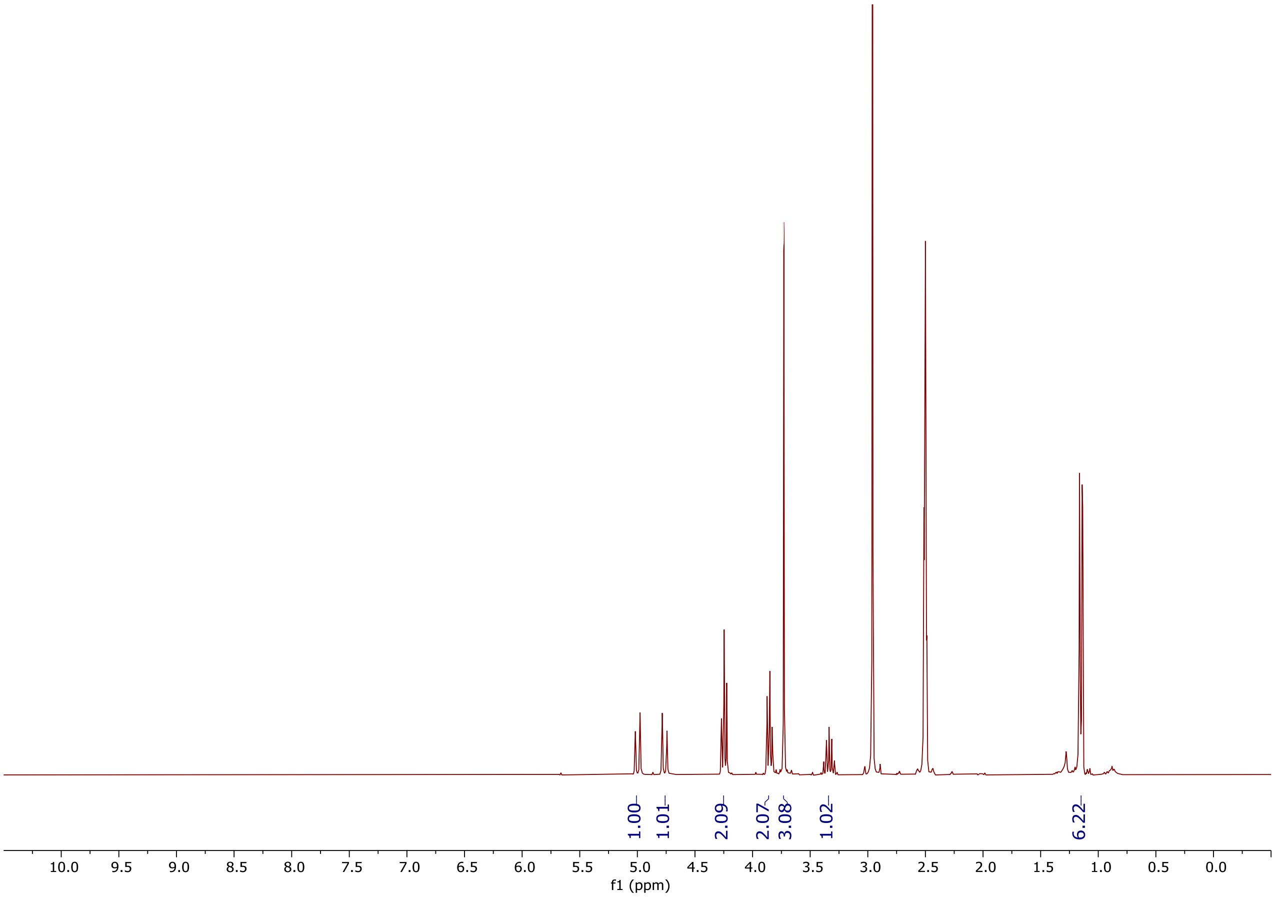

^{13}C NMR (126 MHz, DMSO-d$_6$) 2-methyl 9-(2,2,2-trichloroethyl) -3-isopropyl -1,4,6-trioxa-9-azaspiro[4.4]non-2-ene-2,9-dicarboxylate **10d**

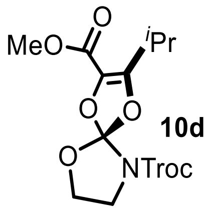

10d

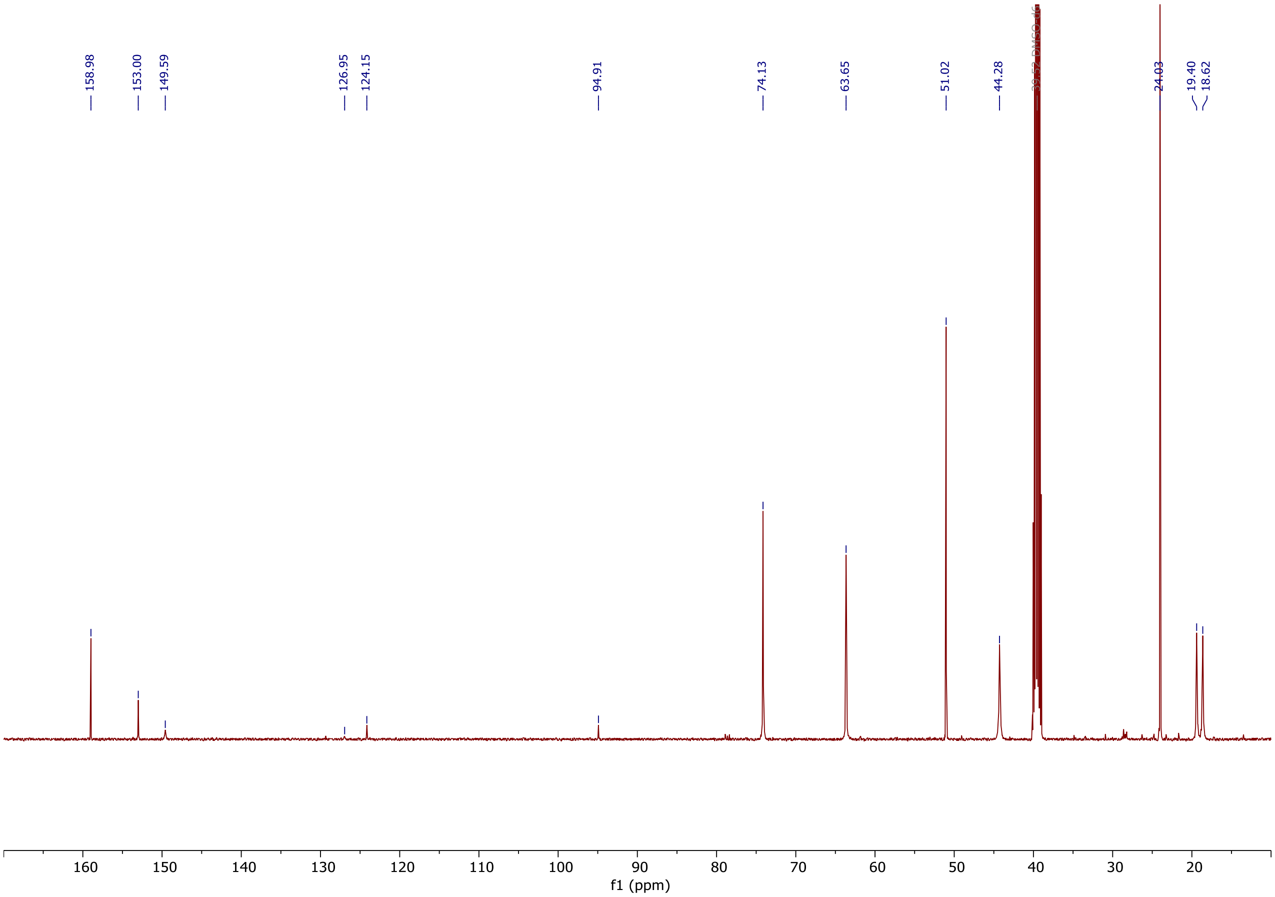

^{1}H NMR (500 MHz, DMSO-d_6) 9-allyl 2-methyl -3-methyl-1,4,6-trioxa-9-azaspiro[4.4]non-2-ene-2,9-dicarboxylate **10e**

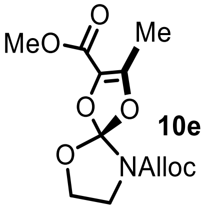

10e

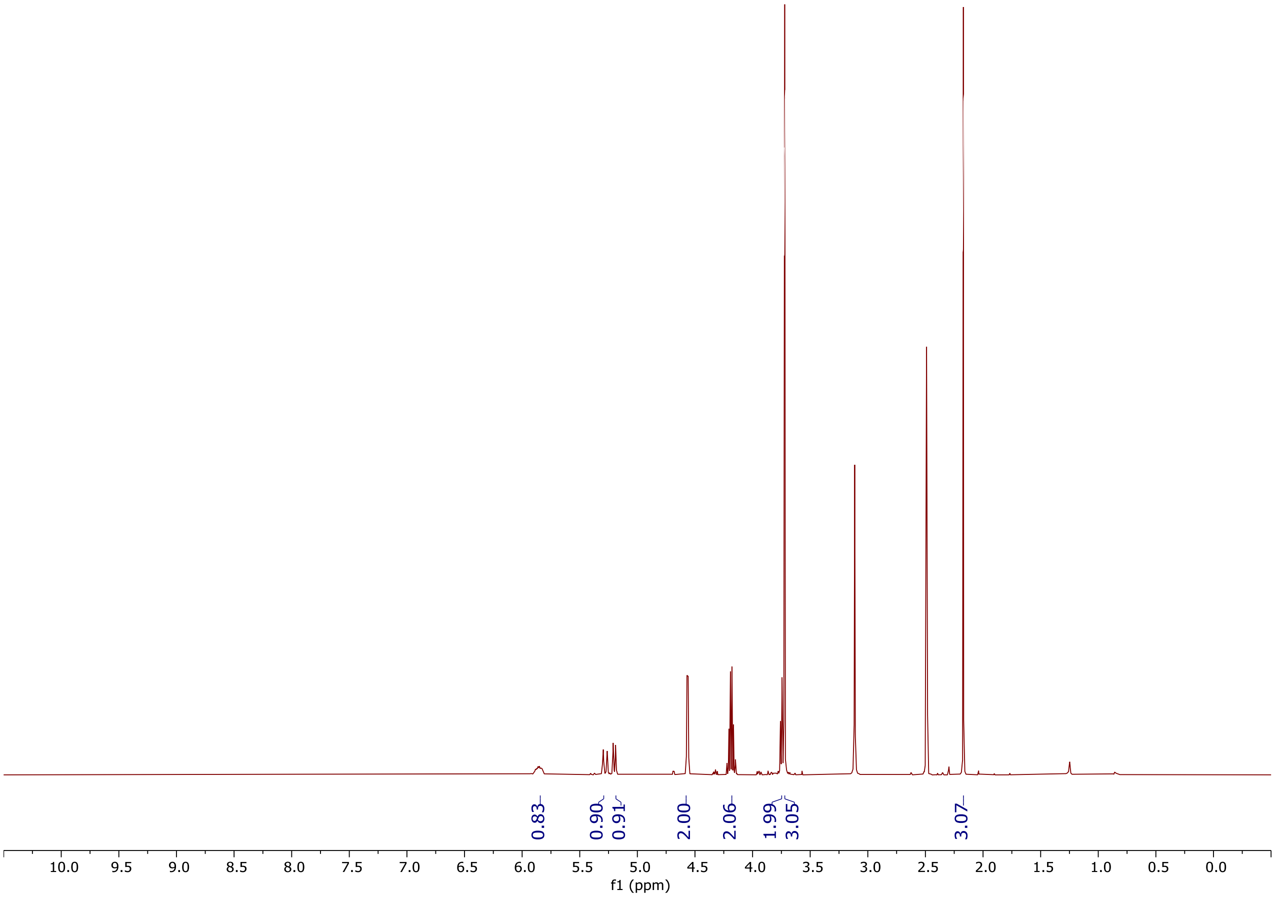

^{13}C NMR (126 MHz, DMSO-d_6) 9-allyl 2-methyl -3-methyl-1,4,6-trioxa-9-azaspiro[4.4]non-2-ene-2,9-dicarboxylate **10e**

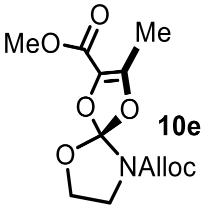

10e

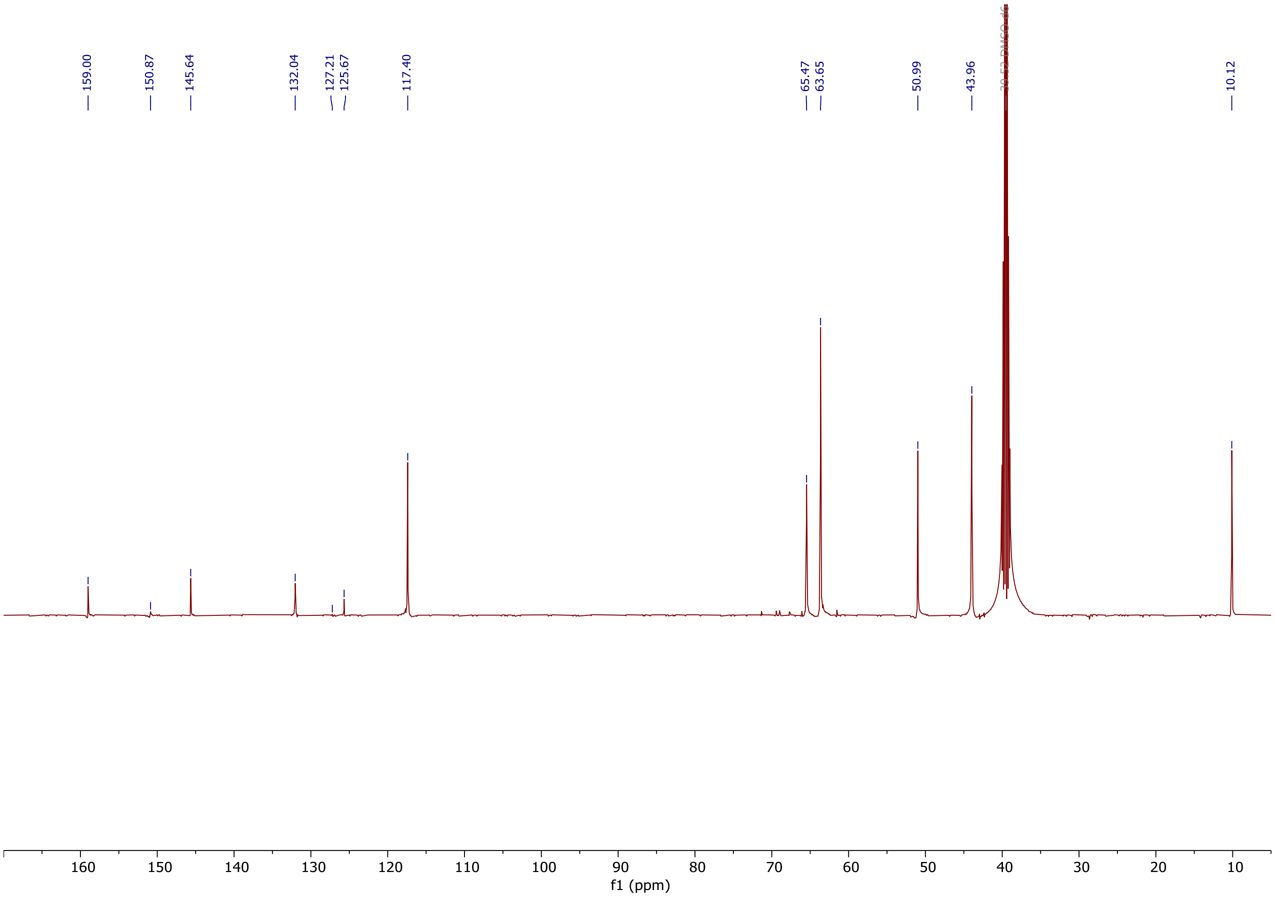

^{1}H NMR (500 MHz, DMSO-d_6) 9-allyl 2-methyl -3-isopropyl-1,4,6-trioxa-9-azaspiro[4.4]non-2-ene-2,9-dicarboxylate **10f**

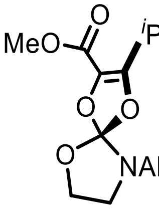

10f

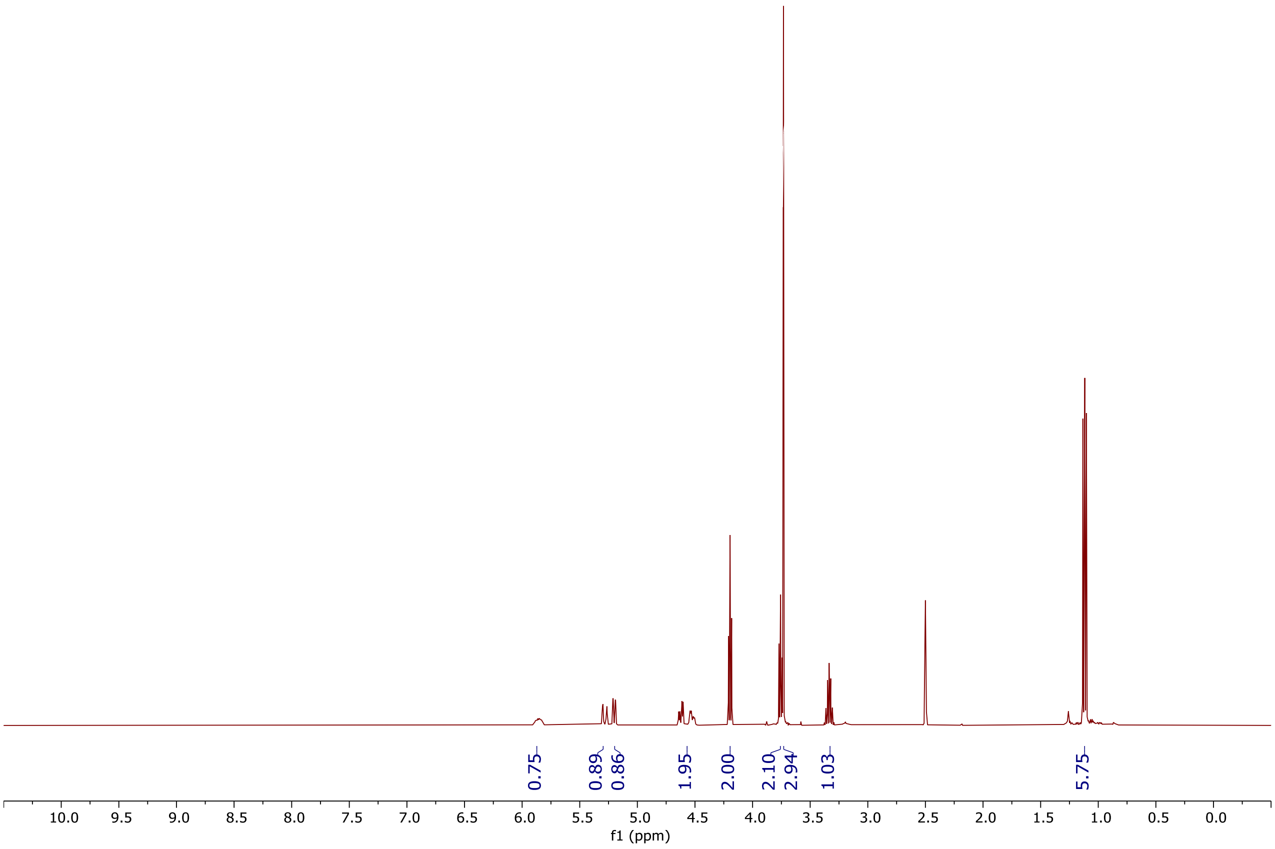

^{13}C NMR (126 MHz, DMSO-d_6) 9-allyl 2-methyl -3-isopropyl-1,4,6-trioxa-9-azaspiro[4.4]non-2-ene-2,9-dicarboxylate **10f**

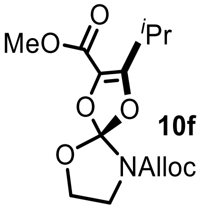

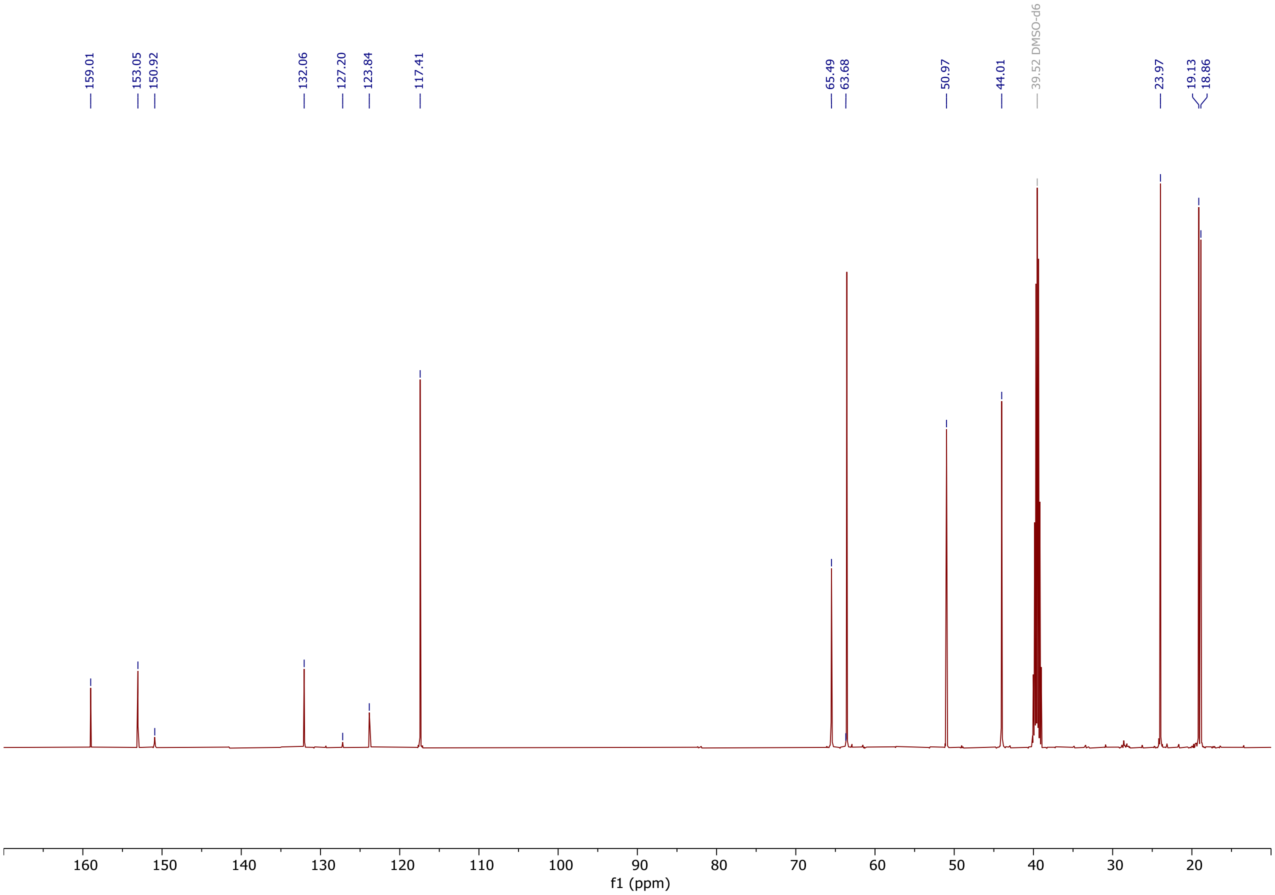

^{1}H NMR (500 MHz, $CDCl_3$) 9-(*tert*-Butyl) 2-ethyl-3-methyl-1,4,6-trioxa-9-azaspiro[4.4]non-2-ene-2,9-dicarboxylate **10g**

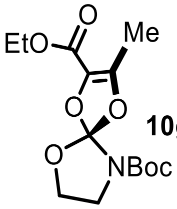

10g

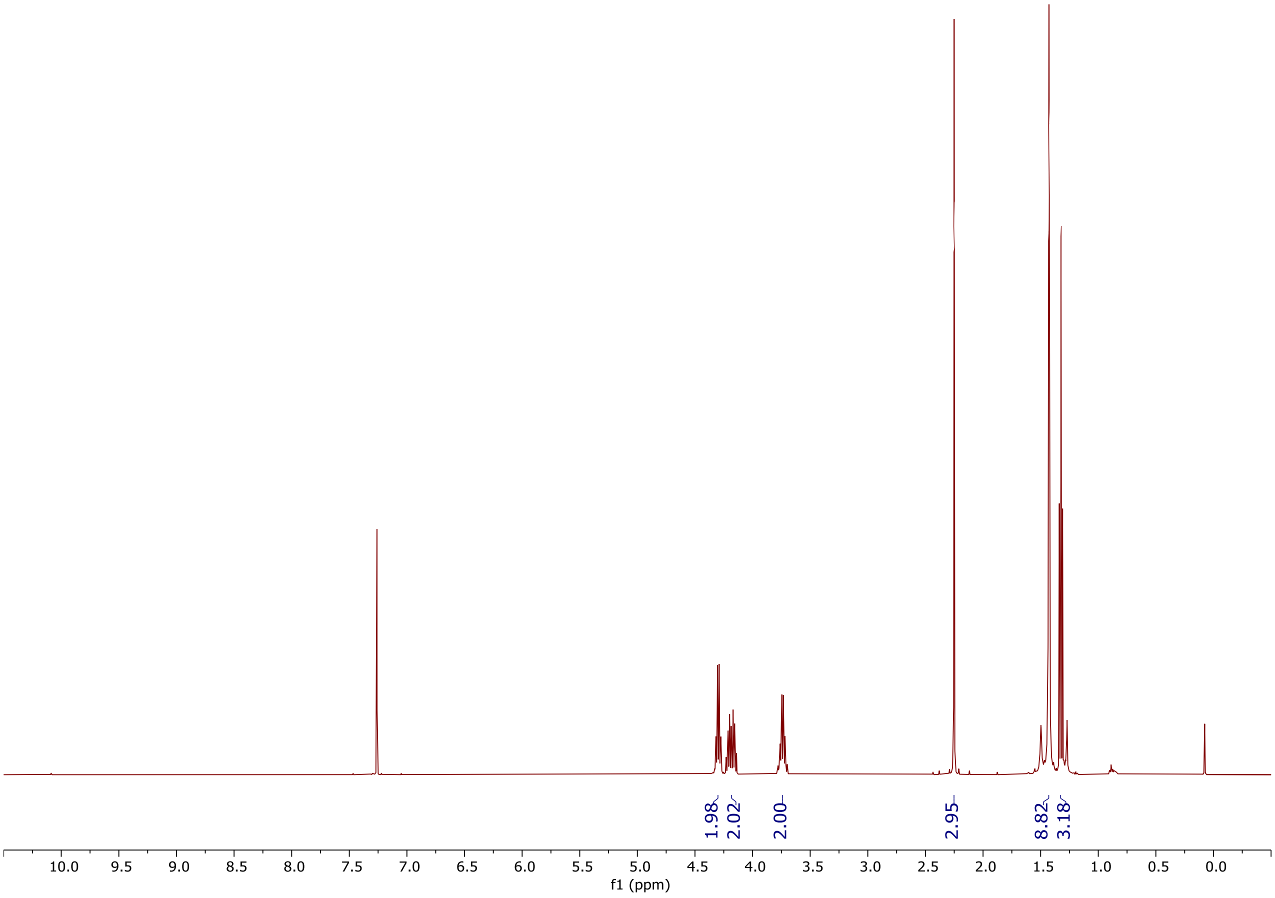

^{13}C NMR (126 MHz, $CDCl_3$) 9-(*tert*-Butyl) 2-ethyl-3-methyl-1,4,6-trioxa-9-azaspiro[4.4]non-2-ene-2,9-dicarboxylate **10g**

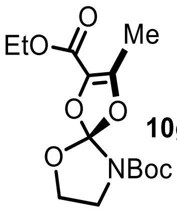

10g

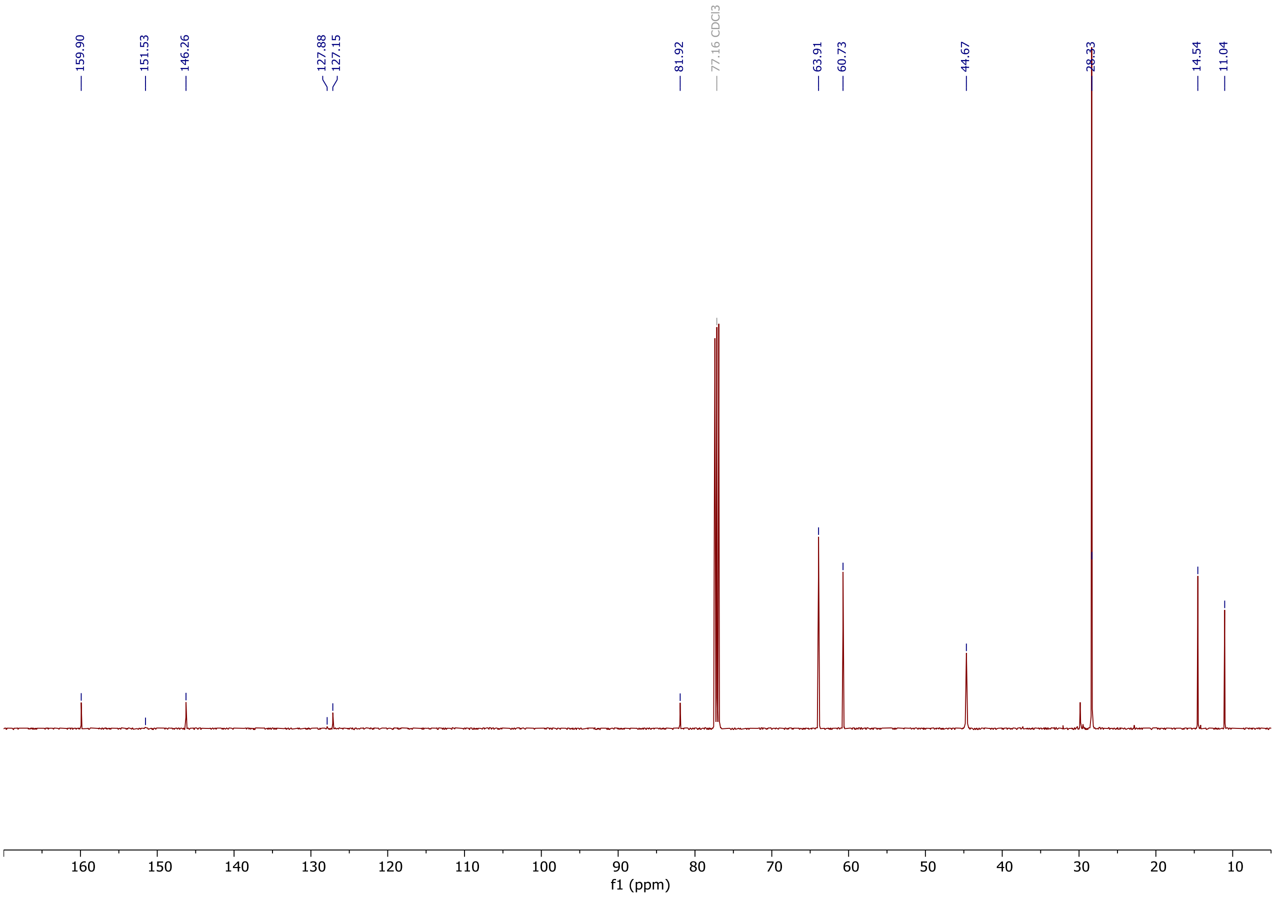

^{1}H NMR (500 MHz, $CDCl_3$) 9-(*tert*-Butyl) 2-isopropyl-3-methyl-1,4,6-trioxa-9-azaspiro[4.4]non-2-ene-2,9-dicarboxylate **10h**

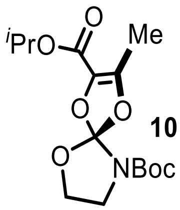

10h

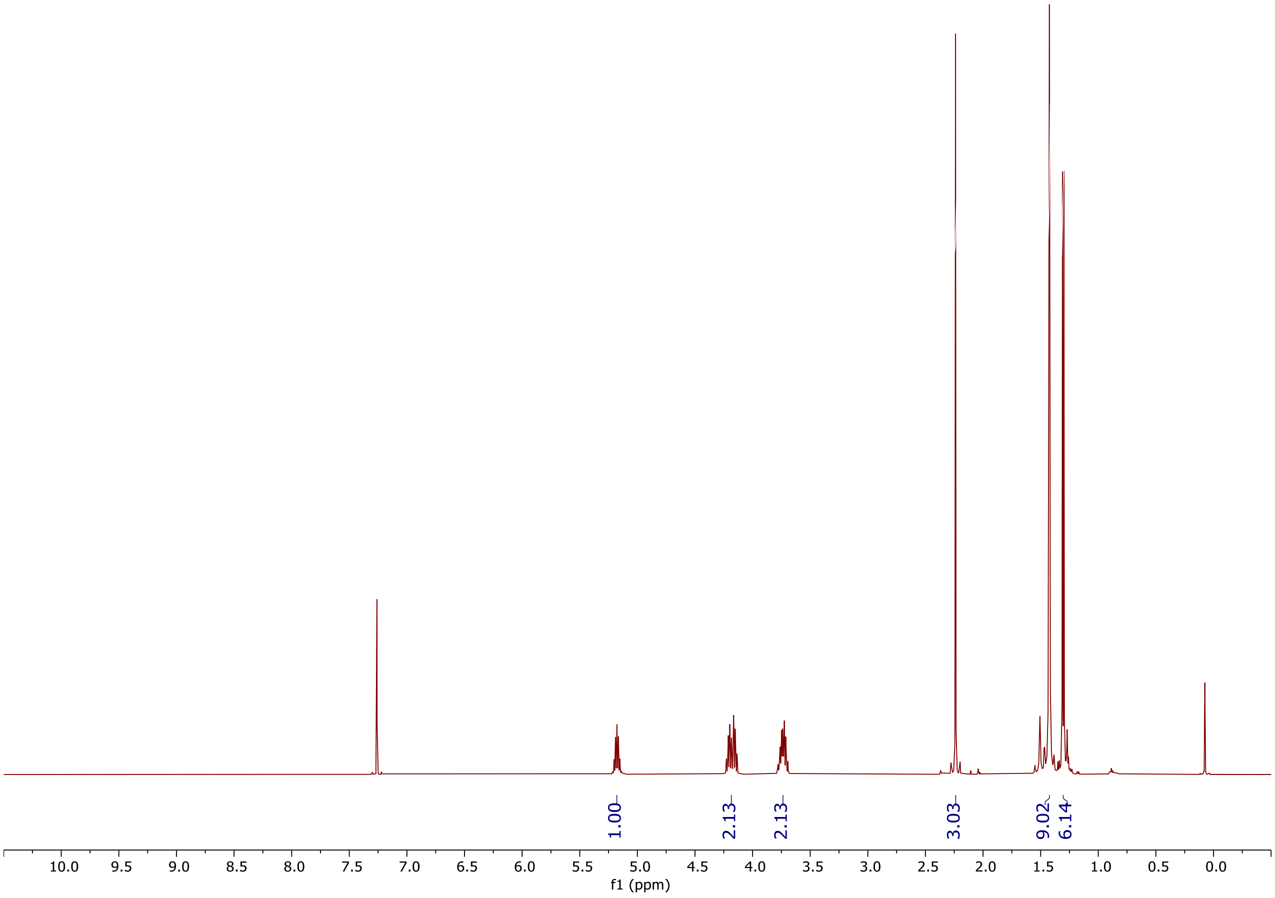

^{13}C NMR (126 MHz, $CDCl_3$) 9-(*tert*-Butyl) 2-isopropyl-3-methyl-1,4,6-trioxa-9-azaspiro[4.4]non-2-ene-2,9-dicarboxylate **10h**

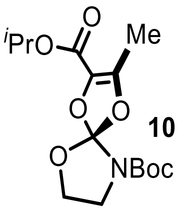

10h

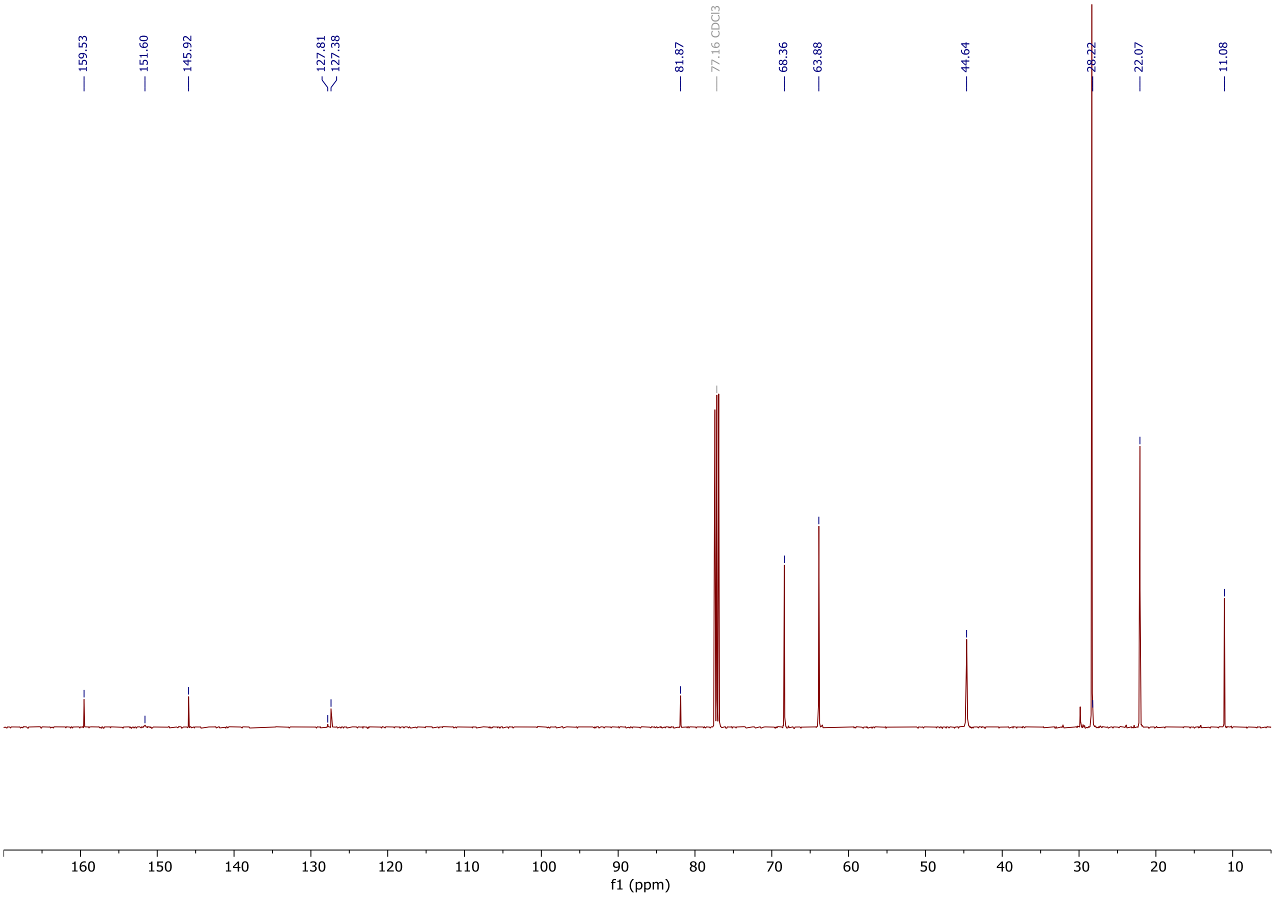

^{1}H NMR (500 MHz, $CDCl_3$) 9-(*tert*-Butyl) 2-methyl (5S,8S)-8-isopropyl-3-methyl-1,4,6-trioxa-9-azaspiro[4.4]non-2-ene-2,9-dicarboxylate **10i’**

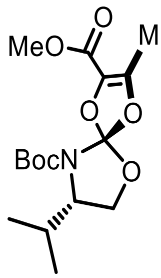

10i'

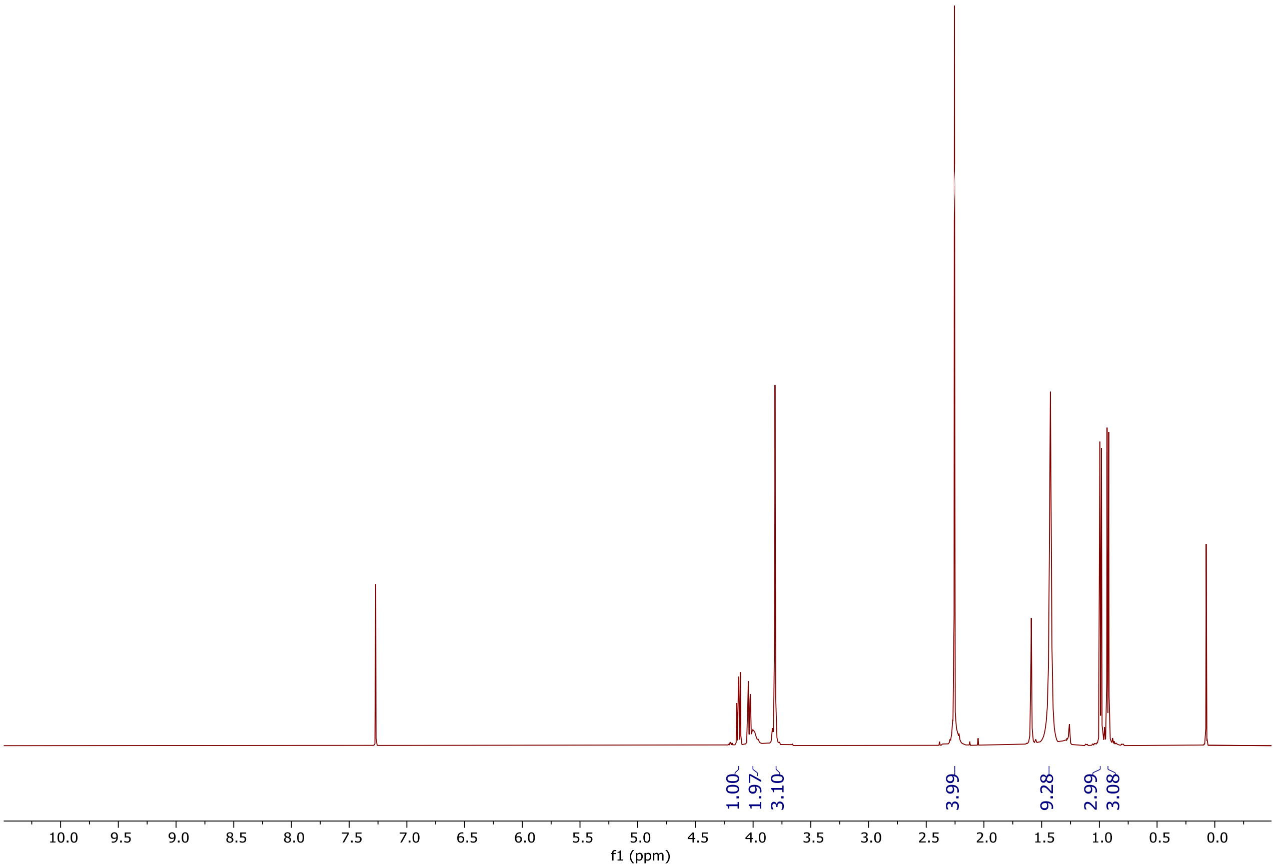

^{13}C NMR (126 MHz, $CDCl_3$) 9-(*tert*-Butyl) 2-methyl (5S,8S)-8-isopropyl-3-methyl-1,4,6-trioxa-9-azaspiro[4.4]non-2-ene-2,9-dicarboxylate **10i’**

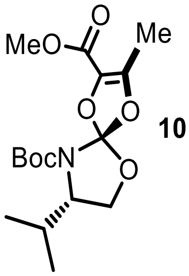

10i'

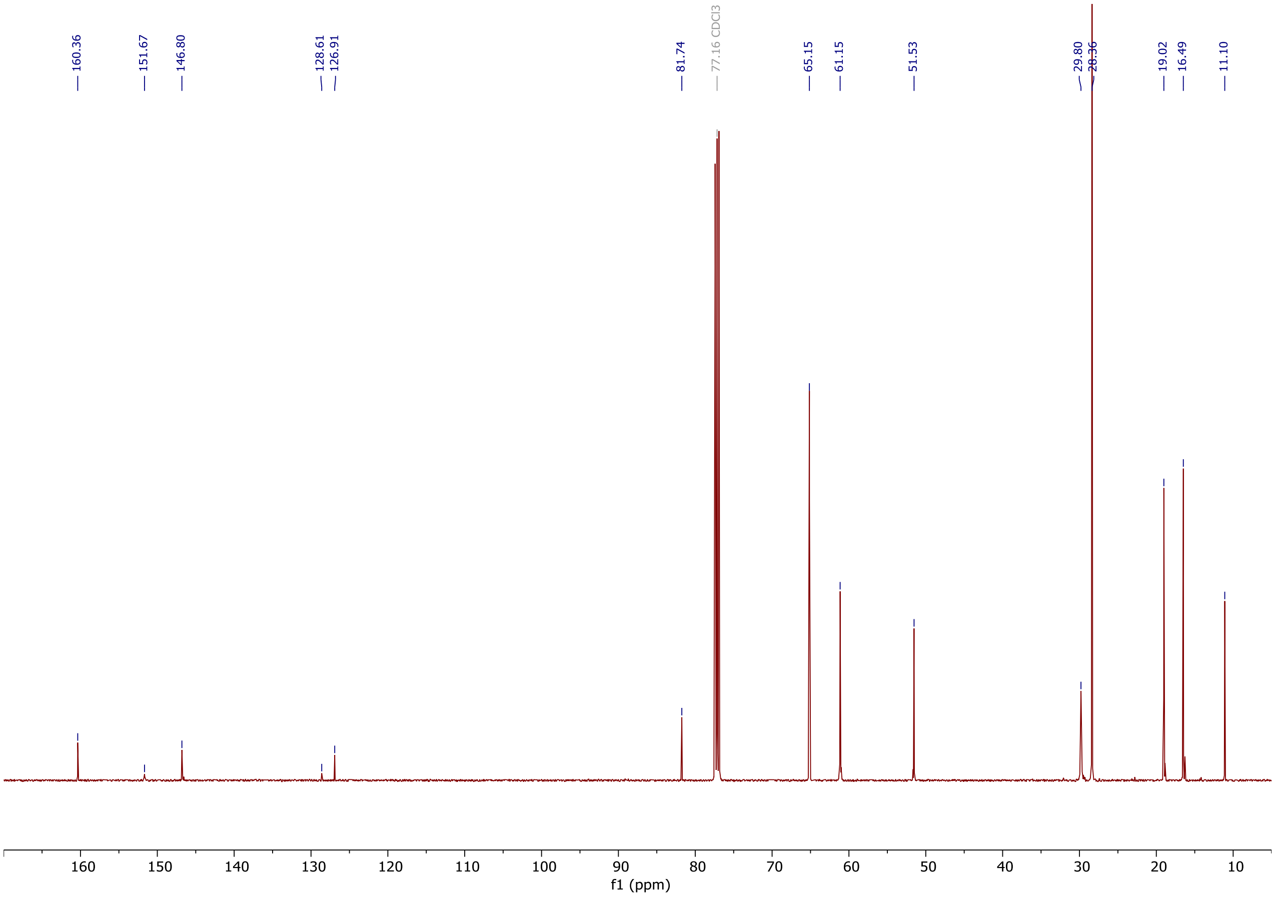

^{1}H NMR (500 MHz, $CDCl_3$) 9-(*tert*-Butyl) 2-methyl (5R,8S)-8-isopropyl-3-methyl-1,4,6-trioxa-9-azaspiro[4.4]non-2-ene-2,9-dicarboxylate **10i"**

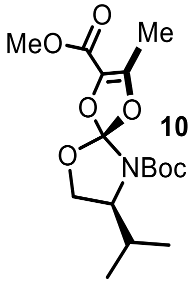

10i"

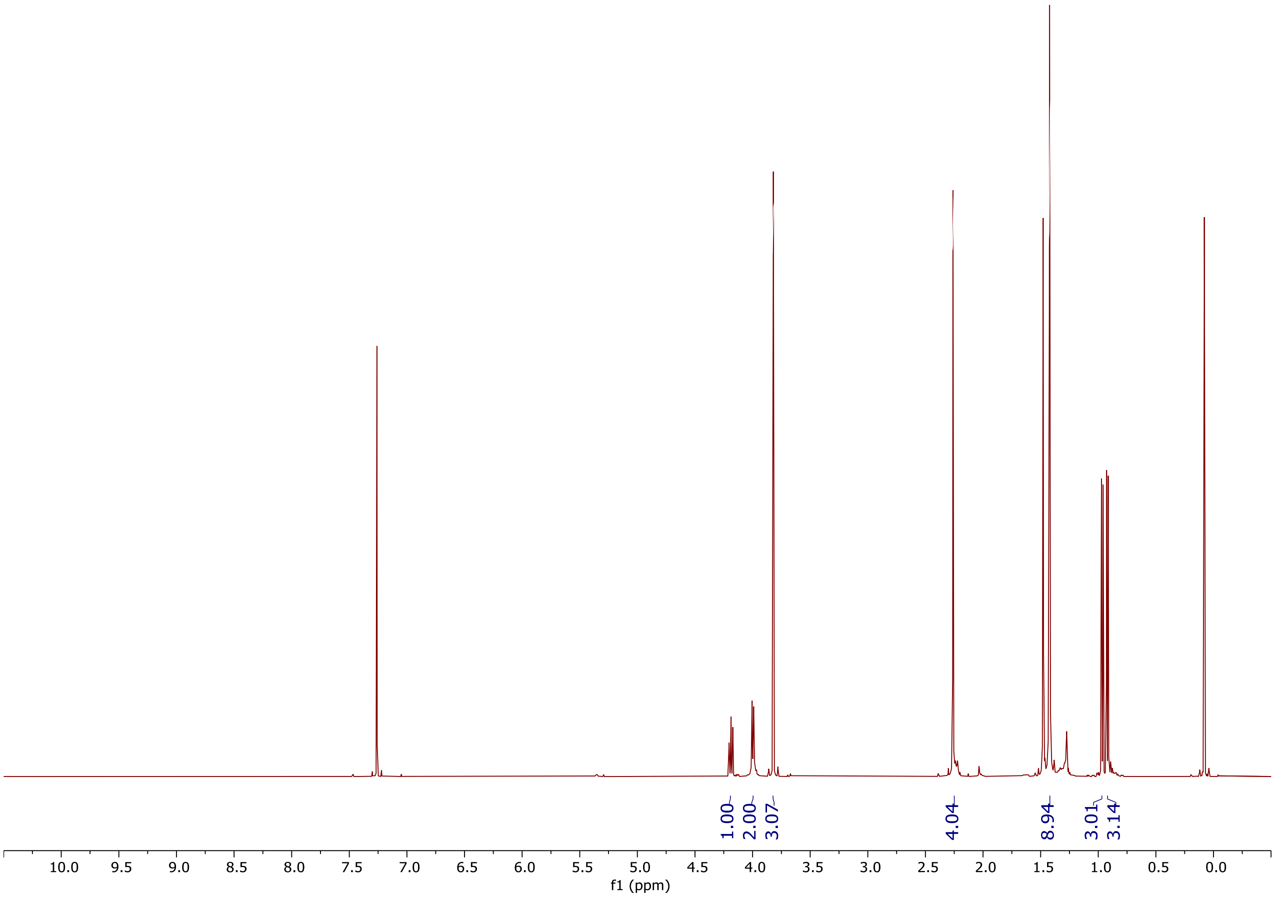

^{13}C NMR (126 MHz, $CDCl_3$) 9-(*tert*-Butyl) 2-methyl (5R,8S)-8-isopropyl-3-methyl-1,4,6-trioxa-9-azaspiro[4.4]non-2-ene-2,9-dicarboxylate **10i"**

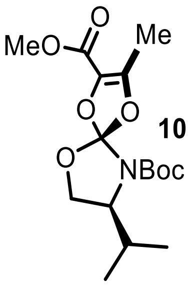

10i"

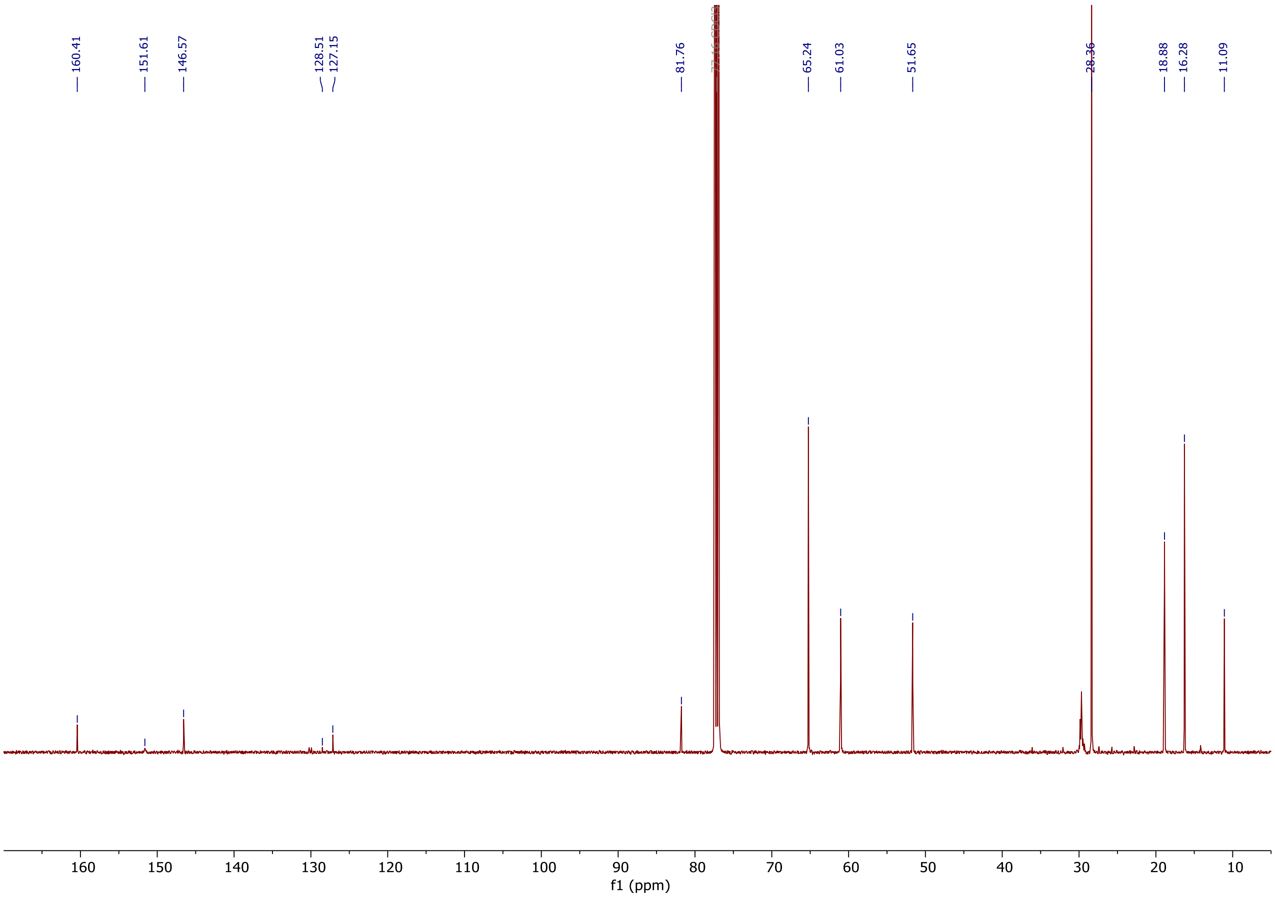

^1H NMR (500 MHz, $CDCl_3$) 9-(*tert*-Butyl) 2-methyl (5S,8S)-3,8-diisopropyl -1,4,6- trioxa-9-azaspiro[4.4]non-2-ene-2,9-dicarboxylate
10j'

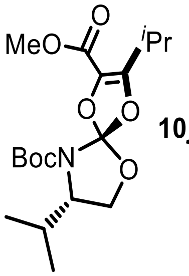

10j'

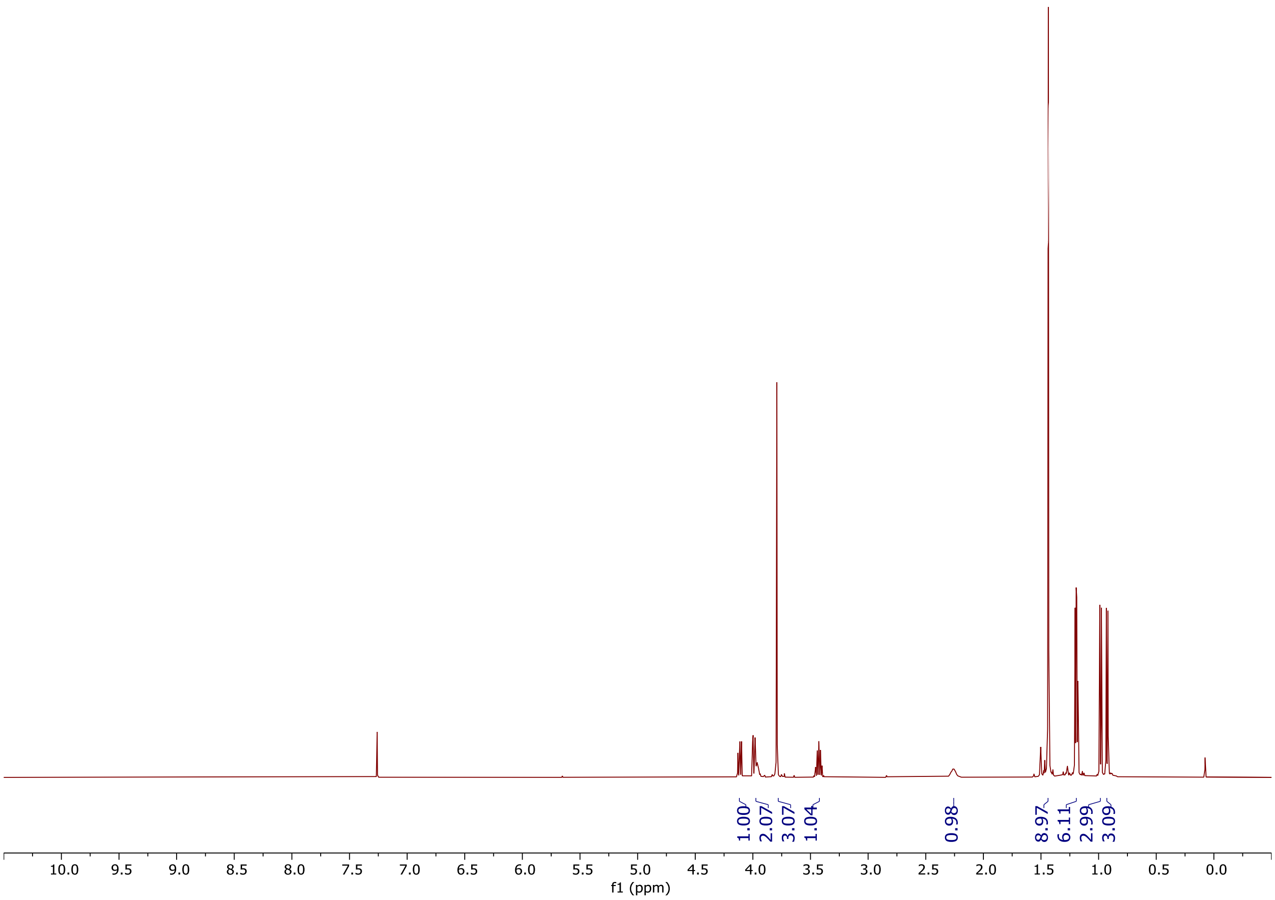

^{13}C NMR (126 MHz, $CDCl_3$) 9-(*tert*-Butyl) 2-methyl (5S,8S)-3,8-diisopropyl -1,4,6- trioxa-9-azaspiro[4.4]non-2-ene-2,9-dicarboxylate
10j’

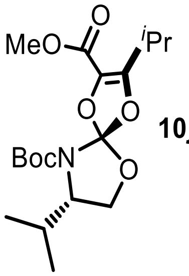

10j'

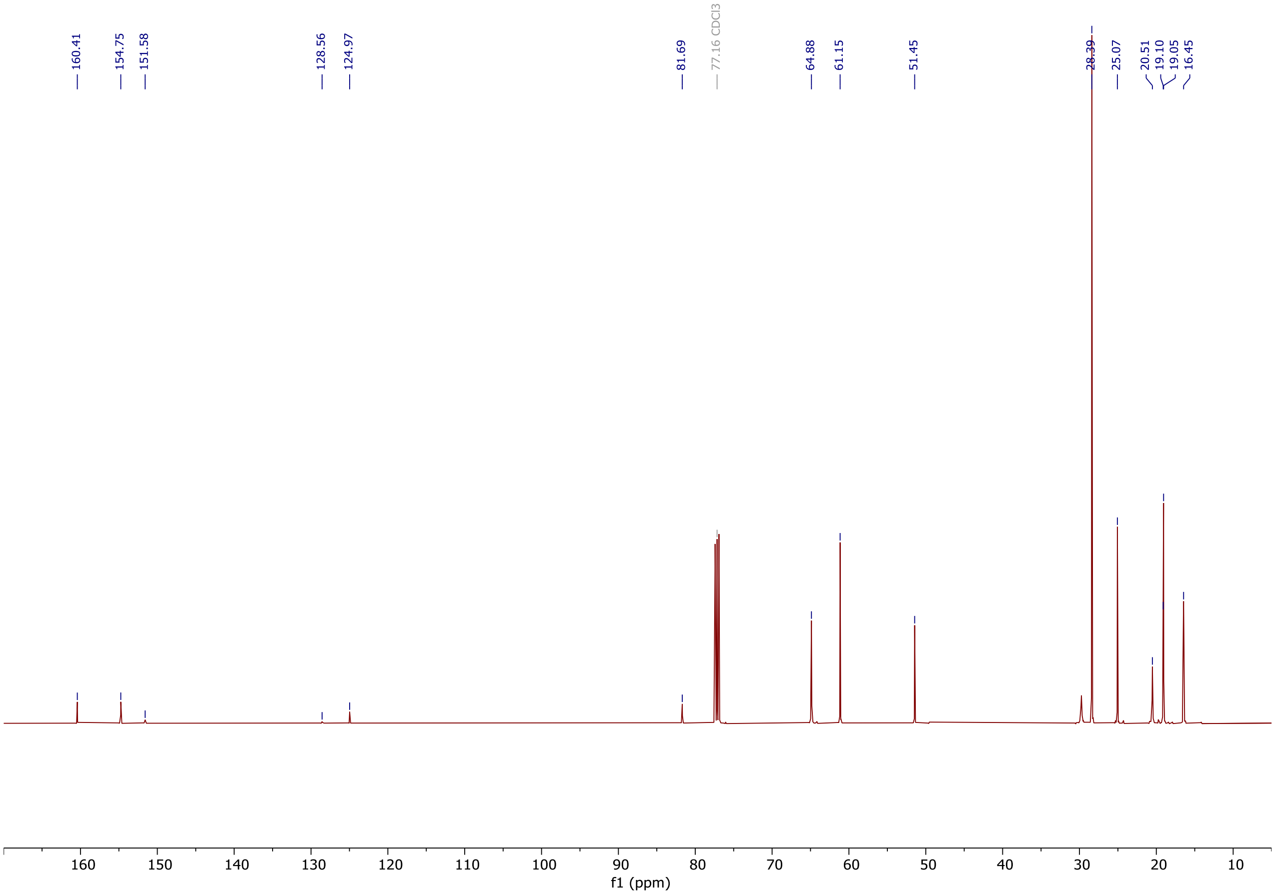

^{1}H NMR (500 MHz, $CDCl_3$) 9-(*tert*-Butyl) 2-methyl (5R,8S)-3,8-diisopropyl -1,4,6- trioxa-9-azaspiro[4.4]non-2-ene-2,9-dicarboxylate
10j''

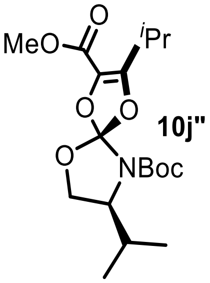

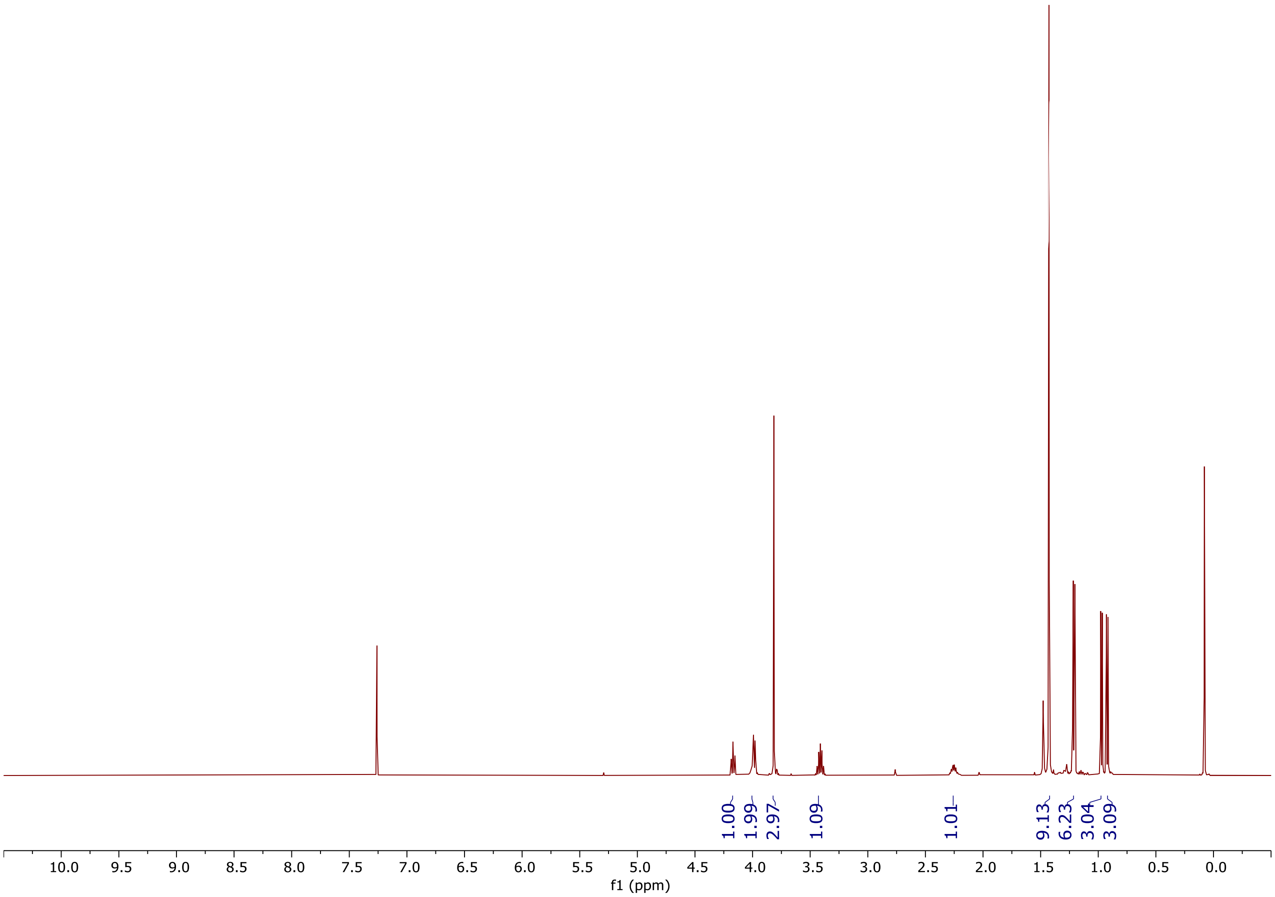

^{13}C NMR (126 MHz, $CDCl_3$) 9-(*tert*-Butyl) 2-methyl (5R,8S)-3,8-diisopropyl -1,4,6- trioxa-9-azaspiro[4.4]non-2-ene-2,9-dicarboxylate **10j''**

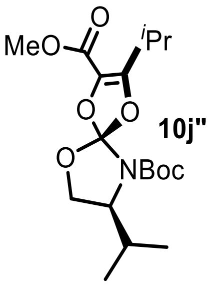

10j''

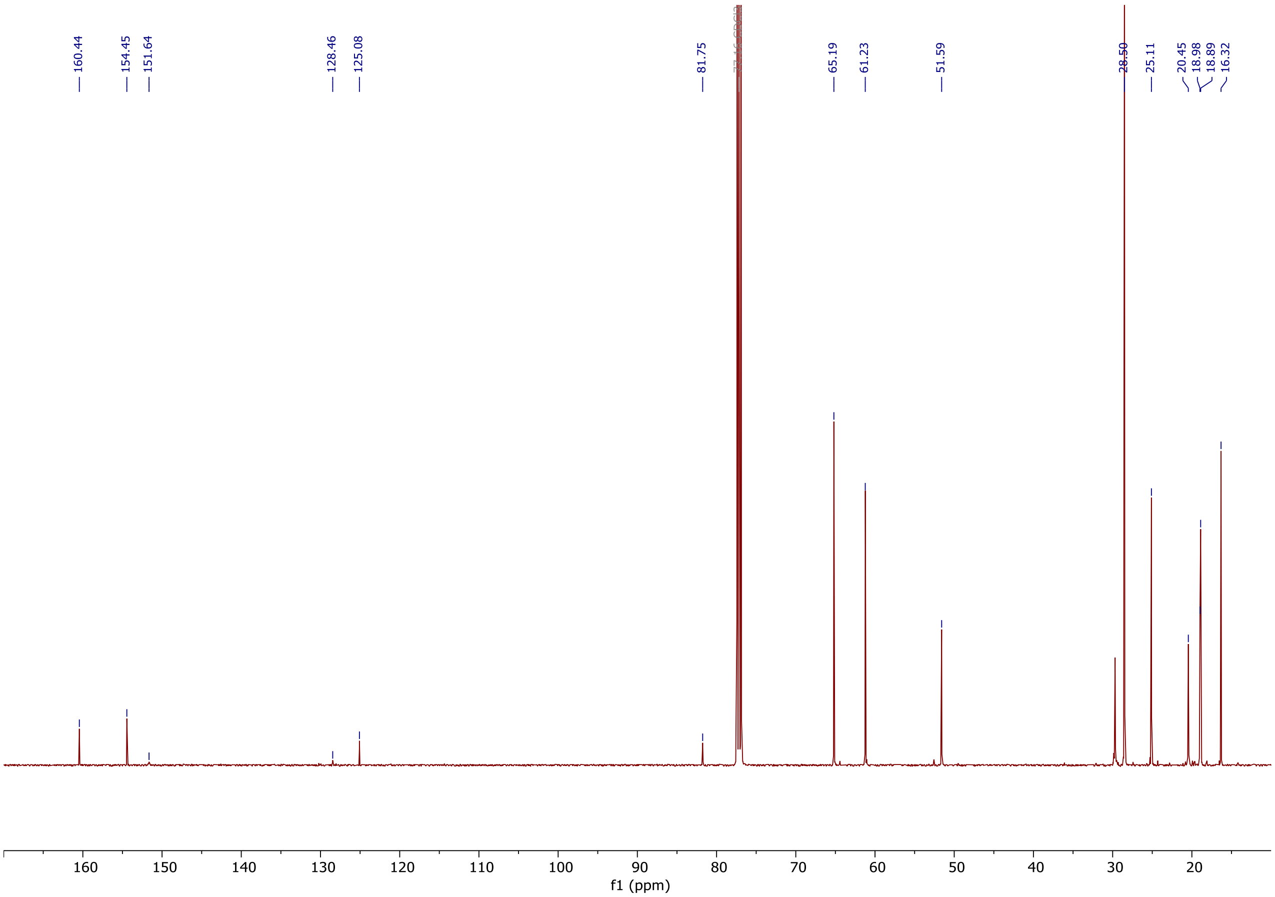

10. Cartesian coordinates

Table S17. Cartesian coordinates of compound **9a** (most stable conformers) optimized at B3LYP-D3BJ/6-311+G(d,p)/PCM level. All structures have 0 imaginary frequencies.

Conf. 1. SCF –841.132803 hartree

Symbol	X	Y	Z
C	-1.3521650	0.0034590	0.0408380
C	-1.0904940	1.3214940	-0.0373340
O	0.2590130	1.5132400	0.0554890
C	0.8899010	0.2308790	0.2184740
O	-0.1613940	-0.7022720	0.1945470
O	1.5541990	0.1494760	1.4269480
C	2.9664170	0.2578120	1.1668580
H	3.4944380	-0.3141910	1.9266180
H	3.2686380	1.3072970	1.2052500
C	3.1007050	-0.3241390	-0.2440940
O	1.7922760	0.0454520	-0.8007280
C	-1.9240930	2.5313270	-0.1983120
H	-2.9743080	2.2624380	-0.2656670
H	-1.7734760	3.2023450	0.6521670
H	-1.6269050	3.0695080	-1.1029370
C	-2.5745960	-0.7829220	-0.0058760
O	-2.6036880	-1.9959020	0.0800860
O	-3.6685510	-0.0141110	-0.1552260
C	-4.9331360	-0.7072240	-0.2123660
H	-5.6800660	0.0720740	-0.3369500
H	-4.9505190	-1.3930030	-1.0592420
H	-5.1048490	-1.2573220	0.7127120
C	4.1735400	0.3485390	-1.0791020
H	4.0076900	1.4261010	-1.1248890
H	4.1762370	-0.0540270	-2.0938250
H	5.1550420	0.1632060	-0.6362440
C	3.2215050	-1.8437700	-0.2418070
H	4.1992960	-2.1396840	0.1451740
H	3.1192490	-2.2282480	-1.2582110
H	2.4492010	-2.2962980	0.3834330

Conf. 2. SCF –841.132797 hartree

Symbol	X	Y	Z
C	-1.4220620	0.3301690	0.0028990
C	-1.0095690	1.6086710	-0.0643700
O	0.3501900	1.6463070	0.0467720
C	0.8283920	0.2980680	0.2123530
O	-0.3224290	-0.5101290	0.1688300
O	1.4610400	0.1384070	1.4295250
C	2.8805350	0.0967140	1.1900740
H	3.3332720	-0.5302890	1.9549060
H	3.2918200	1.1080430	1.2370020
C	2.9725370	-0.4928030	-0.2212060
O	1.7182630	0.0139600	-0.7946860
C	-1.7162290	2.8950210	-0.2307620
H	-2.7862200	2.7222670	-0.3150410
H	-1.5177530	3.5454550	0.6259250
H	-1.3568500	3.4066340	-1.1281720
C	-2.7637230	-0.2228080	-0.0776520
O	-3.7736820	0.4451410	-0.2183220
O	-2.7583810	-1.5619470	0.0206850
C	-4.0466080	-2.2099410	-0.0501320
H	-3.8383090	-3.2718510	0.0475520
H	-4.6862780	-1.8682150	0.7634310
H	-4.5228510	-1.9992940	-1.0075430
C	4.1218330	0.0645140	-1.0392950
H	4.0741280	1.1539400	-1.0795220
H	4.0928400	-0.3302630	-2.0566680
H	5.0721350	-0.2287610	-0.5868980
C	2.9322300	-2.0166660	-0.2225210
H	3.8675130	-2.4145550	0.1780640
H	2.8053470	-2.3864660	-1.2416100
H	2.1071830	-2.3861910	0.3898090

Conf. 3. SCF –841.132736 hartree

Symbol	X	Y	Z
C	-1.3746150	-0.0417020	0.0421510
C	-1.0346960	1.2594640	-0.0088750
O	0.3237360	1.3683760	0.1001730
C	0.8768950	0.0510680	0.2332520
O	-0.2290180	-0.8205500	0.1776540
O	1.5461820	-0.0209650	1.4416200
C	2.8379170	-0.6153320	1.2104880
H	2.7763810	-1.6952270	1.3646630
H	3.5441750	-0.1744170	1.9107410
C	3.1248600	-0.2632500	-0.2527110
O	1.7548210	-0.2399870	-0.7807850
C	-1.7917730	2.5204810	-0.1529010
H	-2.8555430	2.3169130	-0.2352670
H	-1.6101170	3.1647350	0.7120900
H	-1.4531990	3.0570290	-1.0438590
C	-2.6423720	-0.7514280	-0.0250390
O	-2.7437830	-1.9623260	0.0273300
O	-3.6885050	0.0848860	-0.1516640
C	-4.9915780	-0.5307770	-0.2301650
H	-5.0488380	-1.1848680	-1.1000800
H	-5.1951500	-1.1014380	0.6757580
H	-5.6916000	0.2946770	-0.3266160
C	3.7441200	1.1210100	-0.4121750
H	3.1826150	1.8693750	0.1504960
H	3.7519910	1.4077700	-1.4653180
H	4.7734310	1.1122410	-0.0462990
C	3.8924990	-1.3382060	-0.9981430
H	4.8906490	-1.4440430	-0.5667790
H	4.0029620	-1.0685370	-2.0501830
H	3.3772380	-2.2976500	-0.9292680

Conf. 4. SCF –841.132716 hartree

Symbol	X	Y	Z
C	-1.4354000	0.3022440	-0.0019340
C	-0.9394010	1.5515970	-0.0462930
O	0.4198350	1.4973430	0.0760360
C	0.8081630	0.1218620	0.2188830
O	-0.3940120	-0.6116400	0.1417700
O	1.4402960	-0.0277260	1.4400180
C	2.6587700	-0.7680870	1.2330260
H	2.4697620	-1.8332390	1.3861480
H	3.3982850	-0.4098890	1.9460220
C	3.0117190	-0.4547830	-0.2244650
O	1.6633310	-0.2741500	-0.7780150
C	-1.5561250	2.8853990	-0.1948090
H	-2.6352790	2.7875700	-0.2819180
H	-1.3146560	3.5091170	0.6706170
H	-1.1616060	3.3834370	-1.0851500
C	-2.8121600	-0.1570170	-0.0856740
O	-3.7743560	0.5801830	-0.2144590
O	-2.8986590	-1.4944090	-0.0036330
C	-4.2290790	-2.0508730	-0.0743660
H	-4.8382870	-1.6776550	0.7486830
H	-4.6956710	-1.7939600	-1.0251490
H	-4.0940940	-3.1259150	0.0070500
C	3.7899910	0.8481640	-0.3722060
H	3.3091500	1.6575980	0.1804820
H	3.8501650	1.1302990	-1.4249070
H	4.8045200	0.7206450	0.0123160
C	3.6633160	-1.6128580	-0.9555870
H	4.6346290	-1.8321650	-0.5060990
H	3.8231530	-1.3602900	-2.0055210
H	3.0396840	-2.5062590	-0.8957500

Table S18. Cartesian coordinates of compound (2*S*,1’*R*,2’*R*,3’*S*,5’*R*)-**9e’** (most stable conformers) optimized at B3LYP-D3BJ/6-311+G(d,p)/PCM level. All structures have 0 imaginary frequencies.

Conf. 1. SCF –1153.315996 hartree

Symbol	X	Y	Z
O	-1.0859090	1.1853370	0.1423420
O	-1.0541740	-1.0769460	0.0985610
O	0.5185850	0.0134970	1.3374660
O	0.6585940	0.0589480	-0.8887830
O	-3.1877320	-2.8144700	-0.0985690
O	-4.6372840	-1.0793870	-0.1675920
C	-0.2048720	0.0298200	0.1711870
C	-2.3685590	0.7402630	0.0381140
C	-3.4411460	1.7731320	-0.0196890
H	-4.3845930	1.2357220	-0.1055890
C	-3.4580690	2.6045960	1.2759350
H	-4.2764450	3.3269130	1.2369050
H	-2.5220760	3.1539980	1.3998320
H	-3.6029730	1.9674810	2.1512600
C	-3.2615250	2.6663490	-1.2606210
H	-2.3189070	3.2162920	-1.2129360
H	-4.0780340	3.3899610	-1.3124410
H	-3.2688720	2.0730270	-2.1776920
C	-2.3665770	-0.6068170	0.0100230
C	-3.4057970	-1.6172510	-0.0885010
C	-5.7352990	-2.0098560	-0.2687270
H	-6.6277920	-1.3924600	-0.3249220
H	-5.7697480	-2.6521110	0.6112150
H	-5.6343760	-2.6187720	-1.1670320
C	1.8625510	0.5031560	1.1004020
H	1.9160910	1.5092740	1.5181280
C	1.9597440	0.5779720	-0.4627650
C	2.0648110	1.9983340	-1.0021950
H	2.0041700	1.9782670	-2.0921690
H	3.0058210	2.4660920	-0.7196310
H	1.2443760	2.6085270	-0.6191800
C	3.0205110	-0.3783320	-1.0013080
H	3.0560420	-0.3278370	-2.0917670
C	4.3910890	-0.2642460	-0.2319030
C	4.9267560	1.0785510	0.2610950
H	5.2225710	1.7118080	-0.5800730
H	5.8178910	0.9066580	0.8729010
H	4.2231490	1.6430160	0.8722700
C	5.5072590	-0.9532800	-1.0265220
H	5.2156770	-1.9314820	-1.4104970
H	6.3882870	-1.0911240	-0.3926490
H	5.8004580	-0.3335080	-1.8792400
C	3.7348590	-1.2218130	0.8148520
H	4.3955900	-1.9174630	1.3369290
C	2.8316320	-1.7614650	-0.3218040
H	1.8140720	-2.0507020	-0.0597730
H	3.2897080	-2.5697060	-0.8871300
C	2.8737830	-0.4313060	1.8032300
H	3.5057730	0.1555460	2.4730200
H	2.3005920	-1.1200830	2.4280540

Conf. 2. SCF –1153.316022 hartree

Symbol	X	Y	Z
O	1.1307420	-1.3167460	0.1220160
O	1.1667370	0.9486780	0.0859630
O	-0.4308500	-0.0997390	1.3303440
O	-0.5848760	-0.1333760	-0.8949390
O	4.7594060	0.9820300	-0.1844280
O	3.2105240	2.6277240	-0.0959070
C	0.2845860	-0.1334880	0.1597960
C	2.4243480	-0.9087340	0.0178140
C	3.4796930	-1.9571090	-0.0467720
H	4.4280820	-1.4263800	-0.1347040
C	3.4918600	-2.7923250	1.2463910
H	3.6482700	-2.1597320	2.1229860
H	4.3007830	-3.5249070	1.2021480
H	2.5494010	-3.3302750	1.3725150

C	3.2848020	-2.8442500	-1.2896030
H	2.3356390	-3.3828240	-1.2397770
H	4.0920810	-3.5777230	-1.3468640
H	3.2957870	-2.2485090	-2.2050470
C	2.4644150	0.4368510	-0.0054310
C	3.5965780	1.3404540	-0.1039450
C	4.2660080	3.6077970	-0.1895480
H	3.7642030	4.5714270	-0.1710540
H	4.8178700	3.4810670	-1.1208400
H	4.9454510	3.5135800	0.6574690
C	-1.7891540	-0.5519540	1.0998370
H	-1.8672130	-1.5581540	1.5134220
C	-1.8981720	-0.6165760	-0.4630420
C	-2.0473790	-2.0306170	-1.0085180
H	-1.9944690	-2.0067960	-2.0988200
H	-2.9990370	-2.4729720	-0.7208860
H	-1.2416540	-2.6655820	-0.6346530
C	-2.9338120	0.3728840	-0.9905400
H	-2.9768440	0.3291740	-2.0810160
C	-4.3028470	0.2946780	-0.2139550
C	-4.8746360	-1.0345180	0.2752940
H	-5.1942750	-1.6542700	-0.5672220
H	-5.7564440	-0.8398110	0.8937410
H	-4.1840630	-1.6226350	0.8789960
C	-5.4028020	1.0203830	-0.9984160
H	-5.0848690	1.9917930	-1.3787380
H	-6.2758610	1.1803010	-0.3587400
H	-5.7187760	0.4142080	-1.8527690
C	-3.6130520	1.2270390	0.8341120
H	-4.2502530	1.9388020	1.3636650
C	-2.7010570	1.7463440	-0.3049940
H	-1.6740850	2.0047100	-0.0474550
H	-3.1390080	2.5703970	-0.8634000
C	-2.7700930	0.4062670	1.8132750
H	-3.4152910	-0.1666540	2.4825020
H	-2.1746120	1.0744480	2.4396180

Table S19. Cartesian coordinates of compound (2*R*,1'*R*,2'*R*,3'*S*,5'*R*)**-9e"** (most stable conformers) optimized at B3LYP-D3BJ/6-311+G(d,p)/PCM level. All structures have 0 imaginary frequencies.

Conf. 1. SCF –1153.315724 hartree

Symbol	X	Y	Z
O	0.7553030	1.0150550	0.1010910
O	1.4773580	-1.1287630	0.1139810
O	-0.3447910	-0.5771430	1.3749240
O	-0.5478010	-0.6227820	-0.8537870
C	0.3019320	-0.3552620	0.1835450
C	2.1138660	1.0168960	-0.0058920
C	2.5573180	-0.2538320	-0.0004320
C	-1.7668910	-0.7373240	1.1608530
H	-2.0388840	-1.6298740	1.7203130
C	-1.8722670	-1.0214940	-0.3683190
C	-2.0331560	-2.4984470	-0.6983450
H	-1.9205730	-2.6456140	-1.7742870
H	-3.0169060	-2.8618110	-0.4030580
H	-1.2731960	-3.0901350	-0.1829760
C	-2.9113670	-0.1261720	-1.0424250
H	-3.0145010	-0.3916770	-2.0967600
C	-4.2380130	-0.0061740	-0.1975380
C	-4.8122480	-1.1949520	0.5720060
H	-5.1983310	-1.9518230	-0.1168100
H	-5.6507220	-0.8532400	1.1867220
H	-4.1052570	-1.6849140	1.2400730
C	-5.3666710	0.5804580	-1.0549980
H	-5.0542630	1.4468600	-1.6386010
H	-6.1996400	0.8893330	-0.4164630
H	-5.7407370	-0.1749700	-1.7525180
C	-3.4708900	1.0992640	0.6004060
H	-4.0624300	1.9217470	1.0088180
C	-2.6229620	1.3518400	-0.6692690
H	-1.5813400	1.6360680	-0.5286020
H	-3.0854020	2.0510800	-1.3622190
C	-2.5706350	0.4823440	1.6766880
H	-3.1641030	0.1761010	2.5412150
H	-1.8587080	1.2330960	2.0275840
C	3.9113350	-0.7725170	-0.0922940
O	4.9094030	-0.0797840	-0.1958260
O	3.9354410	-2.1154470	-0.0489770
C	5.2387230	-2.7303300	-0.1309750
H	5.8623260	-2.4067320	0.7023170
H	5.7201520	-2.4714420	-1.0738950
H	5.0544520	-3.7998630	-0.0773630
C	2.7954360	2.3367650	-0.1018630
H	3.8612770	2.1217150	-0.1826640
C	2.3389260	3.0901050	-1.3644460
H	1.2697340	3.3104890	-1.3219330
H	2.5349310	2.5045090	-2.2654080
H	2.8804710	4.0353080	-1.4438850
C	2.5471990	3.1669490	1.1706840
H	1.4843450	3.3898300	1.2899140
H	3.0897770	4.1125320	1.1037610
H	2.8904140	2.6356280	2.0611690

Conf. 2. SCF –1153.315770 hartree

Symbol	X	Y	Z
O	0.7636370	0.8387340	0.1180040
O	1.3437710	-1.3449850	0.1272290
O	-0.4453600	-0.6814030	1.3800770
O	-0.6381330	-0.7073540	-0.8499070
O	3.8684270	-2.4485880	-0.0570010
O	4.7945590	-0.3878440	-0.1573290
C	0.2218700	-0.4984010	0.1929770
C	2.1210050	0.7541190	0.0172560
C	2.8703040	2.0387980	-0.0696390
H	3.9245320	1.7777510	-0.1522680
C	2.4505300	2.8230950	-1.3261650
H	3.0379920	3.7410530	-1.3984760

H	1.3935820	3.0954330	-1.2815280
H	2.6175450	2.2354150	-2.2316260
C	2.6617860	2.8703310	1.2093250
H	1.6110440	3.1436100	1.3302610
H	3.2497740	3.7888600	1.1494100
H	2.9785650	2.3160570	2.0956210
C	2.4795840	-0.5441450	0.0204100
C	3.7542640	-1.2371830	-0.0660520
C	6.1027170	-0.9893370	-0.2510300
H	6.7961080	-0.1551600	-0.3160880
H	6.1697930	-1.6128790	-1.1424540
H	6.3080640	-1.5889770	0.6356490
C	-1.8738890	-0.7351160	1.1573860
H	-2.2146870	-1.6049920	1.7148450
C	-1.9904360	-1.0108690	-0.3724280
C	-2.2538730	-2.4727840	-0.7034390
H	-2.1455770	-2.6282580	-1.7786520
H	-3.2626980	-2.7652370	-0.4139260
H	-1.5408520	-3.1164840	-0.1833740

C	-2.9595810	-0.0440310	-1.0521080
H	-3.0763640	-0.3022490	-2.1068380
C	-4.2783370	0.1712250	-0.2137580
C	-4.9394480	-0.9730730	0.5533500
H	-5.3737450	-1.7018650	-0.1370240
H	-5.7552400	-0.5725340	1.1630320
H	-4.2721490	-1.5102880	1.2257200
C	-5.3581460	0.8365780	-1.0766930
H	-4.9819400	1.6783420	-1.6587690
H	-6.1699670	1.2044000	-0.4421480
H	-5.7819700	0.1095140	-1.7758880
C	-3.4379360	1.2196020	0.5873650
H	-3.9710680	2.0825650	0.9926920
C	-2.5682470	1.4099730	-0.6784390
H	-1.5097740	1.6195710	-0.5328260
H	-2.9766330	2.1395140	-1.3740950
C	-2.5886100	0.5410220	1.6681720
H	-3.2056430	0.2805850	2.5312370
H	-1.8252390	1.2389570	2.0199770

Table S20. Cartesian coordinates of compound (*R*)-**10a** (most stable conformers) optimized at B3LYP-D3BJ/6-311+G(d,p)/PCM level. All structures have 0 imaginary frequencies.

Conf. 1. SCF –1088.5588721 hartree

Symbol	X	Y	Z
O	-2.8216880	-0.4126110	-1.4996490
O	-3.6281760	-0.1605390	0.5988860
O	0.4196750	-1.3643890	1.4154570
O	-0.4362010	-1.2308850	-0.6767800
O	1.0722530	-2.8707840	-0.2317120
O	2.9481530	1.1644100	-0.8379020
O	0.8656050	1.1353460	0.1014370
N	1.8924600	-0.7891090	-0.3606840
C	-4.0893870	0.0321240	-2.0274850
H	-4.3044590	1.0455660	-1.6889740
H	-4.8872470	-0.6396620	-1.7112630
H	-3.9743210	0.0067770	-3.1076610
C	-2.7226890	-0.4613050	-0.1605690
C	-1.4075940	-0.9004200	0.2671480
C	-0.8868230	-0.9847880	1.5031490
C	-1.4304600	-0.7231150	2.8506470
H	-1.3854630	-1.6302960	3.4600390
H	-2.4617380	-0.3874580	2.7759870
H	-0.8305020	0.0432500	3.3499490
C	0.7390160	-1.5547790	0.0199300
C	2.5121820	-2.9933390	-0.3118840
H	2.7341000	-3.8377430	-0.9587300
H	2.9146570	-3.1692000	0.6880000
C	2.9573640	-1.6483020	-0.8748140
H	2.9730080	-1.6403790	-1.9675920
C	1.9714680	0.5838610	-0.4004380
C	0.6663490	2.6052760	0.1509300
C	-0.7312180	2.7251520	0.7501740
H	-1.4696780	2.2481050	0.1038970
H	-0.9927070	3.7795910	0.8577430
H	-0.7708690	2.2546030	1.7340220
C	0.7009390	3.1786700	-1.2630510
H	1.6962990	3.1083400	-1.6978690
H	0.4082670	4.2305410	-1.2268170
H	-0.0081190	2.6488070	-1.9032930
C	1.7113760	3.2299630	1.0709250
H	2.7125080	3.1404500	0.6525690
H	1.6886440	2.7488790	2.0514750
H	1.4817120	4.2895660	1.2048070
H	3.9285880	-1.3343220	-0.4973710

Conf. 2. SCF –1088.5587697 hartree

Symbol	X	Y	Z
O	-3.5475700	-0.3840350	0.0593800
O	-2.6057060	-0.5394770	-1.9915170
O	0.3502560	-1.3710520	1.3307810
O	-0.1907710	-1.2601440	-0.8623040
O	1.3290940	-2.8159290	-0.2052870
O	3.0220150	1.3284850	-0.5426240
O	0.8412320	1.1681770	0.1193340
N	2.0342000	-0.6882310	-0.2031580
C	-4.7923210	0.0155940	-0.5513080
H	-5.1569070	-0.7676830	-1.2157240
H	-4.6586920	0.9418200	-1.1101780
H	-5.4808740	0.1653430	0.2759180
C	-2.5240950	-0.6269940	-0.7805280
C	-1.3086380	-0.9918690	-0.0754660
C	-0.9750250	-1.0627830	1.2253630
C	-1.7092040	-0.8534080	2.4900750
H	-1.6669960	-1.7588180	3.1020860
H	-2.7460500	-0.5992240	2.2901310
H	-1.2388900	-0.0461530	3.0591360
C	0.8842420	-1.5247030	-0.0003640
C	2.7706010	-2.8521570	-0.0805680

H	3.1322920	-3.6747880	-0.6914240
H	3.0372710	-3.0143150	0.9660240
C	3.2084550	-1.4770280	-0.5710450
H	3.3724240	-1.4545680	-1.6514060
C	2.0338730	0.6872220	-0.2351840
C	0.5458850	2.6228380	0.1264750
C	-0.9166650	2.6578230	0.5584620
H	-1.5440900	2.1296150	-0.1610380
H	-1.2540420	3.6943240	0.6188790
H	-1.0403320	2.1939730	1.5385730
C	0.7098600	3.1865300	-1.2822200
H	1.7511850	3.1707530	-1.5991470
H	0.3562360	4.2200500	-1.2951970
H	0.1103990	2.6114100	-1.9916340
C	1.4344450	3.3202530	1.1526950
H	2.4807660	3.2960400	0.8533020
H	1.3304570	2.8431200	2.1299530
H	1.1207430	4.3625040	1.2463540
H	4.0988250	-1.1119160	-0.0629300

Conf. 3. SCF –1088.5585706 hartree

Symbol	X	Y	Z
O	-3.5698630	-0.2552700	0.0609550
O	-2.6374560	-0.6223940	-1.9669580
O	0.2918100	-1.2601380	1.4250960
O	-0.2439080	-1.3166080	-0.7701460
O	1.2313880	-2.8705590	0.1178370
O	3.1337600	1.1713490	-0.3792730
O	0.8818550	1.1395170	0.0029260
N	1.9930180	-0.7842730	-0.1924360
C	-4.8019210	0.1301760	-0.5837010
H	-5.1921220	-0.6950560	-1.1791540
H	-4.6391640	1.0002770	-1.2196770
H	-5.4843400	0.3722900	0.2265280
C	-2.5564220	-0.6038630	-0.7530860
C	-1.3508930	-0.9440100	-0.0169200
C	-1.0228310	-0.9059070	1.2860570
C	-1.7429690	-0.5515380	2.5258840
H	-1.7514670	-1.4031430	3.2122200
H	-2.7643380	-0.2589950	2.3002990
H	-1.2287550	0.2734320	3.0277180
C	0.8220300	-1.5500080	0.1295970
C	2.4922830	-2.9824050	-0.5842860
H	2.2981880	-3.1426010	-1.6469840
H	3.0261040	-3.8326740	-0.1683680
C	3.1687560	-1.6414770	-0.3273080
H	3.7927600	-1.3171280	-1.1575660
C	2.0795500	0.5893810	-0.2005360
C	0.6765780	2.6094390	0.0091060
C	-0.8266150	2.7276240	0.2406160
H	-1.3789970	2.2438280	-0.5667890
H	-1.1089430	3.7817110	0.2723300
H	-1.1095130	2.2629840	1.1864440
C	1.0634120	3.1887380	-1.3489240
H	2.1352220	3.1142290	-1.5236320
H	0.7757210	4.2421250	-1.3806550
H	0.5329140	2.6658390	-2.1480340
C	1.4586300	3.2287130	1.1638840
H	2.5325190	3.1412960	1.0076140
H	1.1928290	2.7419700	2.1051000
H	1.2015430	4.2875940	1.2420520
H	3.7604950	-1.6444260	0.5918170

Conf. 4. SCF –1088.5586130 hartree

Symbol	X	Y	Z
O	-2.8543810	-0.3724120	-1.4761860
O	-3.6352440	0.1189060	0.5890740
O	0.3347100	-1.2485180	1.5230540
O	-0.5204130	-1.2612020	-0.5722600
O	0.9181680	-2.9447340	0.1197960
O	3.0993820	0.8982160	-0.7031110
O	0.9273020	1.0791410	-0.0190450
N	1.8176660	-0.9409500	-0.3331140
C	-4.0955960	0.0975390	-2.0439480
H	-4.2491470	1.1481810	-1.7980100
H	-4.9304250	-0.4948230	-1.6696630
H	-3.9864150	-0.0315060	-3.1173000
C	-2.7533560	-0.3049540	-0.1383160
C	-1.4650170	-0.7840360	0.3302190
C	-0.9467030	-0.7737700	1.5694020
C	-1.4589340	-0.3405310	2.8840520
H	-1.4657190	-1.1805970	3.5845540
H	-2.4678550	0.0501210	2.7778970
H	-0.8073230	0.4337830	3.2995980
C	0.6403480	-1.5914460	0.1681370
C	2.0500570	-3.1781410	-0.7517810
H	1.6920560	-3.3241800	-1.7731520
H	2.5567730	-4.0729980	-0.4004640
C	2.8776900	-1.9059030	-0.6172500
H	3.4043900	-1.6460910	-1.5331880
C	2.0300450	0.4186070	-0.3737760
C	0.8646290	2.5617390	0.0072730
C	-0.5794370	2.8190280	0.4261920
H	-1.2722760	2.3944830	-0.3024990
H	-0.7553200	3.8947000	0.4877970
H	-0.7836310	2.3765040	1.4024050
C	1.1303680	3.1148240	-1.3902130
H	2.1607040	2.9449180	-1.6971520
H	0.9376990	4.1901340	-1.3888570
H	0.4590330	2.6488590	-2.1152530
C	1.8413190	3.0933580	1.0522370
H	2.8738370	2.9049910	0.7625200
H	1.6503290	2.6263090	2.0211560
H	1.6995130	4.1713630	1.1585560
H	3.5914480	-1.9605480	0.2089160

Table S21. Cartesian coordinates of compound (5*R*,8*S*)- **10i’** (most stable conformers) optimized at B3LYP-D3BJ/6-311+G(d,p)/PCM level. All structures have 0 imaginary frequencies.

Conf. 1. SCF –1206.5532292 hartree

Symbol	X	Y	Z
O	-3.9512540	-1.2458990	0.1751940
O	-3.1144740	-1.1062430	-1.9217400
O	0.1295640	-1.1590380	1.2364650
O	-0.5370680	-1.1374580	-0.9242490
O	1.3914940	-2.2621860	-0.3002380
O	1.8551020	2.1874780	-0.7497170
O	-0.1305800	1.4382560	0.0902500
N	1.4860050	-0.0266090	-0.3815910
C	-5.2890310	-1.1976860	-0.3634490
H	-5.4598750	-2.0433240	-1.0294390
H	-5.4453660	-0.2641000	-0.9038470
H	-5.9465040	-1.2532000	0.4999470
C	-2.9471300	-1.1845700	-0.7191050
C	-1.6407940	-1.2087570	-0.0839600
C	-1.2372620	-1.2130140	1.1983310
C	-1.9301650	-1.2335250	2.5023890
H	-1.6320240	-2.1177690	3.0730610
H	-3.0067810	-1.2416270	2.3592450
H	-1.6457250	-0.3518860	3.0842020
C	0.6200390	-1.1350250	-0.1036930
C	2.5311990	-1.9306880	-1.1247780
H	2.2703020	-2.0776140	-2.1744870
H	3.3362520	-2.6069740	-0.8494410
C	2.8221420	-0.4577590	-0.8140440
H	3.0842330	0.0770220	-1.7277500
C	1.1113230	1.2985560	-0.3776060
C	-0.8136620	2.7551680	0.1313260
C	-2.1865980	2.3956790	0.6905880
H	-2.7044520	1.7021070	0.0265950
H	-2.7893040	3.3008620	0.7867720
H	-2.0928730	1.9341790	1.6751390
C	-0.9336220	3.3146750	-1.2835560
H	0.0402780	3.5807410	-1.6907320
H	-1.5593560	4.2098320	-1.2604430
H	-1.4089010	2.5824730	-1.9403730
C	-0.0677790	3.6906110	1.0790180
H	0.0492640	3.2219940	2.0589390
H	-0.6488460	4.6070030	1.2058650
H	0.9143400	3.9506330	0.6884390
C	3.9129130	-0.1941490	0.2413950
H	3.8800290	0.8830770	0.4321310
C	5.2928860	-0.5263670	-0.3361400
H	5.3946590	-1.5959940	-0.5444530
H	6.0775020	-0.2551320	0.3740430
H	5.4773150	0.0175720	-1.2668500
C	3.6634670	-0.9202250	1.5665020
H	2.6864530	-0.6696090	1.9840220
H	4.4247070	-0.6343250	2.2965660
H	3.7128040	-2.0065020	1.4469610

Conf. 2. SCF –1206.5520843 hartree

Symbol	X	Y	Z
O	-3.1690920	-1.0907110	-1.4578770
O	-4.0235810	-1.2019460	0.6336030
O	0.1991380	-1.2009220	1.4714850
O	-0.6536310	-1.1958260	-0.6260720
O	1.2901680	-2.3662190	-0.1505640
O	1.6568130	1.9874720	-1.0951710
O	-0.1065630	1.3893830	0.2224710
N	1.4370290	-0.1356200	-0.2881300
C	-4.5092390	-1.0113620	-1.9882360
H	-5.0153560	-0.1264050	-1.6027760
H	-5.0743540	-1.9046960	-1.7227820
H	-4.3870440	-0.9425060	-3.0657180
C	-3.0664680	-1.1799120	-0.1215720
C	-1.6805910	-1.2291530	0.3095670
C	-1.1662550	-1.2227470	1.5508350
C	-1.7650110	-1.1955820	2.8994220
H	-1.4427090	-2.0671050	3.4763110
H	-2.8494160	-1.1919910	2.8227470
H	-1.4335950	-0.3002160	3.4335910
C	0.5697270	-1.2129780	0.0937040
C	2.3125300	-2.0715350	-1.1316250
H	1.8885840	-2.1555810	-2.1345880
H	3.1036580	-2.8039080	-0.9988350
C	2.7225780	-0.6351930	-0.7990690
H	2.9871360	-0.0731760	-1.6952830
C	1.0269710	1.1729400	-0.4489490
C	-0.8681120	2.6568170	0.0920110
C	-2.0702370	2.4144190	0.9992150
H	-2.6583190	1.5660850	0.6480580
H	-2.7071490	3.3010660	1.0045800
H	-1.7445670	2.2143230	2.0218830
C	-1.3049760	2.8337440	-1.3593390
H	-0.4507290	3.0016740	-2.0138670
H	-1.9714660	3.6962680	-1.4300350
H	-1.8497090	1.9502290	-1.7000160
C	-0.0297990	3.8234610	0.6086240
H	0.3257750	3.6141050	1.6202680
H	-0.6536450	4.7196600	0.6451930
H	0.8252970	4.0163070	-0.0358050
C	3.8650230	-0.5581310	0.2343790
H	3.6399860	-1.2905680	1.0195730
C	3.9777690	0.8207770	0.8900270
H	4.1838820	1.5941370	0.1456630
H	4.7945880	0.8208190	1.6162030
H	3.0626520	1.0927880	1.4194750
C	5.1895550	-0.9455070	-0.4342510
H	5.1496870	-1.9339910	-0.8983970
H	5.9977120	-0.9582780	0.3006190
H	5.4531010	-0.2198910	-1.2106130

Conf. 3. SCF –1206.5533424 hartree

Symbol	X	Y	Z
O	-3.1549850	-1.2771660	-1.4840810
O	-3.9899820	-1.3165690	0.6177110
O	0.2292530	-1.0667700	1.4228450
O	-0.6302040	-1.2059520	-0.6670200

O	1.3731800	-2.2512570	-0.1454810
O	1.6802520	2.1605640	-0.9526730
O	-0.1962930	1.4390500	0.1283480
N	1.4067450	-0.0255920	-0.3868220
C	-4.5006790	-1.2983030	-2.0059600
H	-5.0506520	-0.4204820	-1.6669640
H	-5.0163880	-2.2033400	-1.6852860
H	-4.3887750	-1.2848660	-3.0866860
C	-3.0395810	-1.2871490	-0.1456470
C	-1.6506130	-1.2441020	0.2759040
C	-1.1323680	-1.1531100	1.5120130
C	-1.7241370	-1.0997200	2.8629940
H	-1.3598210	-1.9333770	3.4701770
H	-2.8080120	-1.1474750	2.7947050
H	-1.4292520	-0.1714300	3.3609910
C	0.5965730	-1.1267170	0.0439290
C	2.4240230	-1.9590620	-1.0936540
H	2.0716230	-2.1855310	-2.1016690
H	3.2672200	-2.5992580	-0.8480200
C	2.7061700	-0.4625910	-0.9147620
H	2.8674900	0.0105360	-1.8841670
C	0.9988170	1.2885450	-0.4455940
C	-0.9107460	2.7399120	0.1328000
C	-2.2114040	2.3973330	0.8523420
H	-2.7698870	1.6391830	0.3016160
H	-2.8294840	3.2934580	0.9347440
H	-2.0084040	2.0216500	1.8568040
C	-1.1833060	3.1780700	-1.3035320
H	-0.2610570	3.4267180	-1.8258630
H	-1.8264170	4.0610140	-1.2909310
H	-1.7013600	2.3854970	-1.8484180
C	-0.1050810	3.7662950	0.9246580
H	0.8248230	4.0180600	0.4181980
H	0.1231820	3.3807870	1.9210460
H	-0.7001230	4.6754390	1.0383870
C	3.8843830	-0.1041650	0.0107050
H	3.8416170	0.9830830	0.1293300
C	5.2122610	-0.4497190	-0.6716260
H	5.3221420	-1.5291950	-0.8150390
H	6.0526250	-0.1138290	-0.0596420
H	5.2945600	0.0307760	-1.6506220
C	3.7795560	-0.7387330	1.4005990
H	2.8399480	-0.4778570	1.8913290
H	4.5982970	-0.3866990	2.0330090
H	3.8458930	-1.8296370	1.3525110

Conf. 4. SCF –1206.5516553 hartree

Symbol	X	Y	Z
O	-3.1868670	-0.9519930	-1.5368140
O	-4.0466780	-1.2025400	0.5407610
O	0.1747020	-1.3178030	1.3716920
O	-0.6773230	-1.1782160	-0.7222410
O	1.2206760	-2.3812400	-0.4088450
O	1.6309650	2.0062490	-1.0016100
O	-0.1155100	1.3465550	0.3092280
N	1.4290220	-0.1495980	-0.2881390
C	-4.5243210	-0.8005010	-2.0577680
H	-5.0078150	0.0664770	-1.6076990
H	-5.1121830	-1.6961190	-1.8574770
H	-4.4001960	-0.6560200	-3.1275170
C	-3.0875490	-1.1440860	-0.2104900
C	-1.7052200	-1.2514550	0.2155580
C	-1.1851960	-1.3335070	1.4527210
C	-1.7873110	-1.3901690	2.7996070
H	-1.4711980	-2.2996720	3.3182220
H	-2.8715540	-1.3739390	2.7227040
H	-1.4500910	-0.5339540	3.3911690
C	0.5414310	-1.2458160	-0.0249650
C	2.6317200	-2.0999800	-0.5074700
H	3.0378130	-2.7347020	-1.2903340
H	3.1089760	-2.3379680	0.4445660
C	2.7155910	-0.6049250	-0.8375870
H	2.7146470	-0.4595490	-1.9225020
C	1.0099330	1.1618650	-0.3846690
C	-0.8902390	2.6107960	0.2288530
C	-2.0738980	2.3324240	1.1499590
H	-2.6586940	1.4858670	0.7896650
H	-2.7203220	3.2113870	1.1872220
H	-1.7282820	2.1122210	2.1619350
C	-1.3526200	2.8235270	-1.2097810
H	-0.5102550	3.0159130	-1.8731310
H	-2.0259980	3.6827930	-1.2463500
H	-1.8962300	1.9456880	-1.5662540
C	-0.0548590	3.7713100	0.7639420
H	0.3255900	3.5347710	1.7604340
H	-0.6894560	4.6571700	0.8430410
H	0.7830950	3.9963590	0.1077360
C	3.9469220	0.1071920	-0.2471070
H	3.9090730	1.1336120	-0.6170870
C	5.2240570	-0.5450220	-0.7922530
H	5.3473140	-1.5658310	-0.4173090
H	6.1017720	0.0265760	-0.4823960
H	5.2171490	-0.5838780	-1.8852680
C	3.9459930	0.1603680	1.2837140
H	3.0432400	0.6377170	1.6702540
H	4.8063820	0.7371490	1.6315770
H	4.0177430	-0.8350960	1.7308270

Conf. 5. SCF –1206.5511481 hartree

Symbol	X	Y	Z
O	-3.8415660	0.7759180	-1.4235600
O	-4.7404900	0.5892580	0.6435500
O	-0.9186270	-1.2954280	1.2480850
O	-1.5913890	-0.4641370	-0.7467460
O	-0.3629610	-2.4297650	-0.6445080
O	0.0844770	1.4388160	0.7101130
O	2.1376630	1.2094340	-0.2625790
N	0.7311480	-0.5080430	-0.2957150
C	-5.0312750	1.4633720	-1.8654220
H	-5.1698240	2.3823100	-1.2959580
H	-5.9043950	0.8216620	-1.7480620
H	-4.8597760	1.6869050	-2.9148720
C	-3.8245260	0.3911430	-0.1363860
C	-2.5851220	-0.2838030	0.2086890
C	-2.1730250	-0.7702730	1.3914600
C	-2.7758030	-0.8218110	2.7380880

H	-2.8558270	-1.8583380	3.0779200
H	-3.7638530	-0.3687240	2.7192360
H	-2.1416760	-0.2852090	3.4499750
C	-0.5278000	-1.1644100	-0.1194580
C	0.7745760	-2.4387850	-1.5355520
H	0.4406410	-2.2001890	-2.5470700
H	1.1928670	-3.4417460	-1.5150490
C	1.7154140	-1.3614240	-0.9815870
H	2.1628190	-0.7973310	-1.7998490
C	0.9211040	0.7936270	0.1082510
C	2.6618770	2.5414370	0.1290040
C	4.0628650	2.5268390	-0.4742440
H	4.6541800	1.7116680	-0.0522810
H	4.5661640	3.4710960	-0.2571670
H	4.0128490	2.4004800	-1.5576700
C	2.7251410	2.6392190	1.6512210
H	1.7295600	2.6538390	2.0912830
H	3.2429260	3.5603870	1.9279740
H	3.2856410	1.7960540	2.0617160
C	1.8077910	3.6389850	-0.5002070
H	1.7356240	3.4903590	-1.5801210
H	2.2818900	4.6066480	-0.3198330
H	0.8059830	3.6551330	-0.0751160
C	2.8387610	-1.8639320	-0.0542510
H	3.3070810	-0.9632040	0.3530150
C	3.8967040	-2.6116830	-0.8728710
H	3.4942230	-3.5316360	-1.3082470
H	4.7399110	-2.8921460	-0.2373250
H	4.2805610	-1.9923270	-1.6884880
C	2.3281080	-2.7065980	1.1179660
H	1.5896780	-2.1640200	1.7111910
H	3.1597120	-2.9706990	1.7759030
H	1.8694180	-3.6388570	0.7758630

Conf. 6. SCF –1206.5520101 hartree

Symbol	X	Y	Z
O	-3.1665220	-0.8856020	-1.4418750
O	-3.9297340	-1.2177500	0.6617350
O	0.3271400	-1.3382220	1.2984520
O	-0.6208070	-1.1143280	-0.7456800
O	1.3262560	-2.3419100	-0.4802890
O	1.5819570	2.0576500	-1.1398340
O	-0.0084480	1.3673050	0.3406220
N	1.4938540	-0.1042560	-0.4023780
C	-4.5264480	-0.7261330	-1.8985240
H	-4.9944160	0.1219840	-1.3988670
H	-5.0997810	-1.6321890	-1.7033100
H	-4.4500810	-0.5448730	-2.9671310
C	-3.0067910	-1.1246740	-0.1296850
C	-1.6039260	-1.2358880	0.2284470
C	-1.0328170	-1.3594610	1.4385280
C	-1.5683360	-1.4624470	2.8098090
H	-1.2239080	-2.3856180	3.2843130
H	-2.6550870	-1.4505470	2.7848910
H	-1.2078180	-0.6226760	3.4112890
C	0.6358400	-1.2136800	-0.0884770
C	2.3066440	-1.9700200	-1.4755220
H	1.8458170	-2.0033060	-2.4649430
H	3.1174230	-2.6925930	-1.4204010
C	2.7203070	-0.5480070	-1.0872470
H	2.8664240	0.0673220	-1.9749520
C	1.0450690	1.2031300	-0.4633860
C	-0.8277310	2.6055790	0.3185070
C	-1.9072630	2.3063850	1.3540660
H	-2.4978410	1.4374670	1.0629400
H	-2.5739390	3.1661970	1.4437720
H	-1.4580610	2.1119150	2.3300260
C	-1.4335450	2.7799300	-1.0713210
H	-0.6660420	2.9898860	-1.8154080
H	-2.1362500	3.6160040	-1.0531310
H	-1.9782760	1.8794370	-1.3634620
C	0.0172550	3.8004500	0.7536070
H	0.4967360	3.5945830	1.7135050
H	-0.6346010	4.6685170	0.8774060
H	0.7825820	4.0374860	0.0177720
C	3.9830700	-0.4727020	-0.2059000
H	4.7822380	-0.8976760	-0.8263920
C	3.8751750	-1.3022410	1.0782540
H	3.0732840	-0.9234840	1.7172280
H	4.8099050	-1.2395310	1.6406700
H	3.6760850	-2.3567100	0.8772380
C	4.3597580	0.9765470	0.1181850
H	4.4498100	1.5808360	-0.7869950
H	5.3145480	1.0062690	0.6493180
H	3.6079610	1.4433550	0.7601800

Table S22. Cartesian coordinates of compound (5*S*,8*S*)- **10i"** (most stable conformers) optimized at B3LYP-D3BJ/6-311+G(d,p)/PCM level. All structures have 0 imaginary frequencies.

Conf. 1. SCF –1206.5535473 hartree

Symbol	X	Y	Z
O	3.8401560	-1.3447260	-0.2637540
O	2.4794450	-1.5689750	-2.0572080
O	0.1841770	-0.7998130	1.7636310
O	0.2558850	-1.2902940	-0.4444530
O	-1.4494010	-2.1352930	0.7933330
O	-1.9744810	2.2745550	0.0763570
O	0.1623210	1.4715640	0.0784700
N	-1.5405200	0.0605830	0.3699850
C	4.9880400	-1.4678540	-1.1290360
H	4.9977230	-0.6604280	-1.8611990
H	4.9759040	-2.4303310	-1.6402500
H	5.8501950	-1.3961830	-0.4714390
C	2.6353120	-1.4043860	-0.8614820
C	1.5445730	-1.2374300	0.0814930
C	1.4885390	-0.9473200	1.3935740
C	2.5036500	-0.7363090	2.4462660
H	2.3943260	0.2677910	2.8666700
H	3.5038730	-0.8524840	2.0391510

H	2.3555950	-1.4533900	3.2587730
C	-0.6433130	-1.0345020	0.6006230
C	-2.7724950	-1.6989430	1.1785230
H	-2.8199990	-1.6091150	2.2653900
H	-3.4726230	-2.4591500	0.8426700
C	-2.9488460	-0.3408090	0.4887120
H	-3.4602300	0.3576990	1.1523500
C	-1.1653340	1.3693770	0.1651010
C	0.8427280	2.7699030	-0.1498850
C	2.3122650	2.3637540	-0.2033590
H	2.6184960	1.8946770	0.7328610
H	2.9294490	3.2491880	-0.3673200
H	2.4870170	1.6617910	-1.0206460
C	0.5743150	3.6998960	1.0298560
H	1.1783270	4.6032870	0.9179130
H	0.8563640	3.2139040	1.9667400
H	-0.4750950	3.9860430	1.0778460
C	0.3955340	3.3598590	-1.4847700
H	0.5350410	2.6305760	-2.2860980
H	1.0093210	4.2352780	-1.7092700
H	-0.6492460	3.6634590	-1.4565080
C	-3.6839270	-0.3652050	-0.8651720
H	-3.5887760	0.6481710	-1.2669480
C	-5.1747520	-0.6460730	-0.6484970
H	-5.3415500	-1.6527710	-0.2526420
H	-5.7164160	-0.5727170	-1.5944260
H	-5.6181920	0.0689560	0.0502760
C	-3.0647710	-1.3373430	-1.8736610
H	-2.0064350	-1.1256190	-2.0382700
H	-3.5777660	-1.2529720	-2.8348680
H	-3.1555750	-2.3755600	-1.5413840

Conf. 2. SCF –1206.5536877 hartree

Symbol	X	Y	Z
O	-2.4928250	-1.8100140	1.6276230
O	-3.9178410	-1.4480340	-0.0918730
O	-0.1422720	-0.5623970	-1.9524870
O	-0.3312660	-1.3111840	0.1776480
O	1.4584940	-1.9823180	-1.0480320
O	1.8553650	2.3208390	0.2003680
O	-0.2617900	1.4922450	-0.0052910
N	1.4821970	0.1494600	-0.3669390
C	-3.6191570	-2.0879050	2.4862420
H	-4.1836720	-2.9391600	2.1060640
H	-4.2681050	-1.2146400	2.5518770
H	-3.1890450	-2.3175140	3.4573300
C	-2.7834790	-1.4985660	0.3530660
C	-1.5875870	-1.2188170	-0.4189440
C	-1.4605180	-0.7751690	-1.6812350
C	-2.4335480	-0.4632570	-2.7471880
H	-2.3217320	0.5797750	-3.0574520
H	-3.4455130	-0.6306710	-2.3869740
H	-2.2501650	-1.0920020	-3.6232600
C	0.6218110	-0.9220810	-0.7761820
C	2.7924420	-1.4867660	-1.3020170
H	2.8991580	-1.2694000	-2.3664140
H	3.4875130	-2.2719690	-1.0169540
C	2.9026630	-0.2167570	-0.4496480

H	3.4368390	0.5614110	-0.9963880
C	1.0702360	1.4204610	-0.0338050
C	-0.9797490	2.7485060	0.3224190
C	-2.4411520	2.3163040	0.2548790
H	-2.6893080	1.9453270	-0.7406420
H	-3.0846320	3.1691760	0.4792690
H	-2.6423090	1.5278100	0.9822960
C	-0.6706720	3.8019530	-0.7374230
H	0.3734340	4.1084010	-0.6992720
H	-1.2988880	4.6787670	-0.5643980
H	-0.8936000	3.4128440	-1.7336020
C	-0.6135760	3.1998360	1.7339220
H	-0.7800040	2.3876470	2.4453870
H	-1.2549800	4.0378340	2.0164660
H	0.4251910	3.5189180	1.7936930
C	3.5603170	-0.3897610	0.9329040
H	3.4245090	0.5692160	1.4422310
C	5.0657950	-0.6267950	0.7727460
H	5.2729710	-1.5801610	0.2766270
H	5.5521230	-0.6545610	1.7506340
H	5.5344440	0.1679470	0.1854560
C	2.9022730	-1.4786580	1.7854690
H	1.8330830	-1.2978640	1.9126440
H	3.3596040	-1.5012710	2.7777060
H	3.0281820	-2.4703510	1.3413830

Conf. 3. SCF –1206.5515179 hartree

Symbol	X	Y	Z
O	3.5225840	-0.0395510	1.8106070
O	4.6209160	-1.1060350	0.1448700
O	1.1076920	0.2600270	-1.9326450
O	1.5074790	0.6980040	0.2532920
O	0.5187640	2.3088520	-1.0085770
O	-0.2294920	-1.6554390	-0.1650900
O	-2.3848680	-0.9061220	-0.0758620
N	-0.7554360	0.5442560	-0.4872200
C	4.5807860	-0.4003990	2.7233600
H	5.5286510	0.0212090	2.3889440
H	4.6645000	-1.4847170	2.7953180
H	4.2903870	0.0242390	3.6804950
C	3.6671120	-0.4577980	0.5416040
C	2.5525300	-0.0459350	-0.2918740
C	2.3012140	-0.3006130	-1.5876540
C	3.0301670	-1.0471770	-2.6328250
H	3.9542250	-1.4497340	-2.2254760
H	3.2586890	-0.3897460	-3.4765660
H	2.4083860	-1.8659260	-3.0065400
C	0.5856130	0.9524130	-0.7754510
C	-0.8411530	2.6754020	-1.3330380
H	-0.9862100	2.6021000	-2.4124790
H	-0.9825750	3.7044600	-1.0134340
C	-1.7089700	1.6627120	-0.5753740
H	-2.5556650	1.3590510	-1.1908600
C	-1.0627260	-0.7721500	-0.2305260
C	-3.0035550	-2.2051080	0.2878380
C	-4.4816240	-1.8409180	0.3860940
H	-4.6388120	-1.0792770	1.1525500
H	-5.0618290	-2.7269060	0.6512260

H	-4.8470910	-1.4586490	-0.5692200
C	-2.4657640	-2.6698090	1.6388740
H	-1.4116960	-2.9348250	1.5781170
H	-3.0289650	-3.5479380	1.9629780
H	-2.5951170	-1.8853500	2.3881690
C	-2.7635870	-3.2213380	-0.8255300
H	-3.1005440	-2.8199370	-1.7841040
H	-3.3392310	-4.1252250	-0.6127210
H	-1.7106730	-3.4869310	-0.9005480
C	-2.2342160	2.1232500	0.7980910
H	-2.6992050	1.2393510	1.2440740
C	-3.3256460	3.1829220	0.6129640
H	-2.9248760	4.1032770	0.1769110
H	-3.7684180	3.4437520	1.5770850
H	-4.1260010	2.8211630	-0.0387780
C	-1.1284190	2.6063340	1.7409800
H	-0.3616100	1.8427670	1.8853410
H	-1.5525880	2.8457970	2.7192180
H	-0.6408380	3.5095820	1.3628860

Conf. 4. SCF –1206.5521254 hartree

Symbol	X	Y	Z
O	-2.6096720	-1.7958400	1.6860420
O	-4.0411780	-1.2438370	0.0236240
O	-0.2567200	-0.5506310	-1.8961740
O	-0.4477410	-1.3679450	0.2086910
O	1.2475850	-2.1288050	-1.1027740
O	2.0377830	2.1505540	-0.0345320
O	-0.1372030	1.4656920	0.0352700
N	1.4654740	-0.0368340	-0.3369220
C	-3.7347330	-2.0399090	2.5566680
H	-4.3649110	-2.8294700	2.1475960
H	-4.3197070	-1.1288280	2.6820360
H	-3.3017310	-2.3488990	3.5041690
C	-2.9043570	-1.3979150	0.4370530
C	-1.7084980	-1.1652290	-0.3513570
C	-1.5802120	-0.6824820	-1.5990780
C	-2.5527850	-0.2697950	-2.6305890
H	-2.3758270	0.7719000	-2.9133990
H	-3.5656230	-0.3783840	-2.2508810
H	-2.4332110	-0.8823020	-3.5289430
C	0.5057220	-1.0172170	-0.7589030
C	2.5930310	-1.7001900	-1.4223830
H	2.6321530	-1.3691010	-2.4623620
H	3.2441740	-2.5575390	-1.2790540
C	2.8395380	-0.5514020	-0.4417990
H	3.4746650	0.2220270	-0.8748620
C	1.1787050	1.2933940	-0.1081570
C	-0.7412310	2.8053460	0.2379660
C	-2.2282280	2.4803230	0.3405700
H	-2.5880000	2.0160540	-0.5785350
H	-2.7920410	3.3996590	0.5102620
H	-2.4148890	1.7983140	1.1723040
C	-0.4541020	3.6837970	-0.9763060
H	0.6065680	3.9169280	-1.0555510
H	-1.0107600	4.6188620	-0.8809140
H	-0.7800670	3.1838400	-1.8912520
C	-0.2281420	3.4097050	1.5425000
H	-0.3875830	2.7140530	2.3696470
H	-0.7860100	4.3249580	1.7536840
H	0.8310020	3.6516320	1.4797400
C	3.4222810	-1.0314560	0.9039690
H	2.8736710	-1.9388450	1.1854450
C	3.2511730	-0.0077180	2.0293340
H	3.7644020	0.9274660	1.7923040
H	3.6735120	-0.4035480	2.9564690
H	2.1993210	0.2187710	2.2136600
C	4.9026320	-1.3888090	0.7229540
H	5.0606140	-2.1304440	-0.0639860
H	5.3112980	-1.7985080	1.6494980
H	5.4830830	-0.4969120	0.4655510

Conf. 5. SCF –1206.5519104 hartree

Symbol	X	Y	Z
O	2.6262640	-1.7205840	-1.5853590
O	4.0295230	-0.9821000	0.0282060
O	0.2182810	-0.4141970	1.9365180
O	0.4514390	-1.3473340	-0.1155860
O	-1.1842890	-2.1296960	1.2549110
O	-2.2154010	2.0553940	0.0446910
O	-0.0212360	1.4571920	-0.1196010
N	-1.5387620	-0.1034810	0.3598800
C	3.7588720	-1.9410090	-2.4523150
H	4.4406860	-2.6653120	-2.0068890
H	4.2838860	-1.0031680	-2.6334460
H	3.3403740	-2.3289540	-3.3770290
C	2.9023810	-1.2326710	-0.3642570
C	1.6987140	-1.0312660	0.4206170
C	1.5457790	-0.4812660	1.6374250
C	2.4953810	0.0532560	2.6340090
H	2.2548090	1.0968970	2.8561400
H	3.5113620	-0.0145060	2.2531980
H	2.4188740	-0.5104330	3.5683180
C	-0.5191210	-0.9984060	0.8346900
C	-2.5542960	-1.7793940	1.5576010
H	-2.6184610	-1.4339270	2.5915450
H	-3.1557410	-2.6758640	1.4283250
C	-2.8858090	-0.6647850	0.5615160
H	-3.5171120	0.0938620	1.0243570
C	-1.3204820	1.2346170	0.0923300
C	0.5151570	2.8164370	-0.3752340
C	2.0088940	2.5531720	-0.5401120
H	2.4326530	2.1315380	0.3721580
H	2.5223670	3.4909260	-0.7610010
H	2.1866610	1.8564830	-1.3616210
C	0.2460240	3.7089690	0.8329750
H	-0.8181940	3.9055720	0.9526730
H	0.7638790	4.6610650	0.6961340
H	0.6273870	3.2402970	1.7430200
C	-0.0822380	3.3699260	-1.6664360
H	-1.1482840	3.5616780	-1.5611810
H	0.0752940	2.6666800	-2.4875120
H	0.4202500	4.3067760	-1.9179570
C	-3.5579000	-1.1627940	-0.7347210
H	-4.4967520	-1.6218630	-0.3995760

C	-2.7373500	-2.2268950	-1.4722880
H	-1.7794360	-1.8168440	-1.8028820
H	-3.2803250	-2.5654720	-2.3580320
H	-2.5327900	-3.1006310	-0.8507060
C	-3.9126630	-0.0083700	-1.6773080
H	-4.4998630	0.7595960	-1.1696010
H	-4.4927350	-0.3834590	-2.5244850
H	-3.0121610	0.4668910	-2.0743040

Conf. 6. SCF –1206.5510073 hartree

Symbol	X	Y	Z
O	-2.6209650	-1.7991370	1.6338060
O	-4.0693910	-1.1693150	0.0146620
O	-0.3068510	-0.4868690	-1.9535210
O	-0.4769490	-1.3554060	0.1303010
O	1.1751140	-2.0872610	-1.3228980
O	2.0585680	2.0994820	-0.0136520
O	-0.1266460	1.4504940	0.0502100
N	1.4510820	-0.0662010	-0.3852670
C	-3.7355630	-2.0517510	2.5153600
H	-4.3861090	-2.8171180	2.0924210
H	-4.3024490	-1.1353560	2.6791860
H	-3.2925290	-2.3979460	3.4451920
C	-2.9289900	-1.3563050	0.4034620
C	-1.7408370	-1.1185120	-0.3971980
C	-1.6306190	-0.5974560	-1.6305270
C	-2.6102070	-0.1272410	-2.6294850
H	-2.4141140	0.9194650	-2.8795250
H	-3.6183190	-0.2257100	-2.2346950
H	-2.5219820	-0.7104780	-3.5506840
C	0.4686990	-0.9954060	-0.8641870
C	2.5181110	-2.0658680	-0.8002730
H	3.1704410	-2.5003980	-1.5530170
H	2.5530460	-2.6687900	0.1084160
C	2.8202930	-0.5877480	-0.5220810
H	3.2946230	-0.1289480	-1.3953500
C	1.1844130	1.2588490	-0.1095580
C	-0.7013010	2.7907520	0.3244070
C	-2.1938520	2.4918230	0.4255290
H	-2.5721270	2.0830770	-0.5123550
H	-2.7360480	3.4131060	0.6472100
H	-2.3877510	1.7727390	1.2236510
C	-0.4078370	3.7233780	-0.8472420
H	-0.7520990	3.2764250	-1.7826590
H	0.6562310	3.9403180	-0.9256510
H	-0.9456900	4.6626410	-0.6991680
C	-0.1639560	3.3183070	1.6522890
H	-0.3320290	2.5861810	2.4455460
H	-0.6992470	4.2340180	1.9136900
H	0.8997360	3.5400490	1.5915000
C	3.7214640	-0.3547620	0.7061490
H	3.9191200	0.7178080	0.7396450
C	5.0598810	-1.0761240	0.4997510
H	4.9393980	-2.1638510	0.5081470
H	5.7548510	-0.8172400	1.3017230
H	5.5233290	-0.7936470	-0.4498560
C	3.0613440	-0.7505990	2.0303360
H	2.1213920	-0.2178510	2.1878110
H	3.7285570	-0.5062040	2.8604450
H	2.8529000	-1.8228230	2.0835870

Table S23. Cartesian coordinates of compound (5*R*,8*S*)-**10j'** (most stable conformers) optimized at B3LYP-D3BJ/6-311+G(d,p)/PCM level. All structures have 0 imaginary frequencies.

Conf. 1. SCF –1285.2130460 hartree

Symbol	X	Y	Z
O	-3.8692180	-0.7838290	-0.5797170
O	-2.8969340	-0.0467420	-2.4846230
O	0.1353130	-1.2883790	0.6358630
O	-0.3922450	-0.6016760	-1.4511760
O	1.4188140	-2.0027840	-1.1011670
O	2.2190080	2.3202320	-0.1536700
O	0.0908940	1.5711740	0.1894360
N	1.6597280	0.1471560	-0.5195520
C	-5.1651650	-0.4679410	-1.1292290
H	-5.3436080	-1.0472530	-2.0350400
H	-5.2324210	0.5968120	-1.3524560
H	-5.8776470	-0.7412160	-0.3555470
C	-2.8084550	-0.5092720	-1.3622060
C	-1.5489530	-0.8180650	-0.7099500
C	-1.2265230	-1.2212310	0.5320800
C	-2.0100590	-1.6038340	1.7404670
H	-3.0300660	-1.2609760	1.5687590
C	0.7086140	-0.9213490	-0.6200930
C	2.6602600	-1.5538200	-1.6891550
H	2.5068140	-1.3687780	-2.7539010
H	3.3870220	-2.3511730	-1.5568420
C	3.0072060	-0.2657410	-0.9341320
H	3.4042110	0.4811740	-1.6226000
C	1.3742280	1.4434970	-0.1532320
C	-0.5040830	2.8881770	0.5284110
C	-1.9795510	2.5472680	0.7126440
H	-2.4043680	2.1662570	-0.2174110
H	-2.5270680	3.4462620	1.0021140
H	-2.1113480	1.7942910	1.4903570
C	-0.3230210	3.8595620	-0.6355310
H	0.7201910	4.1395370	-0.7664660
H	-0.9060450	4.7615860	-0.4361210
H	-0.6931830	3.4126490	-1.5611800
C	0.1109370	3.3979280	1.8284420
H	1.1677560	3.6271360	1.7006500
H	0.0003420	2.6521210	2.6189390
H	-0.4091960	4.3066900	2.1401070
C	3.9875540	-0.4183830	0.2447840
H	3.9990160	0.5564140	0.7419350
C	5.4005820	-0.6929820	-0.2813200
H	5.4609430	-1.6615590	-0.7874710

H	6.1164710	-0.7099880	0.5437780
H	5.7195650	0.0776300	-0.9888190
C	3.5501770	-1.4701170	1.2685490
H	2.5533390	-1.2604590	1.6604760
H	4.2472480	-1.4816170	2.1101250
H	3.5396780	-2.4746320	0.8354460
C	-1.4521170	-0.9239770	3.0015800
H	-2.0817590	-1.1737660	3.8580260
H	-1.4329850	0.1622260	2.8963620
H	-0.4360590	-1.2636000	3.2125180
C	-2.0299560	-3.1375030	1.8960670
H	-2.6275560	-3.4113040	2.7686540
H	-1.0185670	-3.5263160	2.0383780
H	-2.4639050	-3.6155060	1.0154840

H	6.1306500	-0.3663300	0.1541350
H	5.5685390	0.4755480	-1.2956300
C	3.6821510	-1.3383570	1.0169310
H	2.7061090	-1.2206370	1.4908610
H	4.4399360	-1.3466030	1.8043210
H	3.7052760	-2.3163090	0.5271550
C	-1.2291470	-1.2376550	3.1356540
H	-1.7854840	-1.5757570	4.0122980
H	-1.2916120	-0.1489620	3.0897030
H	-0.1807590	-1.5132180	3.2679270
C	-1.7224830	-3.4276020	1.9445550
H	-2.2482380	-3.7872710	2.8320810
H	-0.6806120	-3.7512750	2.0092960
H	-2.1742700	-3.8891410	1.0641480

Conf. 2. SCF –1285.2130284 hartree

Symbol	X	Y	Z
O	-2.9786590	-0.0971490	-2.1845880
O	-3.9398150	-1.0163410	-0.3544530
O	0.2284880	-1.3635630	0.6457150
O	-0.5066810	-0.5956050	-1.3513220
O	1.4147670	-1.8844360	-1.2243980
O	2.0017030	2.4253530	-0.0851970
O	-0.0425080	1.5166090	0.3645440
N	1.5594510	0.2402020	-0.5291560
C	-4.2903110	0.1960510	-2.7102790
H	-4.8256620	0.8651430	-2.0366970
H	-4.8586690	-0.7241950	-2.8446000
H	-4.1138830	0.6792950	-3.6674120
C	-2.9441330	-0.7084080	-0.9879170
C	-1.5837700	-0.9397310	-0.5398190
C	-1.1374440	-1.3907800	0.6450020
C	-1.8147170	-1.8904260	1.8729120
H	-2.8645540	-1.6113690	1.7731050
C	0.6757640	-0.8846110	-0.6252740
C	2.5764090	-1.3179020	-1.8709600
H	2.3307130	-1.0832150	-2.9083270
H	3.3612070	-2.0693610	-1.8415470
C	2.8955290	-0.0542190	-1.0645500
H	3.1892180	0.7565220	-1.7323010
C	1.2176510	1.4939820	-0.0733190
C	-0.6937070	2.7713710	0.8172380
C	-2.1272780	2.3224180	1.0824510
H	-2.5936720	1.9647750	0.1630350
H	-2.7085800	3.1650820	1.4615690
H	-2.1541740	1.5208010	1.8213740
C	-0.6594900	3.8121240	-0.2994990
H	0.3512600	4.1696870	-0.4846640
H	-1.2844550	4.6602570	-0.0104880
H	-1.0646590	3.3895800	-1.2219360
C	-0.0193480	3.2531390	2.0983690
H	1.0096170	3.5566060	1.9113050
H	-0.0272580	2.4625220	2.8520770
H	-0.5699150	4.1093970	2.4947730
C	3.9708920	-0.2044410	0.0286550
H	3.9579250	0.7399970	0.5814020
C	5.3541610	-0.3526640	-0.6142810
H	5.4371280	-1.2856640	-1.1804910

Conf. 3. SCF –1285.2117395 hartree

Symbol	X	Y	Z
O	-2.9730740	0.1543570	-2.0887050
O	-3.9945490	-0.8409390	-0.3327300
O	0.1528690	-1.5044670	0.6266690
O	-0.5407430	-0.5165460	-1.2870890
O	1.2783890	-1.9537970	-1.2974010
O	2.0325670	2.3260610	-0.2930280
O	0.1011250	1.4458000	0.5394580
N	1.5859250	0.0956510	-0.4431790
C	-4.2626600	0.5608340	-2.5932230
H	-4.7608740	1.2108090	-1.8740320
H	-4.8832620	-0.3125130	-2.7930600
H	-4.0532440	1.0996410	-3.5132830
C	-2.9799390	-0.5433850	-0.9403300
C	-1.6352080	-0.8796170	-0.5086580
C	-1.2141400	-1.4626290	0.6271450
C	-1.9186890	-2.0413300	1.8032170
H	-2.9681990	-1.7669540	1.6900510
C	0.6210150	-0.9536470	-0.6041720
C	2.4205200	-1.3808270	-1.9758130
H	2.1067800	-0.9815790	-2.9428330
H	3.1419290	-2.1802300	-2.1193340
C	2.8851220	-0.2781880	-1.0231010
H	3.2874070	0.5791150	-1.5631510
C	1.2822650	1.3929400	-0.0814380
C	-0.5739970	2.7412100	0.8114710
C	-1.8867300	2.3065960	1.4542110
H	-2.4587030	1.6687810	0.7799660
H	-2.4847610	3.1886390	1.6910680
H	-1.6989390	1.7584140	2.3787620
C	-0.8284410	3.4600410	-0.5106560
H	0.1038490	3.7668670	-0.9829150
H	-1.4312430	4.3514270	-0.3230190
H	-1.3800010	2.8098170	-1.1934860
C	0.2554280	3.5679650	1.7908500
H	1.1840080	3.9093050	1.3386510
H	0.4876330	2.9774240	2.6802430
H	-0.3273350	4.4380860	2.1023370
C	3.9022100	-0.7707930	0.0261710
H	3.5379730	-1.7347440	0.4026420
C	4.0298570	0.1863960	1.2144440
H	4.3713090	1.1725780	0.8890940

H	4.7555650	-0.2054560	1.9315380
H	3.0795060	0.3103950	1.7371850
C	5.2662220	-0.9871490	-0.6399910
H	5.2136900	-1.6800430	-1.4833290
H	5.9805420	-1.3953200	0.0786870
H	5.6669950	-0.0375740	-1.0093250
C	-1.3786070	-1.4536010	3.1175650
H	-1.9462780	-1.8576250	3.9584480
H	-1.4698230	-0.3664090	3.1327670
H	-0.3268920	-1.7118300	3.2589140
C	-1.8071400	-3.5784610	1.7886920
H	-2.3583390	-3.9951790	2.6347380
H	-0.7640100	-3.8933810	1.8713320
H	-2.2219430	-3.9939010	0.8678970

H	4.4950420	-0.3482940	2.2438950
H	2.8935390	0.2619970	1.8256100
C	5.2441500	-1.3086100	-0.2056940
H	5.2402980	-2.0453400	-1.0128320
H	5.8425990	-1.7171910	0.6117790
H	5.7479280	-0.4090240	-0.5739380
C	-1.4637510	-0.9942840	3.0199170
H	-2.0787550	-1.2559460	3.8834970
H	-1.4553900	0.0928240	2.9219620
H	-0.4420430	-1.3276530	3.2131740
C	-2.0469580	-3.2023910	1.9038450
H	-2.6370160	-3.4854500	2.7785370
H	-1.0332740	-3.5886990	2.0353750
H	-2.4859320	-3.6753880	1.0229030

Conf. 4. SCF –1285.2117388 hartree

Symbol	X	Y	Z
O	-3.9167910	-0.6621700	-0.4742430
O	-2.9567840	0.1023400	-2.3741930
O	0.0934000	-1.4003970	0.6105540
O	-0.4569760	-0.5905730	-1.4261740
O	1.2946230	-2.0855740	-1.2008430
O	2.2707690	2.2054720	-0.3616410
O	0.1654340	1.5261750	0.1912660
N	1.6523300	0.0189810	-0.5203490
C	-5.2099300	-0.2721660	-0.9816430
H	-5.4309550	-0.8067190	-1.9053620
H	-5.2373900	0.8026070	-1.1610310
H	-5.9166590	-0.5471930	-0.2033530
C	-2.8632740	-0.4053940	-1.2719840
C	-1.6029870	-0.7928780	-0.6641290
C	-1.2669160	-1.2696220	0.5479310
C	-2.0348070	-1.6679020	1.7606730
H	-3.0572840	-1.3256840	1.6043600
C	0.6471020	-0.9976180	-0.6434480
C	2.5286760	-1.6399180	-1.8127050
H	2.3291860	-1.3066550	-2.8333780
H	3.2063250	-2.4889780	-1.8226680
C	2.9752170	-0.4906960	-0.9088260
H	3.5007010	0.2854490	-1.4654330
C	1.4166860	1.3482910	-0.2383220
C	-0.3835540	2.8802030	0.4552870
C	-1.8426600	2.5889370	0.7909270
H	-2.3537330	2.1436040	-0.0637460
H	-2.3485770	3.5209470	1.0501740
H	-1.9165980	1.9062290	1.6383020
C	-0.2858670	3.7390160	-0.8031460
H	0.7468250	3.9842810	-1.0427320
H	-0.8379730	4.6675280	-0.6406590
H	-0.7369040	3.2184920	-1.6511350
C	0.3416330	3.4911570	1.6509850
H	1.3879920	3.6846790	1.4209130
H	0.2827560	2.8207200	2.5115460
H	-0.1391500	4.4349220	1.9187340
C	3.8310130	-0.9693120	0.2834030
H	3.3642520	-1.8846890	0.6676670
C	3.8867370	0.0473320	1.4265260
H	4.3314070	0.9886880	1.0948950

Conf. 5. SCF –1285.2110551 hartree

Symbol	X	Y	Z
O	-4.4122830	0.9395670	-0.4003320
O	-3.3046570	1.2251830	-2.3511070
O	-0.8959280	-1.1483000	0.6593370
O	-1.1544710	-0.2096550	-1.3790340
O	-0.0939560	-2.2602150	-1.1557000
O	0.3613670	1.5272220	0.4135660
O	2.5395540	1.1657140	-0.1686340
N	1.0457130	-0.4344300	-0.5374630
C	-5.5193630	1.6997990	-0.9272350
H	-5.9309510	1.2082020	-1.8087110
H	-5.1977850	2.7091980	-1.1834710
H	-6.2543670	1.7254200	-0.1272080
C	-3.3579930	0.7761780	-1.2213370
C	-2.2906640	0.0044590	-0.6062660
C	-2.1253090	-0.5482970	0.6088410
C	-2.9551440	-0.6255650	1.8426260
H	-3.8876390	-0.1085610	1.6197360
C	-0.2656820	-1.0026140	-0.6113490
C	1.1850510	-2.3284220	-1.8241280
H	1.0614340	-2.0343510	-2.8681270
H	1.5227160	-3.3602190	-1.7718880
C	2.0787250	-1.3428480	-1.0613820
H	2.7118620	-0.7937510	-1.7581970
C	1.2479830	0.8358470	-0.0489710
C	3.0735870	2.4411600	0.3692320
C	4.5591050	2.3335160	0.0397780
H	4.9978530	1.4604190	0.5270950
H	5.0790810	3.2268210	0.3912850
H	4.7080190	2.2459900	-1.0383850
C	2.8531710	2.4924460	1.8790920
H	1.7954320	2.5688830	2.1247640
H	3.3703880	3.3647680	2.2851860
H	3.2669290	1.5990180	2.3525750
C	2.4354660	3.6190070	-0.3624890
H	1.3755100	3.7033080	-0.1301490
H	2.5568400	3.5048330	-1.4421880
H	2.9364170	4.5410630	-0.0583150
C	2.9681430	-1.9552190	0.0380260
H	3.4126360	-1.1043490	0.5623540
C	4.1072620	-2.7587310	-0.5985840
H	3.7313080	-3.6331880	-1.1387790

H	4.7934980	-3.1197360	0.1710770
H	4.6805380	-2.1483390	-1.3021130
C	2.1878230	-2.7883520	1.0590550
H	1.3887930	-2.2085410	1.5247220
H	2.8596990	-3.1309460	1.8499270
H	1.7399410	-3.6745060	0.5999690
C	-2.2539720	0.0945700	3.0092720
H	-2.8886970	0.0556990	3.8973820
H	-2.0621040	1.1416900	2.7671200
H	-1.3013870	-0.3840540	3.2474140
C	-3.2698040	-2.0912600	2.1932260
H	-3.9162190	-2.1271980	3.0729700
H	-2.3548920	-2.6441890	2.4180090
H	-3.7821610	-2.5925270	1.3689780

Conf. 6. SCF –1285.2110504 hartree

Symbol	X	Y	Z
O	-3.3534690	1.4889760	-1.8801840
O	-4.4986690	1.0296710	0.0151140
O	-0.9115760	-1.2740090	0.6389040
O	-1.2962250	-0.0515700	-1.2248980
O	-0.2300690	-2.1072620	-1.3654610
O	0.3348580	1.4152880	0.7027210
O	2.4702400	1.1584570	-0.0646640
N	0.9525490	-0.3830870	-0.5637250
C	-4.4304760	2.3494630	-2.3072530
H	-4.5670720	3.1647600	-1.5968890
H	-5.3557410	1.7806100	-2.3978870
H	-4.1225300	2.7346650	-3.2755450
C	-3.5142360	0.8857630	-0.6899620
C	-2.3751770	0.0496970	-0.3519310
C	-2.1336610	-0.6712970	0.7563150
C	-2.8928010	-0.9062500	2.0138120
H	-3.8234750	-0.3471480	1.9118440
C	-0.3624520	-0.9427880	-0.6359100
C	1.0053490	-2.0726560	-2.1140320
H	0.8186740	-1.6328420	-3.0955380
H	1.3415000	-3.0996370	-2.2307490
C	1.9480350	-1.2018480	-1.2746150
H	2.5390800	-0.5556530	-1.9234560
C	1.1886560	0.8049240	0.0890920
C	3.0398870	2.3486600	0.6142310
C	4.5006480	2.3002620	0.1774150
H	4.9690790	1.3706750	0.5071220
H	5.0437050	3.1391220	0.6170580
H	4.5786940	2.3667440	-0.9096980
C	2.9179540	2.1832180	2.1270470
H	1.8787860	2.2200750	2.4491670
H	3.4652580	2.9906240	2.6188940
H	3.3566020	1.2330050	2.4405370
C	2.3573460	3.6136500	0.1004370
H	1.3151380	3.6563450	0.4112480
H	2.4072410	3.6547100	-0.9901350
H	2.8789300	4.4870190	0.4990770
C	2.9008160	-1.9604000	-0.3302670
H	3.3807940	-1.1904870	0.2808270
C	3.9945280	-2.6615980	-1.1430850
H	3.5820420	-3.4552420	-1.7738580
H	4.7270620	-3.1217630	-0.4758110
H	4.5240270	-1.9567860	-1.7904360
C	2.1810010	-2.9331760	0.6083830
H	1.4159870	-2.4286100	1.2013630
H	2.8989560	-3.3834030	1.2983780
H	1.7002680	-3.7457980	0.0559840
C	-2.1123410	-0.3626170	3.2247970
H	-2.7000100	-0.5069340	4.1341570
H	-1.9042560	0.7032500	3.1128460
H	-1.1619790	-0.8880570	3.3438000
C	-3.2223100	-2.4008120	2.1801660
H	-3.8184000	-2.5473900	3.0836490
H	-2.3092770	-2.9936190	2.2727460
H	-3.7922360	-2.7762180	1.3272960

Table S24. Cartesian coordinates of compound (5*S*,8*S*)-**10j”** (most stable conformers) optimized at B3LYP-D3BJ/6-311+G(d,p)/PCM level. All structures have 0 imaginary frequencies.

Conf. 1. SCF –1285.2133966 hartree

Symbol	X	Y	Z
O	-2.0554650	-0.6854680	2.7048280
O	-3.7303200	-0.8774600	1.1962790
O	-0.2781530	-1.0886600	-1.3628910
O	-0.1449690	-0.9985150	0.8963230
O	1.3755360	-2.2566110	-0.2267190
O	2.2000710	2.1398070	-0.7050880
O	0.0501030	1.5354230	-0.2342070
N	1.6231020	-0.0390670	-0.3989190
C	-3.0353260	-0.5131450	3.7501790
H	-3.6784670	-1.3907360	3.8144770
H	-3.6397120	0.3734700	3.5585880
H	-2.4605410	-0.3928320	4.6644570
C	-2.5402590	-0.8575730	1.4627570
C	-1.4786910	-1.0004060	0.4853340
C	-1.5427070	-1.0606530	-0.8565340
C	-2.6763280	-1.1120390	-1.8208260
H	-3.5752440	-0.8968360	-1.2418920
C	0.6518590	-1.0839040	-0.2533920
C	2.6556200	-2.0380110	-0.8605950
H	2.5659120	-2.2319900	-1.9311160
H	3.3579400	-2.7398240	-0.4188980
C	2.9830890	-0.5668360	-0.5754500
H	3.4331200	-0.1017260	-1.4535630
C	1.3439430	1.3074030	-0.4677540
C	-0.5079580	2.9079700	-0.1565720
C	-1.9588400	2.6493340	0.2380620
H	-2.4578310	2.0219960	-0.5012290
H	-2.4931770	3.5987290	0.3073540

H	-2.0085110	2.1503700	1.2076900
C	-0.4160910	3.5696430	-1.5283310
H	0.6206050	3.7437010	-1.8126940
H	-0.9370860	4.5294400	-1.4999920
H	-0.8930800	2.9437420	-2.2856920
C	0.2090920	3.7016510	0.9329850
H	0.1960440	3.1477070	1.8745530
H	-0.3174800	4.6464260	1.0862070
H	1.2401540	3.9165360	0.6597630
C	3.8893490	-0.3065000	0.6432310
H	3.8994130	0.7798600	0.7729030
C	5.3224810	-0.7556990	0.3390680
H	5.3856410	-1.8404110	0.2073330
H	5.9873880	-0.4872310	1.1633260
H	5.7045240	-0.2825270	-0.5699710
C	3.3595250	-0.9352780	1.9353000
H	2.3440760	-0.6007150	2.1571420
H	3.9977850	-0.6523890	2.7759930
H	3.3522040	-2.0276650	1.8793060
C	-2.5162450	-0.0552730	-2.9259470
H	-2.4501570	0.9513150	-2.5096960
H	-3.3794280	-0.0920660	-3.5937880
H	-1.6170440	-0.2400010	-3.5174450
C	-2.8024920	-2.5284370	-2.4156240
H	-2.9481330	-3.2727410	-1.6299580
H	-1.9061780	-2.7935470	-2.9818020
H	-3.6590030	-2.5681920	-3.0924560

Conf. 2. SCF –1285.2133344 hartree

Symbol	X	Y	Z
O	-3.6506460	-0.7861160	1.2029820
O	-2.0910570	-0.6455130	2.8355760
O	-0.2448540	-1.0697340	-1.2686390
O	-0.0726250	-0.9860750	0.9853690
O	1.3962760	-2.2819550	-0.1621840
O	2.3270680	2.0914220	-0.6560020
O	0.1664910	1.5428830	-0.1653240
N	1.6985040	-0.0710930	-0.3360280
C	-4.6856790	-0.5825900	2.1870900
H	-4.5611410	0.3854810	2.6723330
H	-4.6598710	-1.3760360	2.9338530
H	-5.6185860	-0.6128370	1.6305840
C	-2.3848260	-0.7823320	1.6622380
C	-1.4121400	-0.9486860	0.5987650
C	-1.5013370	-1.0043860	-0.7428380
C	-2.6394850	-1.0286440	-1.7051620
H	-3.5311490	-0.7654030	-1.1370480
C	0.7023490	-1.0908430	-0.1761450
C	2.6723200	-2.0954560	-0.8140070
H	2.5624330	-2.2857300	-1.8833170
H	3.3626380	-2.8156090	-0.3829640
C	3.0416560	-0.6336480	-0.5324390
H	3.4905980	-0.1791910	-1.4166990
C	1.4524720	1.2815450	-0.4080560
C	-0.3565410	2.9293780	-0.0911400
C	-1.8131470	2.7093870	0.3061910
H	-2.3303230	2.0968460	-0.4330620
H	-2.3213170	3.6729350	0.3773490

H	-1.8745940	2.2109810	1.2753520
C	-0.2508630	3.5853880	-1.4647440
H	0.7890690	3.7342150	-1.7512700
H	-0.7491800	4.5572010	-1.4378790
H	-0.7437750	2.9692540	-2.2199460
C	0.3829760	3.7068070	0.9950610
H	0.3543120	3.1573350	1.9388740
H	-0.1158860	4.6671040	1.1445270
H	1.4197530	3.8907620	0.7205880
C	3.9724060	-0.3982090	0.6727000
H	4.0118080	0.6873680	0.8035190
C	5.3888980	-0.8831010	0.3460480
H	5.4222050	-1.9687780	0.2112220
H	6.0728670	-0.6336440	1.1606280
H	5.7691290	-0.4177900	-0.5678010
C	3.4465390	-1.0156360	1.9717870
H	2.4438060	-0.6551090	2.2100350
H	4.1049270	-0.7513050	2.8029410
H	3.4097390	-2.1073340	1.9140100
C	-2.4365880	-0.0027960	-2.8321530
H	-2.3246850	1.0082530	-2.4370010
H	-3.3033290	-0.0154820	-3.4962630
H	-1.5488000	-0.2387790	-3.4223690
C	-2.8213910	-2.4511600	-2.2706520
H	-3.0011640	-3.1716590	-1.4700380
H	-1.9342570	-2.7649720	-2.8262370
H	-3.6757110	-2.4698330	-2.9512060

Conf. 3. SCF –1285.21128145 hartree

Symbol	X	Y	Z
O	-2.9321470	-0.9306190	2.4179720
O	-4.3378880	0.4287640	1.2814670
O	-1.1062860	0.0236730	-1.5396310
O	-1.1423270	-1.0172680	0.4700500
O	-0.2811770	-2.1352600	-1.3111220
O	0.3924870	1.5049600	0.5037720
O	2.5685180	0.9641530	0.0723720
N	0.9689180	-0.4663120	-0.4971240
C	-3.8539750	-0.9337830	3.5281640
H	-4.8162300	-1.3423380	3.2197970
H	-3.9888100	0.0779780	3.9105030
H	-3.3934880	-1.5678830	4.2808950
C	-3.2966570	-0.2027630	1.3480510
C	-2.3017100	-0.2619280	0.2930020
C	-2.2659560	0.3528310	-0.9023850
C	-3.1982160	1.2799720	-1.5996670
H	-4.0520050	1.4058500	-0.9331170
C	-0.3789530	-0.9019130	-0.7026240
C	1.0266900	-2.2749830	-1.9090280
H	0.9956550	-1.9039320	-2.9351930
H	1.2693910	-3.3343490	-1.9099370
C	1.9515080	-1.4258020	-1.0275910
H	2.6721260	-0.8906330	-1.6458570
C	1.2467650	0.7540660	0.0744780
C	3.1698710	2.1676620	0.6982810
C	4.6613280	1.9331130	0.4805780
H	4.9795630	1.0094870	0.9682940
H	5.2293900	2.7639920	0.9034300

H	4.8876480	1.8636690	-0.5853560
C	2.8344950	2.1935810	2.1873970
H	1.7723200	2.3640970	2.3535200
H	3.3969950	2.9990260	2.6650340
H	3.1233310	1.2503830	2.6569310
C	2.7008580	3.4209870	-0.0360750
H	2.8979470	3.3286580	-1.1066570
H	3.2565970	4.2831500	0.3400260
H	1.6375830	3.5963380	0.1166620
C	2.7128950	-2.1880350	0.0740510
H	3.2000700	-1.4179590	0.6789910
C	3.8103510	-3.0552050	-0.5520060
H	3.3897850	-3.8552400	-1.1691860
H	4.4127950	-3.5263730	0.2281220
H	4.4786380	-2.4599840	-1.1807090
C	1.7973660	-3.0047430	0.9905560
H	1.0280130	-2.3804630	1.4491320
H	2.3839660	-3.4591130	1.7928110
H	1.2989700	-3.8130860	0.4476760
C	-2.5312360	2.6498550	-1.8211560
H	-2.2148490	3.0912410	-0.8741160
H	-3.2398710	3.3291980	-2.3001350
H	-1.6550960	2.5570920	-2.4670000
C	-3.6846270	0.6678660	-2.9255990
H	-4.1710530	-0.2960560	-2.7601260
H	-2.8513280	0.5196770	-3.6163780
H	-4.4046850	1.3398550	-3.3977910

H	-1.6315220	2.3952670	1.3319620
C	-0.0528960	3.4819130	-1.5598790
H	0.9801140	3.5476620	-1.8974080
H	-0.4916880	4.4822150	-1.5784860
H	-0.6134310	2.8497680	-2.2515870
C	0.7092570	3.7161230	0.8536280
H	0.6834800	3.2379000	1.8355240
H	0.2934820	4.7214820	0.9523580
H	1.7430710	3.7962960	0.5233000
C	3.7997720	-1.0052530	0.8590780
H	3.2802800	-1.7095150	1.5205500
C	3.9166790	0.3321210	1.5948400
H	4.4109330	1.0824490	0.9733280
H	4.5053170	0.2008520	2.5064280
H	2.9388720	0.7215130	1.8846050
C	5.1908720	-1.5669190	0.5404440
H	5.1447950	-2.5316750	0.0290830
H	5.7645650	-1.7074030	1.4594070
H	5.7462570	-0.8736160	-0.0995540
C	-2.4898700	0.1979900	-2.7663080
H	-2.3452960	1.1947370	-2.3469880
H	-3.3541180	0.2316430	-3.4329060
H	-1.6088510	-0.0546590	-3.3597490
C	-2.9860270	-2.2428680	-2.2666630
H	-3.1949850	-2.9753030	-1.4842060
H	-2.1179250	-2.5830440	-2.8366060
H	-3.8441920	-2.2027000	-2.9413980

Conf. 4. SCF –1285.211559 hartree

Symbol	X	Y	Z
O	-3.7209720	-0.6649020	1.2549240
O	-2.1552360	-0.7092790	2.8869420
O	-0.3408060	-1.0152530	-1.2435650
O	-0.1590830	-1.0489440	1.0111720
O	1.1937410	-2.4110750	-0.2109750
O	2.4414590	1.8590310	-0.7917020
O	0.2853810	1.5031990	-0.1371800
N	1.6829910	-0.2282340	-0.2746510
C	-4.7434580	-0.4697360	2.2538810
H	-4.5677410	0.4576370	2.7990640
H	-4.7592110	-1.3092570	2.9487400
H	-5.6768440	-0.4154310	1.7000320
C	-2.4569410	-0.7593090	1.7088790
C	-1.4955850	-0.9187080	0.6333220
C	-1.5911400	-0.9069130	-0.7090270
C	-2.7336760	-0.8458110	-1.6649090
H	-3.6088610	-0.5563300	-1.0840950
C	0.6012630	-1.1641610	-0.1586220
C	2.4589380	-2.2819520	-0.9023400
H	2.2899310	-2.3158630	-1.9808470
H	3.0787780	-3.1192410	-0.5949970
C	2.9725800	-0.9159550	-0.4411940
H	3.5447090	-0.4122760	-1.2210530
C	1.5321630	1.1330610	-0.4386410
C	-0.1366320	2.9253180	-0.1415420
C	-1.5835150	2.8372260	0.3348700
H	-2.1807000	2.2268560	-0.3433530
H	-2.0167360	3.8384630	0.3755670

Conf. 5. SCF –1285.2115845 hartree

Symbol	X	Y	Z
O	-2.1223830	-0.8390490	2.7302600
O	-3.8090920	-0.7489470	1.2252850
O	-0.3895460	-0.9697700	-1.3765990
O	-0.2410570	-1.0873440	0.8804210
O	1.1491000	-2.3828900	-0.3728190
O	2.3418620	1.9252380	-0.7642780
O	0.1847390	1.5107990	-0.1485590
N	1.6081290	-0.1930220	-0.3460500
C	-3.0848230	-0.7060930	3.7973490
H	-3.7946070	-1.5328040	3.7720520
H	-3.6180570	0.2404450	3.7089140
H	-2.5015730	-0.7310950	4.7138460
C	-2.6205220	-0.8434840	1.4818090
C	-1.5737940	-0.9617330	0.4845030
C	-1.6476400	-0.9007540	-0.8569190
C	-2.7880180	-0.8138100	-1.8110710
H	-3.6673570	-0.5764250	-1.2111360
C	0.5387250	-1.1471880	-0.2820970
C	2.4214070	-2.2102290	-1.0414650
H	2.2671420	-2.2058530	-2.1226660
H	3.0490920	-3.0497980	-0.7571660
C	2.9094850	-0.8558060	-0.5217900
H	3.4843340	-0.3148200	-1.2741400
C	1.4396930	1.1719710	-0.4527130
C	-0.2580040	2.9255270	-0.0938180
C	-1.7062640	2.7958760	0.3684920
H	-2.2903250	2.2058730	-0.3385430
H	-2.1547890	3.7879100	0.4487430

H	-1.7533760	2.3115660	1.3457820
C	-0.1729800	3.5440150	-1.4861720
H	0.8612630	3.6377850	-1.8133300
H	-0.6251780	4.5382750	-1.4650580
H	-0.7204340	2.9348760	-2.2083090
C	0.5693370	3.6844140	0.9411010
H	0.5455990	3.1622050	1.9004190
H	0.1364250	4.6774120	1.0822110
H	1.6036160	3.7961530	0.6217250
C	3.7212500	-0.9847480	0.7849720
H	3.2049940	-1.7239600	1.4098950
C	3.8064990	0.3225680	1.5768360
H	4.2962610	1.1059620	0.9938280
H	4.3853190	0.1628710	2.4901880
H	2.8186970	0.6838780	1.8691390
C	5.1253350	-1.5105230	0.4621040
H	5.1015250	-2.4529320	-0.0908250
H	5.6891000	-1.6815030	1.3820370
H	5.6777870	-0.7817570	-0.1398780
C	-2.5676300	0.2938360	-2.8534440
H	-2.4459660	1.2692060	-2.3801150
H	-3.4315020	0.3444490	-3.5194630
H	-1.6803610	0.0936380	-3.4577790
C	-3.0109510	-2.1808670	-2.4883910
H	-3.2001320	-2.9601900	-1.7472770
H	-2.1375490	-2.4689910	-3.0786370
H	-3.8724850	-2.1233410	-3.1575100

Conf. 6. SCF –1285.2099080 hartree

Symbol	X	Y	Z
O	-3.0622300	-1.3448210	2.1939440
O	-4.3851460	0.2550360	1.2964100
O	-1.0941300	0.2389580	-1.4846730
O	-1.2208050	-1.1466520	0.3005130
O	-0.3423680	-1.9497640	-1.6401060
O	0.4003560	1.3702310	0.6830460
O	2.5541220	0.9038350	0.0927380
N	0.9356090	-0.5037290	-0.5047200
C	-4.0155030	-1.5138770	3.2640500
H	-4.9810430	-1.8243280	2.8648670
H	-4.1286240	-0.5834040	3.8202690
H	-3.5978610	-2.2900310	3.8996390
C	-3.3695880	-0.4196360	1.2683720
C	-2.3476290	-0.3253790	0.2416800
C	-2.2567030	0.4954250	-0.8195360
C	-3.1324180	1.5738990	-1.3525830
H	-3.9966510	1.6155570	-0.6889130
C	-0.4217390	-0.8452710	-0.8147310
C	0.9802480	-1.9974770	-2.2263480
H	1.0071340	-1.3666890	-3.1174430
H	1.1741830	-3.0319150	-2.4952220
C	1.8798040	-1.4576670	-1.1124280
H	2.7331240	-0.9118030	-1.5127470
C	1.2390340	0.6701600	0.1492940
C	3.1730190	2.0524300	0.8005440
C	4.6529650	1.8715650	0.4779520
H	5.0163430	0.9147670	0.8579540
H	5.2307150	2.6723290	0.9435750
H	4.8175410	1.9062910	-0.6009470
C	2.9229320	1.9288020	2.3015740
H	1.8683820	2.0543030	2.5408140
H	3.4931130	2.7020940	2.8214770
H	3.2587920	0.9549340	2.6640800
C	2.6451040	3.3619600	0.2199400
H	2.7806550	3.3772630	-0.8640360
H	3.2103970	4.1939530	0.6465040
H	1.5903140	3.5009630	0.4491200
C	2.3522700	-2.5649110	-0.1453940
H	1.4899190	-3.2171020	0.0396590
C	2.8320180	-2.0261210	1.2044800
H	3.6945070	-1.3672270	1.0820350
H	3.1278800	-2.8575720	1.8491950
H	2.0476450	-1.4695170	1.7205610
C	3.4566310	-3.3904260	-0.8167330
H	3.1413150	-3.8076200	-1.7762440
H	3.7525210	-4.2238480	-0.1755110
H	4.3424530	-2.7717910	-0.9937350
C	-2.4037710	2.9296230	-1.3098370
H	-2.0850990	3.1728320	-0.2945530
H	-3.0743690	3.7166940	-1.6619800
H	-1.5206760	2.9179970	-1.9527210
C	-3.6104980	1.2345020	-2.7758080
H	-4.1428660	0.2809050	-2.7964020
H	-2.7666450	1.1735910	-3.4670540
H	-4.2878080	2.0142880	-3.1311070

Table S25. Cartesian coordinates of intermediate **I’** and transition state **TS’** obtained from (*S*)-**9a** optimized at B3LYP-D3BJ/6-31+G(d)/PCM level.

I’ SCF –840.902890 hartree, 0 imaginary freq.

Symbol	X	Y	Z
C	-1.4172110	0.3975070	-0.2563910
C	-1.1512070	1.7805410	-0.1795080
O	0.0077870	2.2167680	-0.4295960
C	0.8351090	-0.1741880	-0.2265280
O	-0.2983270	-0.3567920	-0.7912830
O	0.9831550	0.0372790	1.0501920
C	2.4169730	0.2446470	1.2962800
H	2.6626020	-0.2724650	2.2222730
H	2.5720280	1.3204430	1.3869880
C	3.0888420	-0.3657980	0.0517220
O	1.9171670	-0.3600260	-0.9115030
C	-2.2522510	2.7372480	0.2258160
H	-3.1390040	2.6218160	-0.4050130
H	-2.5685690	2.5488500	1.2581570
H	-1.8710430	3.7593330	0.1470980
C	-2.6021660	-0.3478330	-0.0044100

O	-3.6817850	0.1294530	0.3642820
O	-2.4366490	-1.6911120	-0.2061650
C	-3.5971880	-2.5061410	0.0272670
H	-3.2793590	-3.5279110	-0.1875610
H	-3.9267380	-2.4269730	1.0675300
H	-4.4160650	-2.2202100	-0.6395080
C	4.1690590	0.5089020	-0.5435650
H	3.7886420	1.5128450	-0.7540180
H	4.5623740	0.0648920	-1.4629440
H	4.9880810	0.5905680	0.1797030
C	3.4915410	-1.8171680	0.2313730
H	4.3317830	-1.8631580	0.9325670
H	3.8084880	-2.2465710	-0.7237060
H	2.6670100	-2.4123230	0.6386920

TS’ SCF –840.881271 hartree, 1 imaginary freq.

Symbol	X	Y	Z
C	1.5507010	0.3800860	-0.0062800
C	1.7532740	1.7861220	0.0066080
O	0.8140260	2.6123540	-0.0003120
C	-0.9799980	0.1183270	-0.0792830
O	0.2345010	-0.1961000	-0.0188600
O	-1.5130160	1.3083620	-0.0430200
C	-2.9589980	1.1584260	-0.2663750
H	-3.1494180	1.4401230	-1.3034970
H	-3.4619800	1.8336790	0.4238900
C	-3.2191150	-0.3289660	0.0193420
O	-1.8329490	-0.8770670	-0.1717700
C	3.1849370	2.3029880	0.0286680
H	3.7502680	1.9646210	-0.8454550
H	3.1384160	3.3959630	0.0359600
H	3.7281510	1.9517430	0.9116950
C	2.5432260	-0.6570730	-0.0013150
O	3.7643520	-0.4710870	0.0143580
O	2.0185720	-1.9193300	-0.0160170
C	2.9761380	-2.9904130	-0.0093370
H	2.3817990	-3.9058150	-0.0240310
H	3.6190930	-2.9462820	-0.8934220
H	3.5928530	-2.9582010	0.8937840
C	-3.6058780	-0.6024650	1.4627320
H	-2.9164730	-0.1114310	2.1587120
H	-3.6070790	-1.6789520	1.6589390
H	-4.6136050	-0.2117710	1.6401090
C	-4.1227850	-0.9950680	-0.9955790
H	-5.1192520	-0.5448300	-0.9285570
H	-4.2127170	-2.0654890	-0.7863480
H	-3.7429540	-0.8521910	-2.0118910

Table S26. Cartesian coordinates of intermediates **IV’** and extended-**IV’** obtained from (*S*)-**10a** optimized at B3LYP-D3BJ/6-31+G(d)/PCM level.

SCF -1088.210712 hartree, 1 imaginary freq.

Symbol	X	Y	Z
C	-1.7300920	-0.8767530	0.2122970
C	-1.1983060	-1.3286550	1.3629850
O	-0.0527410	-2.0644790	1.0094010
C	0.0615250	-1.9966380	-0.3026800
O	-0.9101830	-1.3136180	-0.8506090
O	0.9177110	-2.5906780	-1.0143460
C	2.2522500	-2.9842620	-0.4344370
H	2.5619500	-3.7977710	-1.0891900
H	2.0712560	-3.3417050	0.5797710
C	3.1655010	-1.7683050	-0.4942960
H	3.2738040	-1.4678810	-1.5466400
H	4.1406470	-2.1562020	-0.1574060
N	2.7248610	-0.6991570	0.3695470
C	-1.5140690	-1.2169740	2.7940190
H	-2.4310970	-0.6395580	2.9196570
H	-1.6464620	-2.2134780	3.2313840
H	-0.6922940	-0.7138910	3.3176230
C	-2.9109460	-0.0490640	-0.0717330
O	-3.6312470	0.3728260	0.8155250
O	-3.0640930	0.1576310	-1.3759580
C	-4.1951340	0.9804540	-1.7668080
H	-4.1450710	1.0277460	-2.8537080
H	-5.1253970	0.5102090	-1.4407140
H	-4.0952240	1.9765130	-1.3302490
C	2.1557010	0.3323420	-0.2516580
O	1.9287450	0.5053220	-1.4733750
O	1.7888150	1.3029250	0.6797920
C	1.2201660	2.5830830	0.2815140
C	-0.1659550	2.3924700	-0.3423940
H	-0.1069990	1.7937180	-1.2532280
H	-0.6036880	3.3682460	-0.5862770
H	-0.8300760	1.8958240	0.3734700
C	2.1665760	3.3506470	-0.6481380
H	1.7981210	4.3748110	-0.7841450
H	3.1688710	3.4065640	-0.2055780
H	2.2411670	2.8679250	-1.6240970
C	1.0878770	3.3298020	1.6120890
H	0.6347640	4.3145630	1.4501680
H	2.0707490	3.4725080	2.0762770
H	0.4543320	2.7676350	2.3080450

IV' ext. conf. SCF –1088.202225 hartree, 0 imaginary freq.

Symbol	X	Y	Z
C	3.4084000	0.3246000	-0.0840000
C	2.3314000	1.1278000	-0.1556000
O	1.2200000	0.2819000	-0.3556000
C	1.6742000	-0.9521000	-0.3911000
O	2.9715000	-1.0125000	-0.2348000
O	0.9823000	-1.9887000	-0.5701000
C	-0.5498000	-1.8805000	-0.6135000
H	-0.8103000	-2.7962000	-1.1389000
H	-0.7798000	-0.9963000	-1.2062000
C	-1.1161000	-1.8181000	0.7966000
H	-0.6949000	-0.9425000	1.3159000
H	-0.8124000	-2.7206000	1.3399000
N	-2.5628000	-1.7785000	0.6864000
C	2.0696000	2.5716000	-0.0798000
H	3.0111000	3.1053000	0.0576000
H	1.5856000	2.9135000	-1.0021000
H	1.4014000	2.7873000	0.7624000
C	4.8397000	0.6082000	0.1052000
O	5.2568000	1.7451000	0.2316000
O	5.5645000	-0.5057000	0.1146000
C	6.9981000	-0.3389000	0.2864000
H	7.4006000	-1.3507000	0.2774000
H	7.4027000	0.2475000	-0.5414000
H	7.2020000	0.1519000	1.2404000
C	-3.0308000	-0.5620000	0.4054000
O	-2.3927000	0.5027000	0.2239000
O	-4.4201000	-0.5787000	0.3414000
C	-5.1886000	0.5932000	-0.0546000
C	-4.8166000	1.0396000	-1.4729000
H	-3.7940000	1.4207000	-1.5105000

11. References and Notes

(1) Baral, E. R.; Lee, J. H.; Kim, J. G. Diphenyl Carbonate: A Highly Reactive and Green Carbonyl Source for the Synthesis of Cyclic Carbonates. *J. Org. Chem.* **2018**, *83*, 11768-11776.
(2) Burk, R. M.; Roof, M. B. A safe and efficient method for conversion of 1,2- and 1,3-diols to cyclic carbonates utilizing triphosgene. *Tetrahedron Lett.* **1993**, *34*, 395-398.
(3) Pertschi, R.; Brun, E.; de Aguirre, A.; Guénée, L.; Poblador-Bahamonde, A. I.; Lacour, J. Spirocyclic Amide Acetal Synthesis by [CpRu]-Catalyzed Condensations of α-Diazo-β-Ketoesters with γ-Lactams. *Helv. Chim. Acta.* **2021**, *104*, e2100122.
(4) Davies, H. M. L.; Cantrell Jr, W. R.; Romines, K. R.; Baum, J. S. Synthesis of Furans via Rhodium(II) Acetate-Catalyzed Reaction of Acetylenes with α-Diazocarbonyls: Ethyl 2-Methyl-5-Phenyl-3-Furancarboxylate. In *Organic Syntheses*, 2003; pp 93-93.
(5) Achard, T.; Egger, L.; Tortoreto, C.; Guénée, L.; Lacour, J. Preparation and structural characterization of [CpRu(1,10-phenanthroline)(CH_3CN)][X] and precursor complexes (X= PF_6, BAr_F, TRISPHAT-N). *Helv. Chim. Acta* **2020**, *103*, e2000190.
(6) Green, S. P.; Wheelhouse, K. M.; Payne, A. D.; Hallett, J. P.; Miller, P. W.; Bull, J. A. Thermal Stability and Explosive Hazard Assessment of Diazo Compounds and Diazo Transfer Reagents. *Org. Process Res. Dev.* **2020**, *24*, 67-84.
(7) (a) Trapp, O.; Schoetz, G.; Schurig, V. Determination of enantiomerization barriers by dynamic and stopped-flow chromatographic methods. *Chirality* **2001**, *13*, 403-414. (b) D'Acquarica, I.; Gasparrini, F.; Pierini, M.; Villani, C.; Zappia, G. Dynamic HPLC on chiral stationary phases: A powerful tool for the investigation of stereomutation processes. *J. Sep. Sci.* **2006**, *29*, 1508-1516. (c) Trapp, O. Unified Equation for Access to Rate Constants of First-Order Reactions in Dynamic and On-Column Reaction Chromatography. *Anal. Chem.* **2006**, *78*, 189-198. (d) Trapp, O. The unified equation for the evaluation of degenerated first-order reactions in dynamic electrophoresis. *Electrophoresis* **2006**, *27*, 2999-3006. (e) Trapp, O. The unified equation for the evaluation of first order reactions in dynamic electrophoresis. *Electrophoresis* **2006**, *27*, 534-541. (f) Trapp, O. A novel software tool for high throughput measurements of interconversion barriers: DCXplorer. *J. Chromatogr. B* **2008**, *875*, 42-47. (g) Trapp, O.; Bremer, S.; Weber, S. K. Accessing reaction rate constants in on-column reaction chromatography: an extended unified equation for reaction educts and products with different response factors. *Anal. Bioanal. Chem.* **2009**, *395*, 1673-1679.
(8) *Gaussian 16 Rev. C.01*; Wallingford, CT, 2016. (accessed.
(9) Bruhn, T.; Schaumlöffel, A.; Hemberger, Y.; Pescitelli, G. SpecDis version 1.71. *Berlin, Germany* **2017**.
(10) Resource for Biocomputing, Visualization, and Informatics at the University of California, San Francisco, with support from National Institutes of Health R01-GM129325 and the Office of Cyber Infrastructure and Computational Biology, National Institute of Allergy and Infectious Diseases.
(11) Pettersen, E. F.; Goddard, T. D.; Huang, C. C.; Meng, E. C.; Couch, G. S.; Croll, T. I.; Morris, J. H.; Ferrin, T. E. UCSF ChimeraX: Structure visualization for researchers, educators, and developers. *Protein Sci.* **2021**, *30*, 70-82.
(12) Johnson, E. R.; Keinan, S.; Mori-Sánchez, P.; Contreras-García, J.; Cohen, A. J.; Yang, W. Revealing Noncovalent Interactions. *J. Am. Chem. Soc.* **2010**, *132*, 6498-6506.
(13) (a) Lu, T. A comprehensive electron wavefunction analysis toolbox for chemists, Multiwfn. *J. Chem. Phys.* **2024**, *161*, 082503. (b) Lu, T.; Chen, F. Multiwfn: A multifunctional wavefunction analyzer. *J. Comput. Chem.* **2012**, *33*, 580-592.

(14) (a) Achard, T.; Tortoreto, C.; Poblador-Bahamonde, A. I.; Guénée, L.; Bürgi, T.; Lacour, J. CpRu-catalyzed carbene insertions into epoxides: 1,4-dioxene synthesis via S_N1-like chemistry with retention of configuration. *Angew. Chem. Int. Ed.* **2014**, *53*, 6140–6144. (b) Pertschi, R.; Brun, E.; de Aguirre, A.; Guénée, L.; Poblador-Bahamonde, A. I.; Lacour, J. Spirocyclic Amide Acetal Synthesis by [CpRu]-Catalyzed Condensations of α-Diazo-β-Ketoesters with γ-Lactams. *Helv. Chim. Acta* **2021**, *104*, e2100122. (c) Nikolova, Y.; Fabri, B.; Moneva Lorente, P.; Guarnieri-Ibáñez, A.; de Aguirre, A.; Soda, Y.; Pescitelli, G.; Zinna, F.; Besnard, C.; Guénée, L.; Moreau, D.; Di Bari, L.; Bakker, E.; Poblador-Bahamonde, A. I.; Lacour, J. Chemo and Regioselective Multiple $C(sp^2)$−H Insertions of Malonate Metal Carbenes for Late-Stage Functionalizations of Azahelicenes. *Angew. Chem. Int. Ed.* **2022**, *61*, e202210798.
(15) Cremer, D.; Pople, J. A. General definition of ring puckering coordinates. *J. Am. Chem. Soc.* **1975**, *97*, 1354-1358.
